# Genome-wide identification and comparison of legume *MLO* gene family

**DOI:** 10.1038/srep32673

**Published:** 2016-09-06

**Authors:** Nicolas Rispail, Diego Rubiales

**Affiliations:** 1 Institute for Sustainable Agriculture, CSIC, Avda. Menéndez Pidal s/n, 14004 Córdoba, Spain

## Abstract

MLO proteins are highly conserved proteins with seven trans-membrane domains. Specific *MLO* genes have been linked to plant disease susceptibility. Others are involved in plant reproduction and in root thigmomorphogenesis. Functions of the remaining *MLOs* are still unknown. Here we performed a genome-wide survey of the *MLO* family in eight legume species from different clades of the Papillionoideae sub-family. A total of 118 *MLO* sequences were identified and characterized. Their deduced protein sequences shared the characteristics of MLO proteins. The total number of *MLO* genes per legume species varied from 13 to 20 depending on the species. Legume *MLOs* were evenly distributed over their genomes and tended to localize within syntenic blocks conserved across legume genomes. Phylogenetic analysis indicated that these sequences clustered in seven well-defined clades. Comparison of MLO protein sequences revealed 34 clade-specific motifs in the variable regions of the proteins. Comparative analyses of the *MLO* family between legume species also uncovered several evolutionary differences between the tropical legume species from the Phaseoloid clades and the other legume species. Altogether, this study provides interesting new features on the evolution of the *MLO* family. It also provides valuable clues to identify additional *MLO* genes from non-sequenced species.

Grain and forage legumes are among the most important crops worldwide for both animal and human consumptions^1^. They are also important players of sustainable agriculture^2^. Their capacity to fix atmospheric nitrogen allows them to grow in poor soils without application of nitrogenous fertilizers. As a consequence, they contribute to reduce both fossil energy requirement and greenhouse gas emission^3^. Inclusion of legume crops in rotation impacts positively on subsequent crop production^2^. However, legume yield is constantly threatened by fungal diseases^4^.

Powdery mildew emerged as one of the most widespread and damaging legume diseases^4^. One of the most efficient and durable powdery mildew resistance mechanisms was originally found in barley^5^,^6^. Lines carrying homozygous recessive alleles at the *Mlo* locus showed an efficient penetration resistance to this pathogen^5^. *mlo*-based resistance is one of the few examples of monogenic traits that confer broad spectrum resistance in the field^6^,^7^,^8^. Recently, *mlo*-based resistance has been identified in other crops including pea (*er1*)^9^,^10^and tomato (*ol*-2)^11^. Penetration resistance was also detected in other legumes including, *Medicago truncatula*^12^ and *Lathyrus belinensis*^13^. Although the genetic base controlling the resistance in these species is not known, their phenotypes are reminiscent to that of *mlo-*based resistant accessions. It is thus possible that they arose from mutations in one *MLO* gene. Other species may, thus, contain natural *mlo* mutants that could be very useful to breed crops for resistance to powdery mildew.

The barley MLO is a seven trans-membrane domain protein that localizes at cell plasma membrane^5^. This gene belongs to a highly conserved family found in both monocots and eudicots^14^. To date, the total number of *MLO* genes identified varied from 11 to 31 according to the plant species^15^,^16^ (Table 1). The biological function of most *MLO* genes remains largely unknown. Phylogenetic analyses classified these genes in 6 to 8 clades^14^,^17^. All *MLO* genes with a function in powdery mildew susceptibility clustered in clades IV and V^14^,^18^. A clade V *MLO* gene, from pepper, *CaMLO2*, has also been associated with susceptibility to bacterial and oomycete pathogens, and to drought^19^,^20^. In addition, the expression of some *Lathyrus sativus MLO* transcripts were induced shortly after rust infection in resistant genotypes, which might suggest their involvement in rust resistance^21^. Although the exact role of clade V *MLOs* is still unclear, they might interfere with the plant immune response to stresses. This is similar to the *Lr34* resistance gene that protects wheat by controlling the induction of multiple defense pathways^7^,^22^. Apart for the known functions of clade IV and V*MLOs*, two clade I *MLO* genes from *A. thaliana* (*AtMLO4* and *AtMLO11*) were found to play an important role in root thigmomorphogenesis^23^,^24^. Two clade III genes, *AtMLO7* and *OsMLO12,* were also shown to be required for normal pollen tube perception and pollen hydration, respectively^25^,^26^, which suggested a role of clade III *MLOs* in plant reproduction^14^. The MLO family may thus play a wider range of functions than initially thought. Isolating and characterizing new *MLOs* from other plant species is thus a promising approach to get new insights on this highly conserved family.

*MLO* genes have been intensively studied in some plant species. However, little is known about the *MLO* members of *Fabaceae*. Here, we performed the genome-wide characterization of the *MLO* gene family in eight legume species belonging to the major clades of the Papillionoideae sub-family (Genistoid, Dalbergioid, Phaseoloid and Galegoid). This included three species from the Galegoid clade (the temperate legumes, barrel medic, chickpea and pea), one from the Genistoid clade (narrow-leaf lupin), one from the Dalbergioid clade (peanut) and three from the Phaseoloid clade that regroups the tropical legumes (pigeon pea, common bean and mung bean) (Table 2). The newly identified sequences were then compared with previously identified *MLOs* to get insights about the evolution of the *MLO* family in legumes.

## Results

### Identification of legume MLOs

Datamining of the different legume genomes (Table 2) identified from 14 to 23 sequences with homology to *A. thaliana MLOs* (Supplementary Table S1). In most genomes, several hits were predicted to encode for truncated proteins. This included the *M. truncatula* sequences *MtMLO12* and *MtMLO16* (Supplementary Table S1). Most of these truncated versions were located close to retro-transposon-like sequences. Thus, these shorter sequences were considered pseudogenes and they were not analysed further.

The remaining sequences were confirmed as putative full length *MLOs*. This led to the identification of 14 *MLO* genes in *M. truncatula,* 13 in *Cicer arietinum*, 15 in *Lupinus angustifolius*, 20 in *Cajanus cajan*, 19 in *Phaseolus vulgaris*, 18 in *Vigna radiata* and 13 in each *Arachis* genome (Table 1 and Supplementary Table S1). Interestingly, the sequences SSV2N, from *A. duranensis,* and MQE1N, from *A. ipaensis,* had no counterpart in the second *Arachis* genome (Supplementary Table S1). The peanut genome may, thus, contain 14 potential *MLO* members. They have been named *ArMLO1* to *ArMLO14* (Table 1 and Supplementary Table S1). To avoid redundancy, only one sequence for each *Arachis MLO* orthologue was used in the analysis.

Since the pea genome has not been sequenced yet, we used the large transcriptomic resources available to search for potential *MLO* genes in this species. We identified several pea transcripts showing homology with 11 *MtMLO* sequences. This suggested the presence of, at least, 11 potential *MLOs* in pea (data not shown). In addition to *PsMLO1,* three full length *MLO* genes could be reconstructed. These sequences, named *PsMLO2, PsMLO3* and *PsMLO4,* showed high similarity to *MtMLO9, MtMLO11* and *MtMLO15,* respectively (Supplementary Tables S1).

### Organization and distribution of legume MLOs

The gene characteristics are summarized in Supplementary Table S1. Large variations in gene size were detected within and between legume species. The longest gene, *ArMLO13,* covered a genomic region of 28.05 kb, although this might be due to assembly errors. The mean genomic length of the other legume *MLOs* varied from 4.41 to 6.62 kb. The length of their coding regions varied from 1.63 to 1.65 kb on average distributed on 12 to 17 exons. Accordingly, the mean protein size varied from 539 to 547 amino acids (Supplementary Table S1).

One to four *MLO* genes were detected on almost all legume chromosomes indicating an even distribution over legume genomes. In addition, we observed that physically close *MLO* pairs, in any given species, had orthologous pairs in the corresponding chromosome of other legume species (Supplementary Table 1). For instance, the orthologous sequences of *MtMLO5*, *MtMLO*8 and *MtMLO*9, from *M. truncatula* chromosome 3, are located on the same chromosome in *P. vulgaris*, *Cicer arietinum*, *V. radiata*, *Cajanus cajan* and *Arachis* spp. (Supplementary Table S1). Similar situation was found for the orthologues of *MtMLO2* and *MtMLO6*, located in *M. truncatula* chromosome 5, and those of *MtMLO4* and *MtMLO7,* from chromosome 2, that were detected, in the same order, on the corresponding chromosomes of *Cicer arietinum*, *P. vulgaris* and *V. radiata,* respectively (Supplementary Table S1). This would suggest that at least some of the *MLOs* localized within syntenic blocks which are conserved across legume genomes.

### Characterization of protein and domain organization

**Figure 1:**
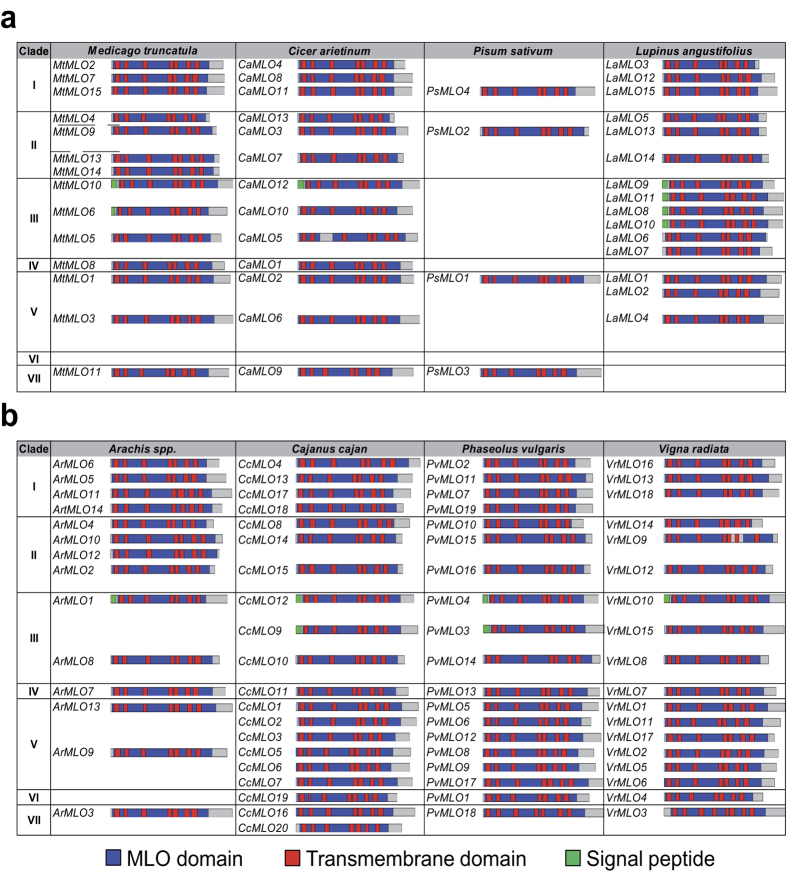
Domain organization of legume MLOs. The figure represents the domain organization of all MLO protein sequences isolated from (**a**) Genistoid and Galegoid legumes and (**b**) Dalbergioid and Phaseoloid legumes. Sequences are drawn to scale with the IBS server^51^ following Interproscan 5^29^, TMHMM^27^ and SignalP^52^ predictions (Supplementary Table S2).

MLO proteins are characterized by the presence of seven trans-membrane (TM) domains and one MLO functional domain^14^. To determine whether the legume *MLO* genes shared these typical characteristics, their deduced amino acid sequences were subjected to different prediction servers (Supplementary Table S2). Almost all sequences were predicted to contain a single MLO domain covering most of the protein length. The sole exceptions were CaMLO5 and VrMLO9 for which two separated MLO domains were predicted (Supplementary Table S3 and Fig. 1). All potential MLOs were predicted to localise within cell membranes (Supplementary Table S3).

The prediction servers used to estimate the number of TM domains (TMHMM^27^, Psort^28^ and InterProScan5)^29^ implemented different algorithms. This lead to some variations in the total number of TM domains predicted (Supplementary Table S3). Despite these small variations, all sequences, except *VrMLO3,* were predicted to contain seven TM domains. For 97 sequences, the prediction was supported by two or more servers (Supplementary Table S3 and Fig. 1). The TM domain distribution was largely similar between them and it fitted with the distribution of TM domains of typical MLO proteins (Fig. 1). Several putative MLOs were also predicted to contain a signal peptide at their N terminal region (Supplementary Table S3 and Fig. 1).

In parallel, the legume MLO sequences were subjected to the MEME suite server^30^ to identify conserved amino acid motifs and to uncover species-specific or legume-specific signatures. This identified 14 amino acid motifs common to most MLO sequences (Table 3). These motifs co-localized with the TM domains, the internal loops 2 and 3 and the calmodulin-binding region (CaMBD) (Supplementary Fig. S1). These motifs were also found in most MLO sequences from *Glycine max* and from non-legume species including *Arabidopsis thaliana*, *Cucumis sativus*, *Solanum lycopersicum* and *Vitis vinifera.* In addition, they were largely similar and overlapping with the motifs identified in previous studies^15^,^16^,^31^,^32^ (Supplementary Fig. S1).

A previous study by Elliot *et al*.^33^ identified 30 invariable amino acid residues within 38 MLO sequences. Twenty two of these residues were also invariable in legume MLOs. The other residues were also highly conserved since they only changed in one or two sequences per legume species (Table 4).

### Phylogenetic analysis of legume MLOs

The *MLO* family was previously subdivided in six to eight clades^14^,^17^. To classify the legume *MLOs*, a Neighbor-Joining (NJ) phylogenetic analysis was performed. To this aim, their deduced protein sequences were aligned with already characterized MLO sequences (Table 1 and Supplementary Fig. S2).

This analysis separated the MLO proteins into seven well-supported clades (Supplementary Fig. S2). The MLO members from clade I further clustered in two well-separated sub-clades (Ia and Ib). At least one MLO protein, from each legume species, was found in clade IV that contains the barley MLO. Several members from each legume species also clustered in clade V with the powdery mildew susceptibility genes of *Arabidopsis thaliana.* Surprisingly, only sequences from tropical legumes clustered in clade VI with AtMLO3. By contrast, the last group (clade VII) was nearly exclusively composed of legume sequences except for the tomato protein SlMLO2 (Supplementary Fig. S2).

**Figure 2:**
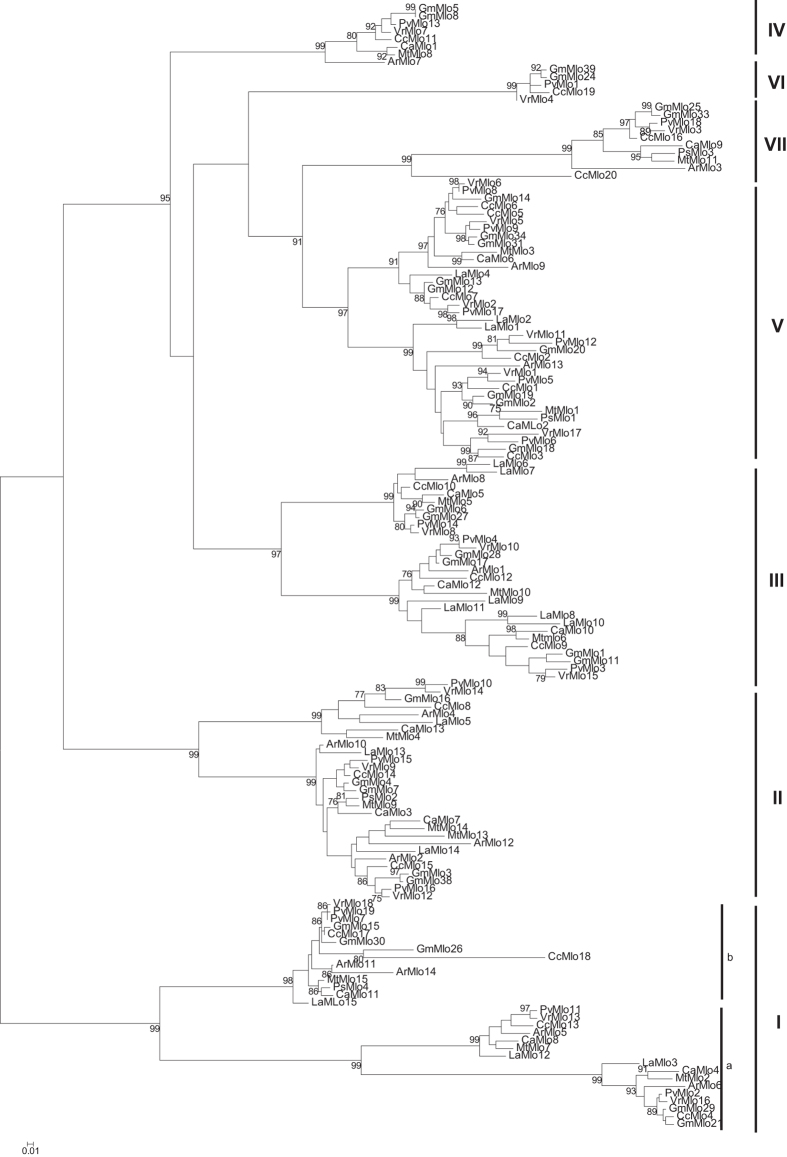
Phylogenetic relationship of legume MLOs. The phylogenetic relationship of legume MLO protein sequences was estimated with the Maximum likelihood (ML) method with MEGA6^48^ software with 1,000 bootstrap independent replicates. The tree was drawn to scale, with branch lengths measured as the number of substitutions per site. Number on a node indicates the percentage of bootstrap when higher than 75%.

To confirm this classification and to analyse further the evolution of the MLO family in legumes, a more detailed phylogenetic analysis was performed using the maximum likelihood (ML) or maximum parsimony (MP) algorithms. The two approaches (ML and MP) retrieved very similar tree topologies, thus only the ML tree is shown (Fig. 2). This approach also grouped the legume MLOs in seven clades with clade I further divided in two well-supported branches (cluster Ia and Ib) (Fig. 2). Clades I, II and III were represented by three to four MLOs per legume species. By contrast, clades IV, VI and VII were only represented by one sequence per species (Fig. 2 and Table 1). As already observed after the NJ phylogenetic analysis, clade VI only contained MLO sequences from tropical legumes including *G. max*, *Cajanus cajan*, *P. vulgaris* and *V. radiata* (Fig. 2 and Supplementary Fig. S2). The ML phylogenetic tree also showed the expansion of clade V MLOs in tropical legumes. In these Phaseoloid species, six clade V genes were detected while the other legume species had only two genes (Fig. 2 and Table 1).

### Conservation of MLO members within clades and identification of clade-specific motifs

**Figure 3:**
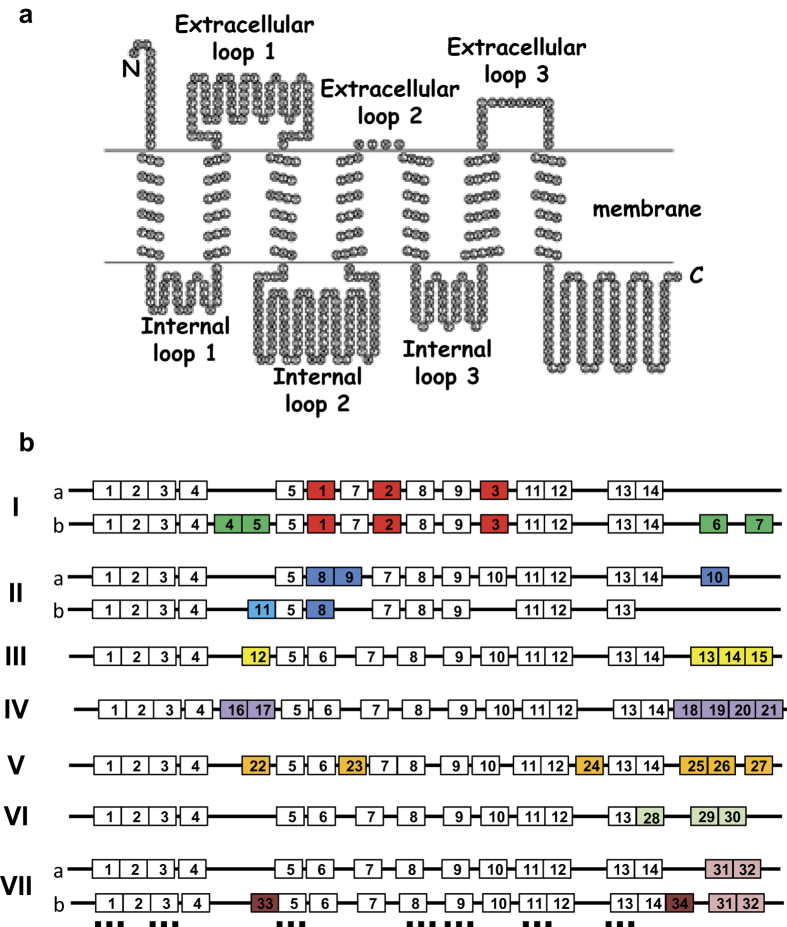
Motif organization of legume MLOs. The figure shows the predicted topology of a typical MLO protein (**a**) and the schematic organization of the common and specific motifs for each MLO clade (**b**). Common and clade-specific motifs are represented by white and colored boxes, respectively. These motifs were identified by scanning the MLO sequences with the MEME suite software^30^ (Supplemental Table S1). Common and clade-specific amino acid motifs are listed in Tables 3 and 4 respectively. Localization of transmembrane domains is shown as dashed horizontal lines.

To determine the presence of clade-specific motifs within legume MLOs, they were classified according to the phylogenetic tree and subjected to MEME (Fig. 3). In parallel, all MLO orthologues were aligned with Clustal W^34^ to visually assess their overall conservation and to locate the conserved motifs (Supplementary Figs S3 to S9). The MEME analysis revealed 34 clade-specific motifs (Fig. 3B and Table 5). According to this analysis, each clade can be recognized by the presence of two to six motifs. These motifs mostly localised within the first extracellular loop, the second intracellular loop and the C-terminal region (Fig. 3 and Supplementary Figs S3 to S9). Among the clade-specific motifs, six were specific to clade V. Three of these motifs localised at the C terminal end of the proteins. Among them, domain 25 and 27 covered the three distinctive regions identified in the MLOs associated with powdery mildew susceptibility^16^,^35^ (Supplementary Fig. S7). On the other hand, four motifs (motifs 4 to 7) could distinguish between the two sub-types of clade I MLOs. Other motifs distinguished between two sub-types within the MLO members of clades II and VII (Fig. 3B). Interestingly, one of the clade VII sub-types was characterized by the presence of two motifs (motifs 33 and 34) that were only detected in Phaseoloid species (Fig. 3 and Supplementary Fig. S9). Many of these motifs were also found in non-legume species including *Vitis vinifera, Cucumis sativus* and *Solanum lycopersicum.* This includes all clade V-specific motifs. Nevertheless, 12 motifs (motifs 5, 10, 11, 14, 15, 16, 19, 20, 21, 29, 30 and 32) were only found in legume sequences (Table 5).

On the other hand, the level of conservation of specific amino acid residues was also compared between clades (Table 4 and Supplementary Table S4). This comparison indicated that five of the 30 residues, previously described as invariable^33^, were modified on a clade specific basis (Table 4). For instance, the barley E_135_ residue was systematically changed to a D in clade VI sequences. Similarly, the Y_243_ and F_135_ were changed, in clade VII sequences, to F and S, respectively. Most of these punctual changes (three out of five punctual changes) were detected in clade VII. In the other clades, these amino acid positions remained invariable, as previously described^33^ (Table 4).

We also challenged the specificity of the 73 amino acids that discriminated between the powdery mildew susceptibility MLO of monocots and eudicots, according to Appiano *et al*.^18^ (Supplementary Table S4). As indicated previously, these sequences belonged to two different clades (clade IV and V). Thus we aimed not only to validate these specific changes but also to determine whether they could discriminate between monocot and eudicot sequences or between clades. For this, we aligned all clade IV and V legume sequences with the sequences included in Appiano *et al*.^18^ (Supplementary Table S4). Thirty six of these amino acid positions differed between clade IV and V sequences. For instance, the monocot valine and serine residues at position 32 and 145, were also found in clade IV legume sequences (Supplementary Table 4). However, they were systematically changed to isoleucine and glycine, in clade V sequences (Supplementary Table 4). These 36 amino acid positions could be instrumental to discriminate between clades IV and V (Supplementary Table S4). In addition, 17 amino acid positions discriminated between monocots and eudicot sequences. These residues were found unchanged in all eudicot sequences from clades IV and V, but, differed, within clade IV, between monocot and eudicot sequences. This is the case for the proline and leucine residues found at position 234 and 271. While these residues were found unchanged in all clade IV legume sequences, they were replaced by a glutamine and a phenylalanine residue in the monocot sequences (Supplementary Table 4). Interestingly, four additional residues varied not only between clade IV and V legume sequences, but also, between monocot and eudicot sequences within clade IV (Supplementary Table 4). For instance the S_325_ residue found in monocot clade IV sequences was modified to asparagine in clade IV legume sequences whereas it was changed to glycine in clade V sequences (Supplementary Table 4). The remaining 16 variable positions did not follow any distinctive pattern.

## Discussion

MLO is a large protein family highly conserved across plant kingdom. Apart from the well-documented role of some *MLOs* in powdery mildew susceptibility, the biological functions of *MLOs* remain largely unknown^14^. Besides providing hints on their potential functions, studying the diversification and multiplication of *MLOs* in a given species may give clues on its genome evolution. Thus we performed a genome-wide characterisation of the *MLO* family in eight legume species belonging to different clades and ecological habitats.

Mining legume genomic databases allowed the identification and characterization of 118 *MLO s*equences. The total number of *MLO* sequences varied from 13 in chickpea to 20 in pigeon pea (Table 1 and Supplementary Table S1). This is broadly similar to the situation found in other eudicot species that demonstrated the presence of 15 *MLO* genes in *A. thaliana*^36^, 17 in grapevine^37^ and tomato^31^, 14 in cucumber^32^, 18 in strawberry^17^ or 19 in peach^17^. The highest number of *MLOs* was identified in soybean with 31 full length *MLOs*^15^ (Table 1). The phylogenetic analysis showed, in most cases, a pair of soybean *MLO* genes clustering together, for any given *MLO* orthologue (Fig. 2). Thus the large *MLO* expansion in soybean is likely consequence of its recent genome duplication^15^. We also detected the presence of shorter truncated sequences with homology to *MLO* genes. Since most of them were close to retrotransposon-like sequences, we concluded that they were inactive pseudogenes. However, shorter *MLO-*like sequences have been described in many plant species including tomato^31^, cucumber^32^, soybean^15^, strawberry^17^ and apple^17^. Thus, these sequences might represent a new family of membrane-proteins not considered before.

The *MLO* genes were widely distributed over the legume genomes. They were found on almost all chromosomes of any given species (Supplementary Table S1). In addition, most legume *MLO* orthologues were located, within conserved syntenic blocks, in related chromosomes in the different legumes (Supplementary Table S1). The *MLO* distribution supported the high level of micro- and macro-synteny that exist between legume genomes^38^,^39^. It also further illustrated the assumption that most legume genes are likely located in syntenic regions^40^ as previously demonstrated for most phenylpropanoid genes of soybean and common bean^41^. This distribution also suggested that they mainly arose from segmental duplication as it was already assumed for rice and several *Rosaceae* species^17^,^42^. Tandem duplication may have also played a minor role in *MLO* evolution since we detected evidence of a few tandem duplication events such as the gene pairs *PvMLO5/PvMLO6* and *VrMLO5/VrMLO6* in *P. vulgaris* and *V. radiata*, respectively (Supplementary Table S1).

The phylogenetic analysis classified the legume *MLOs* in seven clades (Figs 2 and 3), which is in accordance with previous studies^14^,^17^,^37^. The largest clades were clades I, II III and V that contained two to six *MLO* genes, in each legume species (Fig. 2 and Supplementary Fig. S2). In our analysis, the clade I was further divided in two well-supported sub-clades. These sub-clades can also be distinguished by the examination of their sequences (Fig. 2 and Supplementary Figs S2 and S3). One *MLO* per legume species was found in clade IV that was originally thought to be restricted to monocots^14^. Clade VI, characterized by the presence of *AtMLO3,* contained only a small number of legume sequences (Figs 2 and 3). This supports its recent addition to the *MLO* family^14^. The legume sequences in this clade were only from the Phaseoloid legumes including common bean, mung bean, pigeon pea and soybean (Fig. 2 and Supplementary Fig. S2). *AtMLO3* orthologues have been found in all eudicot species studied so far^17^,^31^,^32^,^37^. Lack of *AtMLO3* orthologues in the other legume species is thus surprising. This could be explained by either loss of these orthologues in lupin, barrel medic, chickpea and peanut or by their specific incorporation in the genome of the Phaseoloid species. Lupin and peanut belongs to the early-diverging clades, Genistoid and Dalbergioid, respectively^43^. Their separation from the other Papillionidoids clades has been estimated some 55–56 million years ago^44^. The evolution of the tropical (Phaseoloids) and temperate legumes (Galegoids) is more recent^43^,^44^. It has been estimated to have taken place approximately 52.8 and 50 million years ago, respectively^44^. According to this, it appears more likely that the Phaseoloid species (common bean, pigeon pea, mung bean and soybean) have incorporated this *MLO* clade during speciation. The phylogenetic study also revealed a seventh clade that was mainly represented by legume *MLO*. This is in accordance with recent studies that also identified a seventh clade in cucumber^32^ and tomato^31^. Another recent study on *Rosaceae MLO* identified two new clades apparently restricted to *Rosaceae* species (clades VII and VIII)^17^. However, *MLO* sequences from soybean, cucumber or tomato were not included in their analysis^17^. Thus, a more global analysis of *MLO* sequences, over plant kingdom, would be necessary to determine whether evolution of *MLO* sequences led to the apparition of genera-specific clades.

Previous studies identified several conserved motifs^15^,^16^,^31^,^32^,^35^ that we also detected in the legume MLO protein sequences (Table 3 and Supplementary Fig. S1). One of these common motifs, located at the C-terminal region, was previously shown to bind to the calcium-sensing protein calmodulin^35^. Here, we confirmed that the calmodulin binding site was conserved in all MLO clades (Supplementary Figs S3 to S9), since it was found within the common conserved motif 14 in all legume sequences (Fig. 3). In addition to these common motifs, our study identified 34 clade-specific motifs and several clade-specific amino acid residues. These motifs located in the extracellular loops 1 and 3, the intracellular loop 2 and the C terminal region (Table 4, Fig. 3 and Supplementary Table S4). For instance, six clade V-specific motifs were detected (Table 4, Fig. 3 and Supplementary Fig. S7). Two of them, motifs 25 and 27, contained the previously identified consensus clade V sequences^16^,^35^. This confirmed the efficacy of the method used. Interestingly, the conserved tetrad [E/D]FSF^35^ was also detected in clade IV and VI MLO sequences (Supplementary Figs S6, S7 and S8). The presence of this motif in clade IV sequences may have been expected since it contains the powdery mildew susceptibility genes of monocots. By contrast, its presence in the more divergent clade VI is surprising. It might indicate a common mechanism of action of these three clades. The identification of clade-specific motifs is very useful to isolate MLO orthologues in plant species not yet sequenced.

Beyond finding interesting new features about the *MLO* gene family, our study also showed diverging features between the tropical legumes (Phaseoloids) and the other legume species. One of the most striking differences was the total number of *MLOs* found in each type of legumes. Legumes from the Genistoid, Dalbergioid and Galegoid clades were characterized by 13 to 15 genes while tropical legumes contained from 18 to 31 genes (Table 1 and Supplementary Table S1). Almost all additional genes from tropical legumes clustered in clade V (Fig. 2 and Table 1). Given the importance of this clade in disease susceptibility, the specific multiplication of clade V *MLOs* in tropical legumes may reflect a greater pathogenic variability and pressure in tropical regions. Another phylogenetic difference was the lack of clade VI *MLO* genes in legumes from the Genistoid, Dalbergioid and Galegoid clades. The significance of the absence of this particular clade is not known. Clades V and VI correspond to the most recent diversification of the *MLO* family^14^. At this respect, our data suggested that the *MLO* family evolved differently depending on the legume clade considered. The tropical legume diverged after the separation from lupin and peanut ancestors but before that of the temperate legumes^44^. They are the only ones to have incorporated the clade VI *MLO* in its genome and to have followed a significant expansion of this family (Fig. 2 and Supplementary Fig. 2). Our data also indicate that the Genistoid, Dalbergoid and Galegoids, have evolved in parallel. These assumptions were also supported by the detection of two specific motifs (motifs 33 and 34) only found in clade VII *MLOs* of tropical legumes.

Altogether, our study characterized 118 *MLO* sequences from eight different legume species with different habitats and agronomic characteristics. This comparative analysis revealed interesting new phylogenetic features that may be the base to further determine the function of this gene family. We also detected several differences between tropical and the other legume species that might reflect different evolutionary pressures. In addition, we identified from three to seven genes in clades IV and V that contains the genes associated with powdery mildew susceptibility. These new sequences are very valuable to identify new gene variants to confer powdery mildew resistance in these species and to identify new susceptibility genes in additional legume species.

## Methods

### Identification, annotation and validation of legume *MLOs*

*M. truncatula MLO* sequences were identified by mining the JCVI *M. truncatula* genomic project v4.0 database through BLAST searches with the 15 *Arabidopsis thaliana MLO* sequences as templates. All potential *MLO* sequences from the other legume species (Table 2) were retrieved by BLAST^45^ using the M. *truncatula MLO* CDS and protein sequences. In all cases, the lowest limit of significance (e-value) for any potential hits was set at 1e^−20^. All potential *MLO* sequences were systematically validated by reciprocal BLAST on the *M. truncatula* JCVI *Mt4.0* (http://jcvi.org/medicago/index.php) and *A. thaliana TAIRv10* databases (http://www.arabidopsis.org).

Upon validation, the genomic sequence of each potential *MLO* was examined to reconstruct the full length CDS and correct potential annotation errors. Each genomic sequence was then aligned to its corresponding transcripts by BLAST against its respective transcriptomic (TSA) and EST databases that are stored at the NCBI website (http://blast.ncbi.nlm.nih.gov/Blast.cgi). In parallel, validated *MLO* sequences, from unannotated legume genomes (*Lupinus angustifolius* and *Vigna radiata*), were analyzed with Fgenesh^46^ using the “Medicago legume gene” model (Supplementary Table S2). Manual correction of the annotation was also performed, if necessary, to improve sequence quality. SeqBuilder v12.0 (DNASTAR, Madison, WI) was used to draw and correct the resulting exon-intron organization of each sequence. Supplementary Figs S10 and S11 show the CDS and deduced protein sequences of the legume *MLO*s, respectively.

### Conservation and phylogenetic analyses

Global pair-wise analysis was performed on the deduced protein sequences to determine their level of conservation to their closest homologue in *M. truncatula* and *A. thaliana* with Geneious R8 (http://www.geneious.com)^47^. Multiple protein sequence alignments were performed with ClustalW^34^. The alignments were manually corrected before phylogenetic reconstruction.

To assign each potential *MLO* to its clade, all identified MLO protein sequences were aligned with the MLO sequences from soybean and several non-legume species (Table 1). This alignment was used to calculate a *p-*distance matrix after pair-wise deletion of gaps using the MEGA6 software^48^. Then, a phylogenetic tree was reconstructed based on the *p*-distance matrix with the NJ algorithm. This analysis was performed with 1,000 bootstrap replicates with the MEGA6 software^48^. The phylogenetic relationship of legume MLO was also established using the MP and ML methods implemented in the MEGA 6 software. The search for the most parsimonious tree (MP method) was performed on 10 initial trees with the subtree-pruning-re-grafting method and 1,000 bootstrap replicates. Prior to ML analysis, all gaps and divergent regions were removed from the protein alignment with Gblocks version 0.91b^49^. The resulting alignment was then used to estimate the optimum substitution model with ProtTest 3.4^50^. Subsequently, the ML tree was obtained on 1,000 bootstrap replicates using the JTT substitution model with gamma distribution of 8 categories and α = 1.05 following the ProtTest estimation.

### Protein characterization and motif prediction

The deduced amino acid sequences of the potential *MLO* genes were subjected to several prediction programs to determine their sub-cellular localizations^28^,^51^, protein topologies^27^,^28^,^29^,^52^ and to identify functional domains^29^. The prediction servers used in this study are listed in Supplemental Table S2. Except otherwise stated, the prediction server were run with default settings. The result of these predictions was then used to draw the protein organization of each potential MLO protein on the IBS server^53^ (Supplementary Table S2).

Conserved motifs were determined with the MEME algorithm^30^ (Supplementary Table S2). The MEME parameters were set to search for a maximum of 15 motifs with a motif width comprised between five and 50 residues. Presence or absence of the conserved motifs in each MLO sequence was then determined using FIMO and MAST algorithms also available from the MEME suite web server^30^ (Supplementary Table S2).

## Additional Information

**How to cite this article**: Rispail, N. and Rubiales, D. Genome-wide identification and comparison of legume *MLO* gene family. *Sci. Rep.* **6**, 32673; doi: 10.1038/srep32673 (2016).

## Supplementary Material

Supplementary Information

## Figures and Tables

**Table 1:** *MLO* family members of legume and non-legume species and their phylogenetic classification^a^.

Plant species	Common name	Total	Clade							Reference
			1	2	3	4	5	6	7	
**Non-legume species**										
*Arabidopsis thaliana*	Thale cress	15	3	3	5	0	3	1	0	Chen *et al*.^36^
*Vitis vinifera*	Grapevine	14	3	3	2	1	4	2	2	Feechan *et al*.^37^
*Cucumis sativus*	Cucumber	13	4	2	3	0	3	1	0	Zhou *et al*.^32^
*Solanum lycopersicum*	Tomato	15	3	3	3	0	4	1	1	Chen *et al*.^31^
*Hordeum vulgare*	Barley	11	2	7	1	1	0	0	0	Kusch *et al*.^16^
*Capsicum annuum*	Pepper	2					2			Kim and Huang^19^, Panstruga^35^,
**Legume species**										
*Medicago truncatula* (Galegoid clade)	Barrel medic	14	3	4	3	1	2	0	1	This study
*Cicer arietinum (*Galegoid clade)	Chickpea	13	3	3	3	1	2	0	1	This study
*Pisum sativum* (Galegoid clade)	Pea	4	1	1			1		1	This study
*Lupinus angustifolius* (Genistoid clade)	Narrow-leaf lupin	15	3	3	6	0	3	0	0	This study
*Arachis* spp. (Dalbergioid clade)	Peanut	14	4	4	2	1	2	0	1	This study
*Glycine max* (Phaseoloid clade)	Soybean	31^b^	5	5	6	2	9	2	2	Deshmukh *et al*.^15^
*Cajanus cajan* (Phaseoloid clade)	Pigeonpea	20	4	3	3	1	6	1	2	This study
*Phaseolus vulgaris* (Phaseoloid clade)	Common bean	19	4	3	3	1	6	1	1	This study
*Vigna radiata* (Phaseoloid clade)	Mungbean	18	3	3	3	1	6	1	1	This study

^a^*MLO* classification is based on Neighbor-Joining phylogenetic analysis and literature.

^b^Actualized *G. max MLO* sequences after removing partial sequences and those classified by Genbank as obsolete sequences.

**Table 2:** Legume genomic databases used in this study.

Species	Common name	Legume clade	Depository (Bioproject)	Version	Web address	Reference
*Medicago truncatula*	Barrel medic	Galegoid	JCVI	v.4	http://jcvi.org/medicago/index.php	Young *et al*.^54^
*Cicer arietinum*	Chickpea	Galegoid	NCBI (PRJNA190909)	v.1	ftp://ftp.ncbi.nlm.nih.gov/genomes/Cicer_arietinum/	Varshney *et al*.^55^
			GigaDB		http://gigadb.org/dataset/100076	
*Lupinus angustifolius*	Narrow-leaf lupin	Genistoid	NCBI (PRJNA179231)	Draft	http://www.ncbi.nlm.nih.gov/Traces/wgs/?val=AOCW01#contigs	Yang *et al*.^56^
*Arachis* spp.	Peanut	Dalbergioid	PeanutBase	v.1	http://www.peanutbase.org	Bertioli *et al*.^57^
*Cajanus cajan*	Pigeonpea	Phaseoloid	GigaGB	v.5	http://gigadb.org/dataset/100028	Varshney *et al*.^58^
*Phaseolus vulgaris*	Common bean	Phaseoloid	Phytozome	v.1	http://phytozome.jgi.doe.gov/pz/portal.html#!info?alias=Org_Pvulgaris	Schmut *et al*.^59^
*Vigna radiata*	Mungbean	Phaseoloid	NCBI (PRJNA243847)	v.1	http://www.ncbi.nlm.nih.gov/Traces/wgs/?val=JJMO01#contigs	Kang *et al*.^60^

**Table 3:** Conserved motifs common to all sequences as detected by MEME software.

Motif	Sequence	e-value	N° sequence
1	RSL[EDA]ETPTWAVAVVC[TF]V[FIL][VLI][AL][IV]S	6.4e^−1500^	208/212
2	[LAI][ILV]E[RHK][SI]H[KR][LI]GKWLKKK[HN]KKAL[LYF]	1.6e^−997^	205/212
3	E[AS]LEK[IV]K[EA]ELMLLGFISLLLT[VF]	1.6e^−1654^	209/212
4	[SA][KR]IC[IV][PS][ES][KS]VA[DNS][ST][MW][LHF]PC	1.4e^−821^	193/212
5	P[LF][VLI]S[VY]EG[LI][HE]QLH[IR]FIF[VF]LA[VI][FT]H[VI]L[YF][SC][VIA][LI]T[MVL][LA]L[GA]R[AL]K[IM]R[RS]	3.0e^−2726^	211/212
6	WK[ARK]WE[EAD]ET[KS][TS][LH]EY[QE]F[ASY][NH]DP[ES]RFR[FL][AT][RH][EDQ]T[ST]F[GV]RRHLS	2.5e^−1389^	127/212
7	[CS]FFRQF[YF][GR]SVT[KR][VA]DYL[TA]LR[HL]GF	1.2e^−1793^	212/212
8	KF[DN]F[HQ]KY[IM]KRS[LM]E[DE]DFKV[VI]VG[IV]S[PW]PLW[FA][FS][VA]V	2.9e^−2348^	210/212
9	N[IVT][HN]GW[HYN][TS]YFW[LI][SPA]FIP[LV][IV][IL][ILV]L[LA]VGTKL[QE][HV][IV]I[TA]	1.9e^−2051^	211/212
10	M[AG]L[ERD]I[QAT][ED][RK][HG][AE]V[VI][KQ]GI[PL][LV]V[QE]P[SG]D	1.6e^−956^	156/212
11	FWF[NG][RK]P[RQ]L[VL]L[FH]LIH[FL][IV]LFQNAF	1.7e^−1699^	212/212
12	[AT][FY]F[FL]W[TIS]W[YW][EQT][FY]GFDSC[FI]	3.8e^−946^	207/212
13	R[LV][AI][LM]GV[FA][VI]Q[VF]LCSY[VSI]TLPLYA[LI]VTQMG[ST][TR]MK	7.8e^−2592^	212/212
14	K[ATS]IF[DN]E[QR][VT][ARS]KALK[KNG]WHK[TA][AV]KKKxKHKKxGSS	2.2e^−1128^	189/212

**Table 4:** Conservation of previously identified invariable amino acid residues^33^ in legume MLO sequences at species and clade level.

Barley Residue	Legume species								MLO clade							
	Mt	Ca	La	Ar	Cc	Pv	Vr	Gm	Ia	Ib	II	III	IV	V	VI	VII
E_35_	E/N	E/H	E/Q	E/H/D	E/Q/D	E/Q/D	E/Q	E/Q	Q/H/N	E	E	E	E	E/D	E	E
M_65_	M	M	M	M/I	M	M	M	M	M	M	M	M	M	M/I	M	M
G_68_	G	G	G	G	G	G	G	G	G	G	G	G	G	G	G	G
S_71_	S	S	S	S	S	S	S	S	S	S	S	S	S	S	S	S
L_74_	L/M	L/M	L/M	L/M	L/M	L/M	L/M	L/M	M	L	L/M	L	L/I	L	L	L
C_86_	C	C	C	C	C	C	C	C	C	C	C	C	C	C	C	C
C_98_	C	C	C	C	C	C	C	C	C	C	C	C	C	C	C	C
C_114_	C	C	C	C	C	C	C	C	C	C	C	C	C	C	C	C
F_135_	F/S	F/S	F	F/S	F/S	F/S	F/S	F/S	F	F	F	F	F	F	F	S
W_158_	W	W	W	W	W	W	W	W	W	W	W	W	W	W	W	W
E_163_	E	E	E/Q	E	E/D	E/D	E/D	E/D	E	E	E	E/Q	E	E	D	E
F_207_	F	F	F	F	F/L	F	F	F	F	F	F	F	F	F	F	F/L
Q_210_	Q	Q	Q	Q	Q	Q	Q	Q	Q	Q	Q	Q	Q	Q	Q	Q
Y_220_	Y	Y	Y/F	Y	Y/F	Y	Y	Y	Y	Y	Y	Y/F	Y	Y	Y	Y
R_224_	R	R	R	R	R	R	R	R	R	R	R	R	R	R	R	R
F_227_	F	F	F	F	F	F	F	F	F	F	F	F	F	F	F	F
F_240_	F	F	F	F	F	F	F	F	F	F	F	F	F	F	F	F
Y_243_	Y/F	Y/F	Y	Y/F	Y/F	Y/F	Y/F	Y/F	Y	Y	Y	Y	Y	Y	Y	F
W_263_	W	W	W	W	W	W	W	W	W	W	W	W	W	W	W	W
P_287_	P	P	P	P	P	P	P	P	P	P	P	P	P	P	P	P
F_329_	F	F	F	F	F	F	F	F	F	F	F	F	F	F	F	F
W_330_	W	W	W	W	W	W	W	W	W	W	W	W	W	W	W	W
P_334_	P	P	P	P	P	P	P	P	P	P	P	P	P	P	P	P
F_346_	F/I	F/I	F	F/L	F/I	F/I	F/I	F/I	F	F	F	F	F	F	F	I/L
N_348_	N	N	N/I	N/T	N	N	N	N	N	N	N/I	N	N	N	N	N/T
F_350_	F	F	F/I	F	F	F	F	F	F	F	F/I	F	F	F	F	F
C_367_	C	C	C	C	C	C	C	C	C	C	C	C	C	C	C	C
T_393_	T	T	T	T	T	T	T	T	T	T	T	T	T	T	T	T
P_395_	P	P	P	P	P	P	P	P	P	P	P	P	P	P	P	P
W_423_	W/L	W	W	W	W	W	W	W	W	W	W	W	W	W	W	W

**Table 5:** Clade-specific motifs in MLO sequences as detected by MEME software.

Clade	Motif	Sequence	e-value	N° Sequence
I	1	[DN][SG][LN]S[QE][IS][TK][RKS][ES][LIK][TR][ML]RR[QL][ST]TF[VI][FK][HS]H[TA]S[HN]P[WL]S[RHK][NH][KSP]	1.2e^−405^	42/212
I	2	I[TM][NE]HNL[PS]L[KTS]	1.1e^−100^	29/212
I	3	[TK]LA[LV]E[NI]A[GE][IQR][TC][GP][FP][FM][SKP][EPR][AHT][KQ][LFV][RKN][PL]RDELFWF[KNG]KP[ERD]	8.5e^−565^	40/212
I	4	TRS[EQ]ID[EK][EQ][MI]E[ED]NGSE[EG]RKLL[MT]A	4.1e^−151^	18/212
I	5	[YA][PY][HR][LV][IF][RG]RML[ND]G[IM]NR[SN][ST]	3.9e^−082^	13/212
I	6	TIHTDTSTVLSIEEDDQLID[DAT]PE	1.2e^−161^	16/212
I	7	[AT]VT[SA]TPSPIANETSSRA[VA]TPLLRPSASISS[SV][HQV][PCS][SF]S	1.0e^−190^	14/212
II	8	G[AL][RK]IR[QE]WKHWEDSIAK[QE]NYETx	2.1e^−334^	50/212
II	9	[RP]VL[KE]P[KT]VT[HN]V[HQ]QH[ADE]FI	8.3e^−146^	26/212
II	10	GIQLGS[VI]F[RKQ][KR][AR][SA][AS][PA][EP][DE]	3.7e^−126^	22/212
II	11	[AKN][KR][KR][KR][GL]L[KRS][AG]D[SGN][NQ][SHP][SGQ][HS][GC]S	8.1e^−111^	28/212
III	12	[EG][EG]EH[RH]R[KR]LLSYERR[YF]L[AS][AG][DG][GTA][TG][SG]	8.1e^−314^	33/212
III	13	[DGS]ST[VI]HSSGPTLHR[FY]KTTGHSTR	7.1e^−258^	26/212
III	14	Y[DE]D[QD]D[DE]Y[HEQ]SDIE	4.5e^−079^	18/212
III	15	[PQ]T[AT][SNT][LI][IV][VT]RVD[HN][GD][ED]Q[QE][AQ]EE[EN]E[HE]H	3.0e^−144^	26/212
IV	16	[FD][DE][DE]N[MLV][EV]WRRVLA	7.3e^−076^	11/212
IV	17	A[AS][SG]G[GD]DYCS[QN]KGKV[PS]LISQSGV	9.5e^−091^	9/212
IV	18	SGE[TA]TPSQGTSP[LI]HLL[HQ]K[YF]KPS	1.2e^−099^	12/212
IV	19	[HQ]TDTDSVLYSPRSYQSD	1.1e^−080^	8/212
IV	20	TD[LF]S[DE]TEGS[ST]HQLN[EL]I[TQ][QI][TM][HS]Q[PA]	3.4e^−066^	8/212
IV	21	P[RN]N[GQ][EL]THNI[DE]FSF[VD][KS]P	2.5e^−044^	8/212
V	22	LA[TAG][GK]GYDKC	8.5e^−169^	28/212
V	23	FW[TS][QK][SN][PT][IV][LS][LV]WIV	2.7e^−203^	50/212
V	24	FHSTT[EA]D[VI]VIR[IL]	2.4e^−198^	51/212
V	25	STTPFSSRP[ST]TPTHGMSP[VA]HLL	4.4e^−360^	30/212
V	26	[APR][GRS][RHE]SDS[AFP][QP]TSPR[TAR]SNY[ED]NEQWD	3.0e^−275^	50/212
V	27	P[ITV][SR][SHT][QE][HIL]EI[NR][IV][SA][SL][SK][ED]FSF[EDG][KR][RG][HP][THI]	9.7e^−142^	24/212
VI	28	[SC]KALAK[IM]L[KR]QWH[VL]EVRERR	1.4e^−131^	5/212
VI	29	[QN][RELQ][KE][LQ][VL]KSFSF[RS][HR]	1.3e^−009^	5/212
VI	30	MSSEWSQGNKSAP[ED]FSSTL[CR]E[SN][IANT]RSSDEGEIVEELEH[MPRS][VDEM]KTKA[SCNT]SSSDPP	2.3e^−103^	5/212
VII	31	N[PG]KIITRG[TI]YDGEISFGS[SY][WV][KG][NS]	9.5e^−100^	10/212
VII	32	SSRGI[GR]EI[GV]SI[TAI]EE[DE]D	2.7e^−046^	9/212
VII	33	AT[RH]TSTS[EGQ][LF]D[VI]A[PH]ATN[EQ]S[TAEN][IV]E[VF]	8.2e^−038^	5/212
VII	34	NNSTSSKHSDSLHSK[EG][GC]DNS[AV]RG[ST][VM]DSVH[TN]PDNV[VA][LV]T[SN][NP]P[SF][PH]	1.8e^−071^	5/212
