# Supplementary material for: Genome-wide identification and comparison of legume MLO gene family
Source: Sci Rep. 2016 Sep 6;6:32673. doi: 10.1038/srep32673 (PMC5011691; doi:10.1038/srep32673)
Supplement: Supplementary Information [file srep32673-s1.pdf]

Supplementary Material

Genome-wide identification and comparison of legume MLO gene family

Nicolas Rispail*, Diego Rubiales

Institute for Sustainable Agriculture-CSIC, Córdoba, Spain.

*** Correspondence:** Dr. Nicolas Rispail, Institute for Sustainable Agriculture-CSIC, Avda. Menéndez Pidal s/n, 14004 Córdoba, Spain. Email: nrispail@ias.csic.es

Supplementary Table S1. Characteristics of full length legume *MLO* genes.

Gene name	Contig name	Transcript	Exon	Chr.	Dir.	Lenght (kb/aa)			Protein characteristics	
						Genomic	CDS	Protein	pI	kDa
Medicago truncatula										
MtMLO1	Medtr6g033330		15	Chr6	-	5.91	1.73	574	9.18	66.01
MtMLO2	Medtr5g022400		15	Chr5	+	3.49	1.64	545	8.19	63.11
MtMLO3	Medtr2g093750		14	Chr2	+	3.24	1.78	593	9.19	67.83
MtMLO4	Medtr2g028470		14	Chr2	-	4.27	1.44	479	7.89	55.75
MtMLO5	Medtr3g115960		15	Chr3	+	3.97	1.61	537	7.41	60.75
MtMLO6	Medtr5g024700		15	Chr5	+	5.09	1.70	567	9.69	64.92
MtMLO7	Medtr2g006280		15	Chr2	+	4.44	1.66	551	8.15	63.60
MtMLO8	Medtr3g115940		14	Chr3	+	3.69	1.66	551	9.39	63.49
MtMLO9	Medtr3g118190		12	Chr3	+	4.12	1.55	517	9.35	58.43
MtMLO10	Medtr8g073130		15	Chr8	-	6.11	1.77	588	9.04	67.14
MtMLO11	Medtr4g079140		14	Chr4	-	5.63	1.71	568	7.26	65.31
MtMLO12	Medtr2g030020		5	Chr2		1.19	0.48	159		
MtMLO13	Medtr8g010500		14	Chr8	-	3.54	1.57	522	8.35	59.05
MtMLO14	Medtr8g010530		13	Chr8	-	3.11	1.56	520	9.12	59.41
MtMLO15	Medtr2g079500		15	Chr2	-	5.17	1.65	549	9.63	62.82
MtMLO16	Medtr5g009630		7	Chr5	-	5.60	0.654	217		
		**mean**	**14**			**4.41**	**1.65**	**547**	**8.70**	**62.69**
Cicer arietinum										
CaMLO1	Ca_04814 / LOC101504273	XM_004500787	14	Ca5	-	2.98	1.66	553	9.63	63.72
CaMLO2	Ca_23649 / LOC10500098	XM_004514937	15		+	7.96	1.68	559	9.57	64.53
CaMLO3	Ca_04697 / LOC101490933	XM_004500656, XM_004500657	13	Ca 5	-	3.94	1.60	533	9.44	60.54

Gene name	Contig name	Transcript	Exon	Chr.	Dir.	Lenght (kb/aa)			Protein characteristics	
						Genomic	CDS	Protein	pI	kDa
CaMLO4	Ca_01959 / LOC101505951		15	Ca 8	+	4.63	1.56	518	9.78	60.03
CaMLO5	Ca_04811 / LOC101503406	XM_004500784, XM_004500785	15	Ca5	-	3.40	1.75	481	6.78	65.76
CaMLO6	Ca_02517 / LOC101492588	XM_004487077	15	Ca1	-	3.40	1.77	590	9.28	67.44
CaMLO7	Ca_11758 / LOC101501959	XM_004510141	13	Ca7	-	4.33	1.54	513	8.26	58.71
CaMLO8	Ca_00046 / LOC101508058	XM_004485414, XM_004485415	15	Ca1	+	3.68	1.67	555	8.75	63.95
CaMLO9	Ca_11277 / LOC101495573	XM_004505476	14	Ca6	+	3.89	1.75	560	7.90	64.65
CaMLO10	Ca_10710 / LOC101494654	XM_004512376	15	Ca8	+	4.68	1.67	555	9.20	63.51
CaMLO11	Ca_06880 / LOC101511064	XM_004487635	15	Ca1	+	7.65	1.66	551	9.67	63.06
CaMLO12	Ca_09883 / LOC101496809	XM_004509404	15	Ca7	+	6.35	1.68	586	9.30	66.87
CaMLO13	Ca_07953 / LOC101510624	XM_004486357	14	Ca1	-	5.56	1.40	465	7.08	53.86
	Ca_15834	gnomon17445181	7	Ca7	-			178		
		**mean**	**14**			**4.80**	**1.65**	**540**	**8.82**	**62.82**
Pisum sativum										
PsMLO1	FJ463618	GAMJ01003109					1.72	574	9.38	65.94
PsMLO2		GCMF01001473					1.56	518	9.30	58.58
PsMLO3		GAMJ01004855					1.76	581	7.87	66.81
PsMLO4		GAMJ01021849, GAMJ01018602,GAMJ01042925					1.65	550	9.65	62.73
Lupinus angustifolius										
LaMLO1	AOCW01137726_AOCW01137727	GBRP01032299, GBRP01057990, GBRP01055692	15		+	6.51	1.72	571	8.95	65.84
LaMLO2	AOCW01065981_AOCW01147792	GBRP0101286	15		-	5.98	1.72	572	8.66	65.82
LaMLO3	AOCW01159416		15		+	4.43	1.43	474	9.27	55.48
LaMLO4	AOCW01114694_AOCW01136624	GBRP01058592, GBRP01073743	15		+	8.19	1.79	597	10.22	68.80
LaMLO5	AOCW01127929	GBRP01087504	13		-	4.38	1.54	513	7.51	59.54
LaMLO6	AOCW01133085		15		-	3.91	1.55	514	9.72	58.16
LaMLO7	AOCW01138453	GBRP01046806, GBRP01061917	15		-	4.28	1.62	540	8.54	61.01
LaMLO8	AOCW01146534	GBRP01037926	15		-	6.26	1.78	591	9.34	66.85

Gene name	Contig name	Transcript	Exon	Chr.	Dir.	Lenght (kb/aa)			Protein characteristics	
						Genomic	CDS	Protein	pI	kDa
LaMLO9	AOCW01154130	GBRP01073792, GBRP01080451	15		-	5.49	1.67	554	9.99	63.21
LaMLO10	AOCW01039454_AOCW01039455		15		-	4.87	1.74	578	9.19	66.07
LaMLO11	AOCW01135152	GBRP01069086, GBRP01059884	15		-	5.95	1.75	581	8.88	66.05
LaMLO12	AOCW01152069	GBRP01084057, GBRP01089663	15		-	4.31	1.61	537	9.08	61.96
LaMLO13	AOCW01152789	GBRP01018422	13		-	3.80	1.49	497	9.37	56.89
LaMLO14	AOCW01138218	GBRP01077588	13		-	6.72	1.53	510	8.23	58.02
LaMLO15	AOCW01116641		15		+	5.83	1.66	552	8.85	62.69
		**mean**	**15**			**5.39**	**1.64**	**545**	**9.05**	**62.43**
***Arachis* spp.**										
ArMLO1	ARADU.27DYI / ARAIP.PW5DF	GBIP01027541, GBIR01038206	15	Chr1/Chr10	-	5.53	1.71	569	9.00	63.96
ArMLO2	ARADU.5Y217/ARAIP.Y3LT4	GBJG01024432	13	Chr2/Chr9	-/ +	7.26	1.52	507	9.00	58.15
ArMLO3	ARADU.65SUF/ARAIP.0US16_7B80	GBIY01009746, GBJL01025099	14	Chr3	-	7.34	1.79	594	6.45	67.88
ArMLO4	ARADU.N8G7F/ARAIP.N905Y	GBJI01054888, GAIG01036165, GBJI01028580, GBIX01019295	14	Chr4	-	3.47	1.59	504	7.36	58.79
ArMLO5	ARADU.U7W18/ARAIP.Z8ALS	GBJI01008120	15	Chr4	-	3,45	1.69	563	8.30	64.59
ArMLO6	ARADU.EU0BW/ARAIP.HRR0E		15	Chr5	-	3.20	1.60	533	8.59	61.72
ArMLO7	ARADU.0YR51/ARAIP.SD8AP	GBJI01054216, GBJI01099636	14	Chr8/Chr7	+/ -	3.81	1.69	561	9.75	64.36
ArMLO8	ARADU.QG7T6/ARAIP.L2HMX	LN657014	15	Chr8/Chr7	+/ -	3.38	1.60	531	9.67	59.96
ArMLO9	ARADU.NF3XX_3QT4K/ ARAIP.CFV17	GAER01011659	14	Chr8	+	4.74	1.61	536	9.63	61.38
ArMLO10	ARADU.WJ2TG/ARAIP.N0DTV	GBJE01045697	13	Chr8/Chr7	-	5.20	1.55	515	9.08	58.72
ArMLO11	ARADU.AK182/ARAIP.SR8Z2	GBJE01048686	15	Chr8	+	5.64	1.68	558	9.12	63.30
ArMLO12	ARADU.18BQR/ARAIP.WMV74		13	Chr9	-	4.09	1.51	501	8.25	57.75
ArMLO13	ARADU.SSV2N_48N80	GBJE01033068, GBIY01021795	15	Chr4	-	28.05	1.68	559	9.38	63.91
ArMLO14	ARAIP.MQE1N	GBIX01058238,GBJI01167672	17	Chr9	+	5.29	1.54	512	8.69	58.50
		**mean**	**14**			**6.46**	**1.63**	**539**	**8.73**	**61.64**
Cajanus cajan										
CcMLO1	C.cajan_46346	EZ668429, EZ666134	15		+	8.43	1.76	586	8.9	66.92
CcMLO2	C.cajan_07194	EZ630985.,EZ631564	15	CcLG02	-	4.69	1.76	586	9.1	67.34

Gene name	Contig name	Transcript	Exon	Chr.	Dir.	Lenght (kb/aa)			Protein characteristics	
						Genomic	CDS	Protein	pI	kDa
CcMLO3	C.cajan_32587		15		-	11.51	1.68	558	9.48	63.9
CcMLO4	C.cajan_12516	EZ676746	15	CcLG06	+	4.8	1.6	531	9.18	61.13
CcMLO5	C.cajan_34110		15		+	5.85	1.69	562	9.66	65.12
CcMLO6	C.cajan_34112	EZ634497	15		+	7.4	1.67	557	10.12	64.50
CcMLO7	C.cajan_21483	EZ636890	15	CcLG04	+	6.15	1.72	572	10.07	65.53
CcMLO8	C.cajan_16636	EZ668567,EZ666340, EZ676653	14	CcLG08	+	5.08	1.47	489	7.81	56.80
CcMLO9	C.cajan_01454	EZ623710, EZ653599, EZ647598, EZ680636, EZ680914	15	CcLG11	+	5.13	1.79	596	9.25	67.9
CcMLO10	C.cajan_18941		15	CcLG07	+	3.81	1.58	525	8.12	59.76
CcMLO11	C.cajan_18939		14	CcLG07	+	5.47	1.63	542	9.89	62.35
CcMLO12	C.cajan_36621	EZ682163, EZ633519	15		+	5.75	1.7	567	9.03	64.00
CcMLO13	C.cajan_17246		15	CcLG08	-	4.99	1.7	562	8.52	64.48
CcMLO14	C.cajan_23778	EZ632699, EZ680401	13		+	5.28	1.54	513	9.45	57.64
CcMLO15	C.cajan_47280	EZ658204, EZ65806, EZ675970	13		+	6.6	1.55	515	8.85	58.48
CcMLO16	C.cajan_13783	EZ671180	14	CcLG10	+	7.37	1.77	573	8.28	65.22
CcMLO17	C.cajan_37300	EZ666312, EZ641386, EZ645440	16		+	8.28	1.66	547	8.88	63.03
CcMLO18	C.cajan_37291		14		-	13.11	1.56	519	7.68	59.92
CcMLO19	C.cajan_09813	EZ665270	15	CcLG03	-	6.54	1.49	495	9.33	57.01
CcMLO20	C.cajan_24758		15		-	6.16	1.55	517	9.23	59.8
		**mean**	**15**			**6.62**	**1.63**	**546**	**9.04**	**62.54**
Phaseolus vulgaris										
PvMLO1	Phvul.001G175900	GAMK01034684,GAMK01071155, GAMK01015455	15	Chr1	-	10.97	1.5	498	8.3	57.32
PvMLO2	Phvul.002G107600	GAMK01035468	15	Chr2	-	4.46	1.61	534	9.55	61.5
PvMLO3	Phvul.002G117900	GAMK01111725,GAMK01012053	15	Chr2	-	7.16	1.82	604	9.58	68.77
PvMLO4	Phvul.003G271300	GAMK01039073	15	Chr3	-	6.04	1.73	577	9.32	65.7
PvMLO5	Phvul.004G071700	GAMK01040872, GAMK01074602	15	Chr4	-	6.31	1.72	573	8.76	65.19

Gene name	Contig name	Transcript	Exon	Chr.	Dir.	Lenght (kb/aa)			Protein characteristics	
						Genomic	CDS	Protein	pI	kDa
PvMLO6	Phvul.004G071800	GAMK01030163,GAMK01023790, GAMK01048741, GAMK01043348	15	Chr4	-	10.48	1.62	539	10.09	61.5
PvMLO7	Phvul.005G082500	GAMK01116174, GAMK01040070, GAMK01095386	15	Chr5	-	16.62	1.64	544	8.98	62.21
PvMLO8	Phvul.005G136700	GAMK01048395, GAMK01056244	15	Chr5	-	4.21	1.66	551	9.67	64.03
PvMLO9	Phvul.005G136800	GAMK01065961, GAMK01098978, GAMK01018577	15	Chr5	-	6.1	1.68	560	10.06	65
PvMLO10	Phvul.006G133800	GAMK01046431, GAMK01056998	14	Chr6	+	4.64	1.43	474	7.11	55.79
PvMLO11	Phvul.006G216900	GAMK01044295, GAMK01053693, GAMK01061530, GAMK01054071	15	Chr6	-	4.11	1.54	512	9.1	59.07
PvMLO12	Phvul.007G093600	GAMK01033253	15	Chr7	+	4.95	1.67	556	8.7	63.35
PvMLO13	Phvul.009G006800		14	Chr9	+	3.03	1.66	551	9.85	63.24
PvMLO14	Phvul.009G007000	GAMK01055948,GAMK01088610	15	Chr9	+	4.33	1.65	550	8.62	61.7
PvMLO15	Phvul.009G117900	GAMK01045539, GAMK01119734, GAMK01064264	13	Chr9	-	5.57	1.54	513	9.34	58.01
PvMLO16	Phvul.010G034400	GAMK01051424, GAMK01041048, GAMK01109343, GAMK01017151	13	Chr10	+	5.97	1.52	507	8.71	57.98
PvMLO17	Phvul.011G073800	GAMK01113404	15	Chr11	+	6.42	1.7	566	9.82	64.85
PvMLO18	Phvul.011G122800	GAMK01050130, GAMK01040786, GAMK01049351, GAMK01050286	14	Chr11	+	5.15	1.72	571	8.57	65.85
PvMLO19	Phvul.011G172300		15	Chr11	-	9.14	1.64	545	8.98	62.33
	Phvul.009G193200		6	Chr9	+	4.38	0.3	99	10.15	22.59
		**mean**	**15**			**6.61**	**1.63**	**543**	**9.11**	**62.28**
Vigna radiata										
VrMLO1	JJMO01000124	GBXO01007773	15	Chr1	+	6.97	1.73	577	9.15	65.93
VrMLO2	JJMO01000125	GBXO01030879	15	Chr2	+	4.7	1.71	568	9.74	65.08
VrMLO3	JJMO01000125	FF394677, FF398548	14	Chr2	+	6.3	1.81	603	8.52	69.19
VrMLO4	JJMO01000126	GBXO01029476	15	Chr3	-	4.78	1.48	493	8.75	56.44

Gene name	Contig name	Transcript	Exon	Chr.	Dir.	Lenght (kb/aa)			Protein characteristics	
						Genomic	CDS	Protein	pI	kDa
VrMLO5	JJMO01000120	GBXO01004030	15	Chr4	+	4.78	1.68	560	10.02	65.24
VrMLO6	JJMO01000120		15	Chr4	+	5.29	1.65	548	9.51	63.61
VrMLO7	JJMO01000121	GBX01064970	14	Chr5	+	3.11	1.67	554	9.74	63.6
VrMLO8	JJMO01000121		16	Chr5	+	3.42	1.57	522	9.41	58.77
VrMLO9	JJMO01000121	GBXO01028231, FF546578, FF546738	13	Chr5	+	5.35	1.55	515	8.57	63.75
VrMLO10	JJMO01000123		15	Chr7	+	5.29	1.72	573	9.2	65.04
VrMLO11	JJMO01000128	GBXO01035550,GBXO01044502	15	Chr8	-	4.79	1.67	554	8.25	63.63
VrMLO12	JJMO01000129	GBXO01048488, GBXO01048759	13	Chr9	-	5.53	1.55	514	9.13	58.32
VrMLO13	JJMO01000320	GBXO01033295, GBXO01073055. GBXO01013847	15	Chr10	-	3.97	1.68	559	8.82	63.98
VrMLO14	JJMO01000320	GBXO01075545	14	Chr10	+	4.57	1.41	469	7.62	54.5
VrMLO15	JJMO01000319	GBXO01055350, GBXO01035975	15	Chr11	+	3.96	1.71	569	9.03	64.74
VrMLO16	JJMO01000319	GBXO01055546, GBXO01069063	15	Chr11	+	4.18	1.59	528	9.49	60.93
VrMLO17	JJMO01000831	GBXO01053704, GBXO01009906	15		+	12.95	1.56	520	10.09	59.55
VrMLO18	JJMO01001841	GBXO01045250	15		-	5.96	1.64	546	9.12	62.52
		**mean**	**15**			**5.33**	**1.63**	**543**	**9.12**	**62.49**

Supplementary Table S2. Online prediction servers and tools used for the analysis of legume *MLO* gene family.

Resources	Characteristics and purpose	Web address	References
Fgenesh	ab initio gene prediction program	http://www.softberry.com/berry.phtml?topic=index&group=programs&subgroup=gfind	Solovyev et al.[46]
SignalP	prediction of signal peptide	http://www.cbs.dtu.dk/services/SignalP/	Petersen et al.[59]
TMHMM	prediction of transmembrane domains	http://www.cbs.dtu.dk/services/TMHMM/	Krogh et al.[27]
Psort	prediction of protein subcellular localisation	http://psort.hgc.jp/form.html	Nakai and Horton[28]
Plant-mPloc	prediction of protein subcellular localisation	http://www.csbio.sjtu.edu.cn/bioinf/plant-multi/	Chou and Shen[60]
Interproscan5	Protein domain identification	http://www.ebi.ac.uk/interpro/search/sequence-search	Jones et al.[29]
IBS	Illustrator of biological sequences	http://ibs.biocuckoo.org/online.php#	Liu et al.[51]
MEME Suite	Identification of and Scanning for conserved motif	http://meme-suite.org/index.html	Bailey et al.[30]

Supplementary Table S3. Protein characteristics and identity score of legume *MLO* with its closest homologues in *M. truncatula* and *A. thaliana.*[a] Subcellular localization as predicted by the web server Psort[28] and Plant-mPloc[60]. mb and PM stand for membrane and Plasma membrane, respectively.[b] Prediction of Signal peptide (SP) as indicated by SignalP[59]. [c] Number of transmembrane domains (TM) as predicted by TMHMM[27] (T), Psort[28](P) and Interproscan5[29] (I) webservers.

Gene name	Interproscan prediction		Subcellular localization[a]		SP[b]	TM domain[c]			Identity with *M. truncatula*					Identity with *A. thaliana*				
									M. truncatula gene name	Blastp hit		Pair-wise (%)		A. thaliana gene name	Blastp hit		Pair-wise (%)	
	Pfam Ref.	name	Psort	Plant-mPloc		T	P	I		Id.	E-value	Id.	Sim.		Id.	E-value	Id.	Sim.
Medicago truncatula																		
MtMLO1	PF03094	MLO-related	mb	Cell mb	No	8	7	7	Medtr6g033330					*AtMLO6*	63	0.0	62.4	75.8
MtMLO2	PF03094	MLO-related	PM	Cell mb	No	6	6	7	Medtr5g022400					*AtMLO4*	51	$1e^{-131}$	45.0	62.7
MtMLO3	PF03094	MLO-related	mb	Cell mb	No	7	7	7	Medtr2g093750					*AtMLO6*	56	0.0	56.1	70.4
MtMLO4	PF03094	MLO-related	mb	Cell mb	No	7	6	7	Medtr2g028470					*AtMLO13*	60	$1e^{-144}$	55.9	68.2
MtMLO5	PF03094	MLO-related	mb	Cell mb	No	7	6	7	Medtr3g115960					*AtMLO5*	57	$1e^{-175}$	57.3	71.2
MtMLO6	PF03094	MLO-related	PM	Cell mb	Yes	8	7	7	Medtr5g024700					*AtMLO8*	58	0.0	55.6	70.4
MtMLO7	PF03094	MLO-related	PM	Cell mb	No	6	6	7	Medtr2g006280					*AtMLO4*	68	0.0	64.9	78.4
MtMLO8	PF03094	MLO-related	mb	Cell mb	No	7	7	7	Medtr3g115940					*AtMLO12*	50	$1e^{-150}$	46.5	62.0
MtMLO9	PF03094	MLO-related	PM	Cell mb	No	6	7	7	Medtr3g118190					*AtMLO1*	65	0.0	66.8	75.0
MtMLO10	PF03094	MLO-related	PM	Cell mb	Yes	8	7	7	Medtr8g073130					*AtMLO8*	56	0.0	59.9	72.7
MtMLO11	PF03094	MLO-related	mb	Cell mb	No	7	5	7	Medtr4g079140					*AtMLO12*	42	$1e^{-128}$	38.8	59.2
MtMLO13	PF03094	MLO-related	mb	Cell mb	No	7	8	7	Medtr8g010500					*AtMLO1*	60	0.0	59.9	71.1
MtMLO14	PF03094	MLO-related	mb	Cell mb	No	7	7	7	Medtr8g010530					*AtMLO1*	63	0.0	63.8	75.0
MtMLO15	PF03094	MLO-related	mb	Cell mb	No	7	7	7	Medtr2g079500					*AtMLO11*	68	0.0	67.4	78.3
Cicer arietinum																		
CaMLO1	PF03094	MLO-related	mb	Cell mb	No	7	7	7	Medtr3g115940	91.9	0.0	91.9	95.7	*AtMLO12*	49	$1e^{-150}$	47.0	62.1
CaMLO2	PF03094	MLO-related	mb	Cell mb	No	7	7	7	Medtr6g033330	82.7	0.0	82.3	89.4	*AtMLO6*	62	0.0	62.0	75.9
CaMLO3	PF03094	MLO-related	PM	Cell mb	No	6	7	7	Medtr3g118190	83.7	0.0	81.8	87.1	*AtMLO1*	71	0.0	65.0	73.5
CaMLO4	PF03094	MLO-related	mb	Cell mb	No	7	7	7	Medtr5g022400	83.8	0.0	79.5	86.7	*AtMLO4*	50	$1e^{-30}$	44.8	61.0
CaMLO5	PF03094	MLO-related	mb	Nucleus	No	7	6	7	Medtr3g115960	81.0	0.0	81.1	84.7	*AtMLO5*	54	$1e^{-173}$	54.1	66.3
CaMLO6	PF03094	MLO-related	mb	Cell mb	No	6	6	7	Medtr2g093750	83.4	0.0	83.4	88.1	*AtMLO6*	62	0.0	56.7	71.0
CaMLO7	PF03094	MLO-related	mb	Cell mb	No	6	8	7	Medtr8g010530	82.1	0.0	81.2	89.4	*AtMLO1*	65	0.0	64.5	74.7

Gene name	Interproscan prediction		Subcellular localization[a]		SP[b]	TM domain[c]			Identity with *M. truncatula*					Identity with *A. thaliana*				
	Pfam Ref.	name	Psort	Plant-mPloc		T	P	I	M. truncatula gene name	Blastp hit		Pair-wise (%)		A. thaliana gene name	Blastp hit		Pair-wise (%)	
										Id.	E-value	Id.	Sim.		Id.	E-value	Id.	Sim.
CaMLO8	PF03094	MLO-related	PM	cell mb	No	6	6	7	Medtr2g006280	88.7	0.0	88.3	92.3	*AtMLO4*	71	0.0	64.1	76.9
CaMLO9	PF03094	MLO-related	PM	Cell mb	No	8	6	7	Medtr4g079140	76.8	0.0	75.3	82.4	*AtMLO6*	44	1e^{-123}	38.6	58.9
CaMLO10	PF03094	MLO-related	PM	Cell mb	No	8	6	7	Medtr5g024700	83.7	0.0	80.8	87.0	*AtMLO8*	59	0.0	54.6	69.5
CaMLO11	PF03094	MLO-related	mb	Cell mb	No	7	7	7	Medtr2g079500	90.7	0.0	90.6	93.8	*AtMLO11*	69	0.0	68.5	78.9
CaMLO12	PF03094	MLO-related	PM	Cell mb	Yes	8	7	7	Medtr8g073130	80.8	0.0	76.9	85.3	*AtMLO8*	61	0.0	60.8	74.5
CaMLO13	PF03094	MLO-related	mb	Cell mb	No	7	6	7	Medtr2g028470	82.4	0.0	74.4	81.2	*AtMLO13*	57	1e^{-146}	56.9	69.4
Pisum sativum																		
PsMLO1	PF03094	MLO-related	mb	Cell mb	No	8	7	7	Medtr6g03330	83.3	0.0	83.3	91.0	*AtMLO12*	64	0.0	61.2	74.2
PsMLO2	PF03094	MLO-related	PM	Cell mb	No	7	7	7	Medtr3g118190	88.3	0.0	88.3	92.5	*AtMLO1*	70	0.0	66.4	74.5
PsMLO3	PF03094	MLO-related	mb	Cell mb	No	8	6	7	Medtr4g079140	82.5	0.0	78.9	84.1	*AtMLO6*	44	1e^{-124}	38.6	56.4
PsMLO4	PF03094	MLO-related	mb	Cell mb	No	7	7	7	Medtr2g079500	91.1	0.0	91.1	95.8	*AtMLO11*	69	0.0	68.6	79.6
Lupinus angustifolius																		
LaMLO1	PF03094	MLO-related	mb	Cell mb	No	6	7	7	Medtr6g03330	74.1	0.0	73.8	84.9	*AtMLO6*	63	0.0	62.3	76.4
LaMLO2	PF03094	MLO-related	mb	Cell mb	No	7	7	7	Medtr6g03330	73.2	0.0	72.9	84.1	*AtMLO6*	61	0.0	60.6	75.5
LaMLO3	PF03094	MLO-related	mb	Cell mb	No	7	6	7	Medtr5g022400	76.4	0.0	66.6	75.6	*AtMLO4*	50	1e^{-124}	40.8	55.7
LaMLO4	PF03094	MLO-related	mb	Cell mb	No	7	6	7	Medtr2g093750	68.2	0.0	63.9	75.5	*AtMLO6*	60	0.0	56.7	70.5
LaMLO5	PF03094	MLO-related	mb	Cell mb	No	7	6	7	Medtr2g028470	66.0	0.0	63.6	71.5	*AtMLO13*	55	1e^{-142}	52.0	64.8
LaMLO6	PF03094	MLO-related	PM	Cell mb	No	7	6	7	Medtr3g115960	75.4	0.0	73.1	80.1	*AtMLO5*	62	1e^{-167}	57.4	72.3
LaMLO7	PF03094	MLO-related	PM	Cell mb	No	7	7	7	Medtr3g115960	75.0	0.0	74.6	82.3	*AtMLO5*	57	1e^{-171}	57.2	70.9
LaMLO8	PF03094	MLO-related	PM	Cell mb	Yes	9	8	7	Medtr5g024700	70.7	0.0	68.0	79.0	*AtMLO8*	58	0.0	56.9	71.4
LaMLO9	PF03094	MLO-related	mb	Cell mb	Yes	9	7	7	Medtr8g073130	69.0	0.0	65.7	76.1	*AtMLO8*	60	0.0	55.4	69.0
LaMLO10	PF03094	MLO-related	mb	Cell mb	Yes	8	7	7	Medtr5g024700	69.1	0.0	67.2	77.4	*AtMLO8*	59	1e^{-177}	54.9	68.9
LaMLO11	PF03094	MLO-related	PM	Cell mb	Yes	8	7	7	Medtr8g073130	75.6	0.0	68.9	78.1	*AtMLO8*	61	0.0	57.6	71.8
LaMLO12	PF03094	MLO-related	PM	Cell mb	No	6	4	7	Medtr2g006280	79.4	0.0	78.4	85.3	*AtMLO4*	67	0.0	61.0	74.5
LaMLO13	PF03094	MLO-related	PM	Cell mb	No	7	7	7	Medtr3g118190	75.2	0.0	74.9	84.6	*AtMLO1*	71	0.0	66.9	76.5
LaMLO14	PF03094	MLO-related	mb	Cell mb	No	6	7	7	Medtr8g010530	73.0	0.0	72.2	83.7	*AtMLO1*	65	0.0	65.0	75.5

Gene name	Interproscan prediction		Subcellular localization[a]		SP[b]	TM domain[c]			Identity with *M. truncatula*					Identity with *A. thaliana*				
									M. truncatula gene name	Blastp hit		Pair-wise (%)		A. thaliana gene name	Blastp hit		Pair-wise (%)	
	Pfam Ref.	name	Psort	Plant-mPloc		T	P	I		Id.	E-value	Id.	Sim.		Id.	E-value	Id.	Sim.
LaMLO15	PF03094	MLO-related	mb	Cell mb	No	7	7	7	Medtr2g079500	82.6	0.0	82.4	89.6	*AtMLO11*	69	0.0	67.9	78.5
***Arachis* spp.**																		
ArMLO1	PF03094	MLO-related	PM	Cell mb	Yes	8	7	7	Medtr8g073130	73.0	0.0	66.9	77.3	*AtMLO8*	63	0.0	58.6	70.7
ArMLO2	PF03094	MLO-related	mb	Cell mb	No	7	7	7	Medtr8g010530	72.5	0.0	71.1	82.2	*AtMLO1*	69	0.0	65.9	74.5
ArMLO3	PF03094	MLO-related	mb	Cell mb	No	7	6	7	Medtr4g079140.	60.7	0.0	59.9	72.0	*AtMLO6*	41	$1e^{-124}$	39.3	57.1
ArMLO4	PF03094	MLO-related	mb	Cell mb	No	6	6	7	Medtr2g028470	67.4	0.0	63.9	73.8	*AtMLO13*	56	$1e^{-152}$	54.1	67.2
ArMLO5	PF03094	MLO-related	mb	Cell mb	No	6	7	7	Medtr2g006280	82.3	0.0	82.0	88.0	*AtMLO4*	66	0.0	66.6	80.0
ArMLO6	PF03094	MLO-related	PM	Cell mb	No	7	7	7	Medtr5g022400	73.5	0.0	73.7	84.0	*AtMLO4*	50	$1e^{-125}$	44.0	60.3
ArMLO7	PF03094	MLO-related	mb	Cell mb	No	7	6	7	Medtr3g115940	74.1	0.0	74.6	84.5	*AtMLO12*	51	$1e^{-155}$	47.5	65.0
ArMLO8	PF03094	MLO-related	mb	Cell mb	No	7	7	7	Medtr3g115960	72.6	0.0	72.7	80.3	*AtMLO5*	56	$1e^{-169}$	57.4	69.8
ArMLO9	PF03094	MLO-related	PM	Cell mb	No	7	7	7	Medtr2g093750	63.4	0.0	61.3	73.4	*AtMLO6*	56	$1e^{-177}$	54.4	68.3
ArMLO10	PF03094	MLO-related	mb	Cell mb	No	7	7	7	Medtr3g118190	79.6	0.0	79.0	85.9	*AtMLO1*	71	0.0	66.6	74.5
ArMLO11	PF03094	MLO-related	mb	Cell mb	No	6	7	7	Medtr2g079500	84.0	0.0	84.0	90.6	*AtMLO11*	71	0.0	69.2	79.6
ArMLO12	PF03094	MLO-related	mb	Cell mb	No	7	7	7	Medtr8g010530	63.5	0.0	62.4	76.8	*AtMLO1*	63	$1e^{-177}$	59.0	71.1
ArMLO13	PF03094	MLO-related	mb	Cell mb	No	5	6	7	Medtr6g033330	69.7	0.0	68.5	81.6	*AtMLO6*	60	0.0	59.6	74.2
ArMLO14	PF03094	MLO-related	mb	Cell mb	No	6	7	7	Medtr2g079500	80.6	0.0	74.7	82.3	*AtMLO11*	70	0.0	63.6	74.9
Cajanus cajan																		
CcMLO1	PF03094	MLO-related	mb	Cell Mb	No	6	7	7	Medtr6g033330	74.0	0.0	73.0	84.1	*AtMLO12*	62	0.0	61.1	72.8
CcMLO2	PF03094	MLO-related	PM	Cell Mb	No	7	7	7	Medtr6g033330	62.3	0.0	62.0	74.6	*AtMLO6*	56	0.0	56.0	68.9
CcMLO3	PF03094	MLO-related	mb	Cell Mb	No	7	6	7	Medtr6g033330	71.1	0.0	70.4	80.3	*AtMLO12*	60	0.0	59.8	71.9
CcMLO4	PF03094	MLO-related	PM	Cell Mb	No	7	7	7	Medtr5g022400	76.7	0.0	76.7	85.1	*AtMLO4*	51	$1e^{-132}$	44.1	60.8
CcMLO5	PF03094	MLO-related	mb	Cell Mb	No	7	6	7	Medtr2g093750	71.4	0.0	69.2	77.6	*AtMLO6*	59	0.0	55.1	68.5
CcMLO6	PF03094	MLO-related	mb	Cell Mb	No	7	7	7	Medtr2g093750	70.3	0.0	67.8	76.8	*AtMLO6*	57	0.0	57.8	71.5
CcMLO7	PF03094	MLO-related	mb	Cell Mb	No	7	7	7	Medtr2g093750	70.6	0.0	66.4	78.0	*AtMLO6*	61	0.0	58.3	73.3
CcMLO8	PF03094	MLO-related	mb	Cell Mb	No	7	7	7	Medtr2g028470	72.3	0.0	67.2	76.7	*AtMLO13*	57	$1e^{-145}$	53.7	67.5
CcMLO9	PF03094	MLO-related	PM	Cell Mb	Yes	9	7	7	Medtr5g024700	76.6	0.0	73.7	83.4	*AtMLO8*	59	0.0	58.3	72.3

Gene name	Interproscan prediction		Subcellular localization[a]		SP[b]	TM domain[c]			Identity with *M. truncatula*					Identity with *A. thaliana*				
									M. truncatula gene name	Blastp hit		Pair-wise (%)		A. thaliana gene name	Blastp hit		Pair-wise (%)	
	Pfam Ref.	name	Psort	Plant-mPloc		T	P	I		Id.	E-value	Id.	Sim.		Id.	E-value	Id.	Sim.
CcMLO10	PF03094	MLO-related	mb	Cell Mb	No	7	6	7	Medtr3g115960	80.6	0.0	80.7	87.2	*AtMLO5*	59	$1e^{-180}$	59.6	75.2
CcMLO11	PF03094	MLO-related	mb	Cell Mb	No	7	7	7	Medtr3g115940	79.5	0.0	79.5	88.4	*AtMLO6*	49	$1e^{-151}$	46.7	66.3
CcMLO12	PF03094	MLO-related	PM	Cell Mb	Yes	8	7	7	Medtr8g073130	77.3	0.0	69.1	77.2	*AtMLO8*	64	0.0	60.5	72.4
CcMLO13	PF03094	MLO-related	PM	Cell Mb	No	6	6	7	Medtr2g006280	83.6	0.0	83.4	89.2	*AtMLO4*	64	0.0	65.4	49.6
CcMLO14	PF03094	MLO-related	mb	Cell Mb	No	7	7	7	Medtr3g118190	78.1	0.0	78.8	87.2	*AtMLO1*	71	0.0	66.8	75.8
CcMLO15	PF03094	MLO-related	mb	Cell Mb	No	7	7	7	Medtr8g010530	76.6	0.0	76.2	84.8	*AtMLO1*	64	0.0	65.0	73.8
CcMLO16	PF03094	MLO-related	mb	Cell Mb	No	7	5	7	Medtr4g079140	69.0	0.0	68.2	77.9	*AtMLO12*	42	$1e^{-130}$	42.4	60.4
CcMLO17	PF03094	MLO-related	mb	Cell Mb	No	7	7	7	Medtr2g079500	86.7	0.0	86.8	92.3	*AtMLO11*	71	0.0	70.7	80.6
CcMLO18	PF03094	MLO-related	mb	Cell Mb	No	6	7	7	Medtr2g079500	60.4	0.0	59.9	71.0	*AtMLO11*	55	$1e^{-172}$	55.2	67.3
CcMLO19	PF03094	MLO-related	mb	Cell Mb	No	7	7	7	Medtr6g033330	47.4	$2e^{-257}$	41.6	59.2	*AtMLO3*	52	$1e^{-135}$	46.3	66.2
CcMLO20	PF03094	MLO-related	PM	Cell Mb	No	6	6	7	Medtr6g033330	50.4	$3e^{-164}$	42.8	58.9	*AtMLO6*	49	$1e^{-135}$	42.4	58.2
Phaseolus vulgaris																		
PvMLO1	PF03094	MLO-related	PM	Cell Mb	No	6	7	7	Medtr6g033330	48.3	$2e^{-156}$	40.6	59.0	*AtMLO3*	49	$1e^{-138}$	47.6	66.9
PvMLO2	PF03094	MLO-related	mb	Cell Mb	No	7	7	7	Medtr5g022400	78.4	0.0	78.6	87.4	*AtMLO4*	47	$1e^{-130}$	45.6	61.9
PvMLO3	PF03094	MLO-related	PM	Cell Mb	Yes	9	7	7	Medtr5g024700	71.2	0.0	68.4	78.8	*AtMLO8*	61	0.0	58.3	72.2
PvMLO4	PF03094	MLO-related	PM	Cell Mb	Yes	8	7	7	Medtr8g073130	78.7	0.0	71.7	79.3	*AtMLO8*	61	0.0	60.1	73.2
PvMLO5	PF03094	MLO-related	mb	Cell Mb	No	7	7	7	Medtr6g033330	74.3	0.0	73.9	85.7	*AtMLO12*	64	0.0	64.0	75.6
PvMLO6	PF03094	MLO-related	mb	Cell Mb	No	6	7	7	Medtr6g033330	71.3	0.0	67.7	80.6	*AtMLO12*	63	0.0	58.7	71.6
PvMLO7	PF03094	MLO-related	mb	Cell Mb	No	7	7	7	Medtr2g079500	84.3	0.0	84.1	90.3	*AtMLO11*	71	0.0	69.5	79.1
PvMLO8	PF03094	MLO-related	mb	Cell Mb	No	7	7	7	Medtr2g093750	69.0	0.0	67.9	76.1	*AtMLO6*	58	0.0	57.1	70.4
PvMLO9	PF03094	MLO-related	mb	Cell Mb	No	7	8	7	Medtr2g093750	67.2	0.0	66.4	76.6	*AtMo6*	59	0.0	56.1	69.1
PvMLO10	PF03094	MLO-related	mb	Cell Mb	No	7	6	7	Medtr2g028470	68.5	0.0	63.2	72.9	*AtMLO13*	59	$1e^{-142}$	53.3	67.3
PvMLO11	PF03094	MLO-related	mb	Cell Mb	No	6	7	7	Medtr2g006280	82.4	0.0	76.6	83.9	*AtMLO4*	71	0.0	62.3	73.8
PvMLO12	PF03094	MLO-related	PM	Cell Mb	No	6	7	7	Medtr6g033330	60.6	0.0	59.2	72.9	*AtMLO6*	55	0.0	53.3	69.3
PvMLO13	PF03094	MLO-related	mb	Cell Mb	No	7	7	7	Medtr3g115940	80.0	0.0	78.9	87.7	*AtMLO12*	48	$1e^{-151}$	45.9	62.9
PvMLO14	PF03094	MLO-related	mb	Cell Mb	No	7	6	7	Medtr3g115960	78.9	0.0	78.9	85.4	*AtMLO5*	56	$1e^{-176}$	57.2	69.9

Gene name	Interproscan prediction		Subcellular localization[a]		SP[b]	TM domain[c]			Identity with *M. truncatula*					Identity with *A. thaliana*				
									M. truncatula gene name	Blastp hit		Pair-wise (%)		A. thaliana gene name	Blastp hit		Pair-wise (%)	
	Pfam Ref.	name	Psort	Plant-mPloc		T	P	I		Id.	E-value	Id.	Sim.		Id.	E-value	Id.	Sim.
PvMLO15	PF03094	MLO-related	mb	Cell Mb	No	7	7	7	Medtr3g118190	78.8	0.0	78.5	86.0	*AtMLO1*	70	0.0	65.4	74.2
PvMLO16	PF03094	MLO-related	mb	Cell Mb	No	7	7	7	Medtr8g010530	75.6	0.0	74.6	84.5	*AtMLO1*	69	0.0	65.4	74.7
PvMLO17	PF03094	MLO-related	mb	Cell Mb	No	7	7	7	Medtr2g093750	70.6	0.0	66.3	76.7	*AtMLO6*	60	0.0	59.0	72.3
PvMLO18	PF03094	MLO-related	mb	Cell Mb	No	7	5	9	Medtr4g079140	68.7	0.0	67.0	76.9	*AtMLO12*	42	$1e^{-127}$	40.7	58.4
PvMLO19	PF03094	MLO-related	mb	Cell Mb	No	7	7	7	Medtr2g079500	84.7	0.0	84.5	90.3	*AtMLO11*	71	0.0	69.9	79.1
Vigna radiata																		
VrMLO1	PF03094	MLO-related	mb	Cell mb	No	7	7	7	Medtr6g033330	74.7	0.0	73.4	84.5	*AtMLO6*	64	0.0	62.9	76.4
VrMLO2	PF03094	MLO-related	mb	Cell mb	No	7	7	7	Medtr2g093750	69.8	0.0	66.4	76.7	*AtMLO6*	60	0.0	59.3	72.2
VrMLO3	PF03094	MLO-related	PM	Cell mb	No	8	5	9	Medtr4g079140	68.5	0.0	63.4	73.4	*AtMLO12*	42	$1e^{-127}$	38.3	55.7
VrMLO4	PF03094	MLO-related	mb	Cell mb	No	6	7	7	Medtr6g033330	47.8	$2e^{-160}$	42.5	59.4	*AtMLO3*	52	$1e^{-138}$	45.9	65.4
VrMLO5	PF03094	MLO-related	mb	Cell mb	No	7	8	9	Medtr2g093750	67.3	0.0	66.7	75.5	*AtMLO6*	58	0.0	57.5	71.4
VrMLO6	PF03094	MLO-related	mb	Cell mb	No	7	6	7	Medtr2g093750	69.1	0.0	67.9	76.8	*AtMLO6*	56	0.0	55.7	70.8
VrMLO7	PF03094	MLO-related	mb	Cell mb	No	7	6	7	Medtr3g115940	79.8	0.0	78.4	87.1	*AtMLO12*	49	$1e^{-152}$	46.9	62.4
VrMLO8	PF03094	MLO-related	mb	Cell mb	No	7	6	7	Medtr3g115960	81.0	0.0	81.3	86.9	*AtMLO5*	60	$1e^{-177}$	60.6	73.4
VrMLO9	PF03094	MLO-related	mb	Cell mb	No	7	6	6	Medtr3g118190	79.3	0.0	64.7	71.9	*AtMLO1*	70	0.0	53.5	60.9
VrMLO10	PF03094	MLO-related	PM	Cell mb	Yes	8	7	7	Medtr8g073130	79.3	0.0	76.7	84.0	*AtMLO8*	61	0.0	60.8	74.1
VrMLO11	PF03094	MLO-related	mb	Cell mb	No	7	7	7	Medtr6g033330	64.7	0.0	63.8	75.8	*AtMLO6*	56	0.0	55.1	70.9
VrMLO12	PF03094	MLO-related	mb	Cell mb	No	6	7	7	Medtr8g010530	74.8	0.0	74.1	83.7	*AtMLO1*	65	0.0	66.7	74.6
VrMLO13	PF03094	MLO-related	mb	Cell mb	No	6	6	7	Medtr2g006280	81.0	0.0	80.6	86.9	*AtMLO4*	71	0.0	42.1	61.4
VrMLO14	PF03094	MLO-related	mb	Cell mb	No	7	7	7	Medtr2g028470	69.2	0.0	63.4	73.6	*AtMLO13*	59	$1e^{-141}$	53.4	67.6
VrMLO15	PF03094	MLO-related	mb	Cell mb	No	8	6	7	Medtr5g024700	73.3	0.0	68.3	78.3	*AtMLO8*	60	0.0	56.2	69.9
VrMLO16	PF03094	MLO-related	PM	Cell mb	No	7	7	7	Medtr5g022400	78.4	0.0	78.5	87.0	*AtMLO4*	49	$1e^{-130}$	44.9	60.8
VrMLO17	PF03094	MLO-related	mb	Cell mb	No	7	7	7	Medtr6g033330	73.6	0.0	66.8	78.8	*AtMLO12*	65	0.0	58.9	70.6
VrMLO18	PF03094	MLO-related	mb	Cell mb	No	7	7	7	Medtr2g079500	83.8	0.0	83.8	89.9	*AtMLO11*	70	0.0	68.7	78.3

Supplementary Table S4. Conservation in legume sequences of the 73 amino acids previously found to discriminate between monocot and eudicot powdery mildew susceptibility proteins[18]. The comparison was performed by aligning the monocot and dicot powdery mildew susceptibility proteins as in Appiano *et al.*[18] with the corresponding legume MLO sequences from clades IV and V. Colored residues highlight residue varying between monocot and eudicot plant species (red), between clades (blue) or both (green), respectively.

**Localization**	**TM1**					**EC1**			**TM3**			**IC2**										
**Position in *HvMLO1***	**13**	**25**	**27**	**28**	**32**	**91**	**96**	**125**	**143**	**145**	**146**	**197**	**198**	**178**	**183**	**184**	**185**	**188**	**189**	**194**	**195**	**197**
**Monocot (Cl. IV)**	**P**	**A**	**M/I**	V/I	**V**	A	**W/L/R**	**G**	T/L	**S**	V	**A/T**	S	**A/S**	T	**H**	**Q**	**V**	K	**G**	L	**S**
**Legume Cl. IV**	**Q/K/E**	**A/S**	**F**	V/I	**V**	A/L	**L**	**S**	F/L	**S**	V	**S/T**	S	**A/S**	T/A	**H**	**Q**	**V**	R/K	**G**	W/L	**R**
**Eudicot (Cl. V)**	**T/E/D**	**F**	L/M	L	**I**	V/I/L	**H**	Y	L/I/F	**C**	I	**K/R**	T	**E**	A/T	**R**	**D**	**G**	R	**S/N/H/**	W	Q/K
**Legume Cl. V**	**T/E/D/Q**	**F/L**	L/M	L/V/I	**I**	V/I/A	**H/N**	Y/E/D/G	L/I	**C**	I/V	**K/R**	T/S	**E**	A	**R/K**	**D/E**	**G**	R/Q	**S/N**	W	Q/K/R

Supplementary Table S4 (continued)

**Localization**	**IC2**										**TM4**				**EC2**				**TM5**		
**Position in *HvMLO1***	**198**	**200**	**202**	**206**	**225**	**229**	**233**	**234**	**241**	**245**	**250**	**260**	**261**	**267**	**271**	**272**	**273**	**274**	**276**	**278**	**279**
**Monocot (Cl. IV)**	**T**	**G**	**R**	**A**	**A/Q**	**N**	S	**Q**	H	K	**D**	**L**	P	**I**	**F**	L	**D**	**I**	**G**	**G**	**T**
**Legume Cl. IV**	**M/K**	**G**	**R**	**A**	**H**	**N**	A	**P**	H/Q	K	**D**	**I/M**	P	**I**	**L**	L/M/V	**N**	**V**	**K**	**Y**	**T**
**Eudicot (Cl. V)**	S/N/T	**V/I/F**	**L/M/I**	**C**	**H**	**M/I**	A/T/P	**P**	R/Q	Q/S/N	**E/K/Q**	**P**	A/V/T/L	**V**	**L**	T	**N/T**	**T/S**	G	**Y/H**	**S**
**Legume Cl. V**	S/N/T	**V/I**	**L/M/I/V**	**C/S**	**H**	**M/T/V**	A/T/G/S	**P**	R/Q/H	E/Q/K	**E/K**	**P**	I/V/T/P	**V**	**L**	T/F/L	**N/H**	**T**	G	**Y/H**	**S**

Supplementary Table S4 (continued)

**Localization**	**TM5**				**IC3**										**TM6**			**EC3**				
**Position in *HvMLO1***	**280**	**284**	**285**	**291**	**303**	**306**	**308**	**313**	**314**	**325**	**326**	**327**	**335**	**336**	**344**	**352**	**354**	**358**	**360**	**361**	**362**	**366**
**Monocot (Cl. IV)**	**L**	**S**	**F**	**L**	**M**	A	**E**	**A**	**S/T**	**S**	**N**	**K**	D	**W**	**I/T**	M	H	T	**A**	**T**	**P**	**K/D**
**Legume Cl. IV**	**L**	**S**	**V**	**L**	**M**	A	**E/Q**	**A/T**	**T**	**N**	**N**	**K**	Q/H	**W**	**T/I**	I/M	F	T	**Y**	**E**	**F**	**S**
**Eudicot (Cl. V)**	**Y**	**P**	F	**I**	**T/S**	G	**R/K**	**G**	**D**	**G**	**D**	**D/H**	R/G	**F/L**	**V**	L/V	F	S	**W/Y/L**	E/K/D	**F**	**S/N**
**Legume Cl. V**	**Y**	**P**	F	**I/V**	**T**	G/A	**R/K**	**G**	**D/E**	**G**	**D**	**E/D/H**	R/G/D/H	**F/L**	**V**	L/V/M	F/H/Y	S/T/A	**L/Y/F**	E/Q/D	**F**	**S/N**

Supplementary Table S4 (continued)

Localization	EC3			TM7	C-TERMINAL					CaMBD							
**Position in *HvMLO1***	**374**	**377**	**378**	**394**	**406**	**409**	**413**	**415**	**416**	**418**	**420**	**421**	**429**	**430**	**431**	**434**	**435**
**Monocot (Cl. IV)**	**L**	**M**	K	**F**	**N/H**	**R/K**	**E/D**	**Q**	**T**	K	**M/T**	**R**	E	K	K	R	D
**Legume Cl. IV**	**L**	**T/V**	R	**F**	**H**	**R/K**	**E**	**Q**	**T**	K	**K**	**Q**	D/E	K/R	K/R/T	R	K
**Eudicot (Cl. V)**	**D**	I	R	**L**	**T/S/K**	**P**	**G/N**	**N/R**	**V**	T	**K**	**H**	K	R/Q/H	V/T/I	G/N	R/K
**Legume Cl. V**	**D/E**	I/V	R/K	**L**	**T/S**	**P/A/H**	**N**	**N/R**	**V/I/L**	T/S/K/E	**K/R**	**H**	K/N/E	N/Q/H	V/K/I	G/N/S/K/R	R/K/D

Supplementary Figure S1. Alignment *of M. truncatula* MLO protein sequences. Alignment was obtained with ClustalW[34]with default parameters. Shading represents amino acid conservation at 90%, 70% and 50% respectively. Red Asterisks on top of the alignment show the 30 highly conserved residues previously detected in MLO sequences[33]. MEME conserved motifs are indicated by numbered horizontal bars on top of the alignment while the transmembrane domains (dashed line) and the CaMBD motif are represented below the alignment. Numbered horizontal bar in red represent the localization of the most significant motifs identified previously by Deskmuth *et al.*[15].

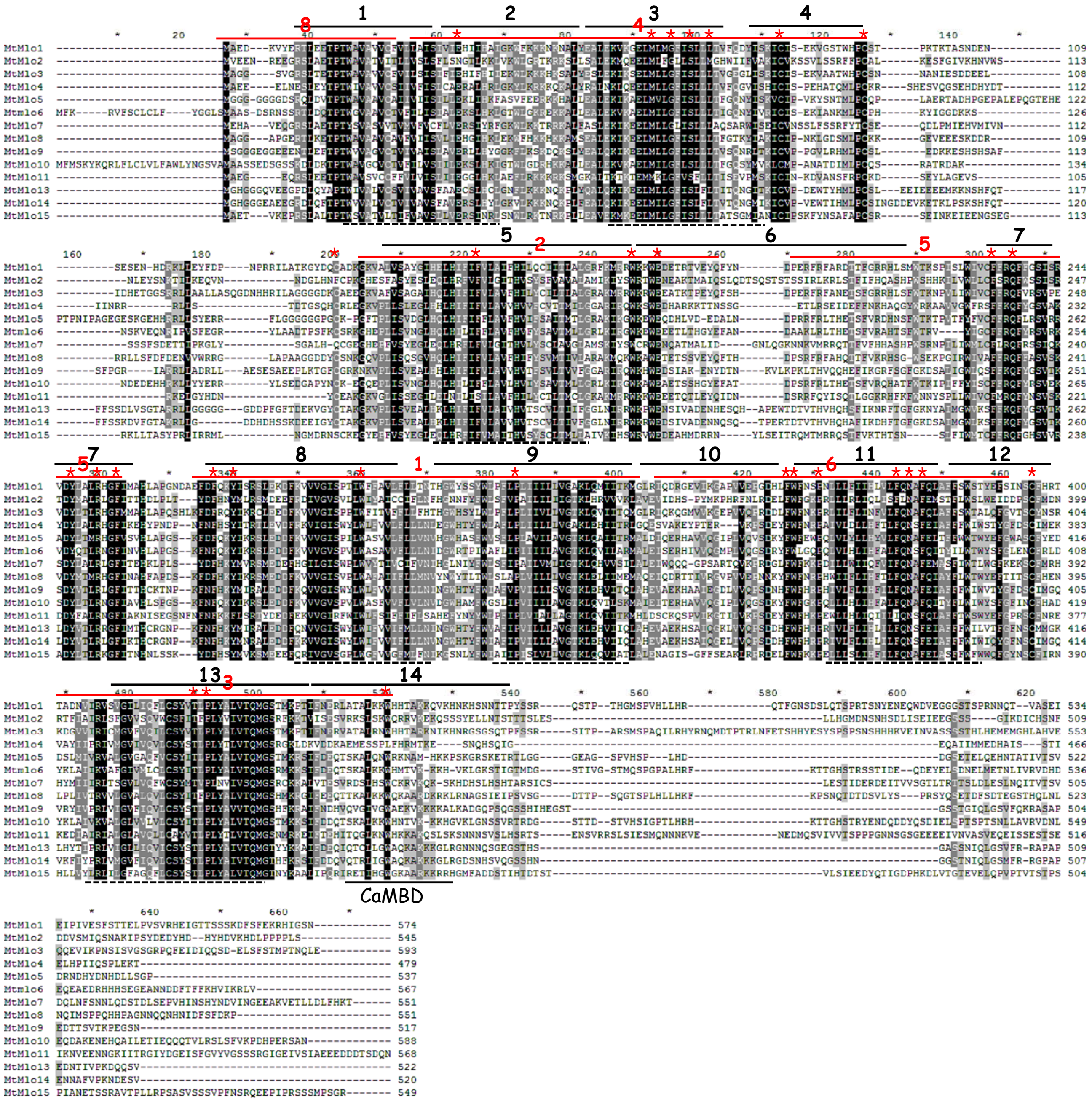

Supplementary Figure S2. Phylogenetic relationship of MLO protein sequences. The phylogenetic tree includes all MLO sequences identified in this study with those of *Arabidopsis thaliana, Cucumis sativus, Solanum lycopersicum, Vitis vinifera* and *Hordeum vulgare* (Table 1). The unrooted tree was built with the Neighbor-Joining method on MEGA 6 software[48] and reveals seven well-supported clades. Clade I MLO appears further subdivided in two distinct groups (Ia and Ib). Numbers above branches indicate the percentage of bootstrap replicates supporting this branch. Only bootstrap values higher than 75 are represented.

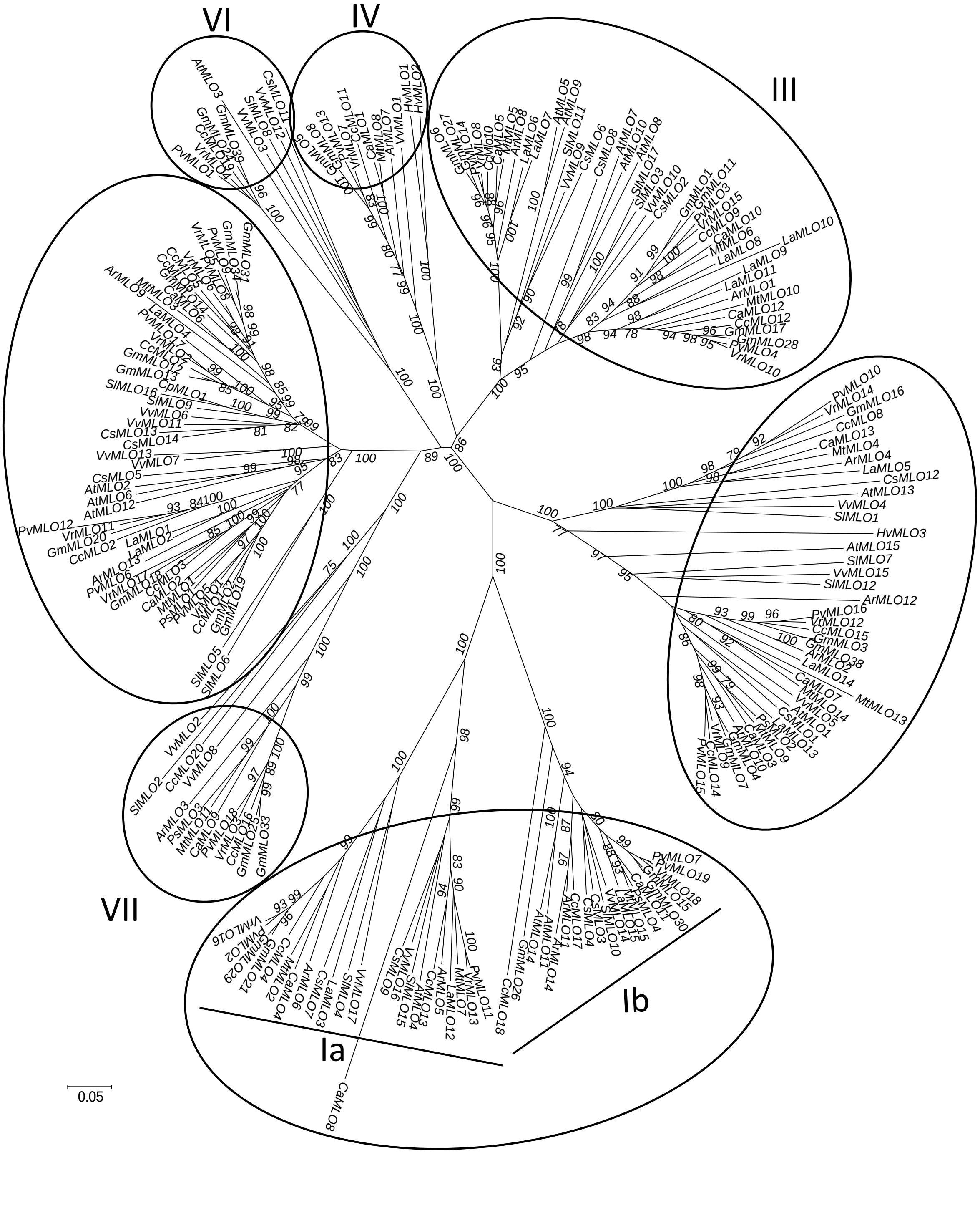

Supplementary Figure S3. Alignment of Clade I MLO protein sequences from legume species. Alignment was obtained with ClustalW[34] with default parameters. Shading represents amino acid conservation at 90%, 70% and 50% respectively. MLO-specific motifs are indicated by black horizontal lines. Clade I-specific MEME motifs are shown in red boxes. Transmembrane domains (green dashed lines) and the CaMBD domain (green line) are represented below the alignment. Red asterisks on top of the alignment show the 30 highly conserved residues previously detected in MLO sequences[33].

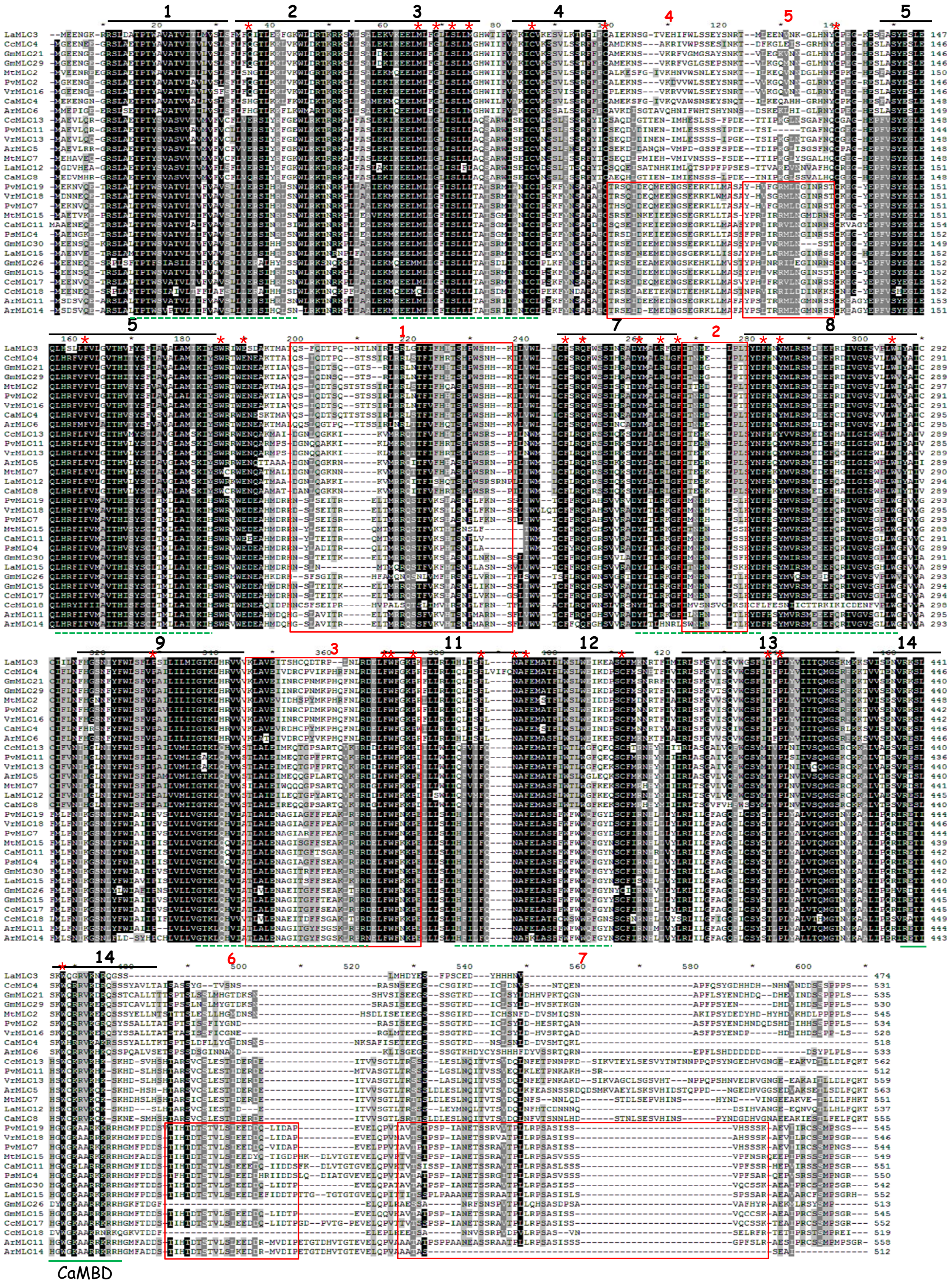

Supplementary Figure S4. Alignment of Clade II MLO protein sequences from legume species. Alignment was obtained with ClustalW[34] with default parameters. Shading represents amino acid conservation at 90%, 70% and 50% respectively. MLO-specific motifs are indicated by black horizontal lines. Clade II-specific MEME motifs are shown in red boxes. Transmembrane domains (green dashed lines) and the CaMBD domain (green line) are represented below the alignment. Red asterisks on top of the alignment show the 30 highly conserved residues previously detected in MLO sequences[33].

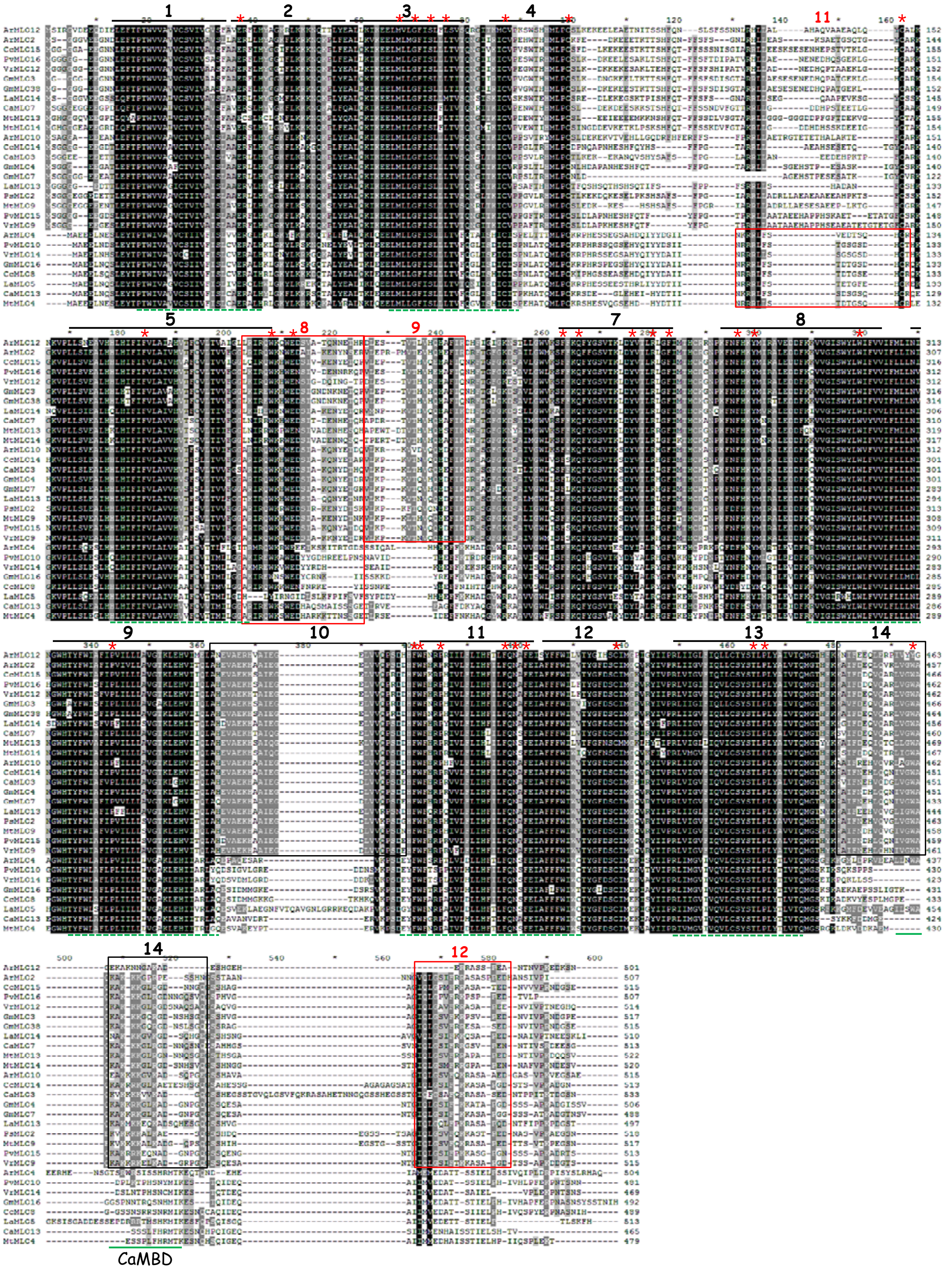

Supplementary Figure S5. Alignment of Clade III MLO protein sequences from legume species. Alignment was obtained with ClustalW[34] with default parameters. Shading represents amino acid conservation at 90%, 70% and 50% respectively. MLO-specific motifs are indicated by black horizontal lines. Clade III-specific MEME motifs are shown in red boxes. Transmembrane domains (green dashed lines) and the CaMBD domain (green line) are represented below the alignment. Red asterisks on top of the alignment show the 30 highly conserved residues previously detected in MLO sequences[33].

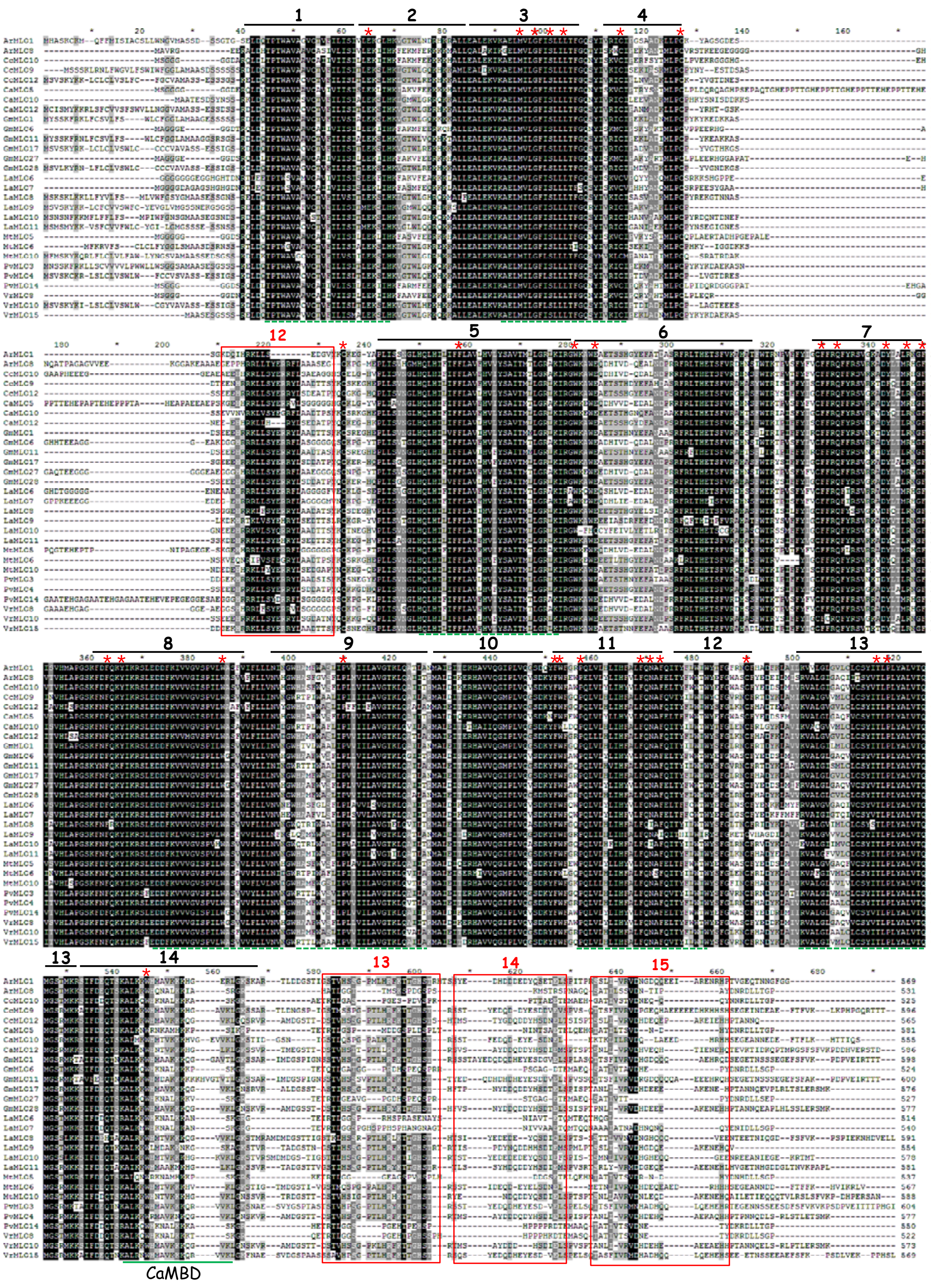

Supplementary Figure S6. Alignment of Clade IV MLO protein sequences from legume species. Alignment was obtained with ClustalW[34] with default parameters. Shading represents amino acid conservation at 90%, 70% and 50% respectively. MLO-specific motifs are indicated by black horizontal lines. Clade IV-specific MEME motifs are shown in red boxes. Transmembrane domains (green dashed lines) and the CaMBD domain (green line) are represented below the alignment. Red asterisks on top of the alignment show the 30 highly conserved residues previously detected in MLO sequences[33]. A motif similar to the [DE]FSF tetrad previously identified in Class V MLO sequences by Panstruga[35] is also shown in numbered blue horizontal line below the alignment.

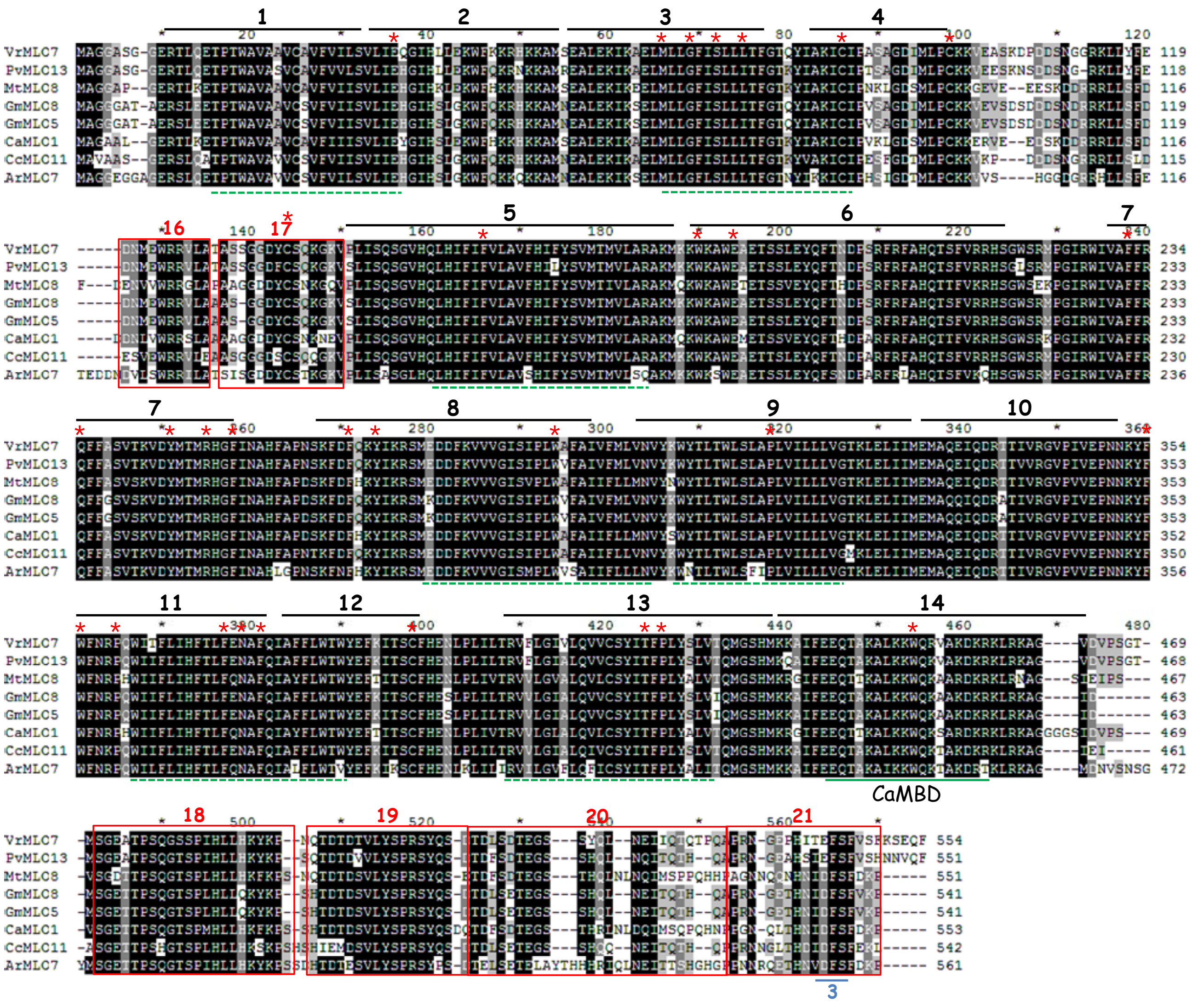

Supplementary Figure S7. Alignment of Clade V MLO protein sequences from legume species. Alignment was obtained with ClustalW[34] with default parameters. Shading represents amino acid conservation at 90%, 70% and 50% respectively. MLO-specific motifs are indicated by black horizontal lines. Clade V-specific MEME motifs are shown in red boxes. Transmembrane domains (green dashed lines) and the CaMBD domain (green line) are represented below the alignment. Red asterisks on top of the alignment show the 30 highly conserved residues previously detected in MLO sequences[33]. Conserved motifs identified previously from clade V MLO sequences[16,35] are also indicated by blue lines below the alignment.

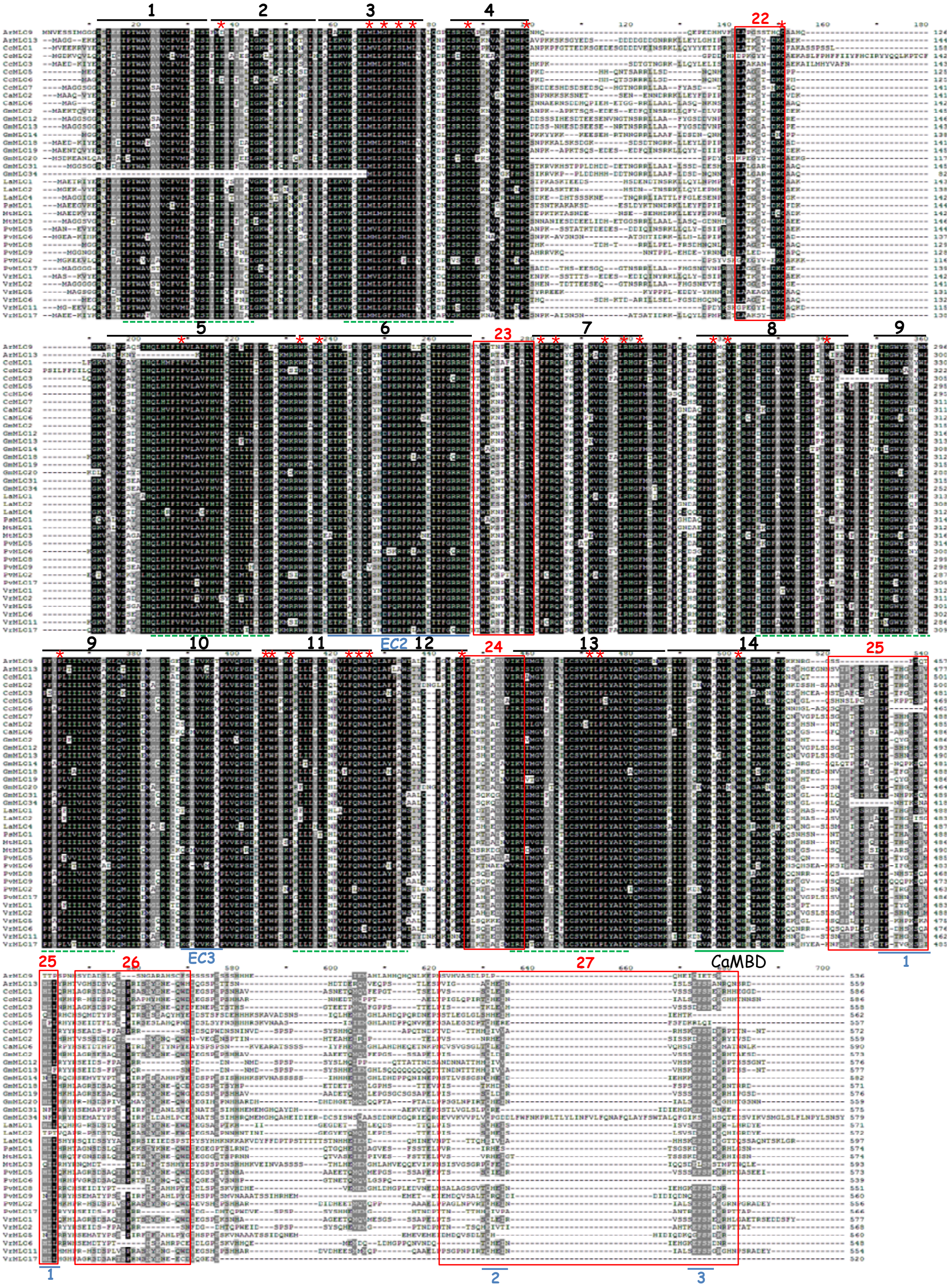

Supplementary Figure S8. Alignment of Clade VI MLO protein sequences from legume species. Alignment was obtained with ClustalW[34] with default parameters. Shading represents amino acid conservation at 90%, 70% and 50% respectively. MLO-specific motifs are indicated by black horizontal lines. Clade VI-specific MEME motifs are shown in red boxes. Transmembrane domains (green dashed lines) and the CaMBD domain (green line) are represented below the alignment. Red asterisks on top of the alignment show the 30 highly conserved residues previously detected in MLO sequences[33]. A motif similar to the [DE]FSF tetrad previously identified in Class V MLO sequences by Panstruga[35] is also shown in numbered blue horizontal line below the alignment.

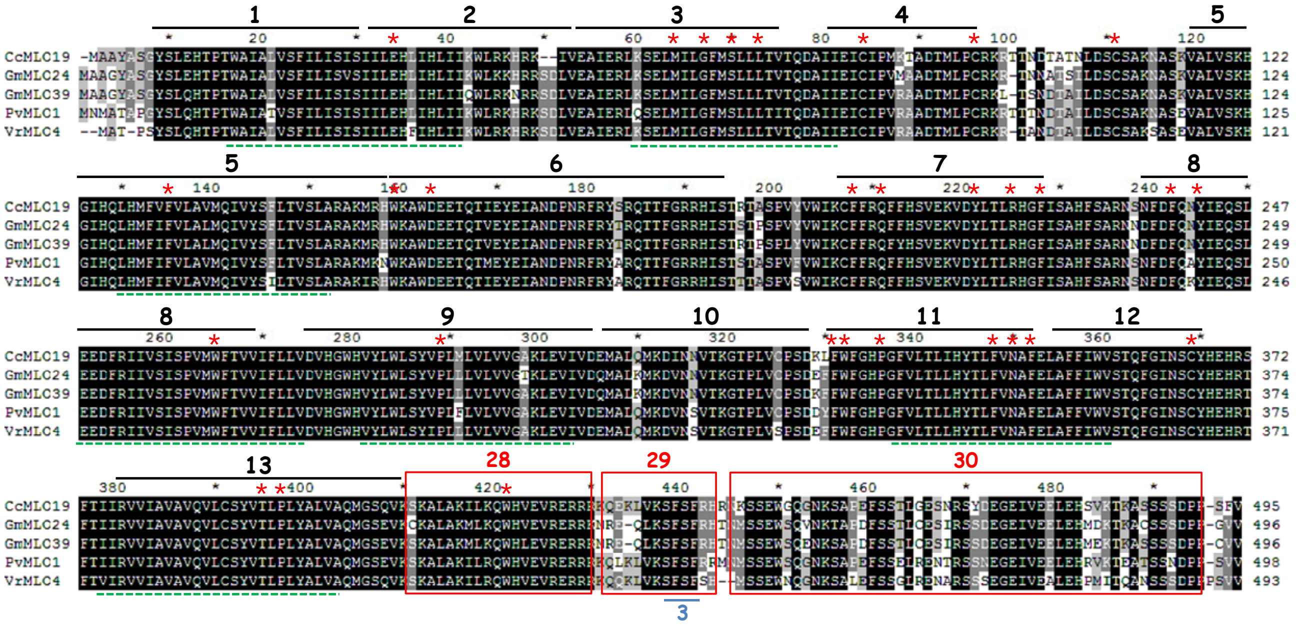

Supplementary Figure S9. Alignment of Clade VII MLO protein sequences from legume species. Alignment was obtained with ClustalW[34] with default parameters. Shading represents amino acid conservation at 90%, 70% and 50% respectively. MLO-specific motifs are indicated by black horizontal lines. Clade VII-specific MEME motifs are shown in red boxes. Transmembrane domains (green dashed lines) and the CaMBD domain (green line) are represented below the alignment. Red asterisks on top of the alignment show the 30 highly conserved residues previously detected in MLO sequences[33].

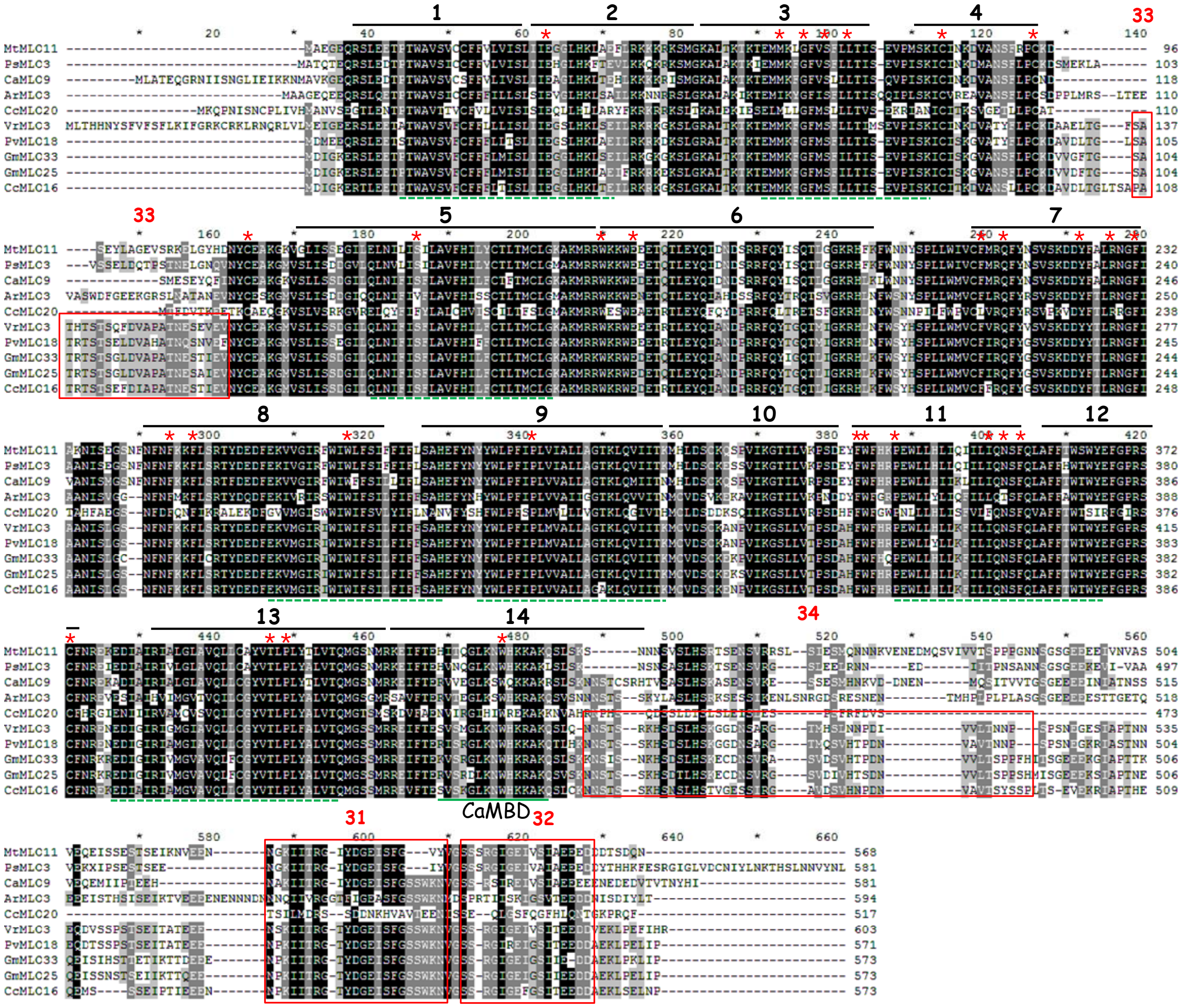

Supplementary Figure S10. List of legume *MLO* CDS sequences.

>ArMLO1 ARADU.27DYI
ATGCATGCCTCAAAGTGCAAAATGCAGTTTTTTATGATCAGCATAGCGTGTAGCTTGTTATGGAATGGTGTAATGGCGTC
AAGTGATAGCAGTGGGACTGGGAGTGAACTTGATCAAACACCAACTTGGGCTGTGGCTGGTGTCTGCACTGTCTTCATCT
TAATTTCCATTGTTCTTGAGAAGGGTCTTCACAAAGTTGGAACGTGGCTAAATGATAGGCACAAGAGGGCTCTGCTTGAA
GCTCTTGAGAAAGTCAAAGCTGAGTTGATGGTTCTAGGCTTCATTTCTCTGCTTTTGACATTCGGGCAGAGTTACATTGT
TAGAATTTGTATTCCTGGCAGTGCCGCGGACAAACTGTTGCCATGTAAATATGCAGGTAGCGGCGATGAGAGTAGCGGCA
AAGATCAAATTCATAGGAAACTTCTATCAGAAGATGGAGTTTATAAATGCAAGGAGGGATACGCGCCGCTTATATCAAGC
AATGGGTTGCACCAGTTACACATCCTCATATTCTTCTTGGCAGTTCTTCATGTGCTTTACAGTGCTGTCACAATGCTTCT
TGGGAGACTGAAGATACGCGGGTGGAAGGCATGGGAGGCAGAGACTTCATCTCATGGCTATGAGTTTGCTACTGATGCTT
CGAGATTCCGGCTAACTCATGAAACATCGTTTGTGAAAGCCCATGCAACTTTTTGGACTAGAAATCCCGTCTTCTTCTAT
ATCGGTTGCTTCTTTAGACAGTTTTATAGGTCTGTGGGTAAAGCTGACTACTTAGCTCTTCGCAATGGTTTTATCAGTGT
CCATATGGCTCCTGGAAGTAAATTTGACTTCCAAAAGTATATCAAAAGATCCTTGGAGGACGACTTCAAGATAGTTGTTG
GAGTCAGTCCTGTCCTCTGGGCGTCGTTCGTCATTTTTCTACTACTAAATATTAATGGATGGCATGCTATGTTTTGGGCA
TCCTTAATTCCTGTCGTGATAATTTTGGCTGTTGGAACAAAACTTCAAGCCACATTAGCAAATATGGCTATAGAAATCAC
AGAAAAGCATGCTGTTGTCCAAGGGATTCCTCTTGTTCAGGGCTCTGACCAATATTTTTGGTTCGGTCGGCCTCAGTTAG
TTCTTCATCTTATTCACTTTGCTTTGTTCCAGAATGCATTCCAAATAACATATTTCTTGTGGATATGGTACACTTTTGGG
TTTAGAAACTGTTTTCATGCTGACTTCAAGCTTGCAATACTTAAAGTAGTTTTAGGGCTTGGAGTGCTATGCCTATGCAG
TTATATCACGCTTCCGTTATACGCTCTTGTTACTCAGATGGGCTCAACAATGAAAAGATCAATATTTGATGAACAAACAT
CTAAGGCGCTAAAGAAATGGCACATGGCTGTGAAAAAGAAGCATGGAGAGAGGCTAGGGAAGTCGAAGGCGCGAACCTTG
GACGACGGAAGTACCATTGGTTCGACGGTGCATTCTTCAGGACCAATGCTACACCGCTTCAAAACCACCGGCCACTCGAC
ACGGCACACTTCATCCTATGAGGACCATGATGATGACGAAGATTATCAATCTGAGACTGAATTGTCTCCCATTACTCCAA
GATCAAGCTTGATAGTGAGAGTGGATAATGGTGACCAGCAAGAAGAAATAGCAAGAGAAAATAGGCACCCCACTGTTGGA
GAACAAACCAATAATGGTTTTGGAGGTTAA

>ArMLO2 ARAIP_Y3LT4
ATGAGCGGCGGAGGCGTGGAAGAAGGTAACGATTTGGAGTTCACTCCGACGTGGGTTGTGGCCGTCGTTTGCTCCGTCAT
CGTCGCCGTTTCCCTCCTCGCCGAGCGTCTTCTTCACTATGCCGGAAAATTTCTTAAGAGGAAGAACCAGAAGCCACTCT
TTGAAGCTCTCCTCAAAGTCAAAGAAGAGTTGATGCTGATGGGATTTATATCTCTGCTTCTGACGGTAACACAAAATGGG
ATCATAAGAATCTGTGTGCCAGAGCATTGGACTCACCACATGCTTCCATGTAAATTTGAGGATAAAGAAGAAGAAACAAA
AACAACATCACATTTTCAGAATTTTTTCTCTTTCTCTGGAAATCTCAGGCACCTTCTGGAAGCTTCCAAATCAGCAGAAA
CAGGGTCCCAAACTGGATATTGCGCTCGGAAGGGCAAGGTGCCTCTATTGTCCATTGAAGCCCTTCATCACTTGCATATC
TTCATTTTTGTGTTAGCTATAGTTCATGTAACCTTTTGTGTTCTCACGGTAGTTTTTGGAGGATTAAGAATACGACAGTG
GAAGAACTGGGAAGACGCTATTGCAAAAGAAAACTATAATTCAGAACGAGTTTTGGAACCAAGGCCAATGGTGACTGAAG
TTCATCAACATGAATTTATAAGGGACCGTTTCACAGGTTTTGGCAAAGATTCCGCTATTTTGGGATGGATGAAATCCTTT
TTCAAGCAATTTTTTGGATCCGTGACGAAATTAGATTATGTGACATTGAGGCTTGGTTTTATTATGACCCACTGCAGGGG
AAATCCAAAGTTTAATTTTCACAAGTATATGATTCGTGCTCTTGAAGATGATTTCAAGAAAGTTGTTGGTATAAGTTGGT
ATCTTTGGATCTTTGTGGTCATCTTCTTGTTGATTAATATTAACGGTTGGCATACATATTTCTGGATCGCTTTCATCCCT
GTCATTGTGCTGCTTGCGGTAGGGACAAAGCTGGAACATGTAATAACCCAATTAGCACATGAAGTGGCAGAGAAGCATGC
AGCCATTGAAGGTGAATTGGTGGTTCAACCATCTGATGATCACTTCTGGTTTCACCGACCTCGCATTGTTCTCTTCTTGA
TTCACTTCATCCTTTTCCAAAATGCTTTTGAGATTGCTTTCTTCTTCTGGATTTGGGTTACTTATGGGTTTGACTCGTGT
ATAATGGGTCAAGTTCGTTACATTGTTCCAAGGCTCATTATTGGGGTATTTATTCAGCTTCTATGTAGTTACAGCACTCT
CCCACTCTATGCTCTCGTTACACAGATGGGAACTCACTTTAAGAAGGCAATATTTGATGAGCAATTACAAGTGAGGCTTG
TTGGTTGGGCACAGAAGGCAAAGAAGAAAGGACCAAGACCTGAAAGTTCTCACAACGGTTCTTCAACCGCCGCTAATAAT
AATGGAGTTCAGCTTGGCTCAATTTTCAGGAGGGCTTCAGCATCTCCCCCAGAAGACCATGCCAATTCCATTGTCCCAAT
CTAA

>ArMLO3 ARADU.65SUF
ATGGCGGCGGGGGAACAAGAAGAGCAAAGAAGCCTTCAAGAAACACCAACATGGGCTGTCTCAATCTGCTGTTTCTTCTT
TATTTTGTTATCTTTGTCCATTGAAGTTGGTTTACACAAATTGAGTGCGATTTTGAAGAAAAATAATCGAAGGTCCCTAG
GGAAGGCTTTGGCTAAGATCAAAACAGAGATGATAAAATATGGATTCATATCGTTTCTTCTGACAATATCGCAACAAATA
CCGTTATCCAAAATCTGTGTGAGAGAGGCTGTGGCAAATTCTTTCCTACCATGTAGCGACCCACCATTGATGAGGAGCTT
AACAGAAGAGGTAGCTTCTTGGGACTTTGGAGAAGAAAAAGGACGTTCATTAAATGCAACAGCAAACGAGGTCAACTATT
GTGAATCAAAGGGCATGGTTAGTCTGATCTCGGATGATGGAATCCAACAATTAAATATCTTCATCGTATTTCTGGCGGTT
TTTCATATATCGTCCTGCACTCTAACAATGTGTCTTGGAATGGCCAAGATGAGAAGATGGAAAAAATGGGAGAACGAGAC
TCAAACACTTGAATACCAAATAGCTCATGATTCCAGTAGGTTCCAATATACCCGTCAAACATCGGTTGGCAAGCGTCATT
TGAACTTTTGGAGTAATTATTCTCCCTTACTTTGGATGGTTTGTTTTTTGAGGCAATTCTATAGGTCAGTCTCAAAAGAT
GATTACTTCACTCTCCGAAATGGATTCATTGCAGCTAATATTTCTGTAGGGGGCAATTTCAACTTCATGAAGTTTCTTTC
CCGTACTTATGATCAGGATTTTGAGAAGATTGTTCGAATCAGGTCTTGGATCTGGATTTTCTCCATACTTTTCATATTCT
TCTCTGCTCATGAGTTCTATAATCATTACTGGCTTCCATTTATTCCCCTTGTGGTTGCTATAATTGGCGGGACAAAACTG
CAAGTGGTTATAACGAACATGTGCGTGGATAGTGTGAAGGAAAAGGCAGTTATCAAAGGAACTATTCTTGTGAAACCCAA
CGATGACTATTTCTGGTTTGGTAGACCTGAATGGCTTCTCTATTTAATTCAGTTCATCCTTCTTCAGACCTCCTTTCAAC
TAGCATTCTTTGCCTGGACTTGGTATGAATTCGGTCCAAGATCATGCTTTAATCGAGAAGTAGAAAGCATTGCCATACAT

GTTATCATGGGCGTAACTGTGCAGATCCTCTGTGGTTATGTCACTCTACCCCTTTATGCTCTTGTTACTCAGATGGGTTC
TGGCATGAGGAGTGCTGTATTCACAGAGCGCGTAACTGAAGGCCTTAAAAGTTGGCACAAAAGGGCCAAACAATCAGTTT
CAAACAACAATTCAACTTCAAGCAAATACTTAGCTTCTTTACATTCCAGAAAAAGTGAATCTTCAATAAAAGAGAATCTA
AGCAACCGAGGTGATTCAAGAGAATCTAATGAAAACACAATGCACCCTCTTCCACTACCTTTGGCTAGTGGAAGTGGTGA
AGAAGAGGAAGAGAGTACTACAGGTGAAACACAAGAGGAAGAAATATCAACTCATTCCATTTCAGAAATCAAAACTGTTG
AGGAAGAAGAGAATGAGAATAATAACGACAACAACAATCAGATTATTGTAAGAGGAGGAACATTTATTGGAGAAGCCTCT
TTTGGAAGTAGCTGGAAAAATATGGACAGTCCAAGAACCATTATTTCAAAAATTGGTTCCGTAACCGAAGAAGATGACAA
CATTAGTGACATATACCTCACTTGA

>ArMLO4 ARADU.N8G7F
ATGGCAGAAGAAGAACTGAATGAGTCTCTGGAATATACACCAACATGGATTGTAGCTGTTGTTTGCACCATCATTGTTTT
TATCTCTCTTGTTGTCGAGCGCGGCCTCCACAAGCTCGGAAAGTACTTCAAGAAAAAGAACCAAACTCCATTATTTGATG
CCTTGCAAAAATTGCAAGAAGAATTGATGCTTTTAGGGTTCATTTCCCTTCTACTAACTGTCTTTCAAACTGCAATAAGC
CACATTTGCATCTCACCTGAGCTTACAACCACAATGCTTCCATGCAAGAGGCCACATGAATCGTCTGAAGGTTCGGCACA
CGATCAGATCTACTATGATGGTATAATCAACAAAAGAAGGCTTTTTTCTGTTGAAGATACTTCTCAGCATTGTAGGAGTA
AGGGGAAGGTTCCACTGTTATCACAAGAATCATTGCATCATTTGCACATTTTCATCTTTGTATTGGCTTTAGTACATGCA
ATATTCTGTGTCACCACATTGTTTCTTGGAATCACAAGGATGCGTCAGTGGAAGAGATGGGAGGAAGAAATTAAGAGCAA
AATAACAAGAACAGGAGATTCTAGTAGTATCCAAGCTCTCCATCACCATGAGTTCTTCAAGAAACACGCCGACGGATATT
GGAGAAGAGCAGCTGTTGTTGGTTGGCTGATATCATTCTTCAAACAATTTTATGGTTCTGTTACTGAATCTGACTACATT
GCGCTGCGTTATGGATTTATCAAGATATCATTCTTCAAACAATTTTATGGTTCTGTTACTGAATCTGACTACATTGCGCT
GCGTTATGGATTTATCAAGGAACATTGCCCGCGCAAACCTCAGTTTGATTTCCACAATTACATGGTGCGGACACTTGAAG
TTGATTTCAGAAGAATTGTAGGCATAAGCTGGTACCTGTGGCTCTTTGTTGTGCTGTTTTTGCTGCTAAATATTGCAGGG
TGGCACACTTATTTCTGGCTTGCCTTTTTACCAGTGATAATTTTGCTTCTTGTGGGGGCAAAGTTAGAGCACATCATAGC
GCGCTTGGCGCAGGAGCCGGCGCAGGAGTCAGCACGTGTGAAGCCGTCAGATGAATACTTCTGGTTTAGCCGCCCAACAC
TTGTCCTTGACTTGCTTCATTTCACTTTATTTCAGAACTCTTTTGAGATTGCATTTTTCTTCTGGATCTGGTGCACATAT
GGATTTGATTCATGCATTATGGAGAAAATATCCTACATCATCCCAAGACTTATAATGGGTGTGATTGTTCAAGTGCTTTG
CAGTTACAGCACCTTGCCTCTATATACTCTTGTTACTCAGATGGGTAGTGGTTTCAAGAAGGGTATGCTCGATCCGCGGG
TGGAGGAAGCCCTCTTGAATTGGGCAGAGGAAAGACATGAGAACTCCGGCACCAGTAGGTGGCGGAGCATATCATCCCAT
AGAATGACCAAAGAACAAACACAAAATGATGAACATGAAATAGCCATGGAGATGGAAGATGCAACAACATCTTCAATTGA
GCTTCCTAGCAGCATTGTCCAAATTCCTTTGGACAGGCCTATTAGTTATTCTTTGAGACATGCTCAATAA

>ArMLO5 ARADU.U7W18
ATGGCGGAGGTGCTTCGACGAGGTCGCTCTCTGGCTGAGACGCCAACATATTCAGTTGCTTCCGTTATTACTGTTATGGT
CTTCGTCTGCTTCGTCGTTGAGCGCTCCATCTACCGCTTTGGAAAGTGGTTGAAGAAGACAAGAAGGAAGGCTCTCTTTG
CATCACTGGAAAAGATTAAGGAAGAGTTGATGCTACTTGGTCTTATCTCTTTGTTGTTGGCACAAAGTGCAAGATGGATC
TCTGAAATATGTGTGAATTCATCACTGTTCAATAGCGAGTTTTATATTTGTTCTGAAAAAGACCTTGACGCGAACCAGAA
TGTCATGCCCGATGGTTCATCCTTTTCCCCTGATGAAACTGATATTCCAAAGGGACTAATTAGTGGTTCATTCAATCAAT
GCCCTGCCGGGCACGAGCCATTTGTTTCATATGAGGGTCTTGAGCAGCTGCACCGGTTCTTGTTTGTTCTTGGGATCACT
CATGTTCTATACAGTTGTCTGGCAGTCGGTTTGGCAATGAGCAAGATTTATAGTTGGCGCAGATGGGAAAATCAGACCAC
AGCTGCCGCGGACGGAAATCTGCAAGGAAAGAGAAACAAGGTGATGAGGCGGCAGATAACCTTTGTTTTTCATCATGCTT
CACATCCATGGAGCAGGAATCCAATACTCATCTGGATGCTTTGTTTCCTGCGCCAGTTTAGGAGTTCTATAAAAAAATCA
GATTACCTTGCACTCCGTCTGGGTTTCATCACTGAGCACAGATTGCCACTGTCATATAATTTTCATCAATATATGGTTCG
AAGCATGGAAGATGAATTTCATGGCATTCTAGGCATAAGCTGGCCACTTTGGATTTATGCTATAATCTGCATCTTTGTGA
ACATCCATGGTCTGAATATATACTTCTGGCTGTCATTTATCCCTGCAATGCTTGTGATGTTGATAGGCACAAAACTTCAA
CATGTAGTATCCACTTTAGCACTTGAAATTATGGAACAACAGGGTCCACTTGCTAGGACACAAGTAAAGCCACGTGATGA
TCTCTTCTGGTTTAAAAAGCCAGAAATACTCCTATGGCTGATTCAATTTGTGATATTTCAGAATGCCTTTGAAATGGCAA
CATTTATTTGGTCATTGTGGGGACTAGAGCAAAAATCATGCTTTATGAAGAACCATTACATGATCATCATTCGTTTGGCA
TCGGGTGTTCTTGTTCAGTTCTGGTGTAGCTACATGACAGTGCCCCTGAATGTGATAGTTTCACAGATGGGATCGCGGTG
TAAGAAGGCACTAGTAACAGAGAGTGTTAGAGATTCATTGCACAGTTGGTGTAAAAGAGTAAAACAGAAATCGAAACATG
ATCACTCGCATCATACTGCAAGATCAGTCTGTTCCCTTGAATCAACAATTGATGAGAGGGATGAGATAACAGTAGTATCT
GGTACTCTAACTAGAAGCTCTTCCTTAGACTTAGAGTCTTTGAATCAGATAACAGTAACTTCAGTAGACCAACTCAAATT
TGAAGCTTCCAACAGCTCCAGAGACCAAGATTCAATGAAAGTGGCCGAGTATTTGTCCAAATCTGTACATATTGACTCGA
CACAACCGCCGGATAATGGCGAGGACCATGTTGGTGGCAGTGAAGACGTAGCAAAATCTGAGACTCTTTCTGATTTGTTT
CAGAAAACATGA

>ArMLO6 ARAIP.HRR0E
ATGATGGAACCAATCGGAGAAGAAAGATCCTTGTCAGAAACACCAACTTGGGCTGTGGCAACTGTTGTCACTATCCTTGT
TTCTCTTAGTTTCTTGTTTCATGGCACATTAAAGAAGTTTCTTAAGTGGATGGCCAGAACCAAGAGAAAATCTCTGCTTT
CTGCTCTGGAAAAGATCCAAGAAGAATTAATGCTGTTTGGGCTGCTGTCATTGATCATGGGTCATTGGATTATCTTTGTG
GCGAAGATTTGTGTCAAATCATCAGCATTAAGCAGCAGGTTCTTCCCTTGTGCAGTCAAAGACATTTCTGGAACAGCAGT
GCAACACAATATTTTCTGGCATACTTCACACTATAATAACAGTGATTCCAAAGAGAAACTCCACATTGGCCTCCGAAATT
ATTGTCCTGAGGGACATGAATCATTGGCTTCTTATGAGAGCCTTGAGCAGCTTCATCGCTTTATGTTTGTTCTTGCAATC
ACACATGTTACATATAGCTTTGTTGCTGTTGCCTTGGCCATGATCAAGATATATAGCTGGAGAACATGGGAAAATGAAGC
AAAGACTATGGCTTTACAACAAAGCTTGCAAGGTACTCCACAAACTACATCAAGCATAAGATTGAATCGGCTTACAACGT

TCGTCTTTCACCAAACATCCCATCCCTGGAGTAATCACAAAGTTCTTGTTTGGCTGCTATGCTTCAGCCGCCAATTCTGG
AGTTCTATCAACTGTGCTGATTACATGGCGTTGCGCTTAGGCTTCATTACTAATCATGAGCTTCCCCTGACATATGATTT
CCACAACTATATGCTGCGAAGCATGGATGATGAATTTCGTGACATTGTTGGTGTTAGTGTGCTTCTCTGGATATATGCCA
TATGCTGCATATTTCTAAATTTTCATGGAAGCAACTTTTACTTTTGGCTTTCCTTTGTTCCAGCAATTTTGATACTCATA
ATTGGTACTAAATTGCACCGAGTGGTGGTAAAGTTGGCTACTGAAATAGTAGATCGTTGCCCTTATGTTAAGCCTCATCA
GTTTAATTTGAGAGATGAACTCTTTTGGTTTGGAAAGCCAAGGTTTTTGTTGCGCTTAATACACTTGATATCCTTCTTGA
ATGCGTTTGAGATGGCATCTTTCTTATGGTCCTTGTGGGAAATCAAAGAACCTTCTTGTTTCATGAAAAACAAAACCTTT
ATTGCAATCCGCTTATCATTCGGGGTAGTCTCACAAGTATGGTGCAGCTTCATAACATTTCCTCTATATGTTATCATCAC
ACAGATGGGGTCAAGGTTCAAGAAGTCAGTGGTGTCAGAGAATGTGAGAGAATCACTGTCTAAATGGCAGAGAAGAGTGA
AGCACAAACAGAGTTCCCCTCAAGCACTGGTTTCTGAAACATCTCCATCATCGGATTCTGGTATCAACAATGCAATGGAC
AAGTTAATCAGTGGGGAAGGAAGCTCCTCTGGAACTAAACATGATTATTGTTACTCTCATCATCATTTTGATTATGTCTC
TTCCAGAACACAAAGAAATGAACCATTTCTTAGTCATGATGATGATGATGATGATGATGATTCATATCCTCTTCCACTTTCTT
GA

>ArMLO7 ARADU.0YR51
ATGGCAGGAGGAGAGGGAGGAGCAGGAGAAAGATCTCTTCAGGAAACACCCACTTGGGCAGTGGCTGTGGTGTGCAGTGT
CTTTGTCATCCTCTCTGTCTTAATTGAACATGGAATCCATTCTCTTGGAAAGTGGTTTCAGAAAAAGCAAAAGAAGGCAA
TGAATGAAGCCTTGGAGAAGATCAAATCAGAATTAATGCTGTTAGGATTCTTATCGCTTCTACTTACATTTGGAACAAAT
TATATAAAGAAAATATGCATCCCTCACAGTATTGGGGACACCATGCTTCCATGCAAGAAGGTGGTTTCACATGGTGGTGA
TGGTAGGAGACACTTGCTTTCTTTTGAGACGGAGGACGACAATGATGTGTTGTCATGGCGTCGCATTCTAGCAACTTCTA
TCTCCGGCGACGACTATTGCTCAACCAAAGGTAAAGTGCCACTGATATCTGCTTCAGGGTTGCACCAGTTGCATATATTC
ATATTCGTCCTCGCTGTTTCTCACATTTTCTACAGCGTCATGACTATGGTCCTATCTCAAGCAAAAATGAAGAAATGGAA
GTCTTGGGAAGCAGAGACATCCTCCTTGGAGTATCAATTTTCAAATGATCCTGCAAGATTCAGGTTAGCACATCAAACAT
CATTTGTTAAACAACATTCAGGGTGGTCTAGGATGCCCGGAATTCGATGGATTGTGGCGTTCTTCAGGCAATTCTTTGCA
TCTGTGACCAAAGTTGATTACATGACTATGCGACATGGATTTATAAATGCACATCTTGGTCCCAATAGCAAATTCAACTT
CCATAAGTATATCAAAAGATCTATGGAGGACGATTTTAAAGTGGTTGTTGGTATCAGTATGCCACTTTGGGTTTCTGCTA
TCATCTTTCTGCTTTTAAACGTTTATAAATGGAACACGCTCACCTGGCTCTCATTCATCCCATTAGTGATACTTTTATTA
GTTGGTACGAAGCTTGAGCTAATAATAATGGAAATGGCGCAAGAAATCCAAGATAGAACCACAATTGTAAGAGGGGTGCC
AGTGGTGGAGCCAAATAACAAGTATTTCTGGTTCAATCGCCCCCAGTGGATCCTATTCTTGATACACTTCACCTTATTCC
AGAATGCCTTTCAAATAGCCCTTTTCCTCTGGACAGTGTATGAGTTTAAGATCAAGTCTTGCTTCCATGAAAACTTGAAA
CTAATACTGATAAGGGTCATCCTTGGGGTATTCTTACAATTCATATGCAGTTATATTACATTCCCCCTCTATGCCCTAAT
AACCCAGATGGGATCACACATGAAGAAAGCAATATTTGAGGAGCAAACAGCAAAGGCCATTAAGAAATGGCAAAAGACAG
CAAAGGACAGAACAAAGTTAAGGAAAGCAGGGATGGATAATGTTAGTAACTCAGGTTACATGAGTGGAGAAACAACACCA
AGCCAAGGAACATCGCCAATTCATTTGCTTCACAAGTACAAGCCTAGTAGCGACCACACAGATACCGAGAGTGTTCTCTA
TTCTCCACGTTCATACCCTTCTGATACTGAGTTATCTGAGACTGAATTAGCATATACTCATCATCATCGTATTCAACTCA
ATGAGATTACAACATCACATGGACATGGACCTCCAAATAACAGACAGGAAACTCATAATGTTGATTTTTCTTTTGACAAG
CCTTAA

>ArMLO8 ARAIP.L2HMX
ATGGCGGTAAGAGGAGAGGAAAGGGCGCTAGATCACACCCCTACATGGGCTGTCGCTGCCGTCTGCGCCTCTATCGTCCT
CATTTCTATAGTGCTCGAGAAAATTATACATAAATTCGAAAAAATGTTTGAACGTAAAAAGAAGATGGCTCTGTTACAAG
CACTCGCAAAGATTAAAGGCGAGCTTATGGTTTTGGGATTTATTTCGTTAATATTAACGTTTGGACAAACCTACATTTCC
AAATGGTGTATTCCCGAGAAGTATGCAAATACCATGCTACCTTGTGTTAGAAGCACGAAGGAGGAAGGCGAAGGCGGCGG
CGGCGGCCACAACCAAGCTACTCCAGCGGGTGCGGGTGTTGTGGAAGAGAAGGGTGGTGCTAAAGAGGCTGCTGCTGAAG
GTGAACCGCCCCATCGCAGGCTTTTGACATACGAACGTAGGTTTCTGGCTGCAGCTAGTGAAGGATGTAAACCGGGGATG
GAGCCTCTTATATCTGTGCATGGAATGCATCAATTGCACATTTTCATATTCTTCTTGGCTGTCTTCCATGTCGTATACAG
TGCCATAACAATGACTCTTGGAAGAGCAAAGATTCGTGGGTGGAAGGCATGGGAACAGGACCATATTGTGGATCAAGAAG
CCTTGAATGATCCTAGAAGATTCAGACTTACTCACGAGACATCATTTGTGAGGGATCACAACAGTATTTGGACTAAAACT
CCAGTTTCCTTCTATTTTGTATGCTTCTTTCGACAGTTTTTCAGATCTGTTCGCAAGGCCGACTATTTGACCATGCGACA
TGGATTTGTTACTGTTCATTTAGCACCTGGAAGTAAGTTTGATTTTCAAAAATATATTAAGAGGTCATTAGAAGACGACT
TTAAGGTAGTTGTGGGAATCAGTCCAATACTTTGGGCATCAGTGGTGTTTTTCTTGCTTGTAAATGTTCATGGATGGCAT
GCTTCCTTTGGGGTGTCTTTTCTTCCACTAATGGTTATTCTAGCTGTTGGGACAAAGCTTCAGGCAATTATAACCCGAAT
GGCACTTGACATAAAAGAAAGGCATGCTGTAGTGCAGGGGATCCCTCTTGTTCAAGTCTCTGACAAGTACTTTTGGTTTG
AGTGGCCTGAGTTAGTTCTTTATCTCATCCATTTTGTTCTCTTTCAGAATGCATTTGAGCTGACATATTTCTGGTGGACA
TGGTATGAATTTGGTTGGGCATCTTGCTTTTATGAAGATGAAATACTTATGATTAGTAGAGTTGCACTTGGGATAGGAGC
TCAAATAATCACCAGCTATGTCACTCTTCCACTGTATGCACTTGTTACACAGATGGGATCAACAATGAAGAAGTCAATAT
TTGATGAACAAACATCGAAGGCTCTAAAGAAGTGGCACCAGAATGCTCTGAAGAAGATAGCATCCAGGGGACGCCATGCC
ACTTTGGGAGGCACCACTATGTCAAAATCGCCGGACGGTTCACCCAGCAAAATGTCAACTAGATCCCCAAACGCCGGGCA
GTCAGCCACCATATTGTCGAGCGTAGATAACACTATCCCCGCCTATGACAACCGTGACCTTCTAACTGGACCATGA

>ArMLO9 ARAIP.CFV17
ATGAACGTAGAGAGTAGTATTATGGGTGGTGGAAGAAGCTTAGAGGAAACGCCTACTTGGGCTGTGGCTCTTGTTTGCTT
TGTCTTGCTCTTAATTTCTATTTTCATGGACCACATCTTCCACCTCATAGCAAAATGGTTGAGGAAGAAGCGCAAGAAAG
CTCTCTACGAGTCACTTGCAAAGATCAAGTCTGAGCTTATGTTAATGGGGTTCATATCGCTGCTGCTAACGGTGTTACAA

GGTCCAATATCGAGGATATGTGTACCGGAAAAGCTTGCTGCCACGTGGCACCCCTGTAATCATCAACAAGAGCCAGAGGA
CCATCATGTATTTTTACTAGCCCCTGGATCATCTACTCATCAATGTGCAGCTCATCAGGGTAAAGTGGCGCTTGTATCTG
CTCAAAGCATTCATCAACTGCACATATTTATCTTCGTGCTCGCTCTTTTTCATGTTCTTTACTGCATATTTACTCTCGCT
TTAGGCACTGCAAAGATGAGAAGGTGGAAACAATGGGAAGAGGAAACAAAGAAACCTGAGTATCAATTTTCACATGATCC
TGAAAAGTTCAGACTTACGAGGCAGACTTCCTTTGGGAGGAGACATTTGAGTGTGTGGACCACAAATCCTATTCTCGTTT
GGATTGTTTGTTTCTTCAGGCAGTTTGTATGCTCAGTACCTAAAGTGGATTACTTGACCTTGAGACATGGGTTTGTCATG
GCACACTTGGCACCTCAAAGTCAGCAGAAGTTTGATTTTATGCAATACATTGAAAGAACATTGGAAGAGGACTTCAAAGT
TGTTGTAGAAATCAGTCCTCCAATCTGGTTCATCACAGTGTTGTTTCTCCTGTTTCACACGCATGGGTGGTACTCTTATC
TGTGGATGCCTTTTGTTCCTTTGATTATAATTGTAGTAGTTGGAAGCAAGTTGCAAGTGATAATAACAAAGATGGGTCTT
AGAATTGGAGAAAGAGGAGACATAGTAAAAGGCACACCGGTGGTGCAGCCTGGTGATAACCTCTTCTGGTTTAAGAAGCC
TCAACTTATGCTCTACCTCATTAATTTTGTACTCTTTCAGAACGCTTTTCAGCTTGCTTTCTTTTCTTGGACTGCGCTTC
AATTTGGAATGAAATCATGTTTCCAATCGAAAACAGAGGATGTGGTTATCAAAATCTCAATGGGGATATTGGTTCAAATA
CTTTGCAGCTACGTGACTCTTCCACTCTATGCTCTGGTGACACAGATGGGCTCAAATATGAAGCCCACCATATTCAACGA
AAGAGTAGCATCTGCTTTAAGGAATTGGCACCAAAGCGCCAAGAGAAACATAAAAAAGAAGAACCGTGGTGGATCCTCCT
TCTCTCAAACCACAACTCCATCTCCAAACCACTCTTACGACGCTGATTCTCTTTCGTCCTCCAACGGGGCCAGGGCCCAC
TCTTGTTTTTCTTCTTCGTCTTCCTTTTCCAGTTCATCTCATCACCACGAAATTGAAATGGCCCATCTGGCCCATCACCA
ACACCAGAATCTCAAAGAGCCCAATTCCGTACATGTTGCTTCAGATCTACCACTGCCCCAACATGAAATTCAGATTGAAA
CCAGCAAGTAAT

>ArMLO10 ARADU.WJ2TG
ATGAGTGCCGGAGGTGCAGAAGAGGAGGCGAACCTAGAGTTCACTCCGACGTGGGTTGTGGCCGCCGTTTGCACTGTAAT
CGTGGCCATTTCCCTCGCCGCCGAGCGTTTCCTCCATTACGGCGGCAAGTTCCTCAAAAACAAGAACCAGAAGCCCCTAT
ACGAGGCTCTACAGAAAATCAAAGAAGAGCTGATGCTGCTGGGTTTCATTTCTCTGCTTCTCACGGTATCGCAAAACGGC
ATCACTAAAATCTGCGTTCCTCCGAGCGTGACTCGCTACATGCTCCCCTGCAGCCTCGATGAAAAGGAGAAGGTCACCGT
GGAGCATTTACTGGGACAAGACCGTTTCCATTTCGAACGTTTCTTCTCTTTCCCCAGGCGCCTTCTTGCCGAGACTCGAG
GGACAGAGACAGAGACACATGCACTCGCTAAGACTGAATTCTGCGCTCGCAAGAACAAGGTACCTTTATTATCTGTGGAA
GCGCTGCATCACCTGCACATCTTCATTTTTGTACTGGCCGTCGTTCATGTCACCTTCTCCGTTCTCACTGTTATTTTTGG
AGGAGCAAGAATTCGCCAATGGAAGCACTGGGAAGATTCCATTGCGAAGCAGAACTATGAAACAGACCAAGTTTTGAAAC
GAAAGGTGGTCGATGTTCAGCAGCATGATTTTATCAAAGGTCGCTTTTCGGGCTTTGGCAAAGATTCTGCCATAGTGGGT
TGGTTGAAATCCTTTTTTAAGCAATTTTATGGATCTGTTACAAAGTCAGATTATGTCACACTACGGCATGGTTTCATTAT
GACCCACTGCAAGGCGAATCCAAAGTTTAATTTTCACAAGTACATGATTCGGGCCCTTGAAGATGATTTCAAGCAGGTTG
TTGGCATAAGTTGGTATCTTTGGGTTTTTGTGGTCATATTCTTGTTGCTCAATATCAATGGTTGGCATACATATTTCTGG
ATAGCTTTTATTCCTTTCATTCTTCTACTTGCCGTTGGCACTAAGCTGGAGCATGTGATAACGCAACTAGCTCATGAAGT
AGCTGAAAAGCATGCTGCCATAGAAGGTGAATTAGTTGTACAACCATCAGATGATCACTTTTGGTTTCATCGCCCCCACT
TTGTCCTCTTCTTGATCCACTTTATCCTTTTCCAAAATGCTTTTGAGATAGCATTTTTCTTTTGGATATGGGTTACATAT
GGATTTGACTCTTGTATAATGGGACAAGTTCGTTACATAGTTCCAAGGCTCATTATCGGAGTATTTATCCAGGTACTATG
TAGCTACAGCACCCTACCACTGTATGCAATTGTTACACAGATGGGAACCCATTTTAAGGCAGCAATATTTCGTGAGCATG
TGCAAGTGAGGCTTGCTGGTTGGGCACAAGAGGCAAAGAAGAAGAAAGGGGTAAAAGCTGATAGCCAGCCTGGTGAAGGA
AGTTCTCATGCTGTTGCAGGAATTCAGCTTGGATCAGTTTTCCAGAAGAGAGCATCCGCTGCAGAAGACGGTGCTTCTGT
ACCCAAAGTCGAGGGTTCAGCTGAGTAG

>ArMLO11 ARAIP.SR8Z2
ATGTCGGATAGTGTGCAAGAAGCGCGGTCGCTTGCCTTGACTCCTACATGGTCTGTTGCCACTGTGTTGACCATCTTTGT
TGCTGTCTCTTTGCTTGTGGAGCGCTCCATTCACCGATTAAGCAATTGGTTGCGGAAAACTAATCGAAAACCATTGCTTG
CTGCTTTGGAGAAGATGAAAGAAGAGTTGATGTTGCTTGGATTCATATCACTCCTACTAACAGCTACCTCAAGAATTATA
GCCAATATTTGCCTTCCATCAAAGTTCTACAACAGTGCTTTTGCTCCATGCACAAGATCTGAGATCGATGAAGAAATGGA
AGATAATGGCTCCGAAGGACGTAAATTATTGATGGCTTTTGCTTATCCAAGCTTAACTCGGAGGATGCTGAATGGCATGA
ATAGAAGCTCCTGCAAAGAGGCAGGTTATGAACCATTTGTGTCATATGAAGGTCTAGAGCAATTGCACCGGTTTATTTTT
GTCATGGCAATAACACATATATCTTACAGTTGCCTGACGATGTTGTTAGCAATTGTGAAGATTCATAGTTGGAGAGTGTG
GGAGGATGAGGCTCATATGGATCAGCATGGATCTTTGGCAGTAATCACGAGAGAGTTGACAATGCGAAGACAATCGTCCT
TTGTCAAGGTTCATACATCAAATCCAATGGCCAGAAATAGTTTCCTCGTTTGGGTGACATGTTTTTTCCGACAATTCGGC
CATTCGGTGGTTCGTGCTGACTACCTTACACTTCGTAAAGGATTTATCTTGAATCACAACCTCACATTAAAATATGATTT
TCACAGCTATATGGTTCGATCTATGGAAGAGGAATTCCAAAGGATAGTTGGTGTGAGTGGCTTACTCTGGGGATTTGTTG
TTGCTTTCATGCTTTTCAACATTAAAGGATCAAATCTCTATTTCTGGATAGCTATCATTCCTCTTGTTCTTTTGGTGGGA
ACAAAGCTCCAGCATGTTATTGCAACGTTGGCATTGGAGAATGCCGGAATCACTGGTTACTTCTCGGGATCAAAGTTGAG
GCCTCGCGACGAACTTTTCTGGTTCAATAAGCCTGAACTATTGCTATCCTTGATCCACTTCATTCTCTTCCAAAATGCGT
TTGAATTGGCTTCGTTCTTTTGGTTCTGGTGGCAGTTTGGTTATAATTCCTGCTTTATCAGGAATCACATGCTTGTGTAC
TTAAGGCTAATCTTAGGGTTTGCAGGACAGTTTCTCTGCAGTTACAGTACCTTGCCACTATATGCGCTCGTTACACAGAT
GGGAACAAACTATAAGGCTGCATTGATTCCACAGAGGATAAGGGAGACAATCCATGGATGGGGCAAGGCAGCTAGGAGAA
AGAGAAGGCATGGTATGTTCGCCGATGATTCCACCATTCACACGGACACGAGCACTGTACTATCTCTCGAAGAAGACGAT
AGAATGGTTGATATCCCTGAAACTGGTACTGACCATGTCACTGGAACTGAAGTAGAATTACAACCCGTAGCAGCTGCTAC
GGCTACTCCTTCGCCGCCAGCTGCAAATGAAGCTTCAAGTAGGGCTGCCACACCACTCCTTCGACCATCGGCTTCAATAT
CTTCGTCGGGACCATTCAGTTTGAGAGCAGAATCTATTCCAAGATGTTCTTCTATGCCAATGCCAAGTGGAAGGTAA

>ArMLO12 ARAIP.WMV74
ATGAGTATTAGAGGTGTTGATGAGGAACAAGACATAGAATTAGAGTTCACTCCGACGTGGGTGGTTGCCGTTGTCTGCTC
CGTCATCGTTGGCGTCTCCTTCGCCGTCGAGCGCTTCCTTCATTATGCCGGAAAGCGTCTTAAGAAGAAGAATCAGACCA
CACTCTATGAAGCTCTGCTCAAAATCAAAGAAGAGTTGATGGTGTTGGGTTTTATTTCTCTGTTTCTATCGGTATCACAA
CGTGGGATAACAAAAATGTGTGTCCCAAAAAGTTGGAGTCACCACATGCTTCCATGTAGTCTTAAAGAAAAAGAAGAGTT
AGAAGCTGAAACAAATATTACTTCATCACATTTTCAGAATTCTCTTTCTTTCTCTTCCAATAATCTTAGACACCTTCTTG
CTCTTGCTCATGCCCAAGTAGCCGCAGAGGCTCAGCTTCAATACTGTGCTCTCAAGAACAAGGTACCTTTATTATCCAAC
GAAGCAGTGCATCATCTTCATATCTTCATATTTGTCCTAGCTATCGCTCATGTAACGTTTTGTGTTCTCACAGTTGCCAT
TGGATTATTAAGGATACGTCAGTGGAAGCAATGGGAAGACTCTGTTGCAACTCAAAACAATGAGACACACCGAGATTTGG
AGTCAACAGTTACTCTTGTTCACCAACATGCGTTTATCAGAGATCATTTTATAGGGATAGGCAAAAATTCGACCCTACTG
GGTTGGGTGAAATCTTTCTTCAAGCAATTTTATGGATCTGTGACAAAATTAGATTATGTGACATTAAGGCTCGGTTTTAT
TATGACCCACTGCAGGCGAAATCCAAAGTTTAATTTTCACAAGTACATGATTCGTGCCGTTGAAGATGATTTCAAGAAAG
TTGTTGGTATAAGTTGGTATCTTTGGATCTTTGTGGTCATCTTCATGTTGATTAATATTAACGGTTGGCATACATATTTC
TGGATTGCTTTCATTCCCGTCATACTTCTACTTGCTGTTGGGACAAAGCTAGAGCATGTGATAATGGAATTAGCAAATGA
AGTAGCTGAGAGGCATGTAGCCATTGAAGGCGAATTAGTTGTTCAACCATCAGATCATCATTTCTGGTTCAACCGTCCTC
GCATTATTCTCTTCTTGATCCACTTTACCCTTTTCCAAAATGCTTTTGAGATTTCTTATTTCTTCTGGATCTTGGTAACT
TATGGCATCCATTCCTGTATAATGGGACCAATTGGTTACATTATCCCACGGCTCATTATTGGGTTATTTATTCAGTTACT
TTGTACTTACAGCACACTACCACTCTATGCAATCGTTACACAGATGGGAACTCATTTTAAGAAGAACATATTTGAGGAAC
AATTGGAAAGACCTATTGTTTATTATGGGGGAGAAAAGGCAAAGAATAATGGAGCAAAAGCTGATGAAAGCCATGGTGAG
CATGAAAGAAGGGCATCTTCACCTGAGGCCAATACCAATGTCCCCAAAGAAGACAAGTCTAATTGA

>ArMLO13 ARADU.SSV2N_48N80
ATGGCTGGTGGAGAAAAAGAGCGAACGTTGGAGGAAACGCCAACCTGGGCTGTTGCAATTGTGTGCTTTGTTTTGCTTGC
TATTTCAATCATCATAGAACATATTATTCATGCTATTGGAAAGTGGCTCAAAAGAAAAAACAAAACTGCTCTTTATGAAG
CATTAGAGAAGGTTAAAGGAGAGCTTATCTTGTTGGGATTCATATCTCTACTCCTCACGGTGTTCCAAAATCCAATTTCT
AGAATATGCATATCAAAGAATGTTGCTTCAACGTGGCATCCATGTGCTGTCCCAAAGAAAAGTAAATCTGGTTACGAAGA
TTCTGATGATGATGATGGTGATGATGGTAATCGTCGAAAACTCTTAGAGTATTTAGGCCACATACCTCGCCGTGTTCTTG
CTACAAAGGGATACGACAAGTGTGCAGAAAAGGCAAGATGTTTAAAAAATTATAAAATTTTTCATATACTACAATGTATT
ATAACACTAGCTTTGGGAAGAACCAAGATGAGACGATGGAAGAAATGGGAGGACGAAACAAAAACAATTGAATATCAATT
CTACAACGATCCTGAAAGGTTCAGGTTTGCAAGGGACACAACATTTGGACGAAGACACTTGAGTGCATGGAGTCGGACGT
CAGTTTCCTTATGGATTGTGAGCTTCTTTAGACAATTCTACGGATCAGTTACTAAAGCTGACTATGTAGCATTACGACAT
GGATTTATCATGGCACATTTGGCACCAGGAAGTGAAGCCAAATTTGATTTCCAGAAGTATATACATAGATCACTTGAAGA
GGATTTCAAAATCGTTGTAGGAATAAGTCCAATCATATGGATTTTTGCCGTGCTATTCTTGCTGACTCATACTCATGGCT
GGTACTCTTACTACTGGCTTCCGTTTATCCCATTAACTATAATCTTATTAGTGGGTGCTAAGCTTCAAACAATCATAACA
AAGATGGGACTAAGGATTCAAGAAAGAGGAGAAGTGGTGCAAGGTTCACCTTTGGTTGAGCCTGGAGATGAACTATTTTG
GTTCAATCGACCAGGCCTCCTTCTCTCTTTGGTTCATCTTGTTCTCTTTCAAAATGCATTTCAGCTTGCATTTTTGGCTT
GGAGCACCTATGAATTCTCGATACATTCTTGCTTTCACAAAACAACTGTAGATGTTGTTCTGAGAATTTCAATCGGAGTC
GTCATTCAAGTTCTTTGCAGCTATGTGACTTTGCCCCTTTATGCTCTAGTCACTCAGATGGGTTCAACTATGAAAACCAC
GGTTTTCAATGATAGAGTAGCAACAGCATTGAAGAACTGGCACCATAGTGCAAAGAAGCATGTCAAAGATAGTAAGCATG
GAGAGGGTAACAACAGTGTGACACCATTCTCAAGTAGGCCAACAACACCAACTCACGGTCTCTCTCCGGTTCATCTTTTG
TATAGGCACACTGTGGGTCACAGTGATAGTGTCCAAACCTCCCCAAGGATATCCAACTATGAAAATGAACAATGGCATCT
TCAAGGTTCTCCTTCAACTACTAGCAACCATGACACTGATGAGAGGCAAGTTGTAGAGCAACCTTCCACACTAGAATCAC
CTGTCATCGGTGCTCAACATGAAATCAACATTAGTTTGTCGGAATTTTCTTTTGCAAATAGGCAAAATAGTAGAGATTAG

>ArMLO14 ARAIP.MQE1N
ATGTCGGATAGTGTGCAAGAAGCGCGGTCGCTTGCCTTGACTCCTACATGGTCTGTTCCCACTGTGTTGACCATCTTTGT
TGCTGTCTCTTTGCTTGTGGAGCGCTCCATTCACCAATTAAGCAATCTGCGGAAAACTAATCGAAAACCATTGCTTGCTG
CTTTGGAGAAGATGAAAGAAGAGTTGATGTTGCTTGGATTCATATCACTCCTACTAACAGCTACCTCAAGAATTATAGCC
AATATTTGCCTTCCATCAAAGTTCTACAACAGTGCTTTTGCTCCATGCACAAGATCTGAGATTGATGAAATGGAAGATAA
TGGCTCCGAAGGACGTAAATTATTGATGGCTTTTGCTTATCCAAGCTTAACTCGGAGGATGCTGAATGGCATGAATAGAA
GCTCCTGCAAAGAGGCAGGTTATGAACCATTCGTGTCATATGAAGGTCTAGAGCAATTGCACCGGTTTATTTTTGTCATG
GCAATAACACATATATCTTACAGTTGCCTGACGATGTTGTTAGCAATTGTGAAGATTCATAGTTGGAGAGTGTGGGAGGA
TGAGGCTCATATGGATCAGCATGGATCTTTGGCAGTAATCACAAGAGAGTTGACAATGCGAAGACAATCGTCCTTTGTCA
AGGTTCATACATCAAATCCAATGGCCAGAAATAGTTTCCTCGTTTGGGTGACATGTTTTTTCCAACAATTCGGCCATTCG
GTGGTTCGTGCTGACTACCTTACACTTCATAATAGATTATCTTGGAATCACAACCTCACATTAAAATATGATTTTCACAG
CTATATGGTTAGATCTATGGAAGAGGAATTCCAAAGGATAGTTGGTGTGAGTGGCTTACTCTGGGGATTTGTTGTTGCTT
TCATGCTTTCCAACATTAAAGGATCAAATCTCTTTCTGGATAGCTATCATCCCTGTCACCTTGTTCTTTTGGTGGGAACA
AAGCTCCAGCATGTTATTGCAACATTGGCATTGGAGAATGCCGGAATCACTGGTTACTTCTCGGGATCAAAGTTGAGGCC
TCGCAACGAACTTTTCTGGTTCAATAAGCCTGAACTATTGCTATCCTTGATCCACTTCATTCTCTTCCAAAATGCGTTTA
AATTGGCTTCGTTCTTTTGGTTCTGGTGGCAGTTTGGTTATAATTCCTGCTTTATCAGGAATCACTTGCTTGTGTACTTA
AGGCTAATCTTAGGGTTTGCAGGACAGTTTCTCTGCAGTTACAGTACTTTGCCACTCTATGCGCTTCTTACACAGATGGG
AACAAACTATAAGGCTGCATTGATTCCAGAGAGGATAAGGGAGACAATCCATGGATGGGGCAAGGCAGCTAGGAGAAAGA
GAAGGCATGGTATGTTTGCCGATGATTCCACCATTCACACGGACACGAGCACTGTACTATCTCTCAAAGAAGACGATAGA

ATGGTCGATATCCCTGAAACTGGTACTGACCATGTCACTGGAACTGAAGTAGAATTACAACCGGTAGCAGCTGCTACGGC
TAGTTCTGAAGCTATTTGA

>CaMLO1 LOC101504273
ATGGCTGGAGCAGCTCTAGGAGAAAGAACATTGAAGGAAACACCAACATGGGCTGTGGCTGCCGTTTGTGCAGTGTTCAT
CATCATCTCTGTATTAATTGAATATGGAATTCATTCACTTGAAAAGTGGTTTCACAAAAAACACAAGAAGGCCATGAGTG
AAGCCTTGGAGAAAATAAAAGCAGAATTAATGCTATTAGGATTCATATCCTTGTTACTTACATTTGGTACAAAATATATT
GCAAAGATATGCATCCCAGTGAAGTTAGGTGATAGCATGCTTCCATGTAAGAAAGAGAGAGTGGAAGAAGATTCAAAAGA
TGATAGGAGGAGGTTACTTTCTTTTGATGATGATAATTTGGTATGGAGGAGAAGTTTGGCAGCAGCAGCTGGTGGTGATG
ACTATTGCTCAAATAAAAATGAGGTGCCTTTAATATCTCAATCAGGGGTGCACCAATTGCATATATTTATATTCGTGCTG
GCAGTTTTTCACATTTTCTACAGCGTCATGACTATGGTACTAGCCCGAGCAAAAATGCAGAAATGGAAAGCTTGGGAAAT
GGAAACTTCCTCTGTGGAGTACCAATTTACACATGATCCTGCAAGGTTCAGATTTGCGCATCAAACTACATTTGTGAAGC
GTCACTCAGGCTGGTCTAGGAAGCCAGGAATACGATGGATAGTGGCATTCTTCAGACAATTCTTTGCGTCTGTCAGTAAG
GTGGATTACATGACTATGCGTCATGGATTTATTAATGCACATTTTGCTCCAGATAGTAAATTTGACTTCCATAAATACAT
CAAAAGATCAATGGAGGACGATTTTAAAGTGGTTGTTGGTATAAGTATTCCATTATGGGCATTTGCGATTATCTTTCTGC
TTATGAATGTTTACAGTTGGTACACACTCACTTGGTTGTCATTGGCGCCACTAGTGATACTTCTATTAGTGGGGACTAAG
CTTGAACTCATAATAATGGAAATGGCCCAAGAAATCCAAGATCGAACTACAATTGTAAGAGGTGTACCCGTTGTAGAGCC
CAACAACAAGTATTTTTGGTTTAATCGACCCCATTGGATCATCTTCTTGATTCATTTTACCTTATTCCAGAATGCTTTCC
AAATAGCCTATTTCTTGTGGACGTGGTATGAGTTTACGATCACATCTTGCTTCCATGAAAACTTGCCACTAATAGTCACA
AGGGTTGTCCTTGGGCTAGCCTTACAAGTCCTATGCAGTTACATAACGTTTCCTCTCTATGCACTAGTCACACAGATGGG
ATCACACATGAAGAGAGGAATATTTGAGGAGCAAACAACAAAAGCACTTAAGAAATGGCAAAAGAGTGCAAGAGATAAGA
GGAAGTTAAGGAAAGCAGGAGGAGGAGGATCAATAGATGTTCCTTCAGTGAGTGGAGAAACAACACCAAGTCAAGGAACA
TCACCTATGCACTTGCTTCACAAATTCAAACCTAGTAGCCACACAGACACAGATAGTGTTCTCTATTCTCCAAGATCATA
CCAATCTGATCAAACTGACTTTTCAGACACAGAGGGATCTACACACCGATTGAATCTGGATCAAATCATGTCACAACCAC
AACACAATCCACCAGGGAATCAATTAACTCATAATATTGATTTTTCCTTTGATAAGCCTTAA

>CaMLO2 LOC101500098
TTCAATTTCCTTAATACAATTCTTCAAAAATAAAACTCCATAAGGACTTTTTGTGTGTTTATGCTTCTACATTTCTTTTG
AGTATTGCAACAATTCAAAAAGAAAATATTAATTAATGGCTGCCCAAGTTTATGAACGAACTTTGGAAGAAACACCAACT
TGGGCCGTTGCAGTTGTGTGTTTTGTGTTGCTTGCTATTTCAATCTTAATTGAACATATTATTCATGTTATTGGAAAGTG
GCTAAAAAAGAAACACAAAAATGGTCTCTATGAAGCATTGGAAAAGGTCAAAGCAGAGCTTATGTTATTAGGATTCATAT
CATTGCTCCTAACTGTGTTCCAAGATCCTATTTCTAAAATTTGCATATCACAAAAAGTTGGAAACACTTGGCATCCTTGT
TCCAATCCAAATAAAAAGAGTAAATCTAATGAAACATCTGAAAATGAAAATAATGATAGAAGAAAACTTTTGGAATATTT
TGATCCTATACCAAGGCGAATTCTAGCTACAAAAGGATATGATAAGTGTGTTGATAAGGGTAAAGTTGCTTTAGTTTCTG
CATATGGAATTCACGAGCTCCATGTGTTCATTTTTGTGCTTGCAATATTTCATATCCTACAATGCATAATAACATTAGCA
TTGGGAAGAATCAAGATGAGGCGGTGGAAGAAATGGGAAGATGAGACAAGAAGTGTTGAATATCAATTCTATAATGACCC
TGAGAGGTTCAGGTTTGCAAGGGACACAACATTTGGACGAAGGCACTTGAGCATGTGGTCTCAATCAACCATTTCATTAT
GGATTGTTAGCTTCTTCAGACAATTTTTTGGATCTATTAATAAAGTTGATTATTTGGCACTACGACATGGATTTGTCATG
GCACATCTTGCCCCAGGAAATGATGCACAATTTGATTTCCAAAAGTATATAAGTAGATCACTTGAAAAGGATTTTAAAGT
TGTAGTAGGAATAAGTCCAACCATCTGGTTCTTCGCAGTGCTATTCCTTCTTACTAATACTCATGGGTGGTATTCTCATT
ATTGGCTTCCATTTATCCCATTAATTATAATTTTATTAGTGGGTGTTAAGCTACAAATGATCATAACAAAGATGGGACTA
AGGATTCAAGATAGTGGGGAAATAATCAAAGGTGCACCTTTGGTTGAACCAGGAGATCATCTTTTCTGGTTCAATCGCCC
CGATCTTCTTCTCTTTTTGATTCATCTTGTTCTCTTTCAGAATGCCTTTCAACTTGCATTTTTTTCTTGGAGTACTTATG
AATTCTCCGTAAACTCTTGCTTCCATAAAACAACCGCAGAAATTATCATTAGACTTACACTAGGGGTTGTAATACAAATT
CTATGTAGCTATTCAACTTTGCCTCTTTATGCTCTAGTCACTCAGATGGGTTCAACCATGAAACCAACAATTTTTCATGA
AAGAGTTGCAACAGCACTTAAAAGCTGGCACAACACAGCCAAAAAGCATGTGAAAGAAAGCAAACATTCAAACAACACGA
CACCATTCTCCAGCAGACCATCAACACCAACTCATGGCATGTCTCCTGTCCACTTACTTCATAGACACACTGTTTCCAGC
AGTGACAGTCTTCAAACTTCTAACTATGAAAATCAACAATGGGATAATGTTGAAGGATCAAATTCACCAACAATCAACCA
TACCGAAGCACATGAAATTCGGATACCAACCGAGTTGCCTGTAAGTAATAGACATGAAATTAGTATTAGTTCAAAAGATT
TTTCTTTCGAGAAGCGATATATTGTTAGCGATTAGAATTGTAGATATCGAAAACTAGTTCAATGTATAGCTTTAGTTCAA
TGTCGTAAGATATAACAGCTTGTTCGAAATCTGCTGCTACAATAGGCCACTATAACTGTACTTGACAACATTTGTATTAA
ATAATATATCGCAGAATAATAGCGATTAATTTGTTTGAATTTTACTATATTTTAGTGTTGTTGTAGACGCTATTTAATAA
AACTTTAAGAATGGAGGGAAATT

>CaMLO3 LOC101490933
AACAGTTGGCACGCACGCACACGTTTTAATTAATTAATAATAATGAATGGCGCCTCTGTTACTGATGATAATGTAATTTA
AAGAAGGGAGAAAGGTAGAGAGAGAGAGAGAGAGAGAGAGAGAGAGAATGAGTGGTGGAGAAGAAGAAGAGGAGACAACA
CTTGAGTTCACTCCAACCTGGGTAGTGGCTGGCGTTTGCACTGTGATAGTTGCCATTTCCCTCGTCGCTGAGCGTTTGCT
TCACTATGGTGGAAAGTTTCTGAAACGCAAGGATCAAAAGCCACTCTACGAGGCTCTCCAGAAGATCAAAGAAGAGTTGA
TGCTTCTGGGTTTTATTTCACTTCTTCTGACGGTTTCACAAAACAGCCTCACCAAAATATGCGTTCCTCCCAGTGTATTG
CGCCACATGCTTCCTTGTACCCTGAAAGAGAAAGAGAAAGCGAATCAAGTATCACATTATTCTGCTTTCTCCTTTCCTGG
ACTTGCTAGGCGCATTCTCGCCAATGAAGAAGATGAACATCCTAAAACCCCTTTCTGTGCTCGCAAGAATAAGGTTCCTT
TATTATCTCTGGAAGCTTTGCATCACCTCCACATCTTTATTTTTGTACTCGCCGTCGTCCACGTCACCTTTTCAGTTCTC
ACTGTTGTTTTTGGAAGCGCAAGAATACGTCAGTGGAAGCACTGGGAAGATTCTATTGCAAAGCAGAACTATGATACTCA

CCAAGTTCTCAAACCAAAATTGACTCATGTTGGACAGCATGAATTTATCAAGGGTCGCTTTTCGGGTTTCGGCAAAGACT
CTACTTTAATCGGTTGGTTGCAATCCTTTTTCAAGCAGTTTTATGGTTCTGTGACAAAGTCAGATTATGTGACATTAAGA
CTTGGTTTCATTATGACCCACTGTAAAACAAACCAAAAGTTTAATTTTCACAAGTACATGATTCGTGCCCTTGAAGATGA
TTTTAGGCAAGTTGTTGGTATAAGTTGGTATCTTTGGCTCTTTGTGGTCATCTTCTTGTTGCTAAATATCAATGGTTGGC
ATACATATTTCTGGATTGCTTTTGTTCCTCTTATTCTTTTACTTGCTGTGGGAACTAAGCTGGGGCATGTAATAACCCAA
TTAGCTCATGAAGTGGCTGAAAAGCATGCAGCCATAGAAGGTGACTTGGTTGTACAACCATCGGATGAACACTTTTGGTT
TCATCGGCCCCGCGTTGTTCTCTTCTTGATTCACTTTATCCTTTTTCAAAATGCTTTTGAGATAGCATTTTTTTTCTGGA
TATGGGTTACATATGGATTTGACTCCTGTATAATGGGGCACATTCGGTACATTGTTCCAAGGCTCGTAATTGGGGTATTT
ATTCAGGTACTGTGTAGCTACAGCACCCTACCACTGTATGCACTTGTTACACAGATGGGAACTCACTTTAAGAGGGTAAT
ATTTAATGAGCATGTGCAAGACGGCCTTGTTGGTTGGGCACAAAAGGTAAAAAAGAAGAAAGTGGTAAAAGCTGATGGCC
AAGGAAGTTCTCATGAGGGTTCTAGTACAGGAGTTCAGCTTGGATCAGTTTTCCAGAAGAGGGCATCGGCACATGAGACC
AATAATGGCCAAGGAAGTTCTCATGAGGGTTCTAGTACAGGAATTCAGTTTGGGTCAGCTTTCCAGAAAAGGGCATCTGC
ATCAGAAGACAATACTCCTCCTATCACCAAAACTGATGGATCCAACTGAGTCTGGTTGTCCAGGTTATGGTAATAAGATG
AAGCCAATAGGTGGGAGCTGTGCTCTGTAGGCTTGTCTTTTTTGTGAAGTTGTATAACATATTGCAGTTTCTCTTTATCT
TATTTTCCATGCCAGCGTGGTGGTGAGGCATTACTGTAGGCATGGGATGAAAGATGTATATATAGGCTCAAGATTCTAAG
ATCTGCTAGCACACCTACTTAATAGTTACTGCAACATGTCTTCTGGTTTTTGCTGTGTGCTTCGCCTAATACTTTCCCAG
TCACCGTGACTCTACTCAGAATTGGAAAAGCGTTTTCAATTTTTTGTCTGATAAAGTACAAGACAAATTGGAGGGATGGG
ATTGGGAAGCTTCCTTCCTGTTTGTTTGCACTTTTGCTTCA

>CaMLO4 LOC101505951
ATGGAGAAAGAAAATGGAAATGGAAGATCCTTAGCTGAAACTCCAACTTGGGCTGTTGCAACTGTCGTTGCTCTTTTAGT
TTCTCTTAGTTTCTTATCTCATGGCACATTGAAAAAATTGTTTAAGTGGTTGGATAAAACTAAGAGAAAATCGCTTCTTT
CCGCTATGGAAAAGATGCAAGAAGAACTAATGCTTTTTGGGCTTCTGTCATTGCTGATGGGGCATTGGATTCTTTTTGTG
GCTAAGATTTGCGTTAAGTCATCAGTATTAAGCAGCAGATTTTTTCCATGTGCAATGGAGAAGAGTTTTGGAATTGTGAA
ACAAGTTGCATGGTCAAATTCAGAATATTCAAATCAAACAATTCTCAAAGAGCAAGCGCATCATGGCAGGCATAATTATT
GTCCTAAGGGTCATGAATCATTTGCTTCATATGAAAGCCTTGAGCAGCTTCATCGCTTTGTGTTTGTTCTAGGGATCACG
CATGTTTCCTATAGCTTTGTTTCTGTAGCCCTGGCCATGATCAAGATATACAGCTGGAGAAGATGGGAAAATGAATCAAA
GACTATGGCTGTACAAAGTTTACAAGATACTTCACAAACTACTTCAACTTCAAGCATAAGATTGAGGAGGCTTAGTACAT
TCATTTTTCACCACACATCCCATCCTTGGAGTCATCACAAAGTTCTTGTTTGGCTGATATGTTTCAGTCGCCAATTCTGG
AGTTCCATCAATCGAGCTGATTACATGGCATTGCGCTTAGGCTTCATTACTAATCATGACCTTCCTCTAACATACGATTT
CCACAACTACATGCTTCGAAGCATGGATGAGGAATTCTGTGACATTGTAGGTGTTAGTGTGGTTCTCTGGATATATGCCA
TATGCTGTATTTTTCTAAATTTTCATAGAAGCAACTTTTACTTTTGGCTTTCCTTTGTCCCAGCAATTTTGATCCTCATA
ATCGGTACTAAATTGCATCGAGTGGTTGTAAAGTTGGCGGTTGAAGTAAGAGATCATTGCCCATATATGAAGCCTCATCA
GTTTAACTTAAGAGATGAGCTCTTTTGGTTTGGAAAACCATGGATTCTCTTGAGGTTAATACAATTGATATCCTTCTTGA
ATGCATTTGAAATGTCAACATTTTTATGGTCCTTGTGGGAAATTAATGATCCTTCTTGCTTCATGAACAACCGAACATAC
GTTGCGATCCGATTGTCATTCGGGGTTGTCTCACAAATCTGGTGCAGCTTCATAACGTTTCCTCTCTATGTTATCATTAC
ACAGATGGGATCAAGATTAAAAAAATCAGTGATATCTGAAAATGTAAGAAAATCACTATCTAAATGGCATAGAAGAGTGA
AGGCTAAACGAAGTTCCTCTTATGCACTCCTTACTAAAACATCACCAACATCATTGGATTTTCTGCTGTATGGAATTGAC
AATTCAAATTCAAATAAATCTGCCTTCATCAGTGAGACTGAGGAAGGAAGTTCCTCAGGAACAAAAGACAATAGTCTTTC
TAACCTTGATGATGTGTCAATGACTCAAAAGCTATGA

>CaMlo5 LOC101503406
TCCAGAGTCTAAGCGTCACTTGTTCCTCTCCATTCATTTGCAGTGGTTTGTAGGTTAGAGTTAGAATAGAATGCAACATA
GGAAAAACAATTTGATTTACACTTGCATCTGTCACATGAATGCAATAACAACAACATATGGAAACAATTTGGTTCTCTTG
AACGAATGCACGAGGATGCCATCAAATTATTTGTATCCTCAATATTTTTTTGATTCATACAAAACTCGTATTCTTAACAA
ATTCTCATAATCATCGTTCTAATTTGAATATTAACTATTTCATTATAATTATATCATAATAATAATCAATAATAATGTCG
GGAGGAGGAGGAGGCGGTGACGAAAGGCAGCTAGATGTAACTCCTACATGGGCTGTCGCTGCGGTTTGTGCAGTTATTGT
TATTATTTCCATATCGTTAGAAAAACTCATACATAAAATCGCAAAGGTGTTTGAAGAGAAAAAGAAACATGCTTTGCTAG
AAGCTCTTGAAAAGATTAAAGCCGAGCTTATGGTTTTGGGATTTATTTCTTTGCTTTTAACATTTGGGCAAAACTACATA
TCAAAAGTTTGTATTCCAACGAGGTATTCAAATACTATGTTGCCATGTCTTCCCTTGGATCAACGACAAGCTGGTCATCC
AAGTGAGCCGGCACAAACGGGACATGAGCCTCCAACAACGGGACATGAGCCTCCAACAACGGGACATGAGCCTCCAACAA
CGGAACATGAGCCTCCAACAACAGAACATGAGCCTCCAACAACAGAACATGAGCCTGCACCAACAGAACATGAGCCTCCA
CCAACGGCACATGAGGCACCGGCCGAGGAGGCTGAGCCGTCTAAAGGCGAACATCATCGCAGACTCTTATCATATGAACG
TAGGGTTCTCTCCGGTGGTGGTGGAGGACACGGATGTAAATTGGGGTACGTGCCTCTTATAGCAGTGAATGGGTTACATC
AATTACATATTTTCATTTTCTTCTTGGCAGTCTTCCATGTGATATACAGTGCCATTACAATGACACTTGGTAGAGCAAAG
ATTAAAGGATGGAAGGAATGGGAACAGGACCATGTCGTGGATGAAGATGCCTTGAATGATCCTAGAAGATTCAGACTTAC
TCATGAGACATCATTTGTGAGGGATCACAACAGTTTTTGGACTAAAACACCTGTTTCCTTCTATTTTGTATGTTTCTTTA
GACAATTTTTCAGATCGGTTCGAAGGGCTGATTACTTGACGATGCGACATGGATTTGTTTCTGTCCATTTAGCACCTGGA
AGTAAATTTGATTTTCAAAAATATATCAAAAGGTCATTAGAAGATGACTTTAAGGTAGTTGTAGGAATCAGTCCACTACT
TTGGGCATCAGTGGTGTTATTTTTGCTTTTAAATGTCCACGGTTGGCATGCTGCATTTTGGGTTTCTTTTCTTCCACTTG
TAGTGATTCTAGCTGTTGGAACAAAGCTTCAAGCAATTATAACTCGAATGGCACTTGACATACAAGAAAGACATGCAGTA
GTGCAGGGGATTCCTCTTGTACAAGTTTCTGACAAGAATTTTTGGTTTGAATGGCCTCAGTTAGTTCTTTATCTCCTCCA
TTATGTTCTCTTTCAGAATGCATTTGAGCTGACATTTTTCTGGTGGACATGGTATGAATTTGGTTGGGCATCTTGCTTTT
ACGAGGATGATAGTTTTATGATTGTGAGAGTTGCTCTTGGGTTAGGTGCTCAATTTGTTTGCAGCTATATAACACTTCCT

CTCTATGCATTAGTGACTCAGATGGGATCGACAATGAAGAGGTCAATATTTGATGAACAAACATCAAAGGCATTGAAAAA
TTGGCGTAACAAAGCAATGCATAAGAAACCATCAATCAAACGTCCAACTGAAACAAGAACATTAGGTAGTCCAATGGATG
ATGGGTCACCATTGGATTCTCCATTAACAAATATAAACACAAGTGCAGAAACAGAATTGCAGGAACATGGCACAGCTACC
ATACTCACTAGTGTGGATAATGACCACTATGATAACCGTGACCTTCTAACTGGACCCTGATTCATTCACACTTTATACAT
AAATACAATATGTTGAATCAATCAATCAAACTACAAATGCAGGGCTATGGTAATTCATTAAATGTAAATAATAACCCATT
TGTGCACCTTGGGTTCAATAGAGTTTGTCTACTAGTAGAATTAGCAAACTACCCTAAGCCATTGCATATATAATTTTGTA
GCACCACAGTTCAGATTAGTTGCTATATATATGCTTGTAAACTAAT

>CaMLO6 LOC101492588
ATGGCAGGAGGAAGAAGCTTAACTGAAACGCCTACTTGGGCTGTTGCCGTTGTTTGCTTTGTTATACTCTCTATTTCTAT
CATCATTGAACACATTTTCCACATCATAGGAAAGTGGTTGAAGAACAAGCACAAAAGTGCCTTGTATGAGTCACTTGAAA
AAATCAAATCAGAGCTTATGTTATTGGGGTTCATATCATTGCTCCTAACTATAGGAGAAGGTTTAATATCAAAAATATGT
ATATCAGAAAAGGTTGCAGCCACGTGGCACCCTTGTACCAATAATGCAGAGAGGAATTCGGATGATCACCAACCGATAGA
ACATGAAACCGGTGGCCGGAGATTACTAGCGGCGTTGCTTGCTTCTCAAAACAAAAACTCCCGCCGTATATTGGCTGCCG
GAGGAATCGACAAATGTGCCGCTCAGGGAAAAGTAGCATTTGTGTCATCAGAGGGAATTCATCAACTCCATATATTTATA
TTTGTGCTGGCTGTTTTTCATGTTCTTTACTGCATACTTACTCTGGCTCTAGGCAGAGCAAAGATGAGAAGGTGGAAACG
ATGGGAAGAGGAAACCAAGACACTTGAGTACCAATTTTCACATGATCCTGAAAGATTCAGATTTGCTAGTGAGACTTCAT
TTGGAAGAAGACACTTGAGTTTCTGGACAAAAAATCCTGTCCTCATTTGGATTGTTTGTTTCTTTAGGCAATTTGTAAGG
TCAGTTCCTGAAGTGGATTACTTGACCTTGAGGCATGGATTTATGATGGCACATTTGGCGCCCCAAAGTCACCAAAAATT
CGACTTTAGACATTATATCAAAAGATGTTTGGAAGAAGACTTCAAAGTTGTGGTAGAAATCAGTCCTCCAATCTGGTTCA
TCACAGTGTTGTTCCTCTTGTTCCACACTCATGGCTGGCACTCTTATCTGTGGCTACCATTTGTACCTTTGATTATTGTC
CTATTAGTTGGGACAAAGTTGCAAGTGATAATAACTCAAATGGGGCTTAGAATTCAAAAACAAGGAATGGTGGTAAAGGG
AGAGCCAGTGGTGCAACCTGGGGATGACCTATTCTGGTTTAGCAAACCTCGACTTATTCTCCACCTTATTAATTTTGTGC
TCTTTCAGAATGCCTTCCAGCTTGCTTTCTTTTCATGGACTGCGCTTCAATTTGGGTTGACGTCCTGTTTCAATTCGCGT
ACAGAGGATGTAGTCATTAGAATATCCATGGGGGTCTTTGTTCAAATCCTTTGCAGCTACGTCACTCTTCCTCTCTATGC
TCTTGTGACACAGATGGGTTCAACCATGAAGCCAGCCATATTCAACGAAAGAGTAGCCACAGCTCTAAGGAATTGGCACA
ACACCGCAAAGAAGCACATAAAGCAGAACCGTGGATCGGGGTTTCAAAGCCCAATGTCAACAAGATCCATAACTCCGGCC
CATTCTATGTCCCCGGCCCACATCCTTCGTCCTTACCACAGCGAAACGGACACACACCCCACCACATCTCCAACAAGACT
CAACTTTGAGACCTATAATCCCTACGAGGCCTATTCACCCTCACCCTCCAACAAGGTTGAGGCCCGTGCTACTTCTTCTA
GCTCAATTTATTTTCATGAAATGGAAATGGGTCACTTGGCTCATGATCATGAACAAGAGACCAACAAGCCCAATTGTGTT
TCTGTGGGCTCAGGCCTGACTCAACTTGAGATTGATGTCCAACACAGCGACGAATTTTCATTTTCTAAAATGGCCACAAA
TAACTTAAAATGA

>CaMLO7 LOC101501959
TTGCAGTTGTAACATTAAGTCATAAACACAAACAATAAAAGTAGTTAATGTTGTTGAAATTCAAATATTGACACACACAC
AACATGAGTGGTGGTGGAGAAGGAGGTGAAGAAGGACCAGATTTGCAATTTACTCCAACATGGGTTGTTGCTCTTGTTTG
CACTGTTATTGTTGCCGTTTCATTTGCGGTTGAACGTTCTCTTCATTATGTTGGCAAGTTTCTCAAGAAGAAAAACCAAA
AAGCACTCTTTCAAGCCTTGCAAAAAATCAAAGAAGAGTTGATGTTATTGGGTTTTATTTCTCTGTTTCTGACGGTAACA
CAGAATAGAATTACAAAAATATGTGTTCCTGAGAATTGGACTCATCATATGCTTCCTTGTAGTCTTGATGAAAAAGATGA
AGAATCCAAAAAATCCACTTCACATTTTCAGACTTTTTTCTCTTCCAATGATGTTTTCGGCACTACTAGGCGCCTTTTAG
GTGATGACCATCCATCAACAGAAGAAACACTTGGATATTGTGCTGCTAAGGGCAAGGTGCCTCTATTATCAGTGGAAGCA
CTGCATCATCTGCATATCTTTATTTTTGTCCTAGCTGTTGTTCATGTGACTTCATGTGTTTTGACTATAGTTTTTGGAGG
ATTAAATATTCGTCGGTGGAAGCACTGGGAAGACTCTATTACAGATGAAAATCATGAAACACAACATGCGCCAGATCGAA
CTGTGACTCATGTTCATCAACATGCTTTTATCAAGGATCATTTTACTGGTTTTGGCAAAGATTCTGCTATAATGGGATGG
GTGAGATCCTTTTTCAAGCAATTTTATGGATCTGTTACAAAGTTGGATTATGTGACATTAAGGCTTGGTTTTATTATGAC
TCACTGTAAGGCAAATCCAAAGTTTAATTTTCACAAATACATGAATCGTGCCCTTGAAGATGATTTCAAAAAAGTTGTTG
GTATTAGTTGGTATCTTTGGGTCTATGTGGTCCTCTTTTTGTTGCTTAATATCAATGGTTGGCACACATATTTCTGGATT
GCTTTTATTCCTCTCATTCTTCTACTTGCTGTTGGAACAAAGCTAGAACATGTAATAATTCAATTAGCTCATGAAGTAGC
TGAAAAACATACAGCCATACAAGGTGAATTAGTTGTTCAACCATCAGATGATCACTTCTGGTTTCATCGTCCACGCATCG
TCCTCTTCTTGATTCACTTGATCCTTTTCCAAAATTCTTTTGAGATAGCATTCTTTTTTTGGATACTGGTCACATATGGT
TTTGATTCATGTATAATGGGAAGAATTCGCTACATTATTCCAAGGCTCATTATTGGGGTATTTATTCAGCTGCTATGTAG
TTATAGTACCCTACCATTGTACGCACTTGTCACACAGATGGGAACTCATTTTAAGAGTGTTATATTTGATGAGCAAGTAC
AAGCAAGGCTTGTTGGTTGGGCACAAAAGGCAAAGAAGAAAGGATTAAGAGGTGATAATAACCAATCTAATCAAGAAAGT
GCTCATCATGGTTCAAGTGTTAATATTCAGTTAGGGTCAATATTTAGAAGAGGATCAGTCCCAGAAAACAATACTATTGT
TTCTAGAGATGAAGAGTCTGGATGAAAAAATAGTCATGTAACAAAAATGTTAATAGTTATTATATATTATAATATTTTTT
TTAA

>CaMLO8 LOC101508058
CTTCATTCAACTAATAATCAAAATAAAAACAAAACACTCTCAGTAGCTTCTTTTTTCCTTCTTGGATCCCATCTACCACT
TATTTCTGTGTTTTTTTTTTCAATGAAAGGTTTAAACTTTTTGCATTAGAACTAAAGGGTTGAACTGTGCTGTGTTGATG
TGAATAAGAAAAAATTAATGGAGGATGTTATGCATAGAGGTCGTTCTCTGGCTGAGACACCTACATATTCAGTTGCATCT
GTTGTTACTGTCATGGTCTTTGTCTGCTTCTTGGTTCAGCGTTCTATCTACCGCTTTGGAAAGTGGTTGAAGAAGACAAG
AAGGAAGGCACTTTTTGCTTCACTGGAGAAAATTAAGGAAGAGCTGATGCTGCTTGGACTTATCTCTTTGTTGTTGGCAC
AAAGTGCGAGGTGGATCTCTGAAATTTGTGTAAATTCATCGCTTTTCAGCAGTCGATTTTATACCTGTGCCGAACAACAC

CTTGGCACGATTGAGAATATCATGATTGAAAACTCTTCCTCTTTACCTGATGAAACTAATATTCCAAAGGGACTAAGTAG
TGTCGCACTTCATCAATGTGGAGAGGGCCATGAACCATTTGTTTCATATGAAGGTCTTGAGCAGCTGCACCGGTTTTTAT
TTGTTCTCGGGATTACTCATGTTCTGTATAGTTGTCTGGCTGTCGCTTTAGCGATGAGCAAGATCTATAGTTGGTGCAGA
TGGGAAAATCAGGCTGCAATGGCAACTGATGCAAATTTGCAAGGAAAGAAAAACAAGGTGATGAGGCGGCAAATAACCTT
TGTTTTCCATCGCACTTCTCATCCGTGGAGCAGGAGTCCAATACTAATTTGGATGCTATGTTTTCTGCGTCAGTTTAGGA
GTTCTATACAAAAATCAGATTACCTTGCGCTCCGTCTTGGTTTCATCACTGAACATAAATTGCCATTGTCATATAATTTT
CATAAATATATGGTTCGAAGCATGGAAGACGAATTTCATGCCATTCTAGGAATAAGCTGGCCACTTTGGATTTATACCAT
AGTTTGCATATTTGTGAACATACATGGTCTGAATATATACTTCTGGATCTCCTTTATCCCTGCAATTCTTGTCATGATAA
TAGGTACAAAACTTCAACATGTTGTATCTACTCTAGCACTTGAAATCAGAGAGCAGCAGGGTCCATCTGCTAGAACACAA
GTAAAGCCACGCGACGGTCTTTTTTGGTTCAAAAAACCAGATATATTGTTATGGCTAATACAGTTTGTCATATTTCAGAA
TGCCTTTGAAATGGCTTCATTTATTTGGACATTGTGGGGATTTAAAGAAAAATCATGCTTCATGAGACACCATTACATGA
TCATCATTCGGTTGACGTCCGGTGTTTTTGTTCATTTCTGGAGTAGCTATATGACAGTGCCACTTAATGTAATAGTTTCA
CAGATGGGATCGCGATGTAAGAAGGCGCTAGTGACAGAGAGTGTAAGAGAATCATTGCACAGTTGGTGTAAGAGAGTAAA
ACAGAAGTCTAAGAATGAGTCTATGCACTCACATACAGCTAGATCAGTATGTTCCTTAGAGTCAACAATCGATGAGAGGG
ACGAGATAACTGTGGTATCCGGCACATTATCTCGAACCACTTCATTAGACTTAGAGTCTTTGAATCAGATAACAGTTACT
TCAGTAGACCAGCTGAACTTTGTGACATCTAACAATCTCCATGATTCAACCAATTTGTCCGAATCTGTACATATTAATTC
GCCATACAATGATGGAGACCATGTTGGCAATGCAGAGGAGGCTAAAATTGAGAGCACTCTTTTTGAATTGTTTCAGAAAA
CATGATAAATTTTATGTTTCATATTTTAAGTTCTACTGCATTTCAAGCTGGTATCTATAGATTAGTATATATAGATATGA
AAATCATTCTCGAACCCTATGAAGTATAGTTGTATATTATATATAACACTGTT

>CaMLO9 LOC101495573
GTAATTTTTCTTTCTTTCTCATGTTAGCTACTGAGCAGGGGAGAAACATAATTAGCAACGGTTTAATTGAAATTAAGAAG
AACATGGCTGTTAAAGGAGAACAAAGATCTCTTGAAGATACACCAACATGGGCTGTCTCAGTTTGTAGTTTCTTTGTTCT
AATTGTATCTTTGATCATTGAAGCTGGACTCCACAAATTGACTGAGCATCTAAAGAAAAAGAAAAGAATATCCATGGGGA
AGGCTTTGGCTAAAACTAAAACAGAAATGATGAAATTCGGTTTCGTGTCACTTCTTCTGACAATATCGCAAGTGCCAATA
TCCAAAATCTGTATCAACAAGGATGTGGCGAATTCTTTCCTACCATGTAATGATTCTATGGAGTCGGAATATCAGTTCAT
CAATTATTGTGAAGCAAAGGGCAAGGTTAGTCTTCTTTCAAGTGATGGAATTCTGCAGCTAAATATCTTCATATCCTTTT
TGGCAGTGTTTCATATACTTTTTTGCACATTCACGATGTGCCTTGGAAAGGCCAAGATGAGAAGATGGAAAAAATGGGAG
GACGAGACTCAAACACTCGAATATCAGATAGATAATGATTCAAGAAGATTCCAGTATATAAGTCAAACATTGGGTGGCAA
GCGTCATTTGAAATTATGGAATAACTATTCTCCGTTGCTGTGGATGGTGTGTTTTATGCGGCAGTTCTATAATTCAGTCT
CAAAAGATGATTACTTTGCTCTACGAAATGGGTTCATTGTAGCAAATATTTCAATGGGAAGCAATTTCAATTTCAATTTT
AAGAAATTTCTTTCCCGTACTTTTGACGAAGATTTTGAGAAGGTCGTTGGAATCAGATTTTGGATATGGTTTTTCTCCAT
ACTTCTCATATTCCTTAGTGCACATGAGTTTTATAACTACTACTGGCTTCCATTTATTCCACTTGTGGTTGCTCTCCTTG
CGGGGACTAAACTGCAAATGATAATAACGAATATGCATCTTGATAGTTGCAAGCAAAGCCCAGTGATCAAAGGAACCATA
CTTGTAAGACCAAGTGATGAATATTTCTGGTTTCATCGACCCGAATGGCTTCTCCATATAATCAAATTAATCCTTATACA
AAATTCCTTTCAACTTGCATTTTTCACCTGGACTTGGTATGAATTTGGTCCAAGGTCTTGCTTCAATAGAGAAAAAGCAG
ACATTGCCATAAGGATTGCATTGGGTTTAGCTGTACAGCTACTATGTGGTTATGTTACTCTGCCCCTTTATACACTTGTC
ACTCAGATGGGGTCTAACATGAGGAAGGAAATTTTCACGGAGCGTGTTGTTGAAGGTCTAAAAAGTTGGCAAAAAAAGGC
TAAGAGATCACTTTCGAAAAACAACTCAACTTGTAGCAGACACACAGTTTCAGCTTCTTTACATTCAAAAGCAAGTGAGA
ATTCAGTAAAAGAGTCTTCAGAGAGTATGCACAATAAGGTTGATGATAATGAAAACATGCAATCAATAACAGTGGTCACA
GGAAGTGGAGAGGAAGAAGAGATAAATATTGCAACTAATAGTAGTGTTGAGCAAGAGATGATAATACCAACTGAGGAACA
TAATGCTAAGATCATCACCAGAGGAATCTATGACGGTGAGATCTCATTTGGAAGCAGTTGGAAGAATGTTGGTAGTAGCA
GGAGCATAAGGGAAATTGTTTCAATAGCTGAAGAAGAAGAAAATGAAGATGAAGATGTCACTGTCACAAATTACCAC
ATTTGATTTCATAGGAATTGGACTTATAATATTTATTTCAATAAAAATAAAACAAAACTGATTCTTAAGCAATAATACTA
TCA

>CaMLO10 LOC101494654
ATGGCTGCAACTGAAAGTGATAGTTACAATAGTTCAAGAAAACTTGATGAGACACCAACATGGGCTGTTGCTTCTGTTTG
CACTGTTTTCATTTTGATATCCATAGCTCTTGAGAAGAGTCTTCACAAATTGGGAATGTGGTTAGGAAGAAGGCAAAAGA
AGGCTTTGCTTGAAGCTTTGGAAAAAATCAAGGCTGAGTTGATGATTTTAGGATTCATTTCACTGTTATTGACTTTTGGG
CAGAATTACATTGTAAGAATATGTATTCTTGAAGAAGTTGCAAACAAAATGTTGCCATGTCCACATAAATATAGTAATAT
AAGTGATGACAAGAAATCAAGTAGTGAAGTGGTAAATGTTAGGAAACTTGTGTCTTATGAAGGTAGATTTTTAGCTGCTG
ATACTCCCTCCTTTAAATGCAGCAGGAAGGGGCATGAACCTCTTTTATCTATCAATGGATTGCACCAGTTACACATCTTC
ATATTCTTCCTAGCTGTCTTTCATGTGCTTTATAGTGCTATCACAATGCTTCTTGGGAGACTTAAGATACGTGGATGGAA
GGAATGGGAGGAAGAGACTTCAACTCATGGTAATCAATTTGCCAATGATGCTGCAAAATTTAGGCTCACACATGAAACAT
CATTTGTAAGAGCTCATACCAGTTTTTGGACAAGGATTGCTATCTCCTTCTATATTGGTTGTTTCTTTAGACAATTTTAT
AGGTCTGTTCGTAGGGTTGATTACCAAACTTTGCGCAATGGATTTATCAGTGTCCATCTAGCTCCTGGAAGTAAATTTAA
TTTCCAAAAGTATATCAAAAGATCATTAGAAGATGACTTCAAAGTAGTTGTGGGAGTAAGTCCTGTTCTCTGGGCATCAG
CGGTAGTTTTTCTGCTCCTAAACATTGATGGTTGGCGTACTCCGTTATGGGCATTCTTAATTCCAATTATTATAATTTTG
GCTGTTGGAACAAAACTTCAAGTAATATTAGCAAGGATGGCTCTTGAAATAACAGAAACACATGCAATTATTCAAGGAAT
GCCTCTTGTTCAAGGATCAGACAAATACTTTTGGTTAGATCAACCTCAGTTAGTTCTTCATCTTATCCATTTTGCTTTGT
TTCAGAATGCATTCCAAATAACATATATATTGTGGACATGGTATACTTTTGGGCTGGAAAATTGTTTCCGTTTGGGCTAC
AAACTTGCCGTTATAAAAGTTGCTTGCGGGGTTGTGATGCTATGTCTCTGCAGCTATATTACCCTTCCATTATATGCTCT
TGTGACTCAGATGGGTTCAAGGATGAAGAGGTCAATATTTGATGAACAAACATCAAAAGCATTAATGAAATGGCACATGA

CTGTGAAAAAGAAGCATGTAGGAGAAGTAGTCAAACTTGGAAAGTCAACCATTGATGGTAGCAACATTGGATCAACATTG
CAATCCCCTGGACCAGCACTACACCGTTTCAAAACTACTGGACATTCAACTCGCTCTTCAACTTTTGAGGATCAAGATGA
ATATGAATCTGATAATGAATTGGAAAAAACTAAATTGATTCTAAGAGTTGATCAACTAGCAGAAGAAGATCATAGGCATC
ATAGTGAGGGAGAAGCTAATAATGAAGATGAGTTCACTTTTCTCAAGCATACTACTATTCAAAGTTGAG

>CaMLO11 LOC101511064
ATGGCAGCAGAGAATGAACAAGAAACAAGGTCATTGGCCTTGACTCCTACATGGTCTGTTGCAACTGTGTTGGCCATCTT
TGTTGCTGTCTCTTTGCTTGTCGAGCGCTCCATTAACCGTTTAAGCATTTGGCTTCGAAAAACTAATAGAAAACCTTTGC
TCGAAGCTTTGGAGAAGATGAAAGAAGAGTTGATGTTGCTCGGATTTATATCTCTCCTACTTACTGCTACCTCTGGCATG
ATAGCAAATATTTGCATTCCGTCAAAGTTCTACAATAGTGCTTTTGATCCCTGCTCTCAATCTGAGATTGATAAAGAAAT
TGAAGAGAATGGCTCCGAAGGACGTAAACTATTGATGGCTGCTTCTTACCCACGCATAATTAGAAGGGTGTTGAATGGAA
TAAATAGAAACTCCTGCAAAGTGGCAGGTTATGAACCGTTTGTTTCGTATGAAGGCCTAGAGCAGTTGCATCGGTTCATT
TTTGTCATGGCGATCACACACGTATCTTACAGTTGCCTAACAATGTTGTTAGCCATTGTGAAGATTCACAGTTGGAGAGT
GTGGGAGGAGGAGGCTCACATGGATCGACGCAATTATTTAACAGAAATTACTAGGCAGATGACTATGCGAAGGCAATCAA
CCTTTGTCAAGTCTCATACATCAAATCCATTAGTCATTTGGATGACATGCTTTTTTCGACAATTTGGGCGTTCTGTGGTT
CGCGCTGACTACCTTACACTTCGAAAAGGCTTCATCATGAATCACAACCTTTCATCAAAATATGACTTCCACAGCTACAT
GGTTCGATCTATGGAGGAGGAATTCCAAAGGATAGTTGGTGTGAGTGGCCTACTCTGGGGATTTGTTGTTTGTTTCATGC
TTTTCAACATTAAAGGATCTAATCTATATTTCTGGATAGCTATCATTCCTATATCTCTTGTTCTTTTGGTGGGAACAAAA
CTACAACAAGTTATTGCAACCTTAGCATTGGAGAATGCTGGAATTACCGGTTTCACCTCAGGAGCTAAGTTGAGGCCTCG
CGATGAACTTTTTTGGTTTAAAAAGCCTGAGCTATTACTTTCCTTAATCCATTTCATTCTCTTCCAGAATGCATTTGAAT
TGGCTTCATTCTTTTGGTTTTGGTGGCAGTTTGGATATAATTCCTGCTTTATTAGAAACCACCGCCTTGTTTATTTAAGG
CTAATCTTGGGGTTTGCCGGACAGTTTCTTTGCAGTTACAGCACCTTGCCACTCTATGCTCTCGTCACCCAGATGGGAAC
AAACTATAAGGCAGCATTAATCCCACAGAGGATAAGGGAAACCATTCATGGATGGGGCAAATTAGCTAGGAGGAAAAGAA
GACATGGCATGTTCATTGATGACTCCACCATTCATACAGATACAAGCACTGTATTGTCTATTGAAGAAGAGGATCAAATA
ATAGATGATTCCTTTAAAGACCTTGTCACTGGTACTGGAGTAGAATTACAACCAGTAAAAACTGTTACATCTATCTCTTC
ACCTATTGCTAATGAAACTTCAAGTAGGGCTGTTACGCCACTCCTCCGACCCTCCGCTTCACTATCTTCATCAGTACCGT
TCAGTTCTAGACACGAACCAGTTATAAGATCCTCCTCTATGCCAAGTGGAAGATAA

>CaMLO12 LOC101496809
TTTTCAATTAGTTTGGCAAAAAAAATAATAATAAAAAAATATCAATAAATTCACGCGCTTATGAACAGACCATTTTCCTT
TTCAAAAAACCAACTCCAATCAGATAAAACTATTACCTTTTTCTCATTCTAAACCAACCCATATGATCTTTTTACTTGCA
TTTTAATTTTCACTTTTTTTCTTCTCTTTTCCCTCAGTTCTGTGATCATTTTTGAATTTGGGTTGTTGTGTTTGTTATTG
GTTTCACCTATGTGTATATCAATGTATAAAAAAAGGTTGTCTTTTTGTGTGGTTTCATTTTCATGGGTGTTGTTAAATGG
AGGTGTGGCTATGGCTTCAAGTGAAAGTAGTGACAGTTCTAGAGACCTTGATCAAACACCAACATGGGCTGTTGCTTGTG
TTTGCACTGTTTTCATCTTGATTTCCATTACTCTTGAGAAAAGTATTCACAAAGTTGGAACGTGGCTGGGAGAGAGGCAC
AAGAAGGCTTTGCTTGAAGCTCTGGAAAAAGTCAAAGCTGAATTGATGATTTTAGGTTTCATTTCATTGCTTTTGACTTT
TGGGCAGAGCTACATTGTTAGAATATGTATTCCTGCCAATATGGCAGACAAATTGTTGCCATGTCCATATAGACATACCG
GATCAAAAAATGAAGAGGAGCATCACAGGAAACTTCTACATAGATATTTATCAGAAGATGCTACACCTTACCAATGCAAG
GAGGGACACAAGCCCCTTATATCTGTTAATGGATTGCACCAGCTACACATCCTCATATTCTTCTTAGCAGTCCTCCATGT
GTTATACAGTGCTGTCACAATGCTTCTTGGGAGACTAAAGATACGAGGATGGAAGGCATGGGAGGCGGAGACATCGTCTC
ATGGATATGACTTCGCCAACGATCCTTCAAGATTCCGGCTTACACACGAAACATCATTTGTTAGACAGCATGCCACTTTT
TGGACAAAAATTCCAATCTTCTTCTATATAAGTTGCTTCTTCAGACAGTTTTATAGGTCTGTGGATAAGGCTGACTACTT
GACTCTTCGCAACGGATTTATCACCGTCCACTTGTCAGCGGGAAGTAAATTTAACTTCCAAAAATATATCAAAAGATCGT
TGGAGGATGACTTCAAGGTAGTTATGGGAGTCAGTCCTGTCCTTTGGGCTTCATTTGTTCTTTTTATCCTTCTAAATGTT
GATGGATGGCATGCTATGTTTTGGGCATCTCTGATTCCCGTTGTTATAATTTTGGCCGTTGGAACAAAACTTCAAGCCAC
CTTGGCAAAGATGGCTATTGAAATCACAGAAAGACATGCAGTTATCCAAGGAATTCCTCTAGTCCAAGGCTCAGACAAAT
ATTTTTGGTTTGGTCGCCCTCAGTTACTTCTTCATCTTATCCATTTTGCCTTGTTCCAGAATGCCTTTCAAATAACATAT
TTCTTGTGGATATGGTACTCTTTTGGGCTGAAAAATTGTTTTCATGCTGATTTCAAACTTGCCATAGTCAAAGTAGCTTT
AGGGGTTGGGGTGCTATGCCTCTGCAGCTATATCACTCTTCCATTATATGCTCTTGTTACTCAGATGGGATCAAGAATGA
AAAAATCAATATTTGATGAACAAACGTCAAAGGCCTTAAAAAATTGGCACAATACTGTGAAAAAGAAGCATGGACTTAAA
CTAGGAAAGTCGAGTGTCCGAACCATGGAAGGAAGCACCACCGATTCAACAGTTCACTCTACAACCCCCACGCTACTGCG
TTTCAAAACAACAGGTCACTCAACTCGCTCTGTTTCAGCCTACGATGACCAAGACGATTACCATTCTGATATTGAAATGT
CTCCGACATCACCAACATCAAACTTGCTTGTAAGAGTAGATCATGTTGAGCAACATACAATAGAAAATGAACATCAAACC
GAGGTCAAAACCATTGATAAACAACCAACAATGAGAGGTAGCTTCTCATTTGTCAAGCCTGATGATCATGTGGAAAGAAG
CACAGATTAATTAAATAGCATCACAAGTTACTTCATTGAACCATTTTTTCTTGTTAGCTTATATATACAGAGAGAGAGAG
AGATTTCTAGATATTAGTAATACTAAAATTAGATAAAGTAAGTTTTGTACATTCTTTTTTATTTCTTAGATAATTTTTGT
GCATTGGAAACTTTACTCTGGATCCAAATCATGCTGCTTATAGTTAAAAGCTTTATTATGAGCAATGCCTAATTTTAATT
TTACTTA

>CaMLO13 LOC101510624
AAAGAATTGTCTTCAACTATCTCTCTGCCAAAACTGTATTGCTAAAAAACATGGCCGAAGAACTGAACCAGTCTCTTGAA
TATACACCAACATGGATTGTTGCGGTTGTTTGCTCCATCATTGTTTTCATCTCTCTGTGCGCTGAGCGTGCCCTTCACTA
CCTTGGAAAGTTCTTGAAGAAGAGAAGTCAGACAGCATTATATGAAGCCTTAACAAAATTGGAAGAAGAGTTGATGCTTT
TAGGTTTCATTTCCCTTCTCTTAACCGTCTTTCAAGGTCTAATAAGCGACATTTGCATCACACCTAAGCATGCAACTCAA

ATGCTTCCATGCAAGAGGTCTGATGAAGGTTTAGAGCATGAGATACACTATGATACTATAATCAACAAAAGAAGGCTTTT
TTCTTCAGATTCCGGTTCGCAGCATTGTAGGCAAGAGGGGAAGGTACCACTGTTATCACTGGAAGCATTGCATCATCTTC
ACATTTTCATCTTTGTATTGGCTGTTGTACATGTAGTATTCTGTGTGGTAACAATGATTCTTGGAGGTGTAAGGATCCGT
GAGTGGAAAAGCTGGGAAGATCATGCTCAGAGTATGGCAATAAGTTCAAGTGGTGAAACTCTTAGAGTAGAGGAAGCTGG
GTTCTTCGATAAATATGCTCAAGGGTATTGGAGAAAAGCAGCTGTTGTTGGTTGGCTGAGATCATTCTTCAAACAATTCT
ATGGATCTGTTACAAAGTACGACTACCTTGCGCTGCGGCATGGATTTATCAAGGAACACTGCCCGAACAAGCCCAGTTTT
GATTTTCATAGTTATATGATAAGGACACTTGAAATTGACTTCAAAAGAGTTGTAGGCATAAGTTGGTACCTGTGGCTGTT
TGTTGTGCTGTTTTTGCTGTTGAATCTTGAAGGCTGGCACACTTATTTCTGGTTAGCCTTTTTACCATTGATAATTCTAC
TTCTTGTGGGGGCAAAGTTAGAGCATATAATCACTCGTTTAGCCCAAGAGGCGGTAGCAAATGTAGACCGGACTGAACGA
GTGAAACCATCCGATGAATACTTTTGGTTTGGCCGCCCCGCCATTGTCCTACACTTACTTCACTTCACTTTGTTTCAAAA
CGCGTTTGAGATCGCATTTTTCTTCTGGATTTGGTGCACATATGGATTTGATTCATGCATTATGGAGAAAATAGCCTACG
TCATCCCAAGACTTATAATGGGTGTAATTGTTCAAGTGCTTTGCAGTTACAGCACCTTGCCCCTTTACACTATTGTAACT
CAGATGGGTAGCAGTTACAAGAAGTTTGATGATATGGGGGAATCATCTTCTTTGTTCCATAGAATGACCAAAGAATCAAA
TCAACATCCTCAAATTGGTGAACAATCAATTATCATGATGGAAAATCATGCTATATCATCTACAATTGAGCTTTCACATA
CTGTCTAATCTCCTTTGGAGAGACCTAATACATTCAATACTTTATTCTTATT

>CcMLO1 C.cajan_46346
ATGGTCGAAGAAAAGCGAGTTTATGAACGAACACTAGAGGAAACACCAACATGGGCTGTTGCAGTCGTGTGTTTTGTGTT
GCTTGCTATTTCAATCCTCTTAGAACATATTATTCATGCGATCGGAAAGTGGTTCAAAAGGAAAAACAAGAATGCTCTTT
TTGAAGCGTTGGAAAAGGTTAAAGGAGAGCTTATGGTGCTAGGATTCATATCCTTGATGCTAACTGTGTTGCAAGATCCA
ATTTCGAAGATCTGTGTGTCCCCAAAGGTTGCATCTAGTTGGCATCCTTGTGCCAACCCAAAACCATTTGGTACAACTGA
GGATAAATCTGGTGAGGATGAATCTGGTGATGATGACGTTGAGATCAATTCTAGGAAACTCCTCCAATATTATTATGAAA
CCATACCAAGGCGTGTTCTGGCTACAAAAGGATATGACAAATGCTTTGCAAAGGCAAGTTCACCATCTTCACTGTTGTTT
GGAAAAGTGGCTTTTGTTTCAGCATATGGGATTCACCAGCTCCATATGTTTATCTTTGTGCTAGCTATTTTCCATATCTT
ACAATGCATTGTGACACTGACTTTGGGAAGAACCAAGATGAGAAAATGGAGGGCATGGGAAAATGAAACGAAAACCATTG
AATATCAATTTTATAATGATCCTGAGAGGTTCAGGTTTGCAAGGGATACGACATTTGGAAGAAGGCACTTGAACTCTTGG
AGTCAGTCAGCATTTTCCTTGGCTATAGTTAGTTTCTTTCGACAATTCATTGGATCTGTCAATAAAGTTGACTATTTAAC
ATTACGCCATGGATTTATCACTGCGCATCTGGCACCAGGAAGTGATGCAAGATTTGATTTTCAGAAGTATATCCAGAGAT
CACTTGAGGAAGATTTTAAAGTTGTAGTGGGAATAAGCCCAATCATATGGTTCTTTGCGGTGCTATTCCTGTTGACTAAT
ACTCATGGGTGGTACTCTTATTATTGGCTTCCATTTATGCCATTAATTATAATCTTATTAGTTGGTGCTAAGCTACAAAT
GATCATAACAAAGATGGGACTGAGGATTGCAGACAGAGGGGAAGTTGTGAAAGGTGCACCTGTGGTTGAGCCAGGAGATG
ACCTGTTCTGGTTTAACCGTCCACGCCTCCTCCTCTTCTTGATTCATCTTGTGCTCTTTCAGAATGCTTTTCAACTGGCA
TTCTTTTCTTGGAGTACTTATGAATTCTCCATAGACTCTTGCTTCCATGAAACAACTGTAGATATTGTCATAAGACTTGC
AATGGGGATTATCATACAAGTTTTATGCAGCTATGTGACTTTGCCTCTGTATGCTCTAGTGACACAGATGGGTTCAACCA
TGAAGCCTACAATTTTCAATCAGAGAGTAGCATCAGCACTGAAGAACTGGCACAACACTGCCAAAAAGCAAATAAAAAAC
AGTAAGCAAACCACACCATTCTCAAGCAGGACATCAACTCCAACACATGGTATGTCTCCTATTCATCTGCTACAGAGACA
CCTTGCTGGTAGAAGTGACAGTGCACAAACATCTCCAAGGGCATCCAACTATGAAAATGAACAATGGGATCTTGAAGGCT
CACCTTCCCCAAGCAACCATGCAGTAGCATCTAATGAGACCCAGATGCAAGTTTTTGAACCAGGCACAACTTCAGAATTG
CCTGTCAGCTCTCAACTTGAAATCAGAGTTAGCTCCTCAGAATTTTCCTTTGAGAAGAGACATCATGAGGGAGGTGATTA
G

>CcMLO2 C.cajan_07194
ATGCTTGCTATTTCAATCTTCATTGAACATGCTATTGAATCACTTGGAAAGTTGTTAAAAAAGAAACACAAAAAGGCTCT
TCATGAAGCGCTGGAAAAGGTTAAAGGAGAGCTTATGCTGCTTGGATTCATATCCTTGCTCCTAGTGGTGTTTGAAGATC
GTATTTCTGATATCTGCATCTCAAAAGAAGCTGCATCCTCATGGCATCCTTGTGGTCCCGATGATAAGCATAAAGATCCA
AAAGGATATTATGACAAATGCGCTGAAAAGGCAAGCTTATTCCCTCATTTTTTTATAATTTACTTTCATTGCATTAGATA
TTACCAACAATTAAAGCCTACATGTTTTCCTTCAATTTTATTTTTTGATATTTTGCAGGGTAAAGTTGCTTTTGTGTCTG
AATATGGGATTCACCAGCTCCATATATTCATCTTTGTGCTTGCTATTTTTCATATCCTACAGTGCATCATAACACTCACT
TTGGGTAGAACTAAGATGTCAATATGGAGGATGTGGGAAGACGAATCAAAGAGTCTTGAACATCAGTTCTACCATGATCC
TGAGAGGTTCAGGTTTGCTAGGGATACAACATTTGGACGAAGGCACTTGAGCTCATGGAGTCGATCACCAATTTCCTTAT
GGATAGTTAGCTTCTTCAGACAATTCTATGGGTCAGTTAATAAAGTTGATTATATGGCGTTACGGCATGGATTCATCGTG
GCACATCTGACTCCAGCAAGTGAGGCAAAATTTGATTTCCAGAACTATATCAAGAGAACACTTGAAGAGGATTTTGTAGT
TGTGGTGGGCATAAGTCCAATCATGTGGTTCTTTGCGGTGCTAATCCTGCTCACAAATACTCATGGGTGGTATGCTTATT
TCTGGCTTCCATTTATCCCATTAATTATAATCTTGTTGGTGGGAACAAAGCTACAAATGATCATAACAGATATGGCACTA
AGGATTCAAGATAGGGGGGAAGTGGTAAAGGGTGCACCTCTGGTTGAGCCAGGAGATGAATTGTTCTGGTTCAATCGTCC
CCGCCTCGTCCTCTCTTTGATTCAACTTGTTCTCTTTCAGTCTATGCTGCAGAATGCATTTCAACTAGCGTTTTTTGCTT
GGAGCACATATGACAATGGGTTAAAAATAAACTCTTGCTTCCACAGAACTACTGCAGATATTGTCATTAGACTTACAATG
GGGGTTCTCACACAAGTTCTATGCAGCTACGTCACTTTACCTCTTTATGCTCTAGTCACACAGATGGGTTCAACCATGAA
ACCAACAATTTTCAATGACAACGTAGCAGCAGCACTAAAGAAGTGGCATCACACCGCTAGAAAGCATATAAAACACAAGG
ATTCGAGTCATTCTGCAGCCAACACACCATTCTCAAGCAGGCCAGGAACTCCAACACATGGCATGTCTCCAGTTCACCTG
CTTCACAAACACCCTAGACACAGTGATAGTCCTCTAACTTCTCCAAGGGCACCTCATTACCATAATGAACAATGGGATGT
TGAAGGGTCACCTTCCCCAAGTCATCATGCAAGAAATCATGAAGACACCATGCAGCCTCCAACAGCAGAATTGACTCCTA
TTGGACTCCAACCTATTAGGACTCAACATGAAATCAACATTGCTTTGTCTGAATTTTCATTTGGGAGGGGACACCACACT
AATAATAGTAACTAG

> CcMLO3 C.cajan_32587
ATGGCCGAAGACAAAATTTATGAACGATCGCTTGAGGAAACACCAACCTGGGCGGTTGCAGTTGTATGTTTTGTGCTGCT
TGCTATTTCAATCATCATAGAACATATTATTCATGCGATTGGAAAGTGGTTCAAAAAGAAACACAAAAGTGCTCTTTATG
AATCGTTGGAAAAGGTCAAAGGAGAGCTTATGATGCTAGGATTCATATCCATGCTCCTAACTGTGTTCGAAGGTCCACTT
TCTAAGATCTGCATATCGCAAAATGTTGCATCCACGTGGCATCCGTGTCACAAACCAAAATCTGACACTGGCACCAATGG
TAGGAAACTCCTCCAATATTTGGAACTCATATCTCGGCGTGTTCTAGCTGCGAAAGCAAATGACAAATGTGCTGAAAAGG
CAATTTTGATGCATTATGTTGCATTTAATTTGCAGGGAAGAGTTGCTTTTGTTTCGGCATATGGGATTCACCAGCTCCAT
ATATTCATATTTGTTCTAGCTGTTTTTCATATCCTACAATGCATTATAACACTAGCTTTGGGAAGAACTAAGATGAGACG
CTGGAAGAAGTGGGAAGATGAAACAAAGACAATTGAATATCAATTCTATAATGATCCTGAAAGGTTCAGGTTCGCAAAGG
ACACAACATTTGGACAAAGGCACTTAAATACGTGGAGTCACTCATCAATTTCATTATGGATAGTTAGCTTCTTCAGGCAA
TTCTTTGGATCTGTTAAGAAAGTTGACTATTTCGCATTACGTCATGGATTTATTACCGCACATCTGGCACCAGGAAGTGA
TGCAAGATTTGATTTCCAGAAGTATATCAAAAGATCACTCGATGAGGATTTTAAAGTTGTCGTGGGAATAAGTTTAACTT
TTTGGTTCTATGGGTGGTATTCTTATTACTGGCTTCCATCTATCCCATTAATTATAATCTTATTAGTGGGTGCTAAGCTA
CAAAAGATCATAACAAAAATGGGACTGAGGATTCAAGACAGAGGGGAAGTAGTTAAGGGTGCACCTGTGGTTGAGCCAGG
AGATGACCTGTTCTGGTTCAACCGTCCACGCCTCCTTCTCTTCATCATTCACCTTGTTCTCTTTCAGAATGCCTTTCAAT
TAGCACATTTTGCATGGAGTACATATGAATTCTCCATAAACTCTTGCTTCCACAGATCAACAGCAGATATTGTGATCAGA
CTTACAATGGGGGTTGTCATACAAGTTCTATGCAGCTATGTGACCTTGCCTCTTTATGCTCTAGTCACACAGATGGGTTC
AACCTTGAAACCCACCATTTTCAATGAAAGAGTAGCAGCAGCATTGAAGAAGTGGCACCATGCTGCCAAAAACCATGTGA
AAGACAGCAAGCACTGCGAGGCTAATAGTACCACAGCATTCTGCAGCAGGTCATCAACCCCAACATTTGGCATGTCCCCT
GTTCACCTGCTAAACAGACACCTTGCTGGCAAAAGTGATAGTCCACAAACTTCTCCAAGAACATCCAACTACGAAAATGA
ACAATTTGATTTTGAAAATGAACCTTCCACAAGCACCCATTCAGGAACAGAGGAGACCCCAATGCAAGTTTTTGGGCCAC
CTTCGACAACAGAATTGCCTATTAGCACTAAGCTTGAAGTCCACGTTAGTTCATCAGATTTTTCTTTTGAGAAGTAA

>CcMLO4 C.cajan_12516
ATGGGGGAAGAAAATGAAGAAGGAAGATCCTTAGCTGAAACCCCCACATATGCTGTGGCAACTGTCATCACTGTCTTGGT
TTCTCTCAGTTTCTTGTTTCAAGGCACGTTAAAAAAATTAGTAAAGTGGTTAGACAAAACCAAGAGAAAATCTCTGCTTT
CTGCTCTGGAAAAGATCAAAGAAGAATTAATGCTTTTTGGACTTCTGTCATTGCTGATGGGTCATTGGATTATTTTTGTG
GCAAAGATTTGTGTTAAGTCATCGGTATTGAGCAGCAGATTTTTTCCATGTGCAATGGACAAGAATTCTGCGAAACGTAT
TGTTTGGCCAAGTTCAGAATCTATAAATAAAACAGATTTCAAAGGACTAGAGATCTCTGGCCGGCATAATTATTGTCCTG
AGGGTCATGAATCATTGGCTTCTTATGAAAGTCTTGAACAGCTTCATCGCTTTGTGTTTGTTCTTGGGGTCACCCATATT
ACCTATAGCTTTATTGCTGTTGCCCTGGCCATGATCAAGATATATAGCTGGAGAACATGGGAAAATGAAGCAAAGACTAC
TGCTATACAAAGCATACAAGATACTCCACAAAGTACATCAAGCATAAGATTGAGGCGGCTTAGTACATTCATCTTTCACC
GTACATCCCATCCTTGGAGTAATCACAAAATTCTAGTTTGGCTGCTATGTTTCAGCCGCCAATTTTGGAGTTCCATTCAC
CGAGCTGATTACATGGCATTGCGCTTAGGCTTCATTACCAATCATGAGCTTCCCCTAACATACGATTTCCACAACTATAT
GCTTCGAAGCATGGACGAGGAATTTCGTGACATAGTTGGTGTTAGTGTGCTTCTCTGGGTATATGCCATCTGCTGTATTT
TTCTAAATTTTCACGGAAGCAACTTTTACTTTTGGCTTTCCTTCGTTCCAGCAATTTTGATCCTCATAATTGGTACTAAA
TTGCATCGAGTGGTGGTAAAGTTGGCTGTTGAAATTGTAGATCGTTGCCCAGTTATCAAACCTCACCGGTTTAACTTAAG
AGATGAGCTATTTTGGTTTGGAAAGCCTAGGTTTCTGTTACGGTTAATACAATTGATATCCTTCTTGGTTATCTTTCAGA
ATGCATTTGAAATGGCAACATTCTTATGGTCCCTGTGGGAAATTAAAGATCCTTCTTGCTTCATGAGCAACCACACATTT
ATCGTGATCCGCTTGTCATTCGGGGTCATCTCCCAAGTCTGGTGCAGCTTTATAACGTTTCCTCTCTATGTCATCATCAC
ACAGATGGGGTCAAGGTTCAAGAAGACTGTGGTCACTGAAAATGTAAGAAAATCACTCTCTAAATGGCAGAGAAGAGTGA
AGGACAGACAAAGTTCCTCTTATGCAGTCCTTACCGCAATATCAGCATCGTCATATGGAACTGTGTCTAATTCCCGTGCC
TCAAATAGTGAGGAAGGAAGCTGCTCAGGGATTAAAGATATTTGTCTTGATAATGTGTCAAACACCCAAGAAAATGCACC
ATTTCAAAGTTATGGAGATCATCATGATCATAATCATAATGTAAACGATGATTCATCTTCCCCTCCTCCATCTTGA

>CcMLO5 C.cajan_34110
ATGGAAGGAAGAAGCTTAGCGGAAACACCTACTTGGGCTGTGGCTGTTGTTTGTTTTGTTTTGCTCTCCATATCTATCTT
CATTGAGCACATCCTCCATCTCATAGGAAAGTGGTTGAAGCAAAAGCAGAAAAGTGATCTATACGAGTCACTTGAAAAGA
TCAAATCAGAGCTTATGTTATTGGGGTTCATATCGTTGCTACTAACAGTAGGACAAGGTGTAATATCGAGGATTTGTATA
ACAGAAAAGGTTGCCGACACATTTCACCCCTGCCCAAAGGATCATCATCAAAACACTAGTGCCCGGAGATTACTTTCCGA
TAACCAAAATCACCGTCGTATTTTGGCGGCCGGAGGAAGTGACAAGTGTCCTCCGGGTAAAGTCCCGTTTGTGTCATCGG
AGGGCATTCATCAACTCCATATATTTATCTTTGTGCTCGCTGTTTTTCACGTCCTTTACTGCATACTTACTCTGGCTTTG
GGTAGGGCAAAGATGAGAAGGTGGAAACGATGGGAAGAGGAAACCAAGACAGCAGAGTACCAATTTTCACACGATCCTGA
ACGGTTCAGATTTGCGAGGGAGACCTCGTTTGGGAGAAGGCACTTGAAATTCTGGACCCAAAATCTTGTCCTTGTTTGGA
TTGTTTGTTTCTTCAGGCAGTTCGTACGTTCAGTCCCTAAAGTAGATTATTTGACCTTGAGGCATGGATTTATGATGGCT
CATTTGGGGCCTCAAAGTCACATGAAATTCGACTTTCGACAATATATCAAAAGATCTTTGGAAGAGGACTTCAAAGTTGT
CGTCGAAATCAGTCCTCCAATATGGTTCATCACGGTATTTTTTCTCCTGTTTAACACTCATGGCTGGTATTCTTATCTAT
GGCTGCCATTTGTTCCTTTGATTATCATTCTATTAGTTGGAACCAAGTTGCAAGTGATCATAACAAAGATGGGTCAAAGA
ATTGAACAAAGAGGAGAGGTAGTAAAGGGCGTGCCACTAGTGCAACCTGGAGATGACCTATTCTGGTTTAAAAAACCTCG
ACTTATTCTCTACCTCATTAATTTTGTGCTCTTTCAAAATGCTTTTCAAATGGCCTTCTTTTCATGGACTGCGCTTCAGT
TTAGGATGACATCTTGTTTTCATTCCGAAACAAAGGATATGGCAATCAGAATCTCAATGGGGGTCTTCGTTCAATTCCTT
TGCAGCTATGTGACCCTTCCTTCCTATGCTCTCGTCACACAGATGGGTTCAACGATGAAGCCAACCATATTCAGCGGAAG
AGTAGCGAAGGCTTTAAGGAAGTGGCACCACACAGCGAAGGAGCACGTAAAGCAGAACCGTGGATTGCAATCACATAACT

CTTTGCCATGCAAGCCCACAACCCCCAAGCCCCCTACGTCCCCGGCCCAACTCCTTCGCCACTGCCACAGTCAAATGGAC
ACCTATCCCTCATCTCCGACAAGATCCGATTCGGAGGCCCAATACCACTACGAGATCGATTCAACCTCATTCTCCGACGA
GCATCATCACAAATCCAAGGCTGTTGCTGATTCTAATTCAATTCAGCTCCATGAAATGGAAATGGGCCACCTGGCCCATG
ATCAACCACAGAGGGACAATGAGCCCAGCTCTACCCTTGAGGGTTTAGGTCTGAGTCACCACGAGATTCATATTGAACAT
ACCAAATAA

>CcMLO6 C.cajan_34112
ATGGCAGGAAGAAGCCTAGAGGAAACGCCCACTTGGGCTGTGGCCGTTGTTTGCTTCGTTTTGCTCTCCATATCTATCTT
CATCGACCACATCTTCCATCTCATTGCAAAGTGGTTGCAAAAGAAGCACAAAAAAGCTCTATTTGAGTCACTCGAAAAAA
TCAAATCAGAGCTTATGTTATTGGGGTTCATATCGTTGCTACTAACAGTAGGACAAGGTGTAATATCGAGGATTTGTATA
ACAGAAAAGGTTGCCGACACATTTCACCCCTGCCCAAAGGATCATCATCAAACCAGTAGTCGCCGGAGATTACTTTCCGA
TAACCAAAATCACCGTCGTATTTTGGCGGCCGGAGGAACCGACAAGTGTCCCCACGGTAAAGTCCCGTTTGTCTCATCGG
AGGGTATTCACCAACTACATATATTTATCTTCGTGCTCGCTGTTTTTCATGTCCTTTACTGCATACTCACTCTGGCTTTA
GGTAAAGCAAAGATGAGAAGGTGGAAACGATGGGAAGAGGAAACCAAGACAGCAGAGTACCAATTTTCACACGATCCTGA
ACGGTTCAGATTTGCAAGAGAGACATCGTTTGGGAGAAGACACTTGAAATTTTGGACCCAAAATCCTGTCCTTGTTTGGA
TTGTTTGTTTCTTCAGACAGTTTGTACGATCAGTTCCTAAAGTAGATTATTTGACCTTAAGGCACGGATTTATGATGGCA
CATTTGGGGTCTCAAAGTCACCAGAAATTCGACTTTCGGCAATATATCAAAAGATCTTTGGAAGAGGATTTTAAAGTGGT
TGTTGAAATCAGTCCTCCAATATGGTTCATCGCAGTGCTTTTTCTCCTGCTTAATACTCATGGTTGGTACTCTTATATAT
GGCTACCATTTGCTCCTTTGATTATCATTCTTTTAGTTGGAACCAAATTACAAGTGATCATAACAAAGATGGGTCAAAGA
ATTGAACAAAGAGGAGAGGTCATAAAGGGCATGCCACTAGTGCAACCAGGGGATGATCTATTTTGGTTTAAAAAACCTCG
ACTTATTCTCTACCTCATTAATTTTGTGCTCTTTCAGAATGCTTTTCAGCTTGCCTACTTTTCATGGACCGCGCTTCAAT
TTGGCGTGAAATCTTGTTTCCACTCCCATACAGAGGATTTGCTGATCAGAATCTCAATGGGGCTCTTTGTTCAATTCCTT
TGCAGCTACGTGACTCTTCCTCTCTATGCTCTCGTGACACAGATGGGATCGAGTATGAAGGCAACCATATTCAGTGAAAG
AGTGGCGGAGGCTATAAGGAAGTGGCACCACACGGCGAAGAAGCAGATAAAAAATAACCATGGGCTGGCCTCACAGTCAC
AGACCCCTTTCTCAAGCAGGCCCACAAGCCCGGCCCATCCCCTTCGCCACTACCGCAGTGAAATAGACACCTTCCTCTCA
TCTCCAATAAGATCCGAGTCTTTGGCCCATCAGCCCAACGACATTGATTCACTCTCATACTTTAACTCACATCATCACAG
GTCCAAGGTTAATGCTGCTTCTATCTCAATTGAAATGGGTCACCTGGCCCATGATCCACCACAAAATGTGAAAGAACCCA
GCTCTGCCCTTGAGGGCTCAGACCCGACTCAACACGAGAGGGAATTTTCATTTGATAAAAGATTACAAATTTAA

>CcMLO7 C.cajan_21483
ATGGCAGGAGGAAGTGGAGGAAGGAACTTGGAGGAAACGCCTACATGGGCAGTCTCAGCTGTTTGCTTTGTTTTGATCAC
AATATCTATAATCATCGAGCATATCATCCATCTCATAGGAAAGTGGTTGAAGAAGAAGCACAAAAGAGCTATGTACGAGT
CACTTGAAAAGATAAAATCAGAGCTTATGTTATTGGGGTTCTTATCGTTGCTTCTAACGGTGGGACAAGGTCCAATATCA
AGGATATGTATATCGGAAAAAGTTGCAGGGACATGGCACCCTTGCAGCAAGGACGACGAAAGCCATGACTCAGATAGTGA
AGATTCGCAACACGGAACCAATGGTCGTAGGTTGTTAGCGGCGTTCTATGCTTCGGACACGCGGCGTCTTTTGGCCGGAG
GAGGAAGCCACAAGTGTGCCCAGGGTAAAGTCCCGTTTGTGTCATCGGATGGCATTCATCAACTCCATATATTTATCTTC
GTGCTGGCTGTTTTCCATGTTCTTTACTGCGTCATCACTATGGCTCTGGGTAGAGCAAAGATGAAAAGGTGGAAACGATG
GGAAGAGGAAACCAAGACACCTCAGTACCAATTTTCACACGATCCTGAGAGATTCAGATTTGCGAGGGACACATCGTTTG
GAAGAAGACACTTGAGCTTCTGGACCAAAAATCCTGTCCTCATGTGGCTTGTTTGTTTCTTTAGGCAGTTTGTGCGGTCA
GTTCCTAAAGTGGATTACTTAACCTTGAGACATGGATTTATAATGGCACATTTGGCTCCTCAAAGTCACTCCAAATTTGA
TTTTAGGAAATACATAAAACGATCACTGGACGAGGATTTCAAAGTTGTCGTTGGAATCAGTCCTCCATTCTGGTTCTTCG
CAGTGCTGTTTCTCCTCTTAAACACTCATGGGTGGTACTCTTATCTGTGGCTGCCATTTATCCCTTTGATTATCATCTTG
TTGGTTGGAACCAAGTTGCAAGTGATCATAACCGAGATGGGCCTAAGAATTCAACAAAGGGGAGAGGTACTGAAGGGCGT
GCCTTTGGTGCAACCAGGGGATCACTTGTTCTGGTTTAACAGACCAGGCCTTATTCTTTACCTCATCAATTTTGTGCTAT
TTCAGAATGCTTTCCAGCTGGCTTTTTTCTCATGGTCTGCGCTTCAATTCGGGATTCGATCATGTTTCCATTCGCATACG
GAGGATGTGGTGATCAGAATCACAATGGGGGTCCTGATTCAAATCCTGTGCAGCTACGTCACTCTTCCTCTCTACGCTCT
AGTAACACAGATGGGTTCGACAATGAAGGCAACAATTTTCAACGAAAGAGTGGCGGTTGCGCTGCGGAACTGGCACCAAA
CCGCGAAGAAGCACATAAGACAGAACCGCGTGGGACCGTTGTCGTTGTCGGGGACACCAACGTCAAGCAGACCCACAACG
CCGAGTCACCACATGTCCCCAGTGCACCTCCTACGATACTACCGTTCCGAAGCGGATAGCTTCCCTGCTTCTCCTCGAAG
GTCTAACTTGGATTCCCAACCTTGGGATAATAGTAACATCAACGTTGATTCCCCTTCTCCTTCTTACTCCCACCACCAAT
TGGAAATGGCTCCACAAACCAATGACCCCACCGTTGACACAACGCACCATGAGATTGTTGTTGCACGTCATAGCAAGGAG
TTTTCCTTTGATAAAAGACCTACTACTAATAATACGTAA

>CcMLO8 C.cajan_16636
ATGGCAGAAGAACTGAGCCAGTCTCTGGAATATACACCAACATGGATTGTCGCGGTTGTTTGCTCCATCATTGTTTTCAT
CTCTCTTTGTGTTGAGCGTGCTATTCACCGCCTGGGAAAGTACCTAAAGAAAAAAGATCAGACTGCATTGTATGAAGCCT
TACAAAAATTGGAAGAAGAATTGATGCTTTTAGGGTTCATTTCCCTCCTATTAACCGTATTTCAAGGTCTAATAAGTCAT
ATTTGCATTTCACCAAATCTTGCAACCCAAATGCTTCCTTGCAAGATACCTCATGGGTCTTCAGAAGCTTCAGAGCATGA
TCAGATATACTACGATGCTATAATAAACAGACGAAGGCTTTTTTCAACAGACACCGGTTCAGAGCATTGTAGGCAGAAGG
GGAAGATTCCATTGCTATCACTGGAATCATTACATCATCTTCACATTTTTATCTTTGTATTAGCTGTTGTACACGCAGCA
TTCTGTGTCATCACAATGGTTCTTGGAGGTGCAAGGATCCGGCAATGGAAGAAGTGGGAGAATTTTAATAGGAAGGAGTA
CATAAGTTCAAATAAAGAGTACCATGAGTTCTTCAATATACATACTGATGGACATTGGAGAAGAGCAGCTGTTGTTAGTT
GGCTGAGATCATTCTTCAAGCAATTTTTTGGCTCTGTTACGAAGAATGACTACCTTGCACTGCGATACGGATTTGTCCAG
CAACACCACCCAAACGTTCCGGAGTATGATTTTTATGATTACATGCAGCGGACACTTGAAGTTGATTTCAAAAAAGTTGT

AGGCATAAGCTGGTACCTGTGGCTCTTTGTTGTGCTGTTTTTGCTGTTGAATCTTGAAGGATGGCACACTTATTTCTGGT
TAGCCTTTTTACCATTGATACTTCTACTTCTTGTGGGGGCAAAGTTGGAGCACATAATCGCTCGTTTGGCTCAGGAATCA
ATTGATATGATGGGAAAGAAAGGTACCAAACATAAACAAGTGAAGCCATCAGATGAATACTTTTGGTTTGCTCGCCCAGC
TCTAGTCCTTGACTTACTTCACTTCATTTTGTTTCAAAACTCTTTTGAGCTTGCATTTTTCTTCTGGATTTGGTGTACTT
ATGGATTTGATTCATGCATTATGGAGAAAATATCCTACATAATCCCAAGACTTATAATAGGTGTAATTGTTCAAGTGCTT
TGCAGTTACAGCACCTTGCCTTTGTACACTCTCGTGACTCAGATGGGTAGCAAAATCAAGGCTGATAAGGTGTATGAATC
TCCATTGATGGGGCCTGAAGGTGGCTCCTCCAATTCAAGGCGGTCCAACCGCATGATCAAAGAATCAAACCAATGTGCTC
AAATTGATGAACAAGCAATAATCATGGTGGAAGATGCAACATCTATAATTGAGCTTCCCCACATTGTCCAATCTCCGTAT
GAGAGACCTAATGCATCCAATATTCACTAA

>CcMLO9 C.cajan_01454
ATGAGTAGTTCAAAGTTGAGAAACCTGTTCTGGGGTGTGTTGTTTTCATGGATATGGTTTGGAGGTTTGGCCATGGCGGC
AAGTGACAGTAGCAGCAGCAGCTCCAGAGACCTGGACCAGACACCAACGTGGGCCGTTGCTGCTGTGTGCACTGTTTTCA
TTTTGATATCCATAACATTGGAAAAGAGTCTCCACAAACTTGGCACGTGGCTTGGACAAAAGCATAAGAAGGCTATGCTG
GAAGCTCTGGATAAGGTCAAAGCTGAGTTGATGATTTTAGGGTTCATTTCACTTCTTTTGACTTTCGGGCAGAGCTATAT
TGTCAAAATATGCATTTCTGAAAAGATCGCATCCAAAATGTTGCCATGTCCATATAACTATGAGAGTACCGATAGTGCGT
CAGATACTGAAGAGAACCATCGTAGGAAACTTCTGTCATATGAACGTAGATATTTAGCTGCTGATACTACCTCGTACAAA
TGCAGCAAGGAGGGACATGAGCCACTTTTATCTGTCAATGGATTGCACCAGTTACACATCCTCATATTCTTCTTAGCAGT
TTTTCATGTGCTTTACAGTGCTATCACAATGCTCCTTGGAAGACTAAAGATACGTGGTTGGAAGGCATGGGAGGCAGAGA
CTTCTACTCATGATTATGAGTTCGCTCATGATGCTTCAAGATTTAGGCTTACACATGAAACATCATTTGTGAGGGCCCAT
GCAAGTTATTGGACAAGGATTTCCATCTTCTTCTATATTAGCTGCTTCTTTAGGCAGTTTTATAGGTCTGTCCGTAAGAC
TGACTTCCAGACTTTGCGCCACGGGTTTATCAATGTCCACCTGGCTCCTGGAAGTAAATTTAATTTCCAAAAGTATATCA
AAAGATCATTAGAAGATGATTTCAAGGTGGTTGTGGGAGTCAGTCCTGTCCTCTGGGCATCAGTTGTAGTTTTTCTACTC
ATCAATGTTAATGGATGGCGTACTCCAATTTGGGCAGCCTTAATCCCTATTATTATAATTTTGGCCGTTGGAACAAAACT
TCAAGCCATATTGGCCAGGATGGCTCTTGATATCACCGAAAGACATGCAGTTGTCCAAGGAATGCCTCTTGTCCAAGGCT
CAGACAAATACTTTTGGTTTGGTCAGCCACAGTTGGTTCTTCATCTTATCCATTTTGCTTTGTTCCAGAATGCGTTCCAA
GTAACATATATCTTGTGGATATGGTATTGTTTTGGGGTGAGAAACTGTTTCCGTACTGACTACAAGCTTGCCATGATAAA
AGTAGCTATAGGGGTTGTGATGCTATGCCTCTGCAGCTATATCACCCTTCCATTATATGCTCTTGTAACTCAGATGGGTT
CAAGGATGAAGAAAGCAATATTTGATGAGCAAACAAGCAAGGCTCTAAAGAAATGGCACATGGCTGTGAAAAAGAAGCAT
GGAGCAGTGAAGCTGGGAAAGTCGAGTGCACGTACCTTAGATAATGGAAGCCCCATTGGTTCAACAATGAGCTCCCCTGG
GCCCACACTGCACCGTTTCAAAACAACCGGCCACTCAACCCGCTCTTCAACGTACGAGGATCAAGACGATTATGAGTCCG
ATGATGTTGAGGTGTCTCCCGTGTCGCAAACAACTAGCTTCATTGTAAGAGTGGATCCTGGTGAGCAACATGCAGAAGAA
GAAGAAGAAGATCATAAACACCATAGTCATAGTGAGGGAGAAATCAACGATGAAGCTGAATTCACCTTCGTCAAACTCAA
ACCTCATCCTGGGCAAAGAACCACCACGTAG

>CcMLO10 C.cajan_18941
ATGTCGGGAGGAGGAGGCGGTGACGCCAGGCAGCTAGATCTCACGCCAACATGGGCTGTCGCTGCGGTTTGCGCAATCAT
CGTTATCATTTCTATACTCTTGGAAAAAATCATACACAAATTCGCTAAGATGTTTGAAGAGAAAAAAAAACATGCATTGC
TAGAAGCTCTTGAGAAGATTAAGGCCGAGCTAATGATCTTGGGATTTATTTCCCTGCTCTTGACATTTGGGCAAAACTAC
ATTTCTAAAGTGTGTATACCGGAGAGGTTTTCATACACGATGCTTCCCTGTCTTCCCGTTGAGAAGCGTGGAGGTGGTCA
TGGCGAACATGGAGCTGCACCGCATGAAGAGGAGGGGGGTGAGGCTGAAGCTGAAGAGCATCACCGCAGGCTTTTATCAT
ATGAACGTAGGTTTCTAAGTGCTGAAGCTGGAGGCCACAGTTGTGAGTTGGGGCACGTGCCTCTTATATCAGTGAACGGG
TTGCATCAGTTGCACATTTTTATATTTTTCTTGGCTGTTTTCCATGTGATATACAGTGCCATAACAATGACGCTTGGAAG
AGCAAAGATTCGTGGATGGAAGGCATGGGAACAGGACCATATCGTGGATCAAGATGCCTTGAATGATCCTAGAAGATTCA
GGCTTACTCACGAGACATCATTTGTGAGGGATCACAACAGTTTTTGGACTAAAACACCAGTTACCTTCTATTTTGTATGC
TTCTTTCGACAATTTTTCAGATCTGTTCGTAGGGCCGACTATTTGACCATGAGACATGGTTTTGTGACTGTCCATTTAGC
ACCCGGAAGTAAGTTTGATTTTCAGAAATATATCAAGAGGTCATTAGAAGATGACTTTAAGGTAGTTGTAGGAATCAGTC
CAATTCTTTGGGCATCAGTGGTGTTATTCTTGCTTGTGAATGTCCATGGATGGCATGCTTCATTCTGGGTGTCTTTTCTT
CCACTATTGGTGATTCTGGCTGTTGGAACAAAGCTTCAAGCAATTATCACTCGAATGGCACTTGACATAAAAGAAAGGCA
TGCTGTAGTGCAGGGGATCCCTCTTGTGCAAGTCTCTGACAAGTATTTTTGGTTTGCATGGCCTCAGTTAGTTCTTTATC
TCATCCATTATGTCCTCTTTCAGAATGCATTTGAGCTGACGTACTTCTGGTGGACATGGTATGAATTTGGTTGGGCTTCT
TGCTTTTACGAGGATGATAGTCTCATGATTGTCAGAGTAGCTCTTGGGATAGGAGCTCAAGTTGTTTGCAGCTATGTCAC
ACTTCCACTCTATGCACTTGTAACACAGATGGGATCAACGATGAAGAAGTCAATATTTGATGATCAAACATCGAGGGCTC
TGAAACAGTGGCATAAGAATGCTCTGAAGAAGAAAGCATCCAAGGGACGAGCGGAAACTCGAACGTTAGGTGCGAGTCCA
GGAGAGTCGCCGGATGTTTCGCCACGGCCAACGACGGCCGAAGAAACAGAAATGGCTGAGCATGGCGCCACCATTGTCAG
CACCGTCGATAACGAACAACAACAGTACAATAACCGCGACCTTCTAACTGGACCATGA

>CcMLO11 C.cajan_18939
ATGGCTGTTGCAGCTTCTGGAGAAAGAAGCTTGCAGGCAACACCCACATGGGCAGTAGCTGTTGTTTGTTCAGTTTTTGT
CATTATCTCTGTCATAATTGAACATGGAATCCATTCACTTGGAAAGTGGTTTCAAAAACGACATAAGAAGGCCATGAATG
AAGCGTTGGAGAAAATCAAAGCGGAATTAATGTTGCTAGGATTCATATCCCTATTACTTACGTTTGGTACAAAGTATGTT
GCAAAGATATGCATTCCTGAATCATTTGGTGACACCATGCTGCCATGCAAGAAGGTGAAACCAGATGATGACTCAAATGG
CAGAAGGAGACTACTTTCTTTGGACGAGAGCGTGGAATGGCGTCGAGTTTTAGAAGCTGCGTCTGGTGGTGGTGACTCTT
GCTCACAACAGGGTAAAGTGCCTTTGATATCTCAGTCAGGGGTGCATCAGTTACATATATTTATATTCGTGCTGGCTGTT

TTTCACATTTTTTACAGCGTCATGACTATGGTGCTAGCCCGAGCAAAAATGAAGAAATGGAAGGCTTGGGAAGCGGAAAC
AACCTCCCTGGAGTATCAATTTACAAATGATCCTGCAAGGTTCAGGTTTGCGCATCAAACATCATTCGTTAGGCGTCACT
CTGGTTGGTCTAGGATGCCAGGAATTCGATGGATAGTGGCATTCTTTAGGCAATTCTTTGCGTCTGTGACTAAAGTGGAT
TACATGACTATGCGGCACGGATTTATTAACGCACATTTTGCTCCCAATACCAAATTTGACTTCCAAAAATACATTAAAAG
ATCCATGGAAGACGACTTCAAAGTGGTTGTAGGCATAAGTATTCCGCTATGGGCTTTTGCAATCATCTTTCTGCTTGTCA
ACGTTTACAAATGGTACACGCTCACTTGGCTATCATTAGCGCCGCTAGTGATACTTTTATTGGTGGGCATGAAGCTGGAA
CTTATAATCATGGAAATGGCCCAAGAAATCCAAGATCGAACCACAATTGTTAGAGGAGTACCTGTTGTAGAGCCAAACAA
TAAGTACTTTTGGTTTAACAAGCCTCAGTGGATTATCTTCTTAATCCATTTTACCTTATTCGAGAACGCCTTCCAAATAG
CCTTTTTCTTGTGGACATGGTATGAGTTTAAGATCACATCTTGCTTCCATGAAAACTTGCCATTAATACTAACAAGGGTT
GTCCTTGGGATAGCCTTGCAAATTGTATGCAGTTACATAACATTTCCTCTCTATTCCCTAGTAACACAGATGGGGTCTCA
CATGAAGAAAGCAATATTTGAGGAGCAAACAGCAAAGGCACTTAAGAAATGGCAAAAGACAGCAAAGGATAAGAGGAAGT
TAAGGAAAGCAGGGATAGAAATAGCTTCAGGAGAAACAACACCAAGCCACGGGACATCACCACTGCATTTGCTTCACAAG
TCAAAGCCAAGCCACAGCCACATAGAAATGGACAGTGTTCTCTATTCTCCACGATCGTACCAATCTGATACTGACTTGTC
TGAGACAGAGGGATCCTCACACCAACAAAATGAAATAACACAAACACATCAACCTCCAAGGAATAATGGACTAACTCATG
ATATCGATTTTTCATTTGAGAAGCTTTAA

>CcMLO12 C.cajan_36621
ATGTCTGTCTCAAAGTACAAAAAGCTGTGCCTTTGTCTTGTTTCATTGTTCTGCTTTGGTTGTGTGGCTATGGCTTCTAG
TGAGAGTAGTAGTGGTTCCAAAGAGCTTGATCAGACACCAACATGGGCTGTGGCTTGTGTCTGCACTGTTTTCATCTTGA
TTTCCTTAACTCTGGAGAAAAGCCTTCACAAACTGGGAACGTGGCTACGGGAAAAGCACAAGAAGGCTTTGCTTGAGGCT
TTGGAAAAGGTCAAAGCTGAGTTGATGATTCTTGGTTTCCTTTCATTGCTTCTGACTTTCGGGCAGAGTTACATTGTTAG
AATATGTATTCCTCTCAATGTGGCAGACAAATTGTTGCCTTGTAAGTATGTTGGTACCGACAATGAATCAAGTAGTGAAG
AGGAACACCGTAGGAAGCTTCTTTCTTATGAACGCAGATATTTGTCAGATGAAGCTACCCCTTATCAATGCGGCAAGGGA
CATCAGCCACTTATATCTGTGAATGGATTGCACCAGTTACACATCCTCATATTCTTCTTAGCTGTCCTTCATGTGCTTTA
CAGTGCTGTCACAATGCTTCTCGGAAGACTAAAGATTCGAGGATGGAAGGCATGGGAGGCAGAAACTTCATCTCATGGTT
ACGAGTTTGCCAATGATCCTTCAAGATTCCGGCTTACACATGAAACATCGTTTGTGAGAGGTCATGCTTCATTTTGGACA
AGATATTCAATCTTCTTCTATATAGGATGCTTCTTTAGACAATTTTATAGGTCTGTGGGCAAGGCTGACTACTTGGCTTT
GCGCAATGGATTCATCGCTGTCCACCTGTCTCCTGGAAGTAAATTTAACTTCCAAAAGTATATCAAAAGATCCTTGGAGG
ATGACTTCAAGGTAGTTGTTGGAGTCAGTCCTGTCCTCTGGGCAGCATTTGTTTTTTTCCTGCTTCTAAATGTTTATGGA
TGGCATGCTGGGGTATGGGCATCCTTAATTCCTTTTTTTATAATTTTTGCTGTTGGAACCAAACTTCAAGCCGCATTGGC
AATGATGGCTATTGAAATCACAGAAAGACATGCTGTTGTACAAGGGATTCCTCTTGTTCAAGGCTCAGACAGATATTTTT
GGTTTGGTCAGCCTCAGTTAGTTCTTCATCTTATCCATTTTGCTTTGTTCCAGAATGCATTCCAGATAACATATTTCTTG
TGGATATGGTATTCTTTTGGGCTGAAAAACTGTTTCCATGCTGACACTGAGGTTGCAATAGTGAAAGTAGCTTTAGGGGT
TGGAGTTCTTTGCCTCTGCAGCTACATCACCCTTCCATTATATGCTCTTGTTACTCAGATGGGATCAAGAATGAAAAAAT
CAATATTTGATGAACAAACATCAAAGGCGTTAAAGAAATGGCACATGGCTGTGAAAAAGAAGCAAGGAGTGAAACTAGGG
CAATCCAGAGTGCGAGCCATGGATGGAAGCACCACAGATTCAACAGTACACTCTTCTGGCCCCACACTACACCGTTTCAA
AACCACTGGTCACTCAACTTCTCACACCATGTCAACCTACGGTGACCAAGATGATTACCATTCTGATAATGAATTGACTT
CCATTTCACCAACATCAAGCTTGATAGTAAGAGTGGACCATGATGAGCAAGAACCAGCAAAAGAAATTGAACACCACCCC
ACAGCCAACAATCAATAG

>CcMLO13 C.cajan_17246
ATGGCAGAGGTGCTGCAGCGAGGTCGCTCTCTGGCTGAGACACCAACGTATTCAGTTGCCTCTGTTGTTACTGTCATGGT
CTTTGTCTGCTTCTTGGTTGAGCGTTCAATCTACCGCTTTGGAGAGTGGTTGAGGAAAACAAGAAGGAAGGCACTTTTTG
CTTCACTGGAGAAAATTAAGGAAGAGTTGATGCTGCTTGGGCTTATCTCTTTGTTGTTGGCACAAAGTGCAAGGTGGATC
TCCGAAATTTGTGTGGATTCATCACTTTTTAGCAGTCGATTTTATATTTGTTCTGCACAAGACCTGGGCACGACTGAGAA
TATCATGCACGAAAGCTTGTCCTCTTTCCCTGATGAAACTACTATTCCAAAGGGTCTAAATAGTGGAGCATTCAACCAAT
GTGGAGCGGGCCATGAACCATTTGTTTCATATGAGGGCCTTGAGCAGCTGCACAGGTTCTTATTTGTTCTTGGGATCACT
CATGTTATGTATAGTTGTCTGGCAGTTGGTTTAGCAATGAGCAAGATCTATAGTTGGCGCAGATGGGAAAATCAGGCTAA
GATGGCAATCGATGGAAACTTGCAAGGAAAGAAAATAAAGGTTATGAGGCGGCAGACAACATTTGTTTTCCATCACACAT
CTCATCCATGGAGCAGGAGTCCAATCCTAAACTGGATGCTCTGTTTTCTACGTCAGTTTAGGAGCTCTATACAAAAATCA
GATTACCTTGCACTCCGTTTAGGTTTCATCACTGAACACAAATTGCCGTTGTCTTATAATTTTCATAAATATGTGGTTCG
AAGCATGGAAGATGAATTTCATGCCATTCTAGGAATAAGCTGGCCACTTTGGCTTTATGCCATAGTCTGCATCTTCGTGA
ACATCCATGGTCTGAATATATACTTCTGGCTATCATTTATCCCTGCAATTCTTGTCATGTTGATAGGGACAAAACTTCAA
CATGTTGTATCCACTTTAGCACTTGAAATTATGAAGCAGACAGGTCCATCTGCTAGGACACAAGTGAAGCCGCGCGATGA
TCTTTTTTGGTTCAAAAAGCCAGATATACTGTTATGGCTGATACAATTTGTCATATTTCAGAATGCCTTTGAAATGGCAA
CATTTATTTGGACATTGTGGGGATTCCAAGAACAATCGTGCTTCACGAGAAACCATTACATGATCATCACTCGGTTGGCT
TCTGGTGCTCTTGTTCAATTCTGGTGTAGCTATATGACAGTGCCCCTGAATATTATAGTTTCTCAGATGGGATCTCGATG
TAAGAAGGCCTTAGTAGCTGAGAGTGTTCGAGAGTCTCTGCATAGTTGGTGTAAAAGAGTAAAACACAAGTCTAAGCATG
ACTCGGTGCATTCACACACCGCAAGATCAGTGTGTTCCCTCGAATCAACAATTGATGAGAGGGATGAAATAACAGTTGTA
TCCGGTACTCTAACTCGAAGTTCATCCTTAGAGTCTTTGAATCAGATAACAGTGACTTCAGTAGACCAGCTGAACTTTGA
GACTCCCAACAATCCAAAAGATTCAATTAAGGTGACCGAGTATTTGTCTGAGTCTGTGTATACTAATACTAATAATCCCC
CACAACCGTCATACAACGGGGAAGACCATGTTGGCAATGGAGAGGAAGCAAAAGTTGACACTCTTCTTGATTTGTTTCAG
AAGACATGA

>CcMLO14 C.cajan_23778
ATGAGTGGTGGAGGAGGAGAAGAGGGAGATACTCTGGAGTTCACTCCGACATGGGTAGTGGCCGCCGTTTGCACAGTGAT
TGTGGCCGTTTCCCTGGCCGCCGAGCGCTTCCTCCATTACGGAGGAAAGTTTCTGAAAGCCAAGGGCCAGAAGCCGCTCT
TCGAAGCTCTCCAGAAGATCAAAGAAGAGCTGATGCTGTTGGGTTTCATTTCCCTACTTCTGACGGTTACACAAAACGGC
ATTACCAAAATCTGCGTTCCACCCGGTTTGACGCGCCACATGCTTCCGTGTGACCCCAACCAAGCTCCAAACCACGAATC
TCATTTCCAGTATCATTCGTTTTTCCCTGGAACAGCCAGACGCCTTCTCGCCGAGGCCCACTCCGAGTCCGAGACTCAGA
CTGGTTACTGCGCTCGCAAGCACAAGGTACCTCTGTTATCTTTGGAAGCACTGCATCACCTCCACATCTTCATTTTTGTC
TTGGCTATCGTGCATGTCACCTTTTCCCTACTCACCGTTGTCTTTGGAGGCGCCAGAATACGTCAGTGGAAACACTGGGA
AGATTCAATCGCAAAACAGAATTACGAGACAGCCCGAGTTCTGAAACCGAAGGTGACTAATGTTCAACAGCATGATTTTA
TCAGGGGTCGTTTTGCTGGTTTCGGCAAAGACTCTGCCATCGTTGGTTGGTTGGTATCCTTTTCAAAACAATTTTATGGG
TCTGTGACAAAATCAGATTATGTGACATTACGGCATGGTTTCATCATGACCCACTGCAAGTCGAACCCAAAGTTTAATTT
TCACAAGTACATGATCCGTGCCCTCGAAGATGATTTCAAGCAAGTTGTTGGTATAAGTTGGTATCTGTGGCTCTTTGTGG
TTATCTTCTTGTTGCTTAATATCAATGGTTGGCATACATATTTCTGGATTGCTTTTATTCCCGTCATCCTTTTACTAGCT
GTGGGCACTAAGCTGGAGCATGTAATAACCCAACTAGCTCATGAAGTAGCTGAAAAGCATGCAGCCATAGAAGGTGACTT
AGTTGTGCAGCCATCAGATGAACACTTTTGGTTTCATCGGCCCCGAGTCGTCCTCTTCTTGATTCACTTTATCCTTTTTC
AAAATGCTTTTGAAATAGCATTTTTTTTCTGGATATGGGTTACATATGGATTTGACTCCTGTATAATGGGACAAGTTCGT
TACATTGTTCCAAGGCTCGTTATTGGGGTATTTATTCAGGTACTATGTAGCTACAGCACCTTGCCACTCTATGCAATTGT
TACGCAGATGGGAACTCACTACAAGAGGGCAATATTCAATGAGCATTTGCAACAAAACATTGTTGGTTGGGCACAAAAGG
CAAAGAGGAGGAAAGGACTAAAAGCTGAGACTGAGAGCCATTCTGGCCAAGGAAGTGCTCATGAGAGTTCTGGTGCTGGT
GCTGGTGCTGGTTCTGCTACAGGAATCCAGCTTGGGTCAATTTTCAAGAAGGCATCTGCGCCAGGAGACAGTACCTCTGT
CCCCAAAGCCGATGGTAACTGA

>CcMLO15 C.cajan_47280
ATGGGTGGTGGTGGAGCTGAAGAAGGGAACAATTTGGAATTCACTCCCACTTGGGTTGTTGCTGTTGTTTGCTCTGTCAT
CGTTGCTGTTTCATTTGCCGCTGAGCGTTTTATTCACTATGGAGGGATTTTTCTCAAGAAGAAGAATCAGAAGCCACTCT
TTGAAGCTCTGCAAAAGATCAAAGAAGAGTTGATGCTGTTGGGATTTATTTCTCTGCTACTGACAGTAACACAAAATGGG
ATAGTCAAAATTTGTGTTCCAGTGAGTTGGACTCACCATATGCTTCCCTGCAGCTTCGATCTTAAAGAAAAGGAGGAATC
AACAAAAACCACATCACATTTTCAGACTTTTTTTTCTTCCTCTAATATTTCTGGCATAGCTAGACGCCTTTTAGAAGAGA
GTAAGAGTGAGAGTGAGAATCACGAACCATCAACAGTGACAAAACTTGGACACTGTGCTGCCAAGGGCAAGGTTCCTCTG
TTATCCGTGGAGGCATTGCATCACTTGCATATCTTTATTTTTGTCCTAGCTATCGTCCATGTAGCATTTTGTGTTCTCAC
TGTAGTTATTGGAGGGTTACAAATTCGTCAGTGGAAGCACTGGGAAGACTCTATTGTAAAGGAAAATTATGAAACACAAC
CAGTTCTTGAACCAACAGTGACTCATGTTCAAGAACACGCTTTCATTCAGAATCGTTTTACTGGTTTTGGCAAAGATTCT
GCTGTTCTAGGTTGGGTGAAATCATTTTTCAAGCAATTTTATGGATCTGTGACAAAATTAGATTATGTGACATTGAGGCT
TGGTTTCATTATGACCCACTGCAGAGGAAATCCAAAGTTTAATTTTCATAAATACATGATTCGTGCCCTTGAAGATGATT
TCAAAAAAGTTGTTGGTATAAGTTGGTATCTTTGGATATTTGTGGTCATCTTCTTGTTGCTTAATATCAATGGTTGGCAC
ACATATTTCTGGATTGCTTTCATTCCTGTCATTCTTCTACTTGCTGTGGGTACAAAGTTGGAGCATGTAATAATTCAATT
GGCTCATGAAGTAGCTGAGAAGCATTCAGCCATAGAAGGTGAATTAGTTGTTCAACCAAGAGATGATCACTTTTGGTTCA
AACGACCCCACATTGTCCTCTTCTTGATTCACTTCATCCTTTTCCAAAATGCTTTTGAGATAGCATTTTTCTTCTGGATA
TGGGTCACATATGGCTTTGACTCCTGTATAATGGGAAAAGTGCGTTACATTGTTCCAAGACTCGTTATTGGGGTATTTAT
TCAAATACTATGTAGCTACAGCACCCTACCACTTTATGCAATCGTTACACAGATGGGAACTCACTTTAAGAAGGCTATAT
TTGATGACCAAGTGCAAGCACGACTTGTTGGTTGGGCACAAAAAGCAAAGAAAAAAGGATTAAGAGGTGATAACAATGGA
CAAGGAAGTTCTCATGCAGGTGCTGGAATTCAACTAGGACCAATGTTTAGGAGAGCTTCTGCCACAGAAGACAACGTTGT
TGTTCCCAGAAATGATGGGTCTGAATGA

>CcMLO16 C.cajan_13783
ATGGACATAGGAAAAGAAAGAACTCTTGAAGAAACGCCAACATGGGCTGTCTCAGTATTTTGCTTCTTCTTTCTTACAAT
ATCTTTGATCATTGAGGGTGGTCTTCACAAATTGACTGAGATTTTAAGGAAGAGGAAAGGAAAATCCTTGGGGAAGGCTT
TGACCAAAACCAAAACAGAAATGATGAAATTCGGTTTCATGTCCTTTCTTCTCACAATATCGGAAGTGCCCATATCCAAA
ATCTGCATCACCAAGGATGTGGCAAATTCTCTTCTACCATGTAAAGATGCTGTGGACTTAACAGGATTAACTTCAGCACC
AGCTACTCGCACTTCAACCTCTGAATTTGACATAGCACCTGCTACCAACGAATCCACAATTGAGGTCAACTATTGTGAAG
CAAAGGGAATGGTTAGTTTAATTTCAAGTGATGGAATTCTGCAGCTAAATATATTCATCTCGTTTTTGGCGGTGTTTCAC
ATCTTGTTCTGCACATTGACAATGTGCCTTGGTAAGGCAAAGATGAGAAGATGGAAAAGATGGGAGGACGAGACTCGAAC
ACTTGAATATCAGATAGCTAATGATCCAAGAAGATTCCAGTATACAGGTCAAACATTGATTGGCAAGCGTCATTTGAAAT
TCTGGAGCTACCATTCTCCTTTGCTTTGGATGGTTTGCTTTTTTCGGCAGTTCTATGGTTCAGTCTCAAAAGATGATTAC
TTTACTCTACGAAATGGGTTTATTGCAGCTAATATTTCTTTAGGCAGCAATTTCAACTTCAAGAAATTTCTTTCTCGAAC
TTATGATGAAGATTTTGAGAAGGTTATGGGAATCAGGATTTGGATCTGGATTTTCTCCATACTTTTCATATTTTTCAGCG
CACATGAGTTCTACAATTACTACTGGCTTCCCTTTATTCCACTTGTGGTTGCTTTACTTGCTGGGGCAAAACTTCAAGTC
ATAATAACAAAGATGTGTGTAGATAGTTGCAAGGAAAACCCAGTGATCAAAGGAAGTCTACTTGTAACACCTAGTGATGC
ACATTTCTGGTTTCATAGACCTGAATGGCTTCTCCATTTACTCAAATTCATCCTTATCCAGAACTCCTTTCAACTTGCAT
TCTTCACCTGGACTTGGTATGAATTTGGTCCAAGGTCTTGCTTCAATCGAGAAAAAGAGGACATTGCCATAAGGATTGCC
ATGGGTGTAGCTGTGCAGCTCCTTTGTGGTTATGTGACACTGCCCCTTTATGCACTTGTTACTCAGATGGGTTCTAGCAT
GAGGAGAGAAGTTTTCACTGAGAGTGTCTCCAAGGGCCTCAAAAATTGGCACAAAAAGGCTAAACAATCTCTGTGTAAAA
ACAACTCAACTTCAAGCAAACACTCAAATTCTTTGCATTCCACAGTAGGTGAGAGTTCAATAAGAGGAGCAGTGGATAGT
GTGCATAACCCAGATAATGTAGCAGTCACTAGCTATTCTTCTCCCCTCACCTCAGAGGTGGAAAAGAGAATTGCACCAAC

CCATGAGCAAGAGATGTCAAGTTCAGAAATCCCAACTATTGAGGAAGAGAATAACCCCAAGATCATCACCAGAGGAACCT
ATGACGGTGAGATCTCATTTGGGAGCAGCTGGAAGAATGTGGGAAGTAGCAGGGGAATTGGAGAATTTGGTTCAATAACT
GAAGAAGATGATGCTGAAAAATTGTCTGAGTTGAATCCATAA

>CcMLO17 C.cajan_37300
ATGGAAGAGAATGTACAAGAAACACGGTCGTTGGCCTTGACTCCTACATGGTCTGTTGCTACTGTGTTGACTGTCTTTGT
TGCTGTATCTCTACTTGTGGAGCGCTCCATTCACCGTTTAAGCAATTGGCTACGTAAAACTAATCGAAAACCATTGCTTG
CTGCTTTAGAGAAGATGAAAGAAGAGTTGATGTTACTCGGATTTATATCACTCTTATTAACAGCTACCTCACGCATGATA
GCCAATATTTGCATTCCTTCAAAGTTCTACAATAGTGCTTTTGCCCCATGCACTAGATCTGAGATTGATGAACAGATGGA
AGAGAATGGCTCTGAAGAGCGTAAATTGTTGATGGCTTCTGTTTATCCACACTTATTTAGGAGGGTGTTGAATGGAATAA
ATAGAAGCTCCTGCAAAGAGGGTTATGAACCATTTGTTTCATATGAAGGTCTAGAGCAGTTGCACCGGTTCATTTTTGTC
ATGGCGGTAACGCATATATCTTACAGTTGCCTAACAATGTTGTTGGCCATTGTGAAGATTCACAGTTGGAGAGTGTGGGA
AGATGAGGCTCACATGGATCGGCATAATTCATTAACTGAAATTACAAAAGAGTTGACTATGCGAAGGCAATCGACCTTTG
TCAAGTCTCATGCATCAAATCCATTGGTCAAAAATGGTTCCCTCATTTGGGTGACATGCTTTTTCCGACAATTTGGACGT
TCTGTGGTTCGTGCTGATTACCTTACGCTTCGCAAAGGCTTCATCATGAATCACAACCTTTCATTAAAATATGATTTTCA
CAGCTACATGGTTCGATCTATGGAAGAGGAATTCCAAAGGATAGTTGGTGTGAGTGGCCCACTCTGGGGATTTGTTGTTG
CCTTCATGCTTTTTAACATTAAAGGATCTAATCTTTATTTCTGGATAGCTATCATTCCTGTATCTCTTGTGCTCTTGGTG
GGAACAAAGCTACAGCATGTTATTGCAACCTTGGCATTGGAGAATGCTGGAATTACAGGGTTCTTCTCAGAAGCAAAGTT
GAGGCCTCGTGATGAACTTTTCTGGTTTAACAAGCCTGAACTATTGCTGTCCTTGATCCATTTCATTCTCTTCCAGAATG
CATTTGAGCTGGCTTCGTTCTTTTGGTTTTGGTGGCAGTTCGGATATAATTCCTGCTTTATTAGGAATCACCTTCTCCTT
TATTTAAGGCTAATCTTGGGGTTTGCAGGACAGTTTCTTTGCAGCTACAGCACCTTGCCACTCTATGCACTTGTTACACA
GATGGGAACAAACTATAAGGCAGCACTAATCCCTCAGAGGATAAGAGAAACAATTCATGGATGGGGTAAGGCAGCTAGGA
GGAAGAGGAGACATGGTATGTTCACTGATGACTCCACCATTCATACAGACACAAGCACTGTGTTGTCTATCGAAGAAGAT
GATCAATTAATTGATACCCCTGGAGACCCTGTCACTGGCCCTGAAGTAGAATTGCAACCAGTAACAACTGTTACATCTTC
CCCTTCTCCCATTGCTAATGAAACTTCAAGTAGGGCTGTTACACCCCTCCTAAGACCCTCTGCCTCAATATCTTCAGTGC
AATGCAGTTCCAAAACGGAAGCAATTCCCAGATGCTCCTCTATGCCAAGTGGAAGATAA

>CcMLO18 C.cajan_37291
ATGGAGGAGAATGAACAAGAATCACGACCATTGGCTTTTACTCCAACATGGTCTATTGCTATTGTCTTAACATTTTTCCT
TGCTTTTTCCTTGCTTCTAGAGCGTGCCATTCACCATTTAAGCGATTGGTTGAAGAAAACGAATCAAAAGCCCTTTCTTG
CAGCATTGGAAAAAATGCAAGAAGAGATGCAGCTGCTTGGATTTGTATCACTTCTATTAATAGCTACCTCAGACATGATA
GCAAATATTTGCATTCCAGAGAAGTTCTACAATGATGCTTTTGCTCCATGTACTAGATCCGAGATTGCTGAAGAAACGGA
AAAGAATGACACAGAAGAGCATAAATTGTTGATGGCTTCCACTAACCCACGCGTAATTAGGAGAATATTGGATGACATAA
ATAAAAAATCTTGCAATGAGGGTTATGAACCTTTTGTTTCACATGAAGGTCTAGAGCAATTGCACCGTTACATTTTTATC
ATAGCTGTAACACACATATCTTTCAGTTGTCTCACAATGTTGTTAGCAATTGTGAAGATTCACAGTTGGAGAACGTGGGA
GAATGAGGCTCAAATAGATCCACACAATTGTTCATTTTCTGAAATCCCAAGACATGTGCCAGCACTAAGTCAAACATCCT
TAATTATGGTTCATAGATCAAATCCATTGTTCAGAAACACTTTCCTCATTTGGGTGGCTTGTTTTTTACGACAATTTTGG
AATTCTTTGGTTCGCAGTAATTATCTTACACTTCGCAAAGGCTTCATCATGGTATCCAATTCTGTTTGTCTGAAGTCTAA
ATGTTTCTTATTCGAAAGCAACATAATATGCACTACTAGAAAAATTAAAATTTGTGACGAAAATTTTGTCCCACTCTGGG
GATTTGTTGTTGCTCTCATGCTATTCAACATTAAAGGATCAAATCTCTATTTCTGGATAGCTATCATTCCAATTTTTCTA
GTTCTTTTAGTTGGAACAAAGCTACAGCATGTTGTTGTAACCTTGGTATTAGAGAATGCTGAAATAACTGATTTCTTCTC
TGGAGCAAAGTTGACACCTCGTGATGAACTTTTCTGGTTTAAGAAGCCCGAACTACTACTTTCCTTGATTCATTTCATTC
TCTTTCAGAATGCATTTGAGTTGGCTACATTCTGTTGGTCCTGGTGGGAGTTTGGAAACAGCTCTTGCTTTAACAGGAAT
CATCTCCTCGTGTATTCGAGGCTAATCTTGGGGTTCATTGGACAGTTTATCTGCAGCTACAGTACTTTGCCCCTCTATGC
ACTTGTTACACATATGGGAACAAACTATAAGGCAGCATTAATTCCAGAGAGTGTAAGGGCGACAATTGATGTCTGGGGGA
AGGCAGCAAAGAGAAACAGAAAGCAAGGCAAGGTCACTGATGACTTTGAATTAGAGCCACTAAGGGAAGAAACTTCAGCA
AATAATGTTTCAAGTAGGCCAGTTGACCCCGTCCTTCGACCCTCTGCTTCAGTTAACTCAGAACTCCGTTTTAGAACAGA
AACAATTCCAAGATCTACTTCTATGCCAAGAGAAATATAA

>CcMLO19 C.cajan_09813
ATGGCAGCATATGCATCTGGTTATTCTCTAGAACACACTCCAACATGGGCAATTGCTCTTGTTTCCTTCATTCTTATCTC
CATCTCAATCATCTTGGAGCATCTCATTCATCTCATAATCAAATGGTTGAGGAAACATCGGAAGATAGTTGAAGCAATAG
AGAGGCTTAAATCAGAGTTGATGATCTTAGGATTCATGTCGCTTTTGTTGACAGTAACTCAAGATGCCATAATTGAAATC
TGCATACCAATGAAGACTGCAGATACTATGCTTCCATGTCGTAAACGTACTACAAATGATACCGCAACAAATTTGGATTC
TTGCAGTGCCAAAAATGCAAGTAAGGTTGCTTTAGTATCAAAACATGGAATTCATCAACTTCATATGTTCGTTTTTGTTC
TAGCTGTCATGCAAATCGTGTACAGTTTTCTCACCGTTTCTCTGGCAAGGGCCAAGATGAGGCACTGGAAAGCTTGGGAT
GAAGAGACTCAAACAATCGAATATGAAATTGCCAACGACCCTAATAGATTCAGATATTCGAGGCAAACAACATTCGGAAG
ACGACACATATCTACTCGTACGGCATCACCAGTATATGTTTGGATTAAGTGTTTTTTCAGACAGTTCTTCCATTCAGTAG
AAAAAGTTGATTATCTCACCCTGAGGCATGGTTTCATATCAGCACACTTTTCAGCTAGGAACAGCAACTTCGATTTCCAA
AATTACATTGAACAGTCACTTGAGGAAGATTTCAGAATCATAGTGAGCATTAGCCCTGTGATGTGGTTCACCGTTGTTAT
TTTTTTGCTCGTGGATGTACATGGCTGGCATGTATACCTCTGGTTATCCTATGTTCCCCTCATGCTGGTTCTGGTTGTGG
GAGCTAAACTTGAAGTCATTGTCGACGAAATGGCTCTTCAAATGAAGGATATCAATAATGTAACCAAAGGGACCCCACTT
GTTTGTCCAAGTGACAAACTCTTCTGGTTCGGCCATCCAGGATTTGTGTTAACACTCATACATTACACTTTATTTGTGAA
TGCATTCGAGCTTGCCTTCTTTATATGGGTATCAACACAGTTTGGAATAAATTCTTGCTACCACGAACACAGATCATTTA

CAATCATAAGAGTGGTGATAGCGGTCGCAGTTCAAGTGCTATGCAGCTACGTTACTCTCCCCCTCTATGCATTAGTGGCG
CAGATGGGTTCACAAGTGAAGAGCAAAGCGTTGGCTAAAATTCTGAAGCAGTGGCATGTTGAGGTGAGGGAGAGAAGGAG
GAAGCAAGAAAAGCTTGTGAAGTCTTTTAGTTTTAGGCACAGAAATAAGTCCTCAGAATGGGGCCAAGGAAATAAGAGTG
CCCCTGAGTTTTCTTCCACTCTAGGTGAAAGCAATCGTTCCTATGACGAAGGAGAAATTGTAGAAGAATTGGAGCATTCG
GTGAAAACCAAGGCTAGCTCGTCAAGTGATCCACCTAGTTTTGTCTAA

>CcMLO20 C.cajan_24758
ATGAAGCAGCCCAACATCTCTAATTGCCCTCTTATCGTACATATGGCGAATGTTTCAGAGGGAACTTTAGAAAACACACC
AACATGGGCTGTGACAACAGTTTGCTTCGTATTGCTAGTAATCTCTATTTCCATTGAGCAGTTGCTCCATCTTTTGGCCA
GGTATTTCAAAAGGAAGAGGAGGAAGTCACTCACTAAGGCCCTTGAAAAGATCGAATCAGAATTGATGCTTCTGGGTTTC
ATGTCTCTGCTGCTAACGGTGAGTGAAAAACGGATTGCAAATATTTGCATAACAAAGAGTGTTGGTGAAACTCTTCTTCC
CTGCGCAACCATGACTTTTGACGTGACCAAGGAAGAAACAAAATGTGCCGAGCAGGGCAAAGTATCATTGGTGTCGAGGA
AAGGTGTCCGAGAGTTGCAGTACTTCATCTTTTATCTGGCTTTGTGTCATGTTATTTCTTGCATTCTCACCTTTAGTCTT
GGAATGGCGAAGATGAGACGATGGGAGTCTTGGGAAGCAGAAACTAGAACATTGGAATATCAGTTTCAATATGATCCACG
GAGGTTTCAGCTCACACGTGAGACATCATTTGGGAAGCGGCATCTTAACTATTGGAGTAACAACCCTATACTGTTTTGGC
CTGTCTGTCTTGTTAGACAATTTTATAGATCTGTCCCCAAGGTTGATTACTTCACTCTTCGACGTGGATTCATCACGGCC
CATTTTGCAGAAGGAAGTAACTTTGACTTCCAAAATTTTATTAAACGGGCTTTGGAGAAAGATTTTGGAGTGGTAATGGG
AATAAGCTGGTGGATTTGGATCTTCTCTGTACTGTACATATTCTTGAATGCAAATGTATTCTACAGCCATTTCTGGTTAC
CGTTTAGTCCGCTAATGGTGCTTTTGTTGGTGGGAACAAAGCTACAAGGCATTGTAACACATATGTGTTTGGATAGTGAT
GACAAATCCCAAATAATCAAAGGAAGTTTGCTTGTGAGGCCCAGTGACCACTTTTTCTGGTTTGGATGGCCCAATCTGCT
GCTTCATCTCATTAGCTTCGTATTGTTTCAGAATTCCTTCCAAGTAGCATTTTTCACATGGACTTCGATCCGATTCGGGA
TCAGATCATGTTTTCATCGAGGAATTGAGAATATCATCATAAGGGTAGCAATGTGTGTTTCGGTACAAATCCTCTGTGGC
TATGTAACACTACCTCTCTATGCTTTGGTAACTCAGATGGGTACCTCAATGAGCAAGGATGTGTTTGCGGAGAATGTGAT
TAGAGGCATCCACATTTGGCGTGAGAAGGCGAAAAAGAATGTGGCTCATAGGAATCCACATTCACAAGACTCATCTTTAG
ACACTTCCCTTTCGCTTGAAATTTCACTAGAGTCACCTTCTTTCAGGTTTGATGTATCCACCTCCATTTTAATGGATCGT
TCATCAGATGATAATAAGCACGTGGCTGTTACGGAAGAAAATATATCGAGTGAACAACTAGGTTCATTTCAAGGATTTCA
TCTGCAAAACACAGGAAAACCAAGGCAATTTTGA

>LaMLO1 AOCW01137726_AOCW01137727
ATGGCTGAAACCAGAATTTATGAAAGAACGTTAGAGGAAACTCCAACATGGGCTGTTGCAGTTGTTTGCTTCGTGCTGCT
TTTTATTTCAATCATCATTGAATATAGTATTCATGCTGTTGGAAAGTGGTTCAAAAAGAAGCACAAGAAAGCTCTCTATG
AAGCGTTGGAAAAGGTCAAAGGAGAGCTTATGCTGCTAGGATTCTTATCCTTGCTCCTAACAGTGTTACAAGATCCAATA
TCTAAGATATGTATATCAAAAAATGTTGCATCTACTTGGCATCCTTGTTCTACTCCAAAGGCAAGTAAAACTGAAGAAGA
TTCTCATTCTGATGAAAATGATACTAATTCCAGAAAGCTCCTCCAATATTTGAACCCCATGCCACGGCGTTTTCTAGCTA
CGAAAGGATATGATAAATGCGCTGCAAAGGGAAAAGTTGCTTTTGTTTCTGCATATGGGGCTCACCAGCTCCATATATTC
ATCTTCGTGCTAGCCATTTTTCATATCCTACAATGCATTATAACTCTGGCTTTGGGAAGAACTAAGATGAGACGCTGGAA
GACATGGGAAAATGAAACAAGGACTATTGAACATCAGTATTATAATGATCCTGAAAGGTTCAGGTTTGCGAGGGACACAA
CATTTGGACGAAGACACTTGAAAAAATGGAGTGAGTCATCAATTTCTTTATGGATGGTTAGCTTCTTCAGACAATTCATT
GGATCAATTAATAAAGCTGATTATTTTGCATTAAGGCACGGATTTATCATGACACATTTGGCACCAGGAAGTGATGCAAA
ATTTGATTTCCAGAAGTATATCAATAGATCACTTGAAGAGGATTTTAATGTTGTGGTGGGAATAACTCCACTCATATGGT
TCTGTGCTGTGTTATTCCTACTGACTAATACTCATGGGTGGTATTCTCATTTTTGGCTTCCATTTATCCCATTCATCATA
GTCTTATTGGTTGGTACTAAGTTACAAATGATCATAACAAAGATGGGACTGAGGATTCAAGATACAGGAGAAGTGGTTAG
GGGTGCACCTTTGGTTCAGCCAGGAGATGAGTTGTTCTGGTTCAATCGTCCACGCTTCCTTCTCTTTTTAATTCATCTTG
CTCTCTTTCAGAATGCATTCCAATTGGCATTTTTTACTTGGAGTACTTATGAATTTGGCTTACACTCTTGCTTCCATGAT
ACTACTGCAGATATTGTCATCAGAGTAACAATGGGGGTTCTGGTACAATTCTTATGCAGCTATGTAACTTTGCCTCTTTA
TGCTCTAGTCACACAGATGGGTTCAACAATGAAACACACAATTTTCAATGATAGAGTAGCAACAGCACTAAAGAACTGGC
ACCACACAGCCAAAAAGCATGTAAAAGACAGCAAGCATGCTAGTGCCAATGTCACACCATTCTCAAGCCGGCCAACCACC
CCGACACATGGCTTATCTCCGGTACACCTGCTGCAACAGCATCACGGCCGCAGTGATAGCACACAAACCTCTCCAAGGAC
CTCAAACTATGAAAATGAAGAGTGGGATATTGAAGGGTCAGCTTCCCCAACCAAACATATTATAGGAGAAGGAGATGAGA
CACAAATTTTGGAGCAAGATTCAACAACACAATTGCCTATTAGCTCTCAACATGAAATCAGCATAAGTGTATCAGAATTT
TCTTTTGAGAAGCGGCTTCATAGAGATTATGAGTAG

>LaMLO2 AOCW01065981_AOCW01147792
ATGGGTGAAAAAGTTTATGAACGAACGCTTGAGGAAACACCAACATGGGCTGTTGCAGTTGTTTGCTTTGTACTGCTTTT
TATTTCAATCATCATTGAATATATTATTCATGCTATTGGAAAGTGGTTCAAAAAAAACCACAAGAAAGCTCTTTATGAAG
CATTGGAAAAGGTGAAAGGAGAGCTTATGCTGCTAGGGTTCTTATCCTTGCTCCTAACAGTGTTCCAAGAACCAATTTCT
AAGATCTGTGTATCAAAAAATGTTGCATCTAGTTGGAATCCTTGTTCTACTCCAAAGGCTAGTAAAACTGAGAATCATTC
TGATAATACCAATTCTAGGAAGCTCCTTCAATATTTGGAACCCATGCATAGGCGAATTCTAGCTACAAAAGGATATGATA
AATGTGCTGAAAAGGGAAAAGTTGCTTTTGTTTCTGCATATGGGATTCACCAGCTCCATATATTCATCTTTGTGTTAGCT
ATTTTTCATATCCTACAATGCATTATAACATTAACTTTGGGAAGAACTAAGATGAGAAGGTGGAAAACATGGGAAGATGA
GACAAGAACTATTGAATATCAGTACTACAATGATCCTGAGAGGTTCAGATTTGCGAGGGACACAACATTTGGACGAAGGC
ACTTAAATAAATGGAGCGAGTCATCAATTTCCTTATGGATGGTTTGCTTCTTCAGACAATTCATTGGATCAATCAGTAAA
GTTGACTATTTTGCATTAAGGCATGGATTTATCATGGCACATCTGGCACCAGGAAATGATGCAAAATTTGATTTCCAGAA
GTATATCAATAGATCACTTGAAGAGGACTTTAATGTTGTGGTGGGGATAACTCCAATCATATGGTTCTTTACTGTGTTAT

TTCTGCTGACTAATACTCATGGGTGGTATTCTCAATTCTGGCTACCATTCATCCCATTCATTATAGTCCTATTAGTTGGT
GCTAAGCTACAAATGATCATAACAAGGATGGGACTGAAGATTCAAGATAGAGGAGAGGTGGTGAGGGGTGCACCTCTGGT
TCAGCCAGGAGATGAGTTGTTTTGGTTCAATCGACCGAGGTTCCTTCTCTTTTTGATTCATCTTGTTCTCTTTCAGAATG
CATTCCAACTAGCATTTTTTACTTGGACTACATATGAATTCACTTTGTACTCTTGCTTCCATGATACTACTGCAGATATT
GTCATAAGAGTAACAATGGGGGTTGTGGTACAAATTCTATGCAGCTATGTGACTTTACCACTTTATGCTCTAGTCACACA
GATGGGTTCAACCATGAAACACACAATTTTCAATGATAGAGTAGCAACAGCACTGAAGAGCTGGCACCACACAGCCAAAA
AGCATGTCAAAGACAGCAAGCATGCTAGTGCTAGTGTCACACCATTTTCAAGCAGGCCAGCAACCCCAACACACGGGCTT
ATCTCCGGTACACCTACTGTACAGGCACCACGGCCCAGTGATAGTACACAAACTTCTCCAAGGGCATCAAACTATGAAAA
TGAAGAGTGGGGTATTGAAGGGTCAGCTTCCCCAAACAACCATAATACTATAAACATAGGTGAAGGATATGAGACTCAAA
TGCAAGTTCTGGAGCCAGATTCAACAACAGAATTGCCTATTAGCTCTGAACATGAAATAAGCTTAAGTGTATCAGATTTT
TCTTTTGAGAAGCAGCACATTGGTAGAGATTATGAGTAG

>LaMLO3 AOCW01159416
ATGGAAGAAAATGGAAAAAGAAGATCCTTGGATGCTACCCCCACTTGGGCTGTAGCAACTGTCATCACTCTAATGGTTTC
TCTTAGTTTCATGTTTCAAATCACATTAGAAAAATTTGGAAAGTGGTTGGACAGAACTAAGAGAAAATCTATGCTTTCTG
CTCTTGAAAAGGTCAAAGAAGAACTGATGCTTTTTGGGCTGCTTTCACTATTGATGGGTCATTGGACTATTTTTGTGGCA
AAGATTTGTGTCAAGGAATCAGTATTAAAAACCAGATTTATTCCATGTGCAATAGAAAAGAATTCTGGAACAGTGGAACA
CATTTTCTGGCTGAGTTCAGAATATTCAAATAGAACAATGCTAGAAGAAAATGTGAACAAGGGTCTGCATAATTATTGTC
CTGAGGGTAAGGAATCATTCGCATCTTATGAAAGCCTTGAGCAGCTTCATTCGTTACTGTTTGTCCTTGGGGTCACTCAT
GTTTTCTATAGCTTTATTGCTGTTGCCTTGGCCATGATCAAGATATATAGCTGGAGAATATGGGAAAGTGATGCAAAGAC
TATGGCTACACAAAGCTTCCAAGATACTCCACAAAATACATTAAACATAAGGTTGAGTCGACTCGGTACATTCATCTTTC
ACCACACATCTCATCCCTGGAGTCATCACAAAATTCTTGTTTGGCTGCTATGTTTCAGTCGTCAATTCTGGAGTTCTATC
AACCGAGCTGATTATATGGCATTGCGCTTTGGCTTCATTACCAATCATGAGCTTCCTCTAACATATGATTTCCACAACTA
TATGCTTCGTAGCATGGATGAAGAGTTTCGTGACATTGTTGGTATTAGCGTGCCACTATGGATATATGCCATATGCTGCA
TTATTCTAAATTTTCATGGAAGCAATATTTACTTTTGGCTTTCCTTCCTTCCATCAATTCTGATTCTCATGATTGGTACT
AAGTTGCACCGTGTGGTGGTAAAGTTAGCTGTTGAAATAACGAGTCATTGCCAAGACACAAGACCTCTTAACTTAAGAGA
TGAGCTCTTTTGGTTTGGAAAACCCAAACTTCTATTGCGGTTAATACATTTGATATCATTCTTGAATGCTTTTGAAATGG
CAACATTCTTATGGTCCCTGTGGGAAATTAAAGAGGCTTCTTGTTTTATGAAGAACCGAACGTTTATTATGATCCGTTTG
TCATTTGGGGTCATCTCCCAAGTCTGGGGCAGCTTTATAACATTTCCTCTCTATGTTATCATCACACAGATGGGGTCAAA
GATGAAGAAGTCTGTGATATCTGAAAATGTAAGAAAATCACTCTCTAAATGGCAGGGAAGAGTGAAGAACAGGCAAGGTT
CCTCTTTGATGCATGACTATGAATCATTTCCAAGTTGTGAAGATTATCATCATCATAATGTATAAG

>LaMLO4 AOCW01114694_AOCW01136624
ATGGCAGGAGGAATCGGAGGAAGAAACTTAGAGCAAACTCCTACTTGGGCCGTCGCAGTTGTTTGTTTTGTTTTGATTTC
AATTTCTATAACCATCGAGCACACCATTCATCTCATAGGAAAGTGGTTGACGAAGAAGAACAAAAGATCTCTGTACGAGT
CACTTGAAAAGATAAAATCAGAGCTTATGTTGTTAGGTTTCATATCGTTGCTCCTAACAATAGGACAAAGTCTAATATCA
AAGATATGTATATCAGAAAAAGTTGCGCGTACATGGCATCCATGTAGCGATAAAGAGGACCACACGAGTTCATCAAAAAA
CGAAACTAATCAACGGAGATTAATAGCGACGACGTTGTTCTTTGGTTTGGAGAGTGAAAATCCACGACGAGTTTTAGCGG
GAGGAGGAAGCGATAAATGTGGAAAGGGTAAAGTCCCGTTTGTGTCAACGGATGGTATTCATCAACTCCATAGATTTATC
TTCGTGCTGGCTGGTTTTCACGTCCTTTACTGCATTCTCACTTTGACTCTTGGAAGAGCAAAGATGAGGAGGTGGAAGCA
ATGGGAAGAGGAAACTAAGACACTTGAGTACCAATTTTCACATGATCCTGAGAGATTCAGATTTGCAAGGGACACATCGT
TTGGGAGAAGACACTTGAGTTTCTGGACCAAAAATCCTCTTCTCATTTGGATTGTTTGTTTCTTCAGGCAATTTGTGCGG
TCAGTGCCTAAAGTGGATTACTTGACCTTGAGACATGGATTTATCACGGCACATTTGGCTCCACAAAGTCAACAGAAGTT
CGATTTTCGTAAATATATAAAAAGATCGTTGGAAGAGGATTTCAAAGTTGTAGTAGGAATTAGTCCTCTATTCTGGTTCT
TCGCAGTCATGTTTCTCCTCTTCAACACTCATGGCTGGTACAGTTATTTATGGCTTCCATTTATCCCTTTGATTATAATC
TTATTAGTTGGAACCAAGCTTCAAGTGATCATAACGAAGATGGCTCTTAGTATTCAACAAAGAGGAGAGGTCCTGAAGGG
TATGCCATTGGTGCAACTTGGGGATCACCTCTTTTGGTTTAACCGACCTAGTCTTATTCTTCACCTCATCAATTTTGTTC
TCTTTCAGAATGCATTCCAGCTTGCGTTCTTTGCATGGACTTCGTTTGAATTTGGGATTAAATCCTGCTACCATTCGCAT
ACAGAGGATGTGGTGATTAGAATCTCAATGGGGGTCCTCATTCAAATTCTATGCAGCTACGTTACACTTCCTCTCTATGC
TCTTGTCACACAGATGGGTTCAAGCATGAAGGCATCTATATTCAACGAAAGGATAGCCATGGCCCTACGAAATTGGCACC
ATAGAGCAAAGAAACACAAAAAACAGAACCGAAATGGTTCATTATCAATGACCCCAATATCAAGTAGGGCTACAACCCCA
ACTCATTCTATGTCCCCTACCCATCTTTTAAGCCACTATCGTTCCCAAATAGATAGCTCTTATTATGCCTCTCCAAGAAG
ATCCATTGAAATAGAAGATTCTCCTTCTACTTCCTACTCTTACCACCACAAGAATAAAAAGGCTAAGGTTGATTATTTTT
TTGATCCAACTCCTTCTACTACTACTACTACTTCAACTAATCACCATGAAATTGAAATGGACCAACATATTAATGAAGTA
AACCCCACTACCACACAACATGAGATTGTTATTGCACATCATAGCAAGGAGTTTTCATTCGATAAAGGAACTACACAATC
AAGTGCACAAAATACTAGCAAACTAGGTAGATGA

>LaMLO5 AOCW01127929
ATGGCTGAGGAACTGAACCAATCTCTGGAATATACACCAACATGGATTGTTGCTGGTGTTTGCTCCATCATTGTTTTCAT
CTCTCTAATTGTTGAGCGTTGCCTTCACTGGCTTGGAAAGTACTTAAAGCGTGAAAAGCAGACTGCATTATATGAGGCCT
TAATAAAATTGCAAGAAGAATTGATGCTTTTAGGGTTCATTTCCCTTCTATTAACTGTGACTCAAAATATAATAAGTGAT
ATATGCATCCCACCTAAGCTTGCAACCCAAATGCTTCCATGCAAGAGGCCTCATGAGTCTTCAGAAGAATCAACACATAG
ATTGATCTACTATGATACTATAATCAACAGAAGAAGGCTTTTTTCTACAGAAACTGGTTTTGAGCATTGTAAGGAAAAGG
GAAAGGTTCCGCTATTATCGCAAGAAGCATTACATCATCTTCACATTTTCATCTTTGTATTGGCCATTGTACATGCAATA

TTCTGTGTCACCACAATGCTTCTTGGAGATCATCTATATATAAGAAATGGAATTGACTTGTCTCTTAAATTTCCAATTTT
CTCGGTTTTTTCGTATCCAGATGATTACCATCATCATGAGTTCTTCAAAAAACATGCTGATGGATATTGGAGAAGAGCAG
CTGTTGTAGGCTGGCTGATATCATTCTTCAAGCAGTTTTATGGTTCTGTTACTGAATCCGACTACACTGCACTGCGTCAA
GGATTCATCAAGGAACATTGTTCACACAAACCTGATTTTAATTTTCACAAGTACATAATGCGGACGCTAGAAGTTGATTT
CAAAAAAATTGTAGGCATAAGGTGGCACCTGTGGCTCTTTGTTGTACTGTTTTTGCTGCTAAATCTTCATGGTTGGCACA
CTTATTTCTGGCTATCTTTCTTACCATTGATAATTCTACTGCTGGTGGGGGCAAAGTTAGAGCACATAATAGTTCGTTTA
GCGCAGGAATCGGTAGAAAAATTGGCAGAGGGAAATTTTGTTACACAAGCTGTGGGAAACTTGGGGAGAAGAAAAGAACA
AGATGCAAAACCTGTGAAACCATCAGATGGATACTTCTGGTTTAACCGCCCTGCCCTTGTCCTTGACTTACTTCATTTCA
CTTTGTTTCAGAACTCTTTTGAGATTGCATTTTTCTTTTGGATTTGGTGCACATATGGATTTGATTCATGCATCATGGAG
AAAATAGGCTACATCATCCCAAGGATTATAATGGGTGTAATTGTTCAAGTGCTTTGCAGTTACAGTACCTTGCCTTTGTA
CACTCTTGTGACTCAGATGGGTAGCAGATTCAAGAAGGGTATGTTTGATGAGGTGGTTGAGGCAGGTATTTTGAGTTGGG
CTGGAAAATCTATAAGTGGAGCTGATGATGAGTCCTCTGAACCTGATAGAAGGAGGACCATGTCTCACAAAATGATCAAA
GAATCATTCCAACCTTCTCAAATTAGTGGACAAGCAATCATCATGGTGGAAGATGAAACAACATCTACAATTGAGCTTCC
TACATTGTCAAAGTTCCATTGA

>LaMLO6 AOCW01133085
GGTGGTGGTGGTGGTGGCGGCGAAGGTGGTCACGGTCACACTGATAACAGAACTCTAGAGGAGACACCTACGTGGTCTGT
CGCCATGGTTTGTGCAGTTATCGTTATCATTTCTATATCGTTGGAGAAATTTATACATAAATTCGCAAAGGTGTTTGAAG
CAAAAAAGAAGCTTGCTTTGCTGGAAGCTCTTGAAAAGATTAAAGCAGAGCTTATGGTTTTGGGATTTATTTCATTGCTC
TTAACATTTGGCCAATCCTACATTTCTAAAGTGTGTGTTCCTCAGTATTATGCAAATCAAATGTTACCATGTAGTCGGAA
GAAGTCACATGGGCCTCCAGAGGAAGGGCACGATACGGGTGGTGGTGGTGGTGAGAATGAAGCCGCGGAACATCGTCGCA
GGCTTTTGTCATATGAACGTAGGTTTCTAGCTGGTGGTGGTGGATTTGTCGAGTGTAAATTGGGATCTGAGCCTCTTATA
TCCGTGGGAGGGTTGCATCAGTTACACATTTTCATATTCTTCTTGGCTGTCTTCCATGTGATATACAGTGCCATAACAAT
GACTCTTGGAAGAGCTAAGATTCGTGCATGGAAGCAATGGGAACAGAGCCATCTCGTTGATGAAGACGCCTTGAATGATC
CCAGAAGATTCAGGCTTACTCATGAAACATCATTTGTGAGGGATCACAGCAGTTTCTGGACCAAAACACCAGTTTCCTTC
TATTTTGTATGTTTCTTTCGACAATTTATCAGATCTGTTCGTAGGGCAGACTACTTGACCATGCGACATGGATTTGTGTC
TGTCCATCTAGCACCTGGAAGTAAGTTTGATTTTCAAAAGTACATCAAAAGGTCATTAGAAGATGACTTTAAGGTAGTTG
TGGGAATCAGTCCAATACTTTGGGCTTCAGTGGTGTTATTCTTGCTTGTAAATGTGAATGAATGGCACGCATCATTTGGG
TTGTCTTTTCTTCCACTAGCCGTGGTTCTGTCTGTTGGAACAAAGCTTCAAGCGATTATAACTCGAATGGCACTTGACAT
AAAAGAAAGGCATGCTGTAGTGCAGGGTATCCCTCTTGTGCAAGTCTCTGACAAGTACTTTTGGTTTCAATGGCCTCAGT
TAGTTCTTTATCTCATTCATTATGTCCTCTTTCAGAATGCATTTGAGCTGACATTTTTCTGCTGGACGTGGTATGAGTTT
GGCTTGAATTCTTGCTTTTACGAGAATAAAAAATTGATGTATTTCAGAGTTGCTGTTGGGGTCGGAGCACAAATCGTGTG
TAGCTATGTCACACTTCCACTCTATGCGCTGGTTACTCAGATGGGATCAACAATGAAGAAGTCAATATTTGATGATCAAA
CATCTAGAGCACTGAAAAAGTGGCATAAGAATGCTCTGAAGAAGGCAAATTCAAAGGGTCGTGGTACAGAAATTAGAACA
TTGGGTGGGAGCCCAAGACATTCACCACGTGCAAGTGAAAATGCTTATTCTAACATTGCAGTAACAGAAACACAGATGAC
TGAACAAACCCACCAAGGATTATAA

>LaMLO7 AOCW01138453
ATGGGTGGAGGTGGAGATGCAGGTGCAGGTTCTCATGGTCATGGTGATAACAGAACTCTAGAGCAAACTCCTACATGGTC
TGTTGCTGCTGTTTGTGCAATTATTGTTATCATTTCTATATCATTGGAGAAACTTATACATAAATTCGCATCGATGTTTG
AACAAAAAAAGAAGCTTGCTTTGTTAGAAGCTCTTGAAAAGATTAAAGCTGAGCTTATGGTTTTGGGATTTATTTCATTG
CTCTTAACATTTAGCCAATCCTACATTTCTAAAGTGTGTGTTCCTCATCATTATGCAAATCAAATGTTGCCATGTTCTCG
TCCGGAAGAGTCGTATGGAGCAGCACATGGACCTCCTAAAGAGGAGGAGGGTGGCGAGGATGAAGGCGAACATCGTCGAA
GACTTTTATCGTATGAACGTAGATTTCTTGCTGGTGGTGGTGGAATGGTCAATTGTAAACCGGGATATGAGCCTCTTATA
TCTGTGAATGGGTTGCATCAGTTACACATTTTCATATTCTTCTTGGCTGTCTTTCATGTGATATATAGTGCCATAACGAT
GACGCTCGGAAGAGCGAAGATTCGTGCATGGAAGGAATGGGAACAAGATCATCTCATTGAAGAAGACGCCTTGAATGATC
CTAGAAGATTCAGGCTTACTCATGAGATTTCATTTGTGAGGGATCACAACAGTTTCTGGACCAAAACACCAATTTCCTTC
TATTTTGTATGTTTCTTTCGACAATTTATCAGATCCGTTCGTAGGGCAGACTACTTGACCATGCGACATGGATTTGTGTC
TGTCCATTTAGCACCTGGAAGTAAGTTTGATTTTCAAAAGTACATCAAAAGGTCATTAGAAGACGACTTTAAGGTAGTCG
TCGGAATCAGTCCACTACTTTGGGCATCAGTGGTGTTATTCTTACTTGTGAATGTGCATGAATGGCACGCTGCATTCGTG
TTGTCTTTTCTTCCATTATCGGTGGTTCTGGCTGTTGGAACAAAACTTCAAGCAATTATAACTCGAATGGCACTTGACAT
AAAAGAAAGGCATGCTGTAGTACAGGGTATCCCTCTTGTGCAAGTTTCGGACAAGTACTTCTGGTTTCAATGGCCTCAGT
TAGTTCTTTATCTCATTCATTTTGTCCTCTTTCAGAACGCATTTGAGCTGACATTTTTCTGCTGGACATGGTATGAGTTT
GGCTTGGGTTCTTGCTTTTACGAGGATAAATTTTTATGTTCTTCAGAGTTGCTATTGGGATCGGAACACAGATCGTGTGC
AGCTATGTCACACTTCCACTCTACGCGCTTGTTACTCAGATGGGTTCAACAATGAAGAAGTCAATATTTGATGACCAAAC
ATCGAAGGCGCTGAAAATGTGGCATAAGAATGCTCTGAAGAAGGCAAATTCGAAGGGGCGTGGTACAGAAGTTCGAACAT
TAGGTGGGAGTCCAGGACATTCACCACCGCATTCACCACACGCAAATGGAAATGCTGGCACTAACATAGTAGTAGCAGCA
GAAACACAAATGACTCAACAGAACGCTGCCGCTATAGCCACCAACGCCGATCATAATCAAAACCAGCAGTATGAAAACAT
TGACCTTCTAAGTGGGCCTTGA

>LaMLO8 AOCW0114634
ATGTCTAAGTCAAAGTTGAAAAAACTGTTATTTTATGTTCTGTTTTCAATGCTGGTCTGGTTTGGAAGTTATGGTATGGC
GGCAAGTGAAAGTAGTGGTAATTCGAGAGAGCTTGATCAGACACCAACTTGGGCTGTTGCTGCTGTGTGCACTGTTTTTA
TCTTGGTTTCCATTACTTTGGAGAAGAGTCTTCATAAACTTGGAACGTGGCTAGGACATAGGCAAAAGATGGCTCTGTTC

GAAGCACTAGAAAAGGTTAAGGCTGAGCTTATGATTTTAGGTTTCATTTCACTGCTTTTGACTTTCGGGCAGAGTTACAT
AGTCAAAATATGCATTAGTGCAAGTGTTGCGGATAAAATGCTGCCATGTCCATATAGAGAAAAAGGCACCAACAATGCAT
CAAGTAGTGGAGGGGAACACCGTAGGAAACTTTTTTCTTATGAACGTAGATATTTAGCTGCTGATGCTACCTCGTACAAA
TGCAGCGACGAGGGACATGTGCCACTTCTATCTGTGAATGGATTGCACCAGTTACACATCCTCATATTCTTCTTAGCAAT
CATTCATGTGCTTTACAGTGCTGTCACAATGCTTCTTGGGAGGCTAAAGATACGTGGCTGGAAGGCATGGGAGGAGGAGA
CTTCAACTCATGGGTATGAGTTATCCATTGATGCTTCAAGATTCAGGCTTACTCATGAAACATCATTTGTGAGAGCCCAT
GCAAGTTTTTGGACGAAGATTTCAATCTTATTCTATATTGGCTGCTTCTTTAGGCAGTTCTATAGTTCTGTGGGCAAGGT
TGATTTCCAAACTTTGCGCAATGGGTTTATCGCTGTCCACTTAGCCCCTGGAAGTAAATTTAACTTCCGAAAGTACATCA
AAAGATCCTTGGAAGATGACTTCAAGGTAGTTGTCGGAGTCAGTCCTGTACTCTGGGCATCAGTTGTAGTTTTCCTGCTC
CTAAATGTTAATGGATGGCAAACTCGAATTTGGGCAGCCTTAATTCCTGTTGTTATAATTTTGGCTGTTGGAACAGAACT
TCAAGTCATATTGACAAAGATGGCTCTTGAAATCACAGAGAGACATGCAGTTGTTCAAGGGATTCCTCTTGTTCAAGCCT
CGGACAGATACTTTTGGTTTGGTCAGCCTCAATTAGTTCTCCATCTTATCCATTTTGCTTTGTTCCAGATTGCATTCCAA
ATAACATATATCTTGTGGACATGGTACTCATTTGGGCTAACAAACTGTTTCCGTATTGACTACAAGCTTGCAGTTGTTGA
ATTAGCTTTAGGGGTCATTATGCTATGCCTCTGCAGCTACAGTACCCTTCCATTATATGCTCTTGTTACTCAGATGGGTT
CAAGTTTGAAAAAATCAATATTTGATGAACATACAGCCAAAGCATTAAGGAAATGGCACATGACAGTAAAAAAGAAGCAG
GGAGTAGTAAAGCTAGGAAAATCCACCATGCGAGCCATGGACATGGATGGAAGCACCACCATTGGTTCAACAAAACTGCA
TTCTTCTCGACCCACACTTCACCGTTTAAAAACTACTGGCCATTCAACTCACACATCAATATATGAGGATGAAGATGAAT
ATCAATCTGATATTCAATTGTCTCCAACATCATCGTCAACGACCTTGGTTGTAAATGTGGATAATGGTCATCAGCAACAA
GTAGAAGAAAATACTGAAGAAACCAACATTCAAGGTGACTTCTCATTTGTCAAGCCTAGCCCTATAGAAAAGAACCATGA
CGTAGAATTATTATAA

>LaMLO9 AOCW01154130
ATGTCTGTCTCAAAGTACAAAAAGCTATGTTTTTGTGTGGTTTCTTGGTTCTGTTATGAAGTTGGTTTGGTTATGGGTTC
TTCAAATGAGAAGGGTAGTGGAAGTTCTAGAGAACTTGATCATACACCAACATGGGCTGTTGCATTTGTTTGCACTGTTT
TCATCTTAATATCCATAACTATGGAGAAGAGTCTTCACAAAGTTGGAATGTGGCTCCAACAAAGGCACAAGAAGTCTCTC
CTTGAAGCTCTAGAAAAAGTCAAAGCTGAGCTGATGATTCTTGGTTTCATTTCACTGCTTTTGACATTCGGGCAGAGATA
CATTGTTAGAATATGTATTCCGGCCAAAGTTGCTAACAAACTGTTGCCATGTCCACTCAAAGACAAACATCGCACGAAAC
TAGTCTCTTATGAACACAGATATTTATCAGAAGATACTACCTCTCTCCGATGCAAGGGGAGATACGTGCCTCTTCTATCT
GTCACTGGATTGCACCAGTTACACCTTCTTCTATTCTTCTTAGCAATCCTTCATGTTCTTTACAGTGCTGTGACAATGCT
TCTCGGGAGGTTAAAGATACGTGGATGGAAGGCATGGGAGGAGGAGATTGCATCTGATAGGTTTGAGTTTGACAATGATC
GCTCAAGGTTCCAGTTTACTCATGATACGCCATTTGTGAGAGCTCATGCCACTTTTTGGACACGATATTCAGTCTTCTTT
TATATCGGCTGCTTCTTTAGACAGTTCTATAGGTCTGTGCGTAAGGCCGATTACGTGACTTTGCGCAATGGATTTATCAC
TGTCCACCTTGCTCCTGGCAGTAAATTTAACTTCCAAAAGTATATCAAAAGATCCTTGGAGGATGACTTCAAGCTAGTTG
TCGGAATCAGTCCTGTCCTCTGGGCATCGTTTGTTGTTTTCCTGCTCCTAAATTTTAATGGATTGCAAGCTATGTATTGG
GCATCCTTAATTCCTCTTATTATAATTTTGGTAGTCGGAACAAAACTTCAAGCCACATTGGCAAATATGGCTATTGAAAT
CACAGATAGACATGCAGTTGTTCAAGGGATGCCTCTTGTTCAAGGCTCGGATAAATATTTTTGGTTTGGTCGGCCGCAAT
TAATTCTTCATCTTATCCACTTTGCCTTGTTCCAGAACGCGATCCAAATAACGCATATCTTGTGGATACGGTATTCTTTT
GGGAAGGAAACCTGTGTCCATGCAGGCGACATACTTGCAATAGTGAAAGTAGCTTTAGGGGTTGTGGTCCTATGCCTTTG
CAGCTATATCACTCTTCCATTGTATGCTCTAGTAACTCAGATGGGATCAACGATGAAAAAGTCGATATTCGATGAACAAA
CAAAGAAAGCCTTAAAGAAATGGCTCATGGATGCGAAAAAGAATAAAGGATCGAAAGCTGGAAAATCCACTGTGAGAACT
GCAGATGGAAGCACCAATGGTTCTACAATACATTCATCTGGACCTACACTACACCGATTCAAAACTACGGGTCACTCAAC
TCGAACTCGAACTATATCACCCGATTATAACCAAGATGATCACCATTCTGATATCGAAATGTCTCCGATGTTACCAACAT
CAAGTTTAATTGTTAGAGTGGATCATGGTGACCAAGAAGCAAAAGAAAATGAACACCAAGTGTAG

>LaMLO10 AOCW01039454_AOCW01039455
ATGTCTAACTCAAACTTCAAAAAAATGTTTTTATTTTTTCTGTTTTCAATGCCAATCTGGTTTGGAAATTCTGGTATGGC
TGCAAGTGAAGGTAGCAATGATTCAAGGGATCTTGATCAGACACCAACTTGGGCTGTTGCAGCTGTGTCCACAGTTTTTA
TATTGATATCCATTACCTTGGAGAAGAGTCTTCACAAAATTGGAACATGGCTAGGACATAAGCAAAAAATGGCTCTGCTC
GAAGCACTAGAGAAGGTCAAAGCTGAGCTAATGATTTTAGGTTTCATTTCATTGCTTTTAACTTTCGGGCAAAGTTACAT
TGTCAGAATATGTATTCATGCAAATGTTGCGGCCAAAATGTTGCCATGTCCATATAGAGATCAAAACACCGACAATGAAT
TTGGTAATGAAGAGGAACATCGTAGGAAAGTTCTGTCTTACCAACGTAGATATTTAGCTGCCGACACTACCTCCTACAAA
TGCACCAACGAGGGACATGTTCCACTTCTATCTGTGAATGGATTGCACCAATTACACATCCTCATATTTTTCTTAGCAAT
CATTCATGTGCTTTACAGTGCTGTCACAATGCTTTTGGGGAGACTAAAGATACGTGGATGGTTTCATCAATGTTACTTTG
AAATAGTGCTATATGAGACATTGAGAGATACTTCAAGATTTAGGCTTACTCATGAAACATCATGTGGGAAAGCCCATGCC
AGTTGTTGGACAAGGAGTTCAATGTTCTTCTATATTGGCTGCTTCTTTAGGCAGTTCTATAGGTCTGTGGGTAAGGTTGA
TTTCCAAACTTTGCGCAACGGGTTCATCGCTGTCCACTTAGCTCCTGGAAGTAAATTTAACTTCCAAAAGTACATCAAAA
GATCATTGGAAGATGACTTCAAGGTAGTTGTGGGAGTCAGTCCTGTCCATTGGGCATCAGTTGTAGTTTTCCTGCTCCTA
AATGTTAACGGATGGCAAACTCGGCTTTGGGCAACCTTAATTCCTGTTGCTATAATTTTGGCTGTTGGAACAAAACTTCA
AGCCATATTGACAAAGATGGCTCTTGAAATCACGGAGAGACACGCGGTTGTTCAAGGGATACCTCTTGTTCAAGCCTCAG
ACAGATACTTTTGGTTTGGTCAGCCTCAATTAGTTCTTCACTTTATCCATTTTGCTCTGTTCCAGATTGCTTTCCAAATA
ACATATATCTTGTGGACATGGTATTCTTTTGGGCTTAGAAACTGTTTTCGTGTAGACTACAAGCTTGCAGTTCTTGAAGT
AGCTTTAGGGATCATTATGGTATGCCTCTGCAGCTACATTACCCTTCCATTATATGCTCTTGTAACACAGATGGGTTCAA
GTTTGAAAAAATCAATATTTGATGAACAAACATCGAAGGCATTAAAGAAATGGCACATGACAGTAAAAAAGAAGCATGGA
AAAGTGAAGCTAGGAAAATCCACCGTGCGAAGCATGGACATGGATGGAAGCACCATTGGTTCAACAACATTGCACTCTTC

TGGACCCACACTTCACCGTTTCAAAACTATTGGCCACTCAACTCGCACATCAACATATGAGGATGAGGATGAGGATGAGT
ATCAGTCTGATATTGAATTTTCTCCCATATCAACATCAATGAACTTGATAGTAAAAGTAGATCATGGTCATGAGCAACAA
GGAGAAGAAAATAGGGAAGAAGCAAACATAGAAGGCGAGAAAAGAACCATGACATAG

>LaMLO11 AOCW01135152
ATGTCTATGTCTATGTACAAAAAGGTTTCATTTTGTGTTGTTTTTTGGTTATGTTATGGAATATTAGGAATGGGTTCTTC
AAGTGAGAGTAGTAACAGTTCAAGAGAACTTGATCAGACTCCAACTTGGGCTGTGGCTTGTGTTTGCACTGTTTTCATCT
TGATTTCCATAACTATGGAGAAGAGTCTTCACAAAGTTGGATTGTGGCTCCAACAAAGGCAGAAGAAGGCACTGCTTGAA
GCTCTTGAAAAAGTGAAAGCTGAGCTGATGATTCTAGGCTTCATTTCACTGCTTCTGACTTTCGGACAAAGCTACATTGT
TAGAATATGCATTGGGGCCAACATTGCAGAGAAACTGTTACCTTGTCCATATAATTCGGAAGGTATCGGAAATGAATCAA
GTAGCAAAGATGAACATCGTAGGAAACTCCTCTCTTATGAACACAGATATTTATCAGAAGATACTACCTCTTACCATTGC
AAGGAGGGACATGTGCCACTTCTATCTGCCAATGGATTGCACCAGTTACACATTCTCATATTCTTCTTAGCAATTTTTCA
TGTTCTTTACAGTGCTGTCACAATGCTTCTTGGGAGATTAAAGATACGTGGATGGAAAGCATGGGAGGAGGAGACTTCAT
CTCATGGGTTTGAGTTCGCCACCGACCCTTCAAGATTCCGGCTTACTCATGAAACATCATTTGTGAGAGCTCATGCCACT
TTTTGGACAAGATATTCAATCTTCTTCTATGTTGGCTGCTTCTTTAGACAGTTCTATAGGTCTGTGCGTAAGGCTGACTA
CCAGACTTTGCGCAATGGATTTATCGCTGTCCACCTGGCTCCGGGAAGTAAATTTAACTTCCAAAAGTATATCAAAAGAT
CCTTGGAGGATGACTTCAAGATAGTTGTAGGAGTCAGTCCTGTCCTCTGGGCATCGGTCGTTGTTTTCCTGCTCTTAAAC
GTTAACGGTTGGCATGCTATGATTTGGGCGTCCTTAATTCCTGTTGTTATAATTTTGGTTGTTGGAACAATACTTCAAGC
CACATTGGCAAATATGGCTATTGAAATCACAGAAAGGCATGCAGTTGTTCAAGGGATACCTCTTGTTCAAGGCTCCGATA
AATATTTTTGGTTCGGTCGTCCGCAGTTAGTTCTTCATCTTATTCACTTTGCCTTGTTCCAGAATGCATTCCAAATAACA
TATTTCTTGTGGATATGGTATTCTTTTGGGCTGAAAAACTGTTTCCATGCAGATTATGAGCTTGCAATAATAAAAGTAGC
ATTAGGGTTCGTGGTCCTATGCCTTTGCAGCTACATCACTCTTCCATTGTATGCTCTAGTAACTCAGATGGGATCAAGAA
TGAAAAAATCGATATTCGATGAACAAACAGCCAAAGCGATAAAGAAATGGCACATGGCTGCGAAAATGAAGCACGGACTA
AAGCTAGGAAAGTCCAGCGTGCGAACCGCAGATGGAAGCACGACGGTTGGTTCTACAATACACTCGTCTGGACCCACACT
ACACCGTTACAAAACTACTGGTCACTCAACTCGCACCTTGTCAAGCTATCACGATCAAGATGATTACCATTCCGATATCG
AAATGTCTCCCGTCTCACGAACATCAAGGTTATATGTGAGAATGGATGATGGTGAGCAAGAAGCCGAAGAAAATGAACAC
CTCCATGTAGGCGAAACCAACCATGGAGATGATGGCTTGACAAATGTCAAGCCTGCTCCCTTATGA

>LaMLO12 AOCW01152069
ATGGGAGATGTGCATGAGGCAGGTAGGTCTCTAGCTGAGACACCTACATATTCAGTTGCTTCAGTTATTACTGTCATGGT
CTTCCTCTCCTTCTTAGTTGAACGATCTATCTACCGCTTTGGAAAGTGGTTGAAGAAGACAAGAAGGAAGGCACTTTTTG
CTTCACTTGCCAAAATTAAGGAAGAGCTCATGCTGCTTGGCCTTATCTCCTTGTTCTTGGCACAAAGTGCAAGGTGGATT
TCTGAAATTTGTGTAGATTCCTCACTTTTCACTAGTCAATTTTATACCTGCTCTCAACAAGAAATAAGTGCCACCAATCA
CAAAATCATGCTTCAAACTTCTCCCCCTTTCCCTCCAAGTGAATCAACTACTGTTGCAAAGGAAGTAAATAATGTTGCAT
TTCATCAATGTAGAGAGGGGCATGAACCATTTGTTTCATATGAGGGTCTTGAGCAGCTGCATCGGTTCTTATTTGTTCTT
GGCATCACTCATGTTCTCTACAGTTGTCTCGCAGTTGGCTTAGCAATGAGCAAGATCTATAGTTGGCGCAAATGGGAAAA
CCAAGCTACCATGGCAGCCGATGGAAATCTGCAAGCAAAGAAAATCAAAGTGATGAGGCGGCAGATAACCTTTATTTCTC
ATCAAACTTCTCATCCTTGGAGCAGAAGCAGGAATCCCTTACTAATCTGGATGCTTTGTTTTCTACGTCAGTTTAGGAGT
TCTATACAAAAATCCGATTACCTCGCACTCCGTCTTGGTTTCATCACTGAACACAAATTGCCCCTGTCATATAATTTTCA
TAATTATATGGTTCGTAGCATGGAAGACGAATTTCAAGCCATTCTAGGAATAAGCTGGCCGCTTTGGATTTATGCTATAG
TCTGCATCTTTGTGAATATCCATGGATTGAACATATACTTCTGGCTCTCATTTATCCCTGCACTGGTTGTCATGTTAATA
GGGACAAAACTTCAACATGTTGTATCAACTTTAGCAATTGAAATATTGGAGCAAACAGGTCCATATGCTAGGACACAAGT
AAAGCCACGTGACGATCTGTTTTGGTTCAAAAAACCAGATATACTGTCATGGCTCATACAATTTGTGATGTTTCAGAATG
CCTTTGAAATGGCAAGTTTTCTTTGGACATTGTGGGGTTTCAAAGAACAATCATGCTTCATGAGAAATCATTACATGATC
ATCATTCGGTTGACTTCTGGTGTTCTTGTTCAGTTCTGGTGTAGCTACATGACAGTGCCCCTCAATGTAATTGTTTCTCA
GATGGGATCCCGTTGTAAGAAGGCGTTGGTGGCGGAGAGTGTTAGAGAGTCATTGCATAGTTGGTGCAAGAGAGTGAAAC
AGAAGTCCAAGCATGATTCTTTGCATACAGCTAGATCAGTGTGTTCTATTGAATCAACAATTGATGAGATAACAGTAGTA
TCTGGTACTTTAACTAGAAGCTCTTCCTTAGAGTATATGAATCAGTTAACAGTAACTTCAGTAGACCAGCTCAACTTTCA
CACTTGTGATAACAACAATCAAGATTCAATCCATGTTGCCAATGGAGAGGAACAAAATGTTCAGACACTCCTTCATTTAT
TTCAGAAGACATGA

>LaMLO13 AOCW01152789
ATGGGTGGTGGTGGTGGTGAAGATACGACTTTGGAGTTCACTCCGACGTGGGTAGTGGCTGGCATATGCACAGTGATCGT
GGCCATTTCCCTAGCAGCGGAGCGTGTCCTTCATTATTGCGGTTTGTTCCTAAAAAGGAAGAATCAGAAGCCTCTTTATG
AAGCTCTCCAGAAGATCAAAGAAGAACTCATGCTGCTGGGTTTTATATCCCTACTATTGACGGTCACACAAAACAGCATC
ACCAAAATCTGCGTTCCATCTGCTTTCACTCATCATATGCTCCCTTGCACTTTCCAATCTCATTCACAAACCCATTCTCA
TTCTCAAACCATCTTCTCTTTCCCTCCATTTTCTAGGCGCCTTCTTTCTCATGCCGATGCCAATTTCTGCTCTCACAAGG
ACAAGGTACCTTTGCTATCTCTGGAAGCAATACATCACCTTCACATCTTTATCTTTGTCCTAGCTATTGTCCATGTCACC
TTCTCTGTTCTCACCGTCCTTTTTGGAGGAACAAAAATACGTCAGTGGAAGCACTGGGAAGATTCAATAGCACAACAGAA
CTACGAAACAAATCGAGTTCTGAAGCCAAAGGTGACAAATGTTCAACAACATGATTTTATCAAGGGTCGTTTTTCTGGTT
TCGGCAAAGATTCTGCTTTAATGGGTTGGTTGCTATCCTTTTTCAAGCAATTTTATGGATCTGTGACAAAATCAGATTAT
GTGACATTACGACTTGGCTTCATTATGACCCATTGCAGGGGAAATCCTAAGTTTAATTTTCACAAGTACATGATTCGTGC
CCTCGAAGATGATTTCAGACAAGTTGTTGGGATAAGTTGGTATCTTTGGGTCTTTGTGGTCATCTTCTTGTTGCTTAATA
TCAACGGTTGGCACACATATTTCTGGATTGCTTTCATTCCTTTCTTTCTGTTACTTGCTGTTGGCACTAAGCTGGAGCAT

GTAATAACCCAATTGGCTCATGAAGTAGCTGAAAAACATGCAGCCATAGAAGGTGAATTAGTTGTTCGACCATCAAATGA
CCACTTTTGGTTTCAGCGGCCCCAGATTGTCCTCTTCTTGATTCACTTCATACTTTTCCAAAATGCTTTTGAGATAGCAT
TTTTCTTCTGGATATGGGTTACTTACGGATTTGACTCCTGTATAATGGGTCAAGTCCGTTACATTATTCCGAGGCTCATT
ATTGGGCTATTTATTCAGGTACTATGTAGCTACAGCACCCTGCCACTCTATGCGATTGTTACACAGATGGGAACTAACTA
TAAGAAGGCGATATTTGAGGAGCATGTGCAAGTGGGGCTTATTGGTTGGGCACAAAAGGCAAAGATGAAGAAAGAACAGA
AAGCTGATAGCCAACATGAGTCTGGGCAAGGAAGTTCTCATGTAGGAATTCAGCTTGGTCAATTATTCCCCAAAAGGGCA
TCTGCACCACAAGACAATACTTTTATCCCACCCAAACCTGATGGATCCACCTGA

>LaMLO14 AOCW01138218
ATGAGTGGTGGGGGTGTGGAAGAAGGAAGTAGTTTGCAGTTTACACCAACATGGGTTGTTGCTTTAGTTTGTAGTGTAAT
TGTTGCTGTTTCACTTGCTGTAGAACGTTTACTTCATTATGGTGGAATGTTTCTTAAGAAGAAGAATCAGAAGCCACTTT
ATGAAGCTCTTCAGAAAATCAAAGAAGAGCTGATGCTGTTGGGTTTCATATCACTGCTTATGACTGTATCACAAAATTCA
ATATCCAAAATCTGTGTTTCAAAGAGTTGGACTGAGCATATGCTTCCATGTAGCCGTGAGGATTTAGAAAAAGAAGGATC
AAAAACACCAATTTCACATTTTCAGACATTTTTCTCATTCTCTGGCAATGCTAGGCGCTTACTTTCTGAGGGCCAAGCAG
CACCTGAGGTCAAATCTGGATATTGCTCAAGTAAGAACCAAGTACCTCTATTATCCATTGAAGCACTGCATCAACTGCAT
ATCTTTATATTTGTCCTAGCTATAGTTCATGTAACCTTCTGTGTTCTAACCATTGTTTTTGGAGGATTAAAAATACATGA
GTGGAAGAATTGGGAAGATTCCATAGCGAAAGAAAACTACGAAACACAGCGAGTTATGAATCCTAAGGTGACTCATGTTC
AGCAGCATGCTTTTATCAAGGATCATTTTACGGGTTTCGGCAAAGATTCGAGTCTTCTGGGTTGGGTGAAAGCTTTTTTC
AAGCAATTTTATGGGTCAGTGACAAAATTAGATTATGTGACATTAAGGCTTGGTTTTATTATGACCCATTGCAGAGGAAA
TCAAAAGTTTAATTTTCACAAGTACATGGTTCGTGCCCTTGAAGATGATTTCAAGATAGTTGTTGGTATAAGTTGGTATC
TTTGGATCTTTGTGGTCCTCTTCTTGTTGCTTAATATCAGTGATTGGCATACATATTTCTGGATTTCATTCGTTCCATTC
ATTCTTCTGCTATCAGTGGGCACAAAGTTGGAGCATATAATAACCCAATTAGCACATGATGTAGCTGAAAAGCATGCAGC
CATAGAAGGTGAACTTGTTGTACAACCATCAGATGATCACTTCTGGTTCCATCGTCCCTACATTGTTCTCTTCTTGATTC
ACTTTATCCTTTTCCAAAATGCTTTTGAGATTGCATTCTTTTTCTGGATATGGGTTACATATGGCTTTGACTCTTGTATA
ATGGGACAAGTCAGTTACATTTTTCCTAGACTCATTATTGGGGTATTTATTCAAGTACTATGTAGTTACAGCACCCTGCC
ACTGTATGCAATTGTTACACAGATGGGGACTCACTTTAAAAAGGGAATATTTGATGAACAAGTGCAAGCACGCCTTGTTG
GTTGGGCACAAAATGCAAAGAAGAAAGGAGTAAAAGGTGATAGCCAACATGGTGAAGGAAGTTCTCATAGCAATGGTGCT
GTTGGAGTTCAACTAGGGTCAATTTTCAAGAAGCAATCTGCACCAGAAGACAATGCAATTGTCCCAACAAATGAAGAGTC
CAAGTTAATATAG

>LaMLO15 AOCW01116641
ATGGCAGAAAATGTAGAAGAAACACGGTCGCTGGCCATGACTCCTACATGGTCTGTTGCCACTGTGTTGACTGTCTTTGT
TGCTGTCTCCTTGCTTGTGGAACGCTCCATTCACCGTTTAAGCAATTGGCTGCGTAAAACTAATCGAAATCCGTTGTTTG
CAGCTTTGGAGAAGATGAAAGAAGAGTTGATGTTGCTTGGATTTATATCACTGCTATTAACGGCTACCTCACGCATGATA
GCCAATATTTGCATTCCATCAAAGTACTACAATAGTGCTTTTGCTCCATGCACTAGATCTGAGATTAATGAAGAAATGGA
AGATAATGGCTCCGGAGAACGTAAATTGTTGATCTCTTCGGCCTACCCACACTTACTTAGGAGGATGTTGAATGGCATAA
ACAGAAGCTCCTGCAAAGAGGGTCATGAGCCGTTTCTTTCGTACGAAGGTCTAGAGCAGTTGCACCGATTCATTTTTGTC
ATGGCCGTAACACATATATCTTACAGTTGCCTGACAATGCTGTTAGCAATTGTGAAGATTCACAGTTGGAGAGTGTGGGA
GGATGAAGCTCACATGGATCGGCACAATTCGTTAAATATGACTATGCAAAGGCAATCAACATTTGTCAAGTTCCATACAT
CAAATCCGTTGGCCTCGAACAGTGTCCTCGTCTGGGTGACATGTTTTTTCCGACAATTTGGGCATTCTGTTGTTCGTGCT
GACTATCTTACGCTTCGCAAAGGCTTCATCACGAATCACAACCTCTCAAAGAAATATGATTTTCATAGCTACATGATTCG
ATCCATGGAAGAGGAATTCCAAAGGATAGTTGGTGTGAGTGGCCATCTCTGGGGATTTGTTGTGGCTTTCATGCTTTTCA
ACATCAAAGGATCTAATCTCTATTTCTGGATAGCTGTCATTCCCATATCGCTTGTACTTTTGGTGGGAACAAAGCTACAA
CATGTTATCGCAACCTTGGCTTTGGAGAATGCTGGAATCACTAGTTTCTTCTCAGAAGCAAAGTTGAAACCTCGTGACGA
GCTTTTCTGGTTTAAAAAGCCTGAACTATTGCTGTTCTTGATCCATTTCATTCTCTTCCAGAATGCATTTGAATTGGCTT
CATTCTTTTGGTTCTGGTGGCAGTTCGGATATAATTCCTGCTTTATTAGTAATCATCTTCTGGTGTATATAAGGCTAATC
TTGGGTTTTGCAGGACAATTTCTTTGCAGCTATAGCACCTTGCCACTTTATGCACTTGTTACACAGATGGGAACAAACTT
TAAGGCTGCAATAATTCCACAGAGGATAAGGGAGACAATTCATGGATGGGGTAAGGCAGCTAGGAAAAAGAGAAGACATG
GCATGTTCCCGGACGATTCCACGATTCATACAGACACAAGCACTGTATTGTCCATCGAAGAAGACGATGAATTTATCGAT
GATACTCCTACAACTGGCACTGGCACTGGCACTGGAGTAGAATTACAACCAATAACAACTATTACTTCTTCCCCTTTACC
CGCTGCTGCAAATGAAACTTCGAGTAGGGCCGCGACACCCCTGCTTCGACCCTCTGCCTCAATATCTTTATCATCTCCAT
CTAGCGCGAGAGCAGAAGCAGTTGCAAGATGCTTCTCTATGCCAAGTGGAAGACATTGA

>MtMLO1 Medtr6g033330
ATGGCTGAAGATAAAGTTTATGAAAGAACTTTGGAAGAAACACCAACTTGGGCTGTTGCAGTTGTGTGTTTTGTGTTGCT
TGCTATTTCAATCGTAATTGAACATATTATTCATGCTATTGGCAAGTGGTTCAAGAAGAAAAACAAAAATGCTCTTTATG
AAGCATTGGAAAAAGTCAAAGGAGAGCTTATGTTGATGGGATTTATATCCTTGCTCCTAACTGTGTTCCAAGATTATATT
TCCAAAATATGCATATCAGAAAAAGTTGGATCAACTTGGCATCCTTGTTCCACTCCAAAAACAAAAACTGCATCTAATGA
TGAAAATTCTGAGAGTGAAAACCATGATAGAAAACTCTTGGAATATTTTGATCCTAACCCTAGGCGAATTCTTGCTACAA
AAGGATATGATCAATGTGCTGATAAGGGTAAAGTTGCTTTGGTTTCTGCATATGGAATTCATGAACTCCATATATTCATT
TTTGTGCTGGCAATATTTCATATTCTACAATGTATAATAACACTAGCTTTGGGAAGATTCAAGATGAGGAGGTGGAAGAA
ATGGGAGGATGAAACAAGAACCGTTGAATATCAATTCTATAATGATCCTGAGAGGTTTAGGTTTGCAAGGGACACAACAT
TTGGAAGAAGACACTTGAGCATGTGGACCAAGTCACCCATTTCATTATGGATTGTTTGCTTCTTCAGACAATTCTTTGGA
TCTATTAGTAGAGTTGATTATTTGGCACTAAGGCATGGATTTATCATGGCACATCTTGCCCCAGGAAATGACGCAGAATT

TGATTTCCAAAAGTATATTAGTAGATCACTTGAAAAGGATTTTAAAGTTGTAGTGGGAATAAGCCCAACTATCTGGTTCT
TTGCAGTGCTATTCCTTCTTACAAATACTCATGGGTGGTATTCTTCTTATTGGCTTCCATTTCTCCCACTAATTATAATA
TTATTAGTAGGAGCTAAGCTACAAATGATCATAACAAAGATGGGACTAAGGATTCAAGATAGAGGAGAAGTAATCAAGGG
TGCACCTGTGGTTGAGCCAGGAGATCACCTTTTTTGGTTCAATAGTCCCAACCTTCTTCTCTTTATAATTCATCTTGTTC
TCTTTCAGAATGCCTTTCAACTTGCATTTTTTTCTTGGAGTACATATGAATTCTCTATAAATTCTTGCTTCCACAGAACA
ACTGCAGATAATGTCATTAGAGTCTCAGTAGGGATTTTAATACAATTTCTATGTAGCTATGTCACTTTGCCTCTTTATGC
TCTAGTCACACAGATGGGTTCAACCATGAAACCAACCATTTTTAATGAAAGATTGGCAACAGCACTCAAGAAATGGCACC
ACACAGCCAAAAAGCAAGTCAAACACAACAAACATTCGAACAACACAACACCGTACTCAAGCAGACAATCAACCCCAACA
CATGGCATGTCACCTGTTCATCTTCTACATAGACAAACTTTTGGCAACAGTGACAGTTTACAAACTTCTCCAAGGACTTC
TAACTATGAAAATGAGCAATGGGATGTTGAAGGAGGAGGATCAACTTCACCAAGAAACAACCAAACAGTAGCATCTGAGA
TTGAGATACCAATTGTCGAGTCATTTTCGACAACTGAATTGCCGGTTAGCGTTAGACATGAAATCGGCACAACTTCAAGT
TCAAAAGATTTTTCTTTCGAGAAGCGACACATCGGTAGCAATTAA

>MtMLO2 Medtr5g022400
ATGGTGGAAGAAAATAGAGAAGAAGGTAGATCCTTAGCCGAGACCCCTACTTGGGCTGTTGCAACTGTCATCACTCTTTT
GGTTTCTCTTAGTTTCTTGTCTAATGGCACATTGAAAAAATTGGTTAAGTGGTTGGGTAGAACTAAGAGAAAATCCCTTC
TCTCCGCTTTGGAAAAAATGCAAGAAGAATTGATGCTTTTTGGACTTCTGTCATTGCTGATGGGCCATTGGATTATTTTT
GTGGCAAAGATTTGTGTCAAGTCATCAGTATTAAGCAGCAGATTTTTTCCATGTGCACTGAAAGAGAGTTTTGGAATTGT
TAAACATAATGTGTGGTCCAATTTAGAATATTCAAATAAAACAATTCTCAAAGAGCAAGTGAACAATGATGGTCTGCATA
ATTTCTGTCCTAAGGGTCATGAATCATTTGCTTCTTATGAAAGTCTTGAGCAGCTTCATCGCTTTGTGTTTGTTCTTGGG
ATCACTCATGTTTCCTATAGCTTTGTTGCTGTTGCACTAGCCATGATCAAGATATATAGCTGGAGAACTTGGGAAAATGA
AGCAAAGACTATGGCTATACAAAGCTTACAAGATACTTCACAAAGTACTTCAACTTCAAGCATAAGACTGAAGCGGCTTA
GTACATTCATTTTTCACCAAGCATCTCATCCTTGGAGTCATCACAAAATTCTTGTTTGGCTGCTATGTTTCAGTCGCCAA
TTCTGGAGTTCCATCAGTCGCACTGATTACATGGCATTGCGCTTAGGCTTCATTACTACTCATGACCTTCCTCTAACATA
CGATTTCCACAACTACATGCTTCGAAGCATGGACGAGGAATTCCGTGACATCGTAGGTGTTAGTGTGCTTCTCTGGATAT
ATGCCATATGCTGTATTTTTCTAAATTTTCATGGAAACAACTTTTACTTTTGGCTTTCCTTTGTCCCGGCAATTTTGATC
CTCATAATCGGTACTAAATTGCATCGAGTCGTGGTAAAATTGGCTGTTGAAGTAATAGATCATAGTCCGTATATGAAGCC
TCATCGGTTTAACTTAAGAGATGAACTCTTTTGGTTTGGAAAACCAAGGCTTCTATTGAGATTAATACAATTGATATCCT
TCTTGAATGCGTTTGAAATGTCAACATTCTTATGGTCCCTGTGGGAAATTGATGATCCTTCTTGCTTCATGGACAACCGA
ACATTCATTGCGATCCGCTTATCGTTTGGGGTCGTCTCGCAAGTTTGGTGCAGCTTCATAACTTTTCCTCTGTATGTTAT
CGTCACACAGATGGGATCAAGATTCAAAAAAACAGTGATATCTGAGAGTGTAAGGAAATCATTATCTAAATGGCAGAGAA
GAGTGAAGGAGAAACAAAGTTCCTCTTATGAACTCCTTAATACATCAACAACAACATCATTGGAGTCTTTACTGCATGGA
ATGGACAATTCAAATCACTCTGACTTAATCAGTGAGATTGAGGAAGGAAGTTCCTCAGGAATAAAAGATATTTGTCATTC
TAACTTTGATGATGTGTCAATGATTCAAAGCAATGCAAAAATTCCAAGTTATGATGAAGATTACCATGACCATTATCATG
ATGTAAAACATGATTTGCCTCCCCCTCCACTGTCTTGA

>MtMLO3 Medtr2g093750
ATGGCAGGAGGAAGCGTTGGAAGAAGCTTAACTGAAACACCTACTTGGGCCGTTGCAGTTGTTTGCTTTGTTATACTTTC
TATTTCTATCTTCATTGAACACATTTTCCACATCATAGAAAAGTGGTTGAAGAAGAAGCATAAAAGTGCCTTGTATGAGT
CACTTGAAAAGATCAAATCAGAGCTAATGTTACTAGGGTTCATATCATTGCTCCTAACAGTAGGAGAAGGTTTAATATCA
AGAATATGTATATCAGAAAAAGTTGCAGCCACATGGCATCCATGTAGCAACAATGCAAATATTGAATCAGATGATGAAGA
GTTAATAGACCATGAAACCGGTGGCAGCCGGAGATTACTAGCCGCGTTGCTTGCTTCTCAAGGCGACAATCACCACCGTA
TTTTGGCTGGTGGAGGAGGTGACAAATGTGCAGAAGAGGGAAAAGTAGCATTTGTATCAGCAGGGGCCATTCATCAACTC
CATATATTTATATTTGTGCTTGCTGTTTTTCATATCCTCTACTGCATACTTACTCTGGCTCTAGGTAGAGCAAAGATGAG
AAGGTGGAAAAGATGGGAAGAGGCAACCAAGACACCTGAGTACCAATTTTCACACGATCCTGAAAGATTCAGATTCGCTA
ACGAGACTTCGTTTGGAAGAAGACACTTAAGTTTCTGGACCAAAAATCCTGTCCTCATTTGGATTGTATGTTTTTTTAGG
CAATTTGTAAGGTCAGTTCCTGAAGTGGATTACTTGACCTTAAGGCATGGATTTATGATGGCACATTTGGCTCCTCAAAG
TCACCTGAAGTTTGACTTTAGACAATACATCAAAAGATGTTTGGAAGAAGACTTCAAAGTTGTTGTTGGAATCAGTCCTC
CAATTTGGTTCATCACAGTGTTCTTCCTCCTGTTCCATACTCATGGGTGGCACTCTTATCTATGGCTACCATTTCTTCCT
TTGATTATTGTCCTATTAGTTGGAACAAAGCTGCAAGTGATCATAACTCAAATGGGTCTTAGAATTCAAAAACAAGGAAT
GGTGGTAAAGGGTGAGCCAGTGGTGCAACCTAGGGATGACCTCTTTTGGTTTAACAAACCTAGACTTATTCTCTTCCTTA
TTAATTTTGTACTCTTTCAGAATGCCTTCCAGCTTGCTTTCTTTTCATGGACTGCTCTTCAATTTGGGGTGACATCCTGT
TACAATTCACGTAAAGATGGTGTTGTCATTAGAATTTGCATGGGAGTCTTCGTTCAAATCCTTTGCAGCTACGTCACTCT
CCCTCTCTATGCTCTCGTGACGCAGATGGGTTCGACCATGAAACCAACCATATTCAACGAAAGAGTAGCTACAGCTCTAC
GGAATTGGCACCACACCGCAAGGAAGAACATAAAGCACAACCGTGGATCGGGCTCTCAAACCCCATTTTCAAGTCGGTCC
ATAACTCCGGCACGTTCAATGTCTCCGGCCCAAATCCTTCGTCATTACCGCAACCAAATGGACACTCCAACAAGACTCAA
TTTTGAGACCAGTCACCACTATGAATCTTATTCACCCTCACCTTCCAACTCACACCACCACAAGGTTGAGATCAATGTTG
CTTCTTCCAGCTCAACTCATCTTCATGAAATGGAAATGGGTCACCTAGCCCATGTTGAACAACAAGAAGTCATTAAGCCC
AATAGTATTTCTGTGGGCTCAGGCCGGCCTCAATTTGAAATTGATATCCAACAGAGTGATGAACTCTCATTTTCAACAAT
GCCCACAAATCAATTAGAATGA

>MtMLO4 Medtr2g028470
ATGGCAGAAGAAGAACTGAACGAGTCTCTTGAATATACACCAACATGGATTGTTGCAGTTGTTTGCTCCATCATTGTTTT
CATCTCTCTGTGCGCTGAGCGTGCCCTTCACCGCCTTGGAAAGTACTTAAAGAGGAAAAAGCAGAAAGCATTATATAGAG

CCTTGAATAAATTGCAAGAAGAGTTGATGCTTTTAGGGTTCATTTCCCTTCTCTTAACCGTCTTTCAAGGTGTAATAAGT
CACATTTGCATCTCACCTGAGCATGCAACCCAAATGCTTCCATGCAAGAGGTCTCATGAATCTGTTCAAGGTTCAGAACA
TGATCACTATGATACTATAATCAACAGAAGAAGGCTTTTATCCACAGATACTGGTTCGCAGCACTGTAGGCTAGAGGGGA
AGGTACCACTGCTATCACTGGAAGGATTGCATCACCTTCACATATTCATCTTTGTATTGGCTGTTGTACATGTAGTTTTC
TGTGTCACAACAATGATTCTTGGAGGTGCAAGGATCCGTCAGTGGAAAAGCTGGGAGGATCATGCTAGGAAGAAGACGAC
AAATTCGAGTGGTGAAACTCTTAGATCAGAGATAGACGAGTTCTTCAACAAACATGCCCAGGGGTATTGGAGAAAAGCAG
CTGTTGTTGGTTGGTTTAGATCATTCTTCAAACAATTTTATGGCTCAGTTGCAAAATATGACTACCTTGCGTTGCGACAT
GGATTTATCAAGGAACACTACCCGAACGATCCCAATTTTAATTTTCATAGCTACATCACGAGGACACTTGAAGTTGACTT
CAGAAAAGTTATAGGCATAAGTTGGTACCTGTGGCTCTTTGTTGTGCTTTTTTTGCTGTTGAATCTTGAAGGGTGGCACA
CTTATTTCTGGTTAGCCTTTTTACCATTAATAATTCTACTTCTTGTGGGGGCAAAGTTAGAGCACATAATCACTCGATTA
GGCCAGGAGTCGGTAGCGAAGGAATATCCAACTGAACGAGTGAAGCCATCAGATGAATACTTTTGGTTTAACCGCCCGGC
CATTGTCCTCGACTTACTTCACTTCACTTTGTTTCAGAACTCTTTTGAGATCGCGTTTTTCTTTTGGATTTGGTCCACAT
ATGGATTTGATTCATGCATTATGGAGAAAGTAGCCTACATCATCCCAAGAATTGTAATGGGTGTAATTGTTCAAGTGCTT
TGCAGTTACAGCACCTTGCCTCTTTACACTCTTGTCACTCAGATGGGTAGTAGGGGTAAACTTGACAAGGTTGATGATAA
GGCGGAAATGGAATCGTCTCCCTTGTTCCACAGAATGACCAAAGAATCAAACCAACATTCTCAAATTGGTGAACAAGCGA
TTATCATGATGGAAGATCATGCAATATCATCTACAATTGAACTTCACCCTATTATTCAATCTCCTTTGGAAAAGACCTAA

>MtMLO5 Medtr3g115960
ATGGGAGGAGGAGGAGGAGGCGGTGACTCCAGACAGCTAGATGTGACTCCTACATGGGCTGTCGCCGCGGTTTGTGCGAT
CATCGTTATCATTTCCATACTCTTAGAGAAACTCATACATAAATTCGCATCGGTGTTTGAAGAGAGAAAGAAGCATGCTC
TCCTAGAAGCTCTTGAAAAGATTAAAGCTGAGCTTATGGTTTTGGGATTTATTTCATTGCTATTAACATTTGGACAAAAC
TACATATCAAAAGTGTGTATTCCAGTAAAATATTCAAATACTATGCTACCATGTCAACCCCTGGCTGAAAGGACAGCTGA
TCATCCAGGCGAACCGGCACTTGAGCCTCAAGGGACTGAACACGAGCCTACACCGAACATACCGGCTGGAGAGGGTGAGT
CTAAAGGTGAACATCATCGCAGGCTCTTATCATACGAACGTAGGTTTCTCGGCGGTGGTGGTGGAGGGCCGGGATGTAAA
CCGGGGTTCACGCCTCTTATATCAGTGGACGGGTTACATCAGTTACATATTTTCATTTTCTTCTTGGCTGTCTTCCATGT
GATATTCAGTGCCATTACAATGACACTTGGTAGAGCAAAGATTAAAGGTTGGAAAGAATGGGAGCAAGACCATTTGGTCG
ATGAAGATGCCTTGAATGATCCTAGAAGATTCAGGCTTACTCATGAGACATCCTTTGTGAGGGATCACAACAGTTTCTGG
ACAAAAACACCTGTTACTTTCTATTTTGTATGCTTCTTTCGACAGTTCTTAAGATCGGTTCGAAGGGCAGACTACTTAAC
AATGCGACATGGATTTGTTTCTGTCCATTTAGCACCTGGAAGTAAGTTTGATTTTCAAAAATATATCAAGAGGTCATTAG
AAGATGACTTTAAGGTAGTTGTAGGAATCAGTCCAATACTTTGGGCATCAGTGGTGTTATTTTTGCTTGTAAATGTCCAC
GGTTGGCATGCTTCATTTTGGGTGTCTTTTCTTCCACTAGCAGTGATTCTAGCTGTTGGAACAAAGCTTCAAGCAATTAT
AACTAGAATGGCACTCGACATACAAGAAAGACATGCAGTAGTGCAGGGGATACCTCTTGTACAAGTCTCTGACAAGTATT
TTTGGTTTGAATGGCCTCAATTAGTTCTTTATCTCCTCCATTATGTCCTCTTTCAGAATGCATTTGAGCTGACCTTTTTC
TGGTGGACATGGTATGAATTTGGGTGGGCATCTTGCTTTTATGAAGATGACAGTCTTATGATTGTTAGAGTTGCTCTTGG
GGTGGGTGCTCAATTTGTTTGCAGCTATATCACACTTCCTCTCTATGCACTCGTCACACAGATGGGATCAACGATGAAGC
GGTCAATATTTGACGAGCAAACATCAAAGGCATTGCAAAACTGGCGTAAAAATGCAATGCATAAGAAACCATCAAAAGGA
CGCTCGAAGGAAACTCGAACATTAGGAGGAGGAGAAGCGGGGTCACCTGTGCATTCGCCGTTACATGATGATGGCTCTGA
AACCGAATTGCAGGAGCATAACACAGCTACTATAGTGACCAGTGTGGATCGTAATGATCACTATGATAACCATGACCTCC
TAAGTGGACCGTAA

>MtMLO6 Medtr5g024700
ATGTTCAAGAGGGTATTTTCTTGTTTGTGTCTCTTCTATGGAGGATTATCCATGGCTGCAAGTGATAGTAGGAACAGTTC
AAGAACACTTGATCAAACACCAACATGGGGTGTTGCTGCAGTTTGCACCGTTTTCATCTTGATATCCATAGCCCTTGAGA
AGAGTCTTCACAAATTAGGAACGTGGTTAGGAAAAAGAGAAAAGAAGGCTCTGCTTGAAGCTCTAGAGAAGGTCAAGGCT
GAGTTGATGATTTTAGGATTCATTTCACTGTTATTAACTATTGGACAGAATTACATTGTCAGAATATGTATTTCTGAAAA
AATTGCAAACAAAATGCTACCATGTCCACATAAATATATAGGTGGTGATAAGAAATCAAATAGTAAAGTGGAACAAAATA
GGATACCAGTGTCTTTTGAAGGTAGATATTTAGCTGCTGATACTCCCTCCTTCAAATGCAGCAGGAAGGGACATGAACCA
CTTTTATCTGTGAATGGACTGCATCAGTTACACATCCTCATATTCTTCCTAGCAGTCTTTCATGTGTTTTACAGTGCTGT
CACAATGCTTCTTGGAAGACTCAAGATACGTGGATGGAAGGAATGGGAGGAAGAGACTTTAACTCATGGTTATGAGTTTG
CGAATGATGCTGCAAAACTCAGGCTCACTCATGAAACATCATTTGTTAGAGCTCATACAAGTTTTTGGACTAGGGTTTAT
ATTGGCTGTTTCTTTAGACAGTTTTATAGGTCTGTTCGTAAGGTCGATTACCAGACTTTGCGCAATGGATTTATTAACGT
CCATCTAGCTCCCGGAAGTAAATTTAATTTCCAAAAGTATATCAAAAGATCATTAGAAGATGACTTCAAAGTAGTTGTGG
GAGTAAGTCCTGTTCTCTGGGCATCAGCTGTAGTTTTCTTGCTCCTAAACATTGATGGATGGCGTACTCCAATATGGGCA
TTCTTGATTCCAATTATTATAATTTTGGCTGTTGGAACAAAACTTCAAGTGATATTAGCAAGAATGGCTCTTGAAATCTC
AGAGAGACATATAGTTGTACAAGGAATGCCTCTTGTTCAAGGCTCAGATAGATACTTTTGGTTAGGGCAACCTCAATTAG
TTCTTCATCTTATCCATTTTGCTTTGTTTCAGAATTCATTCCAAATAACATATATCTTGTGGACATGGTATTCTTTTGGT
CTGGAAAATTGTTTCCGTTTGGACTATAAGCTTGCCATTATAAAAGTAGCTTTTGGGATTGTGATGCTATGCCTCTGCAG
CTACATTACCCTTCCTTTATATGCTCTTGTAACACAGATGGGTTCAAGGATGAAAAAATCAATATTTGATGAACAAACAT
CCAAGGCGTTAAAGAAATGGCACATGACTGTGAAAAAGAAGCATGTAAAGCTAGGAAAGTCCACTATAGGAACCATGGAT
GGTAGCACCATTGTTGGATCAACAATGCAGTCCCCTGGACCAGCACTTCACCGTTTCAAGACTACTGGCCACTCGACACG
ATCTTCAACCATTGATGAACAAGATGAATATGAATTATCTGATAATGAGTTGATGGAAACTAACTTGATTGTAAGAGTTG
ATCATGATGAGCAAGAAGCAGAAGATAGGCATCATCATAGTGAGGGAGAAGCCAATAATGATGATTTCACTTTCTTCAAG
CATGTCATTAAAAGATTAGTTTAA

>MtMLO7 Medtr2g006280
ATGGAACATGCTGTGGAGCAAGGTCGTTCTCTAGCTGAGACACCTACATATTCAGTTGCCTCCGTTGTTACTGTCATGGT
CTTTGTTTGCTTCTTGGTTGAGCGTTCTATCTACCGTTTTGGAAAGTGGTTGAAGAAGACAAGAAGGAAGGCACTTTTTG
CTTCTCTGGAGAAAATTAAAGAAGAGCTGATGCTGCTTGGGCTTATCTCTTTGTTGTTGGCACAAAGCGCGAGATGGATC
TCTGAAATTTGTGTAAATTCATCACTTTTCAGCAGTCGATTCTATACCTGCTCTGAACAAGACCTTCCCATGATTGAGCA
TGTCATGATTGTAAACTCTTCATCTTTCTCTGATGAAACTACTATTCCTAAGGGGCTATATAGTGGTGCACTCCATCAAT
GTGGAGAGGGTCATGAACCATTTGTTTCATATGAAGGTCTTGAACAGCTGCACCGGTTTTTATTTGTTCTCGGGATCACT
CATGTTCTGTATAGTTGTCTCGCTGTTGGTTTAGCGATGAGCAAGATCTATAGTTGGTGCAGATGGGAAAATCAGGCTAC
CATGGCTCTTATTGATGGAAATTTGCAAGGTAAGAACAACAAAGTGATGAGGCGGCAAACAACCTTTGTTTTCCATCATG
CATCTCATCCCTGGAGCAGGAATCCTATACTGATATGGATGCTATGTTTTCTGCGTCAGTTTAGGAGTTCTATACAAAAA
TCAGATTACCTTGCACTCCGTCTTGGTTTCATCACTGAACATAAATTGCCATTATCATATGATTTTCATAAATATATGGT
TAGAAGCATGGAAGACGAATTTCATGGCATTCTAGGAATAAGCTGGCCGCTTTGGGTTTATACTATAGTCTGCATCTTTG
TGAACATCCATGGTCTGAATATATACTTCTGGCTCTCCTTCATCCCTGCAATTCTTGTGATGTTAATAGGTACAAAACTT
CAACATGTTGTATCCATTTTAGCACTTGAAATCTGGCAGCAGCAGGGTCCTAGTGCCAGAACACAAGTAAAGCCGCGCGA
TGGTCTTTTTTGGTTCAAAAAACCAGATATATTGTTATGGATCATACAGTTTGTGATATTTCAGAATGCCTTTGAAATGG
CCTCATTTATATGGACATTGTGGGGATTTAAAGAAAAATCATGCTTCATGAGACACCATTACATGATCATCATTCGGTTG
ACATCAGGCGTTCTTGTTCAGTTTTGGTGTAGCTATATGACAGTGCCCCTGAATGTAATAGTTTCACAGATGGGATCGCG
ATGCAAGAAAGCTCTAGTGACGGAGAGTGTTAGAGATTCCTTACACAGTTGGTGTAAGAGAGTAAAACAGAAGTCTAAGC
ATGATCATTCGCTGCATTCACATACTGCTAGATCAATATGTTCCTTAGAATCAACGATCGATGAGAGGGACGAGATAACA
GTGGTATCCGGCACGCTAACTCGAACAACTTCCTTAGACTTAGAGTCTTTGAATCAGATAACAGTAACTTCAGTAGACCA
GCTGAATTTTTCTAACAATCTTCAAGATTCAACCGATCTGTCCGAACCCGTACATATTAATTCGCACTACAACGATGTTA
TCAACGGAGAGGAGGCTAAAGTTGAGACTCTTCTTGACTTGTTTCACAAAACATGA

>MtMLO8 Medtr3g115940
ATGGCCGGAGGAGCTCCTGGGGAAAGAACATTGAAGGAAACACCAACTTGGGCTGTTGCTGCTGTCTGCGCAGTGTTCGT
CATCATCTCAGTATTAATTGAACATGGAATCCATAAACTTGAAAAGTGGTTTCACAAAAAACATAAGAAGGCCATGAGTG
AAGCCTTGGAGAAAATCAAAGAAGAATTAATGCTGTTAGGATTCATATCCTTGTTACTTACATTTGGTACAAAATATATT
GCAAAGATATGCATCCCTAATAAGTTAGGTGATAGCATGCTTCCATGTAAGAAGGGGGAAGTTGAAGAAGAGTCAAAAGA
TGATAGGAGAAGGTTACTTTCTTTTGATTTTGATGAGAATGTGGTATGGCGTCGAGGTTTGGCACCTGCAGCCGGTGGTG
ATGACTATTGCTCAAATAAAGGTCAAGTGCCTTTAATATCTCAATCTGGGGTGCACCAATTGCATATATTTATATTTGTG
CTAGCCGTTTTTCACATTTTCTACAGTGTCATGACTATAGTTCTAGCTCGAGCAAAAATGCAGAAGTGGAAGGCTTGGGA
AACAGAAACATCATCCGTGGAGTACCAATTTACACATGATCCTTCAAGGTTCAGGTTTGCGCATCAAACTACATTCGTTA
AGCGTCACTCAGGCTGGTCTGAGAAGCCAGGAATAAGATGGATAGTGGCATTCTTCAGACAATTTTTTGCGTCGGTGAGT
AAGGTGGATTACATGACTATGCGGCATGGGTTTATTAACGCACATTTTGCTCCTGATAGCAAATTCGACTTTCATAAATA
CATCAAAAGATCAATGGAGGACGATTTTAAAGTGGTCGTTGGTATAAGTGTTCCTTTATGGGCTTTTGCGATCATTTTTC
TGCTTATGAACGTTTACAATTGGTACACGCTCACTTGGTTATCATTAGCGCCGCTGGTGATACTTTTATTAGTGGGCACA
AAGCTTGAACTGATAATCATGGAAATGGCTCAAGAAATCCAAGATCGTACTACAATTGTAAGAGGTGTGCCCGTGGTAGA
GCCCAACAACAAGTATTTTTGGTTTAATCGCCCCCATTGGATCATCTTCTTGATTCATTTTACCTTATTCCAGAATGCTT
TCCAAATAGCCTATTTCTTGTGGACTTGGTATGAGTTTACAATCACATCTTGCTTCCATGAGAACTTGCCACTAATAGTG
ACAAGGGTTGTCCTTGGGGTAGCCTTACAAGTCCTATGCAGTTACATAACATTTCCTCTCTATGCACTAGTGACACAGAT
GGGATCTCACATGAAGAGAGGAATATTTGAGGAGCAAACAACAAAAGCACTTAAAAAATGGCAAAAGGCAGCAAGAGATA
AGAGGAAGTTAAGGAATGCAGGATCAATAGAGATTCCTTCAGTGAGTGGAGACACAACACCAAGCCAAGGAACATCACCT
TTGCACTTGCTTCACAAGTTCAAACCTAGTAACCAAACAGACACAGATAGTGTTCTATATTCTCCAAGATCATACCAATC
TGAAACTGATTTTTCAGACACAGAAGGATCTACACACCAATTAAATTTGAATCAAATTATGTCACCACCTCAACACCATC
CTGCAGGGAACAATCAACAAAATCATAATATTGACTTCTCATTTGATAAGCCTTAA

>MtMLO9 Medtr3g118190
ATGAGTGGTGGAGGTGAAGGAGGAGAAGAAGAAAACACACTTGAGTTCACACCGACGTGGGTAGTTGCCGGTGTATGCAC
TGTGATTGTTGCCATTTCACTTGCGGTGGAGCGTCTTCTTCACTATGGTGGAAAGTTCCTTAAAAGTAAAGACCAAAAGT
CACTTTACGAAGCTCTTCAGAAGATCAAAGAAGAGTTGATGCTTTTGGGTTTCATTTCTCTTCTTCTTACTGTTTCTCAA
AATCGTCTTACCAAAATATGTGTTCCTCCCGGTGTATTGCGTCACATGCTTCCATGTAGTCTTGAGGACAAAAAAGAATC
ACATTCGCATTCAGCTTTCTCTTTTCCTGGTAGAATTGCAAGACGTCTTCTCGCAGATCGCCTCCTTGCAGAATCGGAAT
CAGCAGAAGAACCTCTTAAAACTGGTTTCTGCGGTCGCAAGAACAAGGTTCCTTTATTATCTGTCGAAGCTTTGCATCAC
CTCCACATCTTCATTTTTGTTCTCGCTGTTGTCCACGTCACTTTCTCCGTTCTCACTGTTGTTTTTGGTGGCGCAAGAAT
ACGTCAGTGGAAGCACTGGGAAGACTCTATTGCAAAGGAGAATTATGATACTAACAAAGTTCTCAAACCAAAGTTGACTC
ATGTTCAACAACATGAGTTTATCAAGGGCCGCTTTTCTGGTTTCGGCAAAGACTCTGCTCTCATTGGTTGGTTGCAATCC
TTTTTCAAGCAATTTTATGGCTCTGTGACAAAATCAGATTATGTCACATTAAGACTTGGTTTCATTACGACCCACTGCAA
GACAAACCCAAAGTTTAATTTTCACAAGTACATGATTCGTGCCCTTGAAGATGATTTCAAGCAAGTTGTCGGCATAAGTT
GGTATCTTTGGCTCTTTGTGGTCATCTTCTTGTTGTTAAATATCAATGGTTGGCATACATATTTCTGGATTGCTTTCGTT
CCTGTTATTCTTTTACTTTCCGTGGGAACAAAGCTGGAGCATGTAATAACCCAACTAGCTCATGAAGTAGCTGAAAAGCA
TGCAGCCATAGAAGGTGACTTAGTTGTACAACCATCAGATAATCACTTTTGGTTTCATCGGCCTCACATCGTTCTCTTCT
TGATTCACTTTATCCTCTTCCAAAATGCTTTTGAGATAGCATTTTTTTTCTGGATATGGGTTACATATGGATTTGACTCC
TGTATAATGGGACAAGTTCGTTACATTGTTCCAAGGCTCGTTATTGGGGTATTTATTCAGGTACTATGTAGCTACAGCAC
CCTACCACTCTACGCAGTTGTTACACAGATGGGAAGTCACTTTAAGAGGGCAATATTTAATGACCATGTGCAAGTGGGCA

TTGTTGGCTGGGCAGAGAAGGTAAAAAAGAAGAAAGCACTAAAAGCTGATGGCCAGCCTAGCCAAGGAAGTTCTCATATT
CATGAGGGTAGTACTGGTAGTAGTACTGGAATTCAGCTTGGTTCAGTTTTCCAGAAGAGGGCATCAGCTCCCGAAGACAC
TACTTCTGTAACTAAACCTGAGGGGTCCAACTGA

>MtMLO10 Medtr8g073130
ATGTTTATGTCGAAGTACAAGCAAAGGTTGTTTTTATGTCTGGTTTTATTTGCATGGTTGTACAATGGAAGTGTGGCTAT
GGCGGCTTCAAGTGAAGACAGTGGAAGTTCTAAAGACCTTGATAAGACACCAACATGGGCTGTTGGTTGTGTTTGTACTG
TTTTCATCTTGGTTTCTATAATTCTTGAGAAAAGTCTTCACAAAATTGGAACGTGGCTGGGAGATAGGCACAAGAAGGCT
TTGCTTGAAGCTTTGGAGAAAGTCAAAGCTGAATTGATGATTTTAGGTTTCCTTTCTTTGCTTTTGACGTTTGGACAGAG
CTACATGGTTAAATTATGCATGCCTGCCAATGCGACAGACATAATGCTGCCATGTCAATCTAGAGCTACCCGCGATGCAA
AAAATGATGAAGATGAACATCATAGGAAACTTCTCTATTATGAGCGCAGATATTTATCAGAAGATGGTGCCCCTTACAAC
TGCAAGGAGGGACAAGAGCCCCTTATATCTGTCAATGGATTACACCAGTTACACATCCTCATATTTTTCTTAGCCGTCCT
TCATGTGATATACAGTGCTGTCACAATGCTTCTCGGGAGACTAAAGATACGAGGATGGAAGGCATGGGAGGCAGAGACTT
CGTCTCACGGATATGAGTTTGCCACTGATCCTTCAAGATTCCGTCTTACACATGAAACATCATTTGTTAGACAACATGCC
ACTTTTTGGACTAAAATTCCAATCTTCTTCTATATTAGTTGCTTCTTTAGACAGTTCTATAGGTCTGTCGAGAAATCTGA
CTACCTGACTCTGCGCAACGGATTTATCGCGGTCCACCTCTCACCGGGAAGTAAATTTAACTTTCAAAAATATATCAAAA
GATCCTTGGAGGATGACTTCAAGGTGGTTGTTGGAGTCAGTCCTGTCCTTTGGGCATCATTCGTTGTTTTCCTTGTCCTA
AATGTTGATGGATGGCATGCTATGTTTTGGGGATCCTTAATTCCTGTCATTATAATTTTGGCTGTTGGAACAAAACTTCA
AGTCACATTGTCAAAGATGGCCATTGAAATCACAGAAAGACATGCTGTGGTCCAAGGGATTCCCCTTGTTCAAGGCTCAG
ACAAATATTTTTGGTTTGGGAAGCCTCAGTTGCTTCTTCACCTTATCCATTTTGCTTTGTTCCAGAATGCGTTTCAAATA
ACATATTTCTTGTGGATATGGTATTCTTTCGGGTTCATAAATTGTTTCCATGCTGATTACAAGCTTGCAATAGTCAAAGT
AGCTTTAGGGCTCGTGGTGCTAGTCCTCTGCAGCTACATCACCCTTCCATTATATGCTCTTGTTACTCAGATGGGATCAA
CAATGAAAAAATCAATATTTGATGACCAAACATCAAAGGCGTTAAAAAAATGGCACAATACTGTGAAAAAGAAGCATGGA
GTGAAACTAGGAAACTCGAGCGTGCGAACCAGGGACGGAAGCACCACCGATTCAACAGTGCACTCTATTGGTCCCACTCT
ACACCGTCACAAAACTACCGGTCACTCAACTCGCTACGAAAACGACCAAGATGATTACCAATCTGATATTGAATTGTCTC
CCACATCACCAACATCAAACTTACTAGCAGTAAGAGTAGATAATCTTGAGCAAGATGCAAAAGAAAATGAACACCAAGCC
ATCTTAGAAACCATTGAACAACAACAAACCGTTCTACGTAGCTTGTCATTTGTCAAGCCTGATCATCCGGAAAGAAGCGC
GAATTAA

>MtMLO11 Medtr4g079140
ATGGCGGAAGGAGAACAAAGATCTCTTGAAGAAACCCCAACATGGGCTGTCTCAGTTTGTTGCTTCTTTGTTCTTGTTAT
ATCATTGATCATTGAAGGAGGTCTCCACAAATTGGCTGAGTTTTTGAGGAAAAAGAAAAGAAAATCCATGGGGAAGGCTT
TGACCAAAACTAAAACAGAAATGATGAAACTCGGTTTCGTGTCATTTCTTCTGACAATATCGGAAGTGCCAATGTCGAAA
ATTTGCATCAACAAGGATGTAGCAAATTCTTTCAGGCCATGTAAAGATTCTGAATACTTAGCAGGTGAAGTTTCAAGAAA
AGAATTGGGATACCACGACAACTATTGTGAAGCAAAGGGCAAGGTAGGGCTAATTTCAAGTGAAGGAATCCTGGAGCTAA
ATATCTTAATCTCCATTTTGGCAGTCTTTCATATCCTTTACTGCACATTAACGATGTGTCTTGGGAAGGCTAAGATGAGA
AGATGGAAAAAATGGGAGGAGGAGACTCAAACACTTGAATATCAGATAGACAATGATTCAAGAAGATTCCAGTATATAAG
TCAAACATTGGGTGGCAAGCGTCATTTTAAATTCTGGAATAATTATTCTCCCTTGCTGTGGATTGTCTGTTTTATGCGGC
AGTTCTATAATTCAGTCTCAAAAGATGATTACTTTGCTCTACGAAATGGGTTCATTGCAAAAAATATTTCTGAAGGCAGC
AATTTTAATTTCAACTTCAAGAAATTCCTTTCCCGCACTTATGACGAAGATTTTGAGAAGGTTGTTGGAATCAGGTTTTG
GATCTGGCTTTTTTCCATATTTTTCATATTCCTTAGCGCACACGAGTTTTATAATTACTACTGGCTTCCATTTATTCCAC
TTGTGATTGCTCTTCTTGCGGGGACTAAACTGCAAGTAATAATAACAAAGATGCACCTTGATAGTTGCAAGCAAAGCCCT
GTGATCAAAGGAACCATACTTGTAAAACCAAGTGATGAATATTTTTGGTTTCATAAACCTGAATGGCTTCTCCATTTAAT
CCAAATCATCCTTATCCAGAACTCCTTTCAACTTGCATTTTTCACTTGGAGTTGGTATGAATTTGGTCCAAGGTCTTGCT
TCAATAGAGAAAAAGAGGACATTGCCATAAGGATTGCATTGGGTTTAGCTGTTCAGCTTCTTTGTGCTTATGTCACTCTA
CCCCTTTATACACTTGTCACTCAGATGGGGTCTAACATGAGAAAGGAAATTTTCACTGAGCATATTACTCAAGGTCTAAA
AAATTGGCATAAAAAGGCTAAGCAATCTCTTTCCAAAAGCAACAACAATTCAGTTTCTTTACATTCCAGAACAAGTGAGA
ATTCAGTAAGAAGATCTTTATCTATAGAGAGTATGCAGAATAACAATAAGGTGGAAAATGAAGACATGCAATCAGTAATA
GTGGTGACTAGTCCTCCTCCTGGTAACAATTCTGGAAGTGGAGAAGAGGAAGAGATTGTTAATGTAGCGAGTGTTGAGCA
AGAGATATCAAGTGAATCAACTTCAGAAATTAAGAATGTTGAGGAAAATAATGGTAAGATCATCACCAGAGGAATCTATG
ACGGTGAGATCTCCTTTGGAGTTTATGTGGGTAGTAGTAGCAGAGGCATAGGGGAAATTGTTTCAATAGCTGAAGAAGAA
GATGATGATACTAGTGATCAAAATTAA

>MtMLO13 Medtr8g010500
ATGGGTCATGGTGGAGGACAAGTTGAAGAAGGACCCGATTTGCAATATGCTCCTACATGGATTGTTGCCCTTGTTTGCAG
CGTCATCGTCGCAGTTTCATTCGCCGCTGAATGTTCTCTTCATTGTCTTGGCAACTTTCTTAAGAAGAAAAACCAAAAAC
CACTTTATCAAGCCCTACAGAAAATTAAAGAAGAGTTGATGCTGTTGGGATTCATTTCTCTATTTCTGACTATAACACAA
AATGGGATAACAAAAATTTGTGTTCCTGATGAGTGGACTTATCATATGCTTCCTTGTAGTCTTGAGGAGATAGAAGAAGA
AGAGATGAAAAAAAATTCACATTTTCAGACTTTTTTCTCTTCGGATCTTGTTTCTGGCACTGCTCGGCGCCTTCTTGGTG
GCGGTGGCGGTGGTGATGATCCATTCGGATTCACGGATGAAAAAGTTGGATACTGCACTGCCAAGGGAAAAGTACCTCTA
TTATCTGTGGAAGCATTGCATAAACTGCATATCTTTATTTTTGTCCTAGCTATTGTTCATGTGACTTCTTGTGTTTTGAC
TATTATTTTTGGAGGACTAAATATTCGTCGATGGAAGCCCTGGGAAAATTCTATTGTTGCAGATGAAAACCATGAATCAC
AACATGCACCCGAATGGACGGATACGGTGACTCATGTTCATCAGCATTCTTTTATAAAGAATCGTTTTACTGGTTTTGGC
AAAAATTATGCTATAATGGGATGGGTGAAATCATTTTTCAAGCAATTTTATGGATCAGTGACAAAGTTGGATTATGTGAC

ATTAAGGCGTGGTTTCATTATGACTCATTGTAGGGGAAATCCAAAGTTTAATTTTCACAAGTACATGATCCGTGCACTTG
AAGATGACTTCCAAAACGTTGTTGGTATTAGTTGGTATCTTTGGATCTTTGTGGTCATATTTATGTTGCTTAATATCAAT
GGTTGGCACACATACTTTTGGGTTGCTTTCATTCCTGTCATTCTACTACTTGCTGTGGGAACAAAGCTTGAACATGTAAT
AATTCAATTAGCACATGAAGTGGCTGAGAAGCATTCAGCCATACAAGGCAAATTAGTTGTTCAACCATCAGATGATCACT
TCTGGTTTCATCGTCCTCGCATCGTCCTCTTCTTGATTCATTTGATCCTTTTCCAAAATTCTTTTGAGATAGCATTCTTT
TTCTGGATACTGGTAACATTTGGCTTTAATTCATGTATGATGGGAAAACTTCATTACACTATTCCAAGGCTCGTTATTGG
GTTATTGATTCAGGTCATATGTAGTTATAGTACCCTACCACTCTATGCAATTGTCACACAGATGGGAACTTACTATAAGA
AAGCCATATTTGATGAGCAAATACAAACATGTCTTCTTGGTTGGGCACAAAAGGCAAAGAAGAAAGGATTAAGAGGTAAT
AATAACCAATCTGGTGAAGGAAGTACTCACAGTGGTGCAAGCAGTAACATTCAGTTAGGATCTGTGTTTAGAAGGGCACC
TGCTCCAGAAGACAACACCATTGTCCCTAAAGATCAACAGTCTGTATGA

>MtMLO14 Medtr8g010530
ATGGGTCATGGTGGAGGAGAAGCTGAAGAAGGAAGAGATTTGCAATTTACACCTACATGGGTTGTTGCTCTTGTTTGCAC
TGTCATCGTCGCTGTTTCATTCGCGGTGGAACGTTCTCTTCATTATCTTGGCAAGGTTCTTAAGAAGAAAAATCAGAAAC
CACTCTTTGAAGCCTTACAAAAAATCAAAGAAGAGTTGATGATTTTGGGTTTTATTTCACTGTTTCTAACGGTAACACAA
AATGGGATGATAAAAATTTGTGTTCCGGTAGAATGGACTATTCATATGCTTCCTTGTAGTATTAATGGGGACGATGAAGT
CAAAGAAACAAAATTACCATCCAAGTCACATTTTCAGACTTTTTTCTCTTCCAAGGATGTTTTTGGCACTGCTAAGCGCC
TTCTAGGTGACGATCACGATCATTCGTCCAAAGATGAAGAAATTGGATACTGTACTGCCAAGGGAAAGGTGCCTCTATTA
TCTGTGGAAGCATTGCATAAACTGCATATCTTTATTTTTGTTCTAGCTGTTGTTCATGTGACTTCATGTGTTTTGACTAT
TGTTTTTGGAGGATTAAATATTCGTCGGTGGAAGCGCTGGGAAGACTCAATTGTTGCAGATGAAAACAATCAATCACAAA
CCCCCGAACGGACGGATACGGTGACTCATGTTCATCAACACGCTTTTATCAAGGATCATTTTACTGGTTTTGGCAAAGAT
TCTGCCATAATGGGGTGGTTGAAATCATTTTTCAAGCAATTTTATGGATCTGTGACAAAGTTGGACTATGTGACATTAAG
GCTTGGTTTCATTAAGACTCATTGTAGGGGAAATCCGAAGTTTAATTTTCACAAGTACATGAACCGTGCGCTTGAAGATG
ATTTCAAGAAAGTTGTCGGTATTAGTTGGTATCTTTGGATCTTTGTGGTCATATTTTTGTTGCTTAATGTCAATGGTTGG
CACACATATTTTTGGATTGCTTTCATTCCTGTCATTCTTCTTCTTGCTGTGGGAACAAAGCTTGAACATGTAATAATTCA
ATTAGCACATGAAGTAGCTGAAAAGCATTCAGCCATACAAGGTGAATTAATTGTTCAACCATCCGATGATCACTTCTGGT
TTCATCGTCCTCGCATTGTCCTCTTCTTGATTCATTTGATCCTTTTCCAAAATTCTTTTGAGATAGCATTCTTTTTTTGG
ATATGGATACAATATGGCTTTAACTCATGTATAATGGGACAAGTCAAGTTCATTTATCCAAGGCTTGTTATGGGGGTATT
TATTCAAGTGCTATGTAGTTATAGTACCCTACCATTGTATGCAATTGTTACACAGATGGGAACTCACTTTAAGAGATCCA
TATTCGACGATCAAGTACAAACACGACTTATTGGTTGGGCACAAAAGGCAAAGAAGAAAGGATTAAGAGGTGATAGCAAC
CATTCTGTCCAGGGAAGTTCTCATAATGGTGGAAGTACTAACATTCAGCTAGGGTCGATGTTCAGAAGAGGACCTGCCCC
AGAAAACAATGCCTTTGTCCCTAAAAATGATGAGTCTGTTTGA

>MtMLO15 Medtr2g079500
ATGGCAGAAACTGTAAAAGAACCAAGGTCATTGGCCTTGACTCCTACCTGGTCCGTCGCCACTGTGTTGACCATCTTTGT
TGCCGTCTCTTTGCTTGTGGAGCGCTCCATTAATCGTTTAAGCAATTGGCTGCGGAAAACTAATAGAAAACCTTTGCTTG
AAGCTGTGGAGAAGATGAAAGAAGAGTTGATGTTGCTCGGATTTATATCCCTCCTACTTACTGCTACATCTGGCATGATA
GCCAATATTTGCATTCCATCAAAGTTCTATAATAGTGCTTTTGCTCCGTGCTCTAGATCTGAGATCAATAAAGAAATCGA
AGAGAATGGCTCCGAAGGACGTAAATTATTGACGGCTTCTTATCCACGCTTAATTAGAAGGATGTTGAATGGAATGGATA
GAAACTCCTGCAAAGAGGGTTATGAACCATTTGTTTCGTACGAAGGTCTAGAGCAGTTACATCGGTTCATTTTTGTCATG
GCGATAACACATGTATCTTACAGTTGCCTAACAATGTTGTTAGCCATTGTGAAGATTCACAGTTGGAGAGTGTGGGAGGA
TGAGGCTCACATGGACCGGCGCAATTATTTATCTGAAATTACTAGGCAGATGACTATGCGAAGGCAATCGACTTTTGTCA
AGACTCATACATCAAATTCATTATTCATTTGGATGACATGTTTTTTCCGACAATTTGGGCATTCTGTGGTTCGTGCTGAC
TACCTTACACTTCGCAAAGGCTTCATCACGAATCACAACCTTTCATCAAAATATGATTTCCACAGCTACATGGTTAAATC
AATGGAAGAGGAATTCCAAAGGATAGTTGGTGTGAGTGGCCCACTATGGGGATTTGTTGTCGGTTTCATGCTTTTCAACA
TTAAAGGATCAAATCTCTATTTCTGGATAGCTATCATTCCTATATCGCTTGTTCTTTTGGTGGGAACAAAGCTACAGCAA
GTCATTGCAACCTTAGCATTGGAGAATGCTGGAATTAGTGGTTTCTTTTCGGAAGCTAAGTTGAGGCCTCGCGATGAACT
TTTCTGGTTTAAAAAGCCTGAACTATTACTGTCCTTAATCCATTTCATTCTCTTCCAGAATGCTTTTGAATTGGCTTCAT
TCTTTTGGTTCTGGTGGCAGTTTGGATATAACTCCTGCTTTATTAGGAATCACCTCCTTGTTTATTTAAGGCTAATCTTG
GGGTTTGCTGGGCAGTTTCTTTGCAGCTACAGCACCTTGCCACTTTATGCACTCGTTACACAGATGGGAACAAACTATAA
GGCAGCATTAATTCCACAGAGGATAAGGGAAACCATTCATGGATGGGGTAAAGCAGCTAGGAAGAAGAGAAGACATGGTA
TGTTCGCCGACGACTCCACCATTCATACAGACACAAGCACTGTATTGTCTATTGAAGAAGACTATCAAACAATCGGTGAT
CCACATAAAGACCTTGTCACTGGCACTGAAGTAGAATTACAACCAGTACCAACTGTTACATCAACCCCTTCACCTATTGC
TAACGAAACTTCAAGTCGGGCCGTTACGCCCCTCCTTCGACCCTCAGCTTCAGTATCTTCATCAGTACCATTCAATTCTA
GACAAGAAGAACCAATTCCAAGATCCTCCTCTATGCCAAGTGGAAGATAA

>PsMLO2
ATGAGTGGCGGTGGTGAAGGAGGAGAAGATGAAACAACACTTGAGTTCACTCCCACGTGGGTTGTTGCTGCCGTTTGTAC
TATAATCGTCGCCATTTCACTCGCTGCTGAACGTCTCCTTCACTATGGTGGAAAATATCTCAAACGCAAAGATCAAAAAC
CACTTTACGAAGCTCTCCAGAAAATCAAAGAAGAGTTGATGCTTCTCGGTTTCATTTCTCTTCTTCTCACSGTTTCCCAA
AACGGACTCACCAAAATATGYGTTCCTCCCGGTGTATTGCGTCACATGCTTCCYTGTACTCTYAAGGAGAAGGATCAGAA
AGAGTCTGAACTTCCCAAATCACATTCCGCTTTCTCTTTCCCCGGAATCGCACGGCGTCTTCTCGCCGATCGTCTCCTAG
CAGAAGCGGAAGCAGCAGAAGAGGCTCATCCGAAAACCTCTTTCTGCTCTAGCAAGAGCAAGGTTCCTTTGTTGTCTCTT
GAAGCTTTGCATCATCTCCACATCTTCATTTTTGTTCTCGCCGTCGTTCATGTTACTTTTTCGGTTCTCACCGTTGTTTT

TGGAGGCGCCAGAATACGTCAGTGGAAGCGCTGGGAAGATTCTATTCGAAAAGAGAATTATGATACTAGTAAAGTTCTCA
AACCAAAGTTTACTCAGGTTCAAAATCATGAATTTATCAAAGGTCGCTTTTCTGGTTTCGGCAAAGACTCTGCTTTAATT
GGTTGGTTGCAATCCTTTTTCAAGCAGTTTTATGGTTCTGTGACAAAATCAGACTATGTGACATTAAGACTCGGGTTCAT
TATGACTCACTGCAAGACAAACCCAAAGTTTGATTTTCACAAGTACATGATCCGTGCCCTTGAAGATGATTTCAAGATAG
TTGTTGGCATAAGTTGGTATCTTTGGCTCTTTGTGGTCATCTTCTTGTTGTTAAATATCAATGGTTGGCATACATATTTC
TGGATTGCTTTCATTCCAGTTATTCTTTTACTTTCTGTGGGAACTAAGCTGGAACATGTAATAACCCAACTAGCTCATGA
GGTAGCTGAAAAACATGCAGCCATAGAAGGTGACTTGGTTGTAAAACCATCAGATGGTCACTTTTGGTTTCATCGACCCC
ACATCGTTCTCTTCTTGATTCACTTTATCCTTTTCCAGAATGCTTTTGAGATAGCATTTTTTTTCTGGATATGGGTTACG
TATGGATTTGATTCCTGTATAATGGGACAAGTTCGTTACATTGTTCCAAGGCTTGTGATTGGGGTATTTATTCAAGTACT
GTGTAGCTACAGCACCTTACCACTCTATGCAGTTGTTACACAGATGGGAACTCACTATAAGAGGGCAATATTTAATGAGC
ATGTGCAAGTGGGCCTTGTTGGTTGGGCAGAGAAGGTAAAAAAGAAGAAAGCACTAAAAGCTGAAAGCCAAGGAAGTTCT
CATGATCAAGAGGGTAGTAGTACTAGTGCAGGAATTCAGCTTGGGTCAGTTTTCCAGAAGAAGGCATCAACTCCKGAAGA
CAATGCGTCGGTTCCCAAAGCTGAGGGGTCCAACTGA

>PsMLO3
ATGGCCACACAAACAGAACAAAGATCTCTTGAAGATACCCCAACATGGGCTGTCTCAATTTGTTGCTTCTTTGTCCTTGT
TATATCTTTGATCATTGAACACGGTCTCCACAAATTCACTGAGGTTCTGAAGAAACAGAAAAGAAAATCTATGGGGAAAG
CTTTGGCTAAAACTAAAATAGAAAATGATGAAATTTGGTTTTGTGTCATTTTTGCTGACAATATCGGAAGTTCCAATATC
AAAAATATGCATCAATAAGGATATGGCAAATTCATTTCTACCATGTAAAGATTCTATGGAGAAGTTAGCAGTATCATCAG
AATTAGATCAAACACCTTCTACCAATGAGTTGGGAAACCAAGTCAACTATTGTGAAGCTAAGGGAATGGTTAGTCTAATT
TCAGATGATGGAGTCCTACAGCTAAATGTCTTAATCTCAATTTTGGCAGTGTTTCATATCCTTTACTGCACATTAACCAT
GTGTCTTGGGATGGCAAAGATGAGAAGATGGAAAAAATGGGAGGAAGAGACACAAACACTTGAATATCAGATAGATAATG
ATTCAAGAAGATTCCAGTATATAAGTCAAACATTAGGTGGAAAACGACATTTTAAATTCTGGAATAATTATTCTCCCTTG
CTCTGGATAGTATGTTTTATGCGACAGTTCTATAATTCAGTCTCGAAAGATGATTACTTTGCTCTTCGAAATGGGTTCAT
CGCAGCAAACATTTCTGAAGGTCAGCAATTTTCAATTTCAACTTTAAGAAATTTCTTTCGCGCACTTACGATGAAGATTT
TGAGAAAATTGTTGGAATCAGGTTTTGGATCTGGCTTTTCTCCATATTTTTCATATTCCTTAGTGCACATGAGTTTTATA
ATTACTATTGGCTTCCATTTATTCCACTTGTGATTGCTCTACTTGCTGGGACTAAACTGCAAGTGATAATAACAAAGATG
CATCTAGATAGTTGCAAAGAAAGTTCTGTGATCAAAGGAACCATACTTGTAAAACCAAGTGATGAATATTTCTGGTTTCA
TCGACCCGAATGGCTTCTCCATTTTACTCCAAATCATCCTYATCCAGAACTCCTTTCAACTTGCATTTTTTCACATGGAC
TTGGTaTGAATTTtGGTCCAAGGTCTTGCTTCAATAGGGAAATAGAGGACATTGCTATAAGGATTGTATTGGGATTAGCT
GTTCAGCTTCTTTGTGCTTATGTCACTCTTCCCCTTTATGCACTTGTCACTCAGATGGGGTCTAACATGAGAAAGGAGAT
TTTCACTGAGCATGTGAATCAAGGTCTaAAAAACTGGCATAAAAAGGCTAAGCTGTCGCTTTCGAAAAACAGTAACTCGG
CTTCTTTACATTCCAAAACAAGTGAGAATTCAGTAAGGGGGTCATTAGAGGAGTTGCGGAATAATGAAGACATCATAACA
CCTAATTCAGCCAACAACTCTGGAAGTGGAGAGGAAAAAGAAGTAATTGTAGCTGCTGTTGAGAAAGMGATACCAAGTGA
ATCTACTTCAGAAGAGAATGGTAAGATCATCACCAGAGGAATCTATGACGGTGAGATCTCGTTTGGAATCTATGTGGGAA
GTAGTAGCAGAGGTATAGGGGAAATTGTTGCTATAGCTGAAGAAGAATAGATGATTACACTCATCACAAATTTGAATCCA
GAGGGATTGGTCTTGTAGATTGTAATATTTATTTGAATAAAACTCATTCGTTAAACAATGTCTATAATCTTTGA

>PsMLO4
ATGGCAGAGAATGGAAAAGAAACAAGGTCATTGGCCTTGACTCCTACCTGGTCTGTTGCCACTGTGTTGACCATCTTTGT
TGCTGTTTCTTTGCTTGTGGAGCGCTCGATTAACCGTTTGAGCAATTGGCTGCGGAAAACTAATAGAAAACCTTTGCTTG
AAGCTTTGGAGAAGATGAAAGAAGAGTTGATGTTGCTCGGATTTATATCTCTCCTACTTACTGCTACCTCTGGCATGATA
GCCAATATTTGCATTCCATCAAAGTTCTATAATAGTGCTTTTGCTCCATGCACTAGATCCGATATCGATAAAGAAATCGA
AGAGAATGGATCCGAAGGACGTAAATTGTTGACGGCGTCTTCTTTCCCTCGCTTAATTAGAAGGATGTTGAATGGAATCA
ACAGAAGCTCCTGCAAAGAGGGTTATGAACCATTTGTTTCGTATGAAGGTCTAGAACAGTTGCATCGGTTCATTTTTGTC
ATGGCGATAACACATGTATCTTACAGTTGCCTAACAATGTTATTGGCCATTGTGAAGATTCACAGCTGGAGAGTGTGGGA
GGATGAGGCTCACATGGATCGGCGCAATTATTTAGCTGATATTACTAGGCAGCTGACTATGCGAAGGCAATCGACCTTTG
TCAAGCCTCATACATCAAACCCATTAGCTATTTGGATGACATGCTTTTATCGACAATTTGGGCGATCTGTGGTTCGTGCT
GACTACCTTACCCTTCGCAAAGGCTTCATCATGAATCACAACCTTTCATCAAAATATGATTTTCACAGCTACATGGTTCG
ATCTATGGAAGAAGAATTCCAAAAGATAGTTGGTGTGAGTGGCCCACTCTGGGGATTTGTTGTCGGTTTCATGCTTTTCA
ACATTAAAGGATCTAATCTATACTTCTGGATAGCTATCATTCCTATATCTCTTGTTCTCTTGGTGGGAACAAAGCTACAG
CAAGTTATTGCAACATTAGCACTGGAGAATGCTGGAATTGCTGGTTTCTTCTCAGAAGCTAAGTTGAGGCCTCGCGATGA
ACTTTTCTGGTTTAAAAAGCCTGAACTATTACTTTCCATAATCCATTTCATTCTCTTCCAGAATGCATTTGAATTGGCTT
CATTCTTTTGGTTCTGGTGGCAGTTTGGATATAACTCCTGCTTTATTAGGAATCACCTCCTTGTTTATTTAAGGCTAATC
TTGGGATTTGCCGGACAGTTTCTTTGCAGCTACAGCACCTTGCCACTTTACGCACTCGTTACACAGATGGGAACAAATTA
TAAGGCAGCATTAATTCCACAGAGAATAAGGGAAACCATTCATGGATGGGGTAAAGCAGCTAGGAAGAAGAGAAGACATG
GTATGTTCCCTGACGACTCCACCTTTCATACGGACACGAGCACTGTATTGTCTATTGAAGAAGACGATCATCGAATAATC
GACGATTCCCTTCAAGACATTGCCACTGGCGTTGAAGTAGAATTACAACCAGTAACAGCTGTTACGGCCACCCCTTCCCC
TATTGCTAACGAAACTTCAAGTAGGGGTATTACGCCCCTCCTTCGACCCTCAGCTTCAGTATCTTCATCAGTACCATTCA
GTGCTAGACAAGAACCAGTTTCAAGATCCTCCTCTATGCCAAATGGAAGATAA

>PvMLO1 Phvul.001G175900
ATGAACATGGCAACAGCACCCGGATATTCTCTACAACACACTCCAACATGGGCAATCGCCACTGTTTCCTTCATTCTTAT
CTCAATCTCAATCATCTTGGAGCATCTCATTCATCTTATAATCAAATGGTTGAGGAAACACAGGAAGAGCGATTTGGTCG

AAGCAATAGAGAGGCTTCAATCAGAGTTAATGATCTTAGGATTCATGTCGCTTTTGTTAACAATAACTCAAGACGCCATA
ATAGAAATCTGCATTCCAGTGAGGGCTGCAGATACTATGCTTCCATGTCGAAAACGTACTACTACCAATGATACTGCAAT
TTTGGATTCTTGCAGTGCCAAAAATGCCAGTGAGGTTGCTTTAGTGTCAAAACATGGAATCCATCAACTTCACATGTTTA
TTTTTGTTCTGGCTGTCATGCAAATTGTGTACAGTTTTCTCACAGTTTCTCTGGCAAGGGCTAAGATGAAGAACTGGAAA
GCTTGGGATGAAGAGACTCAAACAATGGAATATGAAATTGCCAACGACCCTAATAGATTCAGATACGCAAGGCAAACAAC
ATTCGGAAGACGCCACATATCTACTAGTACGGCATCACCAGTGTTTGTTTGGATTAAATGTTTTTTCAGACAGTTCTTTC
ATTCAGTAGAAAAAGTTGATTACCTCACCCTCAGGCATGGTTTCATATCAGCTCACTTTTCAGCAAGAAACAGCAACTTC
GATTTCCAAGCTTACATTGAACAGTCACTAGAGGAAGATTTCAGAATCATAGTGAGCATTAGCCCTGTGATGTGGTTCAC
TGTTGTTATTTTTTTGCTCGTGGATGTACACGGTTGGCACGTATATCTCTGGTTATCCTATGTTCCCCTCTTCCTGGTTC
TGGTTGTGGGAGCTAAACTTGAAGTCATTGTCGATGAAATGGCTCTTCAGATGAAAGATGTCAACAGTGTAACCAAAGGG
ACCCCACTTGTTTGTCCAAGTGACGACTACTTCTGGTTTGGCCATCCAGGATTTGTGTTAACACTTCTACATTACACTTT
ATTCGTGAATGCATTCGAACTTGCCTTCTTCGTATGGGTATCAACACAGTTTGGAATAAATTCTTGCTACCACGAACACA
GAACATTTACAATCATAAGGGTGGTGATAGCGGTGGCAGTTCAAGTCCTATGCAGCTATGTTACTCTTCCCCTCTATGCA
CTAGTGGCACAGATGGGATCAGAAGTGAAGAGCAAAGCTTTGGCTAAAATTCTGAGGCAGTGGCATGTTGAAGTGAGGGA
GAGAAGGAGGAAGCAACTAAAACTTGTGAAGTCTTTTAGTTTTCGCCGCATGAATATGTCCTCAGAATGGAGCCAAGGAA
ATAAGAGTGCCCCTGAATTTTCTTCCGAACTACGTGAAAACACTCGTTCCTCTAATGAAGGAGAAATTGTGGAAGAATTG
GAACATAGGGTGAAAACAGAGGCAACCTCGTCGAATGATCCACCTAGTGTTGTCTAA

>PvMLO2 Phvul.002G107600
ATGGGGAAAGAAAATGGCGAAGGAAGATCCTTAGCTGAAACCCCCACCTATGCAGTGGCAACTGTGATCGCTGTCCTTGT
TTCTCTTAGTTTCTTGTTTCAGGGCACGTTAAAAAAGCTGGTAAAGTGGTTGGACAGAACCAAGAGAAAATCTCTGCTTT
CTGCTCTGGAAAAGATCAAAGAAGAATTGATGCTTTTTGGGCTTCTATCATTGTTGATGGGTCATTGGATTATTTTTGTG
GCAAAGATTTGCGTTAAGTCTTCGGTATTAAGCAGCAGATTTTTTCCGTGCGCATTGGAAAAGAATTCTGTGAAAGATGT
TGTCTGGTTGAGTTCAGAATATTCAAATAGAACAGTTGTTAAAGGGCAAGTGAATAATGGTTTGAGAAATTATTGCCCTG
AGGGTCGCGAATCATTGGCCTCTTATGAAAGTCTTGAGCAGCTTCATCGCTTTGTGTTTGTTCTTGGGATAACCCATGTT
ACCTATAGCTTTCTTGCTGTTGCCCTGGCCTTGATCAAGATATATAGCTGGAGAACATGGGAAAACGAAGCAAAGACTAT
TGCTGTACAAAGCATTCAAGATACTTCACAAAGTACATCAAGCATAAGATTGAGGCGGCTTAATACATTCATCTTTCACC
ATACATCCCATCCTTGGAGTCATCACAAAATTCTTGTTTGGCTGCTATGCTTCAGCCGCCAATTTTGGAGTTCCATTCAT
CGAGCTGATTACATGGCATTGCGCTTAGGCTTCATTACTAACCATGATCTTCCCACAACATACGATTTCCACAACTATAT
GCTTCGAAGCATGGATGAAGAATTTCGTGACATAGTTGGTGTTAGTGTACTTCTCTGGGTATATGCAATCTGCTGTATTT
TCCTAAATTTTCACGGTAGCAACTTTTACTTTTGGCTTTCCTTCGTTCCAGCAGTTTTGATCCTCATTATTGGTACTAAA
TTGCATCGAGTGGTAGTAAAGTTGGCTGTTGAAATAATAAATCGTTGCCCAGATATGAAACCTAACCAATTTAACTTAAG
AGATGAGCTCTTTTGGTTTGGAAAGCCCAGGTTCCTATTACGGTTAATTCAATTGATTTCCTTCTTGAATGCATTTGAAA
TGGCTACATTCCTATGGTCTCTGTGGGAAATTAAAGATCCTTCTTGCTTCATGAACAACCGAACCTTTATTGTGATTCGC
TTGTCATTCGGGGTCATCTCCCAAGTCTGGTGCAGCTTTATAACGTTCCCTCTCTACGTTATCATCACACAGATGGGCTC
AAGGTTCAAAAAGACCGTGGTCTCTGAAAATGTGAGAAAATCACTCTCTAAATGGCAGAGAAGAGTGAAGGGGAGACAAA
GCTATTCTTCTGCACTCCTTACTGCAACATCACCAACATCAATTAGTTCTTTTATCTATGGAAACGATCGTGCCTCAATC
AGTGAGGAAGGAAGCTCCTCAGGGACTAAAGATATTTGTCTCTCTTATCTTGATCATGAGTCAAGGACTCAAGCAAATGC
ATCATTTCCAAGTTATGAAAATGATCATAATGATCAGGATTCTCATGATATAATCCATCATTCATCTCCCCCTCCACTGT
CTTGA

>PvMLO3 Phvul.002G117900
ATGAATAGTTCGAAGTTTAGAAAGCTGCTTTCTTGTGTGGTAGTGGTGTTGCCTTGGTGGTTATTATGGTCTGGAGGTTC
GGCCATGGCAGCGAGTGAAAGTGGCAGCAGTTCCAAAGAGCTCGACCAGACACCAACGTGGGCCGTTGCTGTCGTCTGCA
CTGTTTTCATTTTGATATCCATAACACTCGAAAAAAGTCTCCACAAAGTTGGGACGTGGCTTGGAAAAAAGCAAAAGAAG
GCTTTGCTCGAAGCTCTGGAGAAGGTCAAGGCTGAGTTGATGATTTTGGGTTTCATTTCACTGCTTTTGACTTTCGGGCA
GAGCTACATTGTCAAAATATGTATTCCTGAAAAGATGGCAGACAAAATGTTGCCATGTCCATATAAATATAAGGATGCTG
AAAAGGCATCAAATGATGATGGAGAGAAACATCGTAGGAAACTTTTGTCTTATGAACGTAGATATTTAGCAGCTGATAGT
ATCTCGTTTAAATGCAGCAATGAGGGATACGAGCCACTTTTATCCGTGAATGGTTTGCACCAGTTACACATCCTCATATT
CTTCTTAGCAGTCTTACATGTGCTTTACAGTGCTATCACAATGCTTCTTGGAAGACTAAAGATACGTGGATGGAAGGCAT
GGGAGGCAGAGACTTCAACTCATAATTATGAGTTCGCCACTGCTGCTTCAAGATTTAGGCTTACTCATGAAACATCGTTC
GTGAGAGCCCATGCCGATTTATGGACAAGGATTCCCATTTTCTTCTATATTGGCTGCTTCTTTAGGCAGTTTTATAGGTC
TGTAAATAAGACGGATTACCTGACTTTGCGGAACGGGTTTATCACTGTACACCTGGCTCCTGGAAGTAAATTTAATTTCC
AAAAGTATATCAAAAGATCATTTGAAGATGACTTCAAGGTGGTTGTAGGAGTCAGTCCTATCCTCTGGGCATCAGTTGTA
GTTTACCTTCTCATCAATGTTAATGGATGGCGTACTACACTTTGGGTATCCGTAATTCCTGTTGTTATAATTTTGGCAGT
TGGAACAAAACTTCAAGCCATATTGGCAAAGATGGCTATTGAAATCACCGAAAGACATGCAGTTGTCCAAGGAATGCCTC
TTGTCCAAGGCTCAGACAAATACTTTTGGTTTGGTCAGCCACAGTTAGTCCTTCATCTTATCCACTTTGCTTTGTTCCAG
AATGCGTTCCAAGTAACATATATCTTGTGGATATGGTATTCTTTTGGGGTTAGAAACTGTTTCCGTAATGATTACAAGCT
TGCAACAATTAAAGTAGCTTTAGGGATTGTGATGCTATGCCTCTGCAGCTATATCACTCTTCCATTATATGCTCTTTTAA
CTCAGATGGGTTCAAGGATGAAGACAGCAATATTTGACGAGCAAACAAACAAGGCTCTGAAGAAATGGCACATGGCTGTG
AAAAAGAAGCAAAGGGTGGTGAAGCTAGGAAAGTCCAATGCAGAATCAGTATATGGAAGCCCCACTGCTTCAACTTCAAC
AGTGCACCCTTCTGGGCCCACACTTCACCGTTACAAAACCACTGGTCACTCCACCCGCTCTTCAACGTACGAGGATCAAG
ATCATGAATATGAGTCCGATGTTGAGTTGTCGCCTGAGTTGTCCCAAACAACCAGCTTCATTGTGAGAATGAATCATGCT

GATCACCAACAACTACAAGAACATGAACACCGCACTGAGGGAGAAAACAACTCTAGTGAAGAATCTGATTTCTCATTTGT
TAAAGTCAAACCTAGTGACCCTGTGGAAATAACCACCACACCACATGGCATATAA

>PvMLO4 Phvul.003G271300
ATGTCTGTCTCAAAGTGCAAAAGACTGTCTCTTTGTCTGGTTTCATGGCTGTGGTTCTGTTGTGTGTCTGTGGCTTCGAG
TGAGAGTAGTATCGGTTCCAAAGACCTTGATCAGACACCAACATGGGCCGTTGCTTGTGTCTGCACTGTTTTCATCTTGA
TATCCTTAACTCTGGAGAAAAGCCTTCACAAAGTGGGAACGTGGCTACAGGAAAAGCACAAGAAGGCTTTGCTTGAGGCT
TTGGAAAAGGTCAAAGCTGAGTTGATGATTCTTGGTTTCCTTTCACTGCTTCTGACTTTCGGGCAGAGTTACATTGTTAG
AATATGTATTCCTACTGATGTGGCAGACAAATTGTTGCCATGTCCGCTTGTTGGTACCGACAGAGAATCAAGTAGTGAAG
AGGAACACCGTAGGAAACTTCTTTCTTATGAACGCAGATATTTGTCAGATGATGCTACCCCTTACCAATGCAAGAAGGGA
GATCATCAACCACTTATATCTGTCAATGGATTGCACCAGTTACACATCCTCATATTCTTCTTAGCAGTCCTTCATGTGTT
TTACAGTGCTATAACAATGCTTCTTGGGAGACTAAAGATTCGAGGATGGAAGGCATGGGAGGAGGAGACTTCATCTCATG
GATATGAGTTTGCCAATGATCCCTCAAGATTCCGCCTTACACATGAAACATCATTTGTGAGAGCTCATGCTTCTTTCTGG
ACAAGATATTCAATCTTCTTCTACATAGGATGCTTTTTTAGGCAATTTTATAGGTCTGTGGGGAAGGCTGACTACTTGGC
TTTGCGTAATGGATTTATCACTGTACACCTTGCTCCTGGAAGTAAATTTAACTTCCAAAAGTATATCAAAAGATCCTTGG
AGGATGACTTCAAGGTTGTTGTTGGAGTCAGTCCTGTCCTCTGGGCTTCATTTGTTGTTTTCCTGCTCCTAAATGTTAAT
GGATGGCATGCTATGTTCTGGGCATCCTTAATTCCTGTTGTGATAATTTTGGCTGTTGGAACAAAACTTCAAGTCACACT
GGCAAAGATGGCTATTGAAATAACAGAAAGACATGCAGTTGTCCAAGGGATTCCTCTTGTTCAAGGCTCAGACAGATATT
TTTGGTTTGGTCGGCCTCAGTTAGTTCTTCATCTTATCCATTTTGCTTTGTTTCAGAACGCATTCCAAATAACATATTTC
TTGTGGATATGGTATTCTTTTGGGCTGAGAAATTGTTTCCATGCTGACTACAAGCTTGCAATTGTGAAAGTAGCATTAGG
GCTTGCGGCTCTATGCCTCTGCAGCTATATCACCCTTCCATTATATGCTCTTGTTACTCAGATGGGCTCAAGAATGAAAA
AGTCAATATTTGATGAACAAACATCAAAGGCAATAAAGAAATGGCGAATGGCAGTGAAAAAATAAGCAGGGAGTGAAACTT
GGAAACTCCAAGGTGCGAGCCATGGATGGAAGCACAACTGATTCAACAGTACACTCTTCTGGCCCCACACTTCACCGTTT
CAAAACTACTGGTCACTCAACTCGCACCATGTCAGCCTATGATGATGACCAAGATTACCATTCTGACATTGAGCTGTCTC
CCATTTCACCAACAGCAAACTTGATAGTAAGAGTGGACCATGATGAGCACCAAGCAGAAAAAGCTCAACACCACCCAACA
CCCAACAATCAAGACCTATCACGTTTGTCAACCCTGGAAACAAGCATGAAATAG

>PvMLO5 Phvul.004G071700
ATGGCAAACGAAGTTTATGAGCGAACTCTGGAGGAAACACCAACATGGGCTGTTGCAGTTGTGTGTTTTTTGCTTCTTGC
TGTTTCAATCGCCATAGAACATCTTATTCATGGTATTGGAAAGTGGTTCAAGAAGAAACAAAAAAATGCTCTTTTCGAAG
CGTTGGAGAAGGTCAAAGGAGAGCTTATGGTGCTAGGATTCATATCCTTGCTCCTAAGTGTGTTGCAAGATCAAATTTCC
AAGATCTGCGTATCAGAAGAAGTTGCAGCTTCGTGGCATCCCTGTGCCAACCCAAAGTCCTCAACTGCAACAAAAACTGA
TGAGGATGAATCTGATGACATTCAGATAAATTCTAGGAAACTCCTACAACTTTATGACTCCATACCAAGGCGTATTCTGG
CTACAAAAGGTTACGACAAGTGTGCTGAAAAGGGAAAAGTAGCTTTTGTTTCAGCCTATGGGATTCATCAGCTGCATATA
TTCATCTTTGTGTTAGCAATATTTCACATCCTACAGTGCATTATAACACTGGGTTTGGGAAGAACCAAGATGAGAAAGTG
GAGGGCATGGGAAAACGAAACCAAGACCATTGAATATCAATTTTATAACGATCCGGAGAGGTTCAGGTTTGCAAGGGACA
CAACATTTGGAAGAAGACACTTGAATCCATGGAGTCAGTCAACAATTTCGTTATGGATAGTTAGTTTCTTCCGACAATTC
TTTGGATCAGCCACCAAAGTCGACTATTTGACATTACGACATGGATTCATCACGGCGCATCTGGCACCGGGAAGTGATGC
AAGATTTGATTTCCAAAAGTACATCCAAAGATCACTGGAGAAGGATTTTAAAGTTGTTGTGGGAATAAGCCCAGTGATAT
GGTTCTTTGCTGTGCTATTCCTTCTCACAAATACTCATGGGTGGTATTCTTCTTATTGGCTTCCATTCATCCCATTTGTT
GTAATCTTATTAGTGGGTGCTAAACTTCAAATGATCATAACAAAGATGGGACTGAGGATTACAGACAGAGGGGAAGTTGT
CAAAGGTGCACCTCTGGTTGAGCCAGGAGATGACCTGTTCTGGTTCAATCGCCCACGTCTCCTCCTCTTTCTCATTCATC
TTGTGCTTTTTCAGAATGCTTTTCAACTGGCATTTTTCTCTTGGAGTACTTATGAATTCTCCCTAGACTCTTGCTTCCAT
GAAACAACTGCAGATGTTGCCATAAGACTTACAATGGGGGTTGTCATACAAGTTCTATGCAGCTATGTGACATTACCTCT
ATATGCTCTAGTCACACAGATGGGTTCAACCATGAAGCCTACCATCTTCAATCAGAGAGTGGCATCAGCACTGAAGAACT
GGCACAGCACTGCCAAAAAGCAAGTCAAAAACAGCAAGCAAAACACACCATTCTCAAGCAAGCCATCAACCCCAACATAT
GGTATGTCTCCAATGCATCTGTTGCAAAAACACCTTGCTGGTAGAAGTGACAGTGCACAAACATCTCCAAGGACATCAAA
CTATGAAAATGAACAATGGGATGTTGAAGGCTCACCTTCCACAAGCAACCATGCAGCAGCTGAAGAAACTCAAATGCAAG
TCATGGAACCAGGCTCAAGCTCAGCACCAGAATTTCCTACCAGCTCTCAGATTGAAATCAGAGTTAGTTCATCAGAGTTT
TCCTTTGAGAAGAGACACACTGGTGCTAGTGAGGAGATATGA

>PvMLO6 Phvul.004G071800
ATGGGTGAAGCCAAAATCCATAAAAGATCACTGGAGGAAACACCAACCTGGGCGTTTGCAGTTGTTTGCTTTATGCTGCT
TGCTATTTCAATCATCATTGAGCTTGTTATTCATGCTATTGGAAAGTGGTTAAGAAAGAAACACAAAAGTGGTCTTTATG
AGTCGTTGGAAAAGGTCAAAGGAGAGCTTATGATGCTAGGGTTCCTATCTATGCTCCTAACTGTGTTCCAAGATCCACTT
TCTAAGATCTGCATATCACAAAATGCTGCATCAACATGGCATCCTTGCTCCAACCCAAAGGCCGTGAGTAATTCTAACGC
AACATCTCATACCATTGATAGGAAACTTCTCCATTTGGACCCCATTCCACGACGTGTTCTAGCTGCAAAAGGTTATGACA
AATGTGCAGACAAGGGAAAAGTTGCTTTTGTTTCAGCATATGGGATTCACCAGCTCCATATATTCATCTTTGTGTTAGCA
ATTTTTCATATCATACAATGCATTGTAACACTAGCTTTGGGAAGAACTAAGATGAGACGCTGGAAGAAGTGGGAAGACGA
AACAAAGACAATTGAATACCAATTCTATAATGATTCTAAGAGGTTCAGGCTTGCAAAGGACACAACATTTGGACAAAGGC
ACTTGAATACTTGGAGTCAGTCATCGATTTCCTTATGGATAGTTAGCTTCTTCAGACAATTCTTTGGATCGGTTAAGAAA
GTCGACTATTTTGTTTTACGACATGGATTTATCATGGCACATGTGTCACCGAGAAGTGATTCAGGATTTGATTTCCAGAG
GTATATCAAAAGATCACTTGACGAGGATTTTAAAGTTGTGGTAGGCATAAGCCCAATTATTTGGTTCTTTGCGGTTCTCT
TCCTGCTGACTAATACTCATGGGTGGTATTCTAACTATTGGCTTCCATTCATCCCATTAATTACAATTTTGATAGTGGGT

GCTGATCTACAAATGATCATAACAAAGATGGGACTAAGGATTCAAGACAGAGGAGTAGTTATGGGTGCACCTGTGGTTGA
GCCTGGAGATGACTTGTTTTGGTTCAACCGTCCACGACTCCTTCTCTTCATCATTCATCTTGTTCTTTTTCAGAATGCCT
TTCAATTAGCACATTTTGCATGGAGTACATATGAATTCTCCATAGAATCTTGCTTCCACAAAACAAATGCAGAGAATGTG
GTTAGACTTACAATGGGGGTTGTAACACAAGTTCTATGCAGCTATGTGACTTTGCCTCTTTATGCTCTAGTTACACAGAT
GGGTTCAACCTTGAAACCAACCATTTTCAATGATAGAGTAGCAGCAGCATTGAAGAAATGGCACCATACTTCCAAAAAAC
ATCTCAAACACAGCCAACACTCTGAGGCTAAAAAATCAATACCATTCTCCAGCACATCATCAAGCCCAATATTTGGCATG
TCTCCCATTCACCTGCTGCATAGACACCCTGTTGGAAGAAGTGACAGTGCACAAACTTCTCCAAGGACCTCCCTCTATGA
AAATCAACAATGTGATGTGCAAGGTGAATCTTCCGTAAGCAACCATCCAGGAACAAATGAGACCCAAATGCAAGTTTTGG
GTTCTCCTCAAACAACATAA

>PvMLO7 Phvul.005G082500
ATGGAAAAAAATGTACAAGAAACAAGGTCATTGGCCTTGACTCCTACATGGTCTGTTGCTACTGTGTTGACTGTCTTTGT
TGCTGTCTCTCTGCTTGTGGAGCGCTCCATTCACCGTTTAAGCAATTGGCTGCGGAAAACTAATCGAAAACCGTTGCTTG
CAGCTTTAGAGAAGATGAAGGAAGAATTGATGTTACTCGGGTTTATATCACTCTTATTAACAGCTACCTCACGCATGATA
GCTAATATTTGCATTCCATCAAAGTTCTACAACAGTGCTTTTGCTCCATGCACTCATTCCCAGATTGATGAACAAATGGA
AGAGAATGGCTCTGAAGAGCGTAAATTACTGACGGCTTCTGCTTATCATGTATTTGGAAGGATGTTAGATGGAATAAATA
GAAGCACTTGCAAAGAGGGTTATGAACCATTTGTTTCCTATGAAGGTCTAGAGCAGTTGCACCGGTTCATTTTTGTCATG
GCAGTAACTCATATATCTTACAGTTGTCTTACTATGTTGTTGGCCATTGTGAAGATTCACAGTTGGAGAGTGTGGGAGGA
TGAGGCTCACATGGATCGGCGTAATTCATTATCTGAGATTTCAAGAGAGCTGACTATGAGAAGGCAATCAACCTTTGTCA
AGTCTCATGCATCAAATCCATTGTTCAAAAACAGTACCCTTATTTGGGTGACATGCTTTTTCCGACAATTTGCACATTCT
GTGGTTCGTGCTGATTATCTTACACTTCGCAAAGGCTTCATCATGAATCACCACCTTTCACTAAAGTATGATTTCCATAG
CTACATGGTTCGGTCTATGGAAGAGGAATTCCAAAGGATAGTTGGTGTGAGTGGCCCGCTCTGGGGATTTGTTGTTGGCT
TCATGCTTTTCAACATTAAAGGATCTAACCTATATTTCTGGATAGCTATTATTCCTGTATCTCTTGTGCTATTGGTGGGA
ACAAAGCTACAGCATGTTATTGCAACCTTGGCGTTGGAGAATGCTGGAATTGCAAGGTTCTTCCCAGAAGCAAAGTTGAG
GCCTCGTGATGAACTTTTTTGGTTTAACAAGCCTGAACTATTGTTGTCCTTGATCCATTTCATTCTCTTCCAGAATGCAT
TTGAGCTGGCTTCATTCTTTTGGTTCTGGTGGCAGTTCGGATATGATTCTTGCTTTATTAGGAATCACCTTCTCCTGTAT
TTAAGGCTAATATTGGGGTTTGCAGGACAGTTTCTTTGTAGCTACAGCACCTTGCCACTATACGCACTTGTTACACAGAT
GGGAACAAACTATAAGGCAGCATTAATCCCACAGAGGATAAGAGAAACAATTCATGGATGGGGTAAAGCAGCTAGGAAGA
AGAGAAGGCATGGTATGTTCCCTGATGACTCCACCATTCATACAGACACAAGCACTGTGTTGTCCATTGAAGAAGATGAT
CAAGTAATTGATGCCCCTGAAGTAGAATTACCACCAGTCACGGCTGTTACACCTACCCCTTCTCCCATTGCTAATGAAAC
TTCAAGCAGGGCTGTTACACCCCTCCTAAGACCCTCTGCCTCGATATCTTCTGTGCATAGCAGTTCTAAAGCTGAAGTAA
TTATAAGATGCTCCTCTATGCCAAATGGGAGTTAA

>PvMLO8 Phvul.005G136700
ATGGGAGGAAGAAGCTTGGAGGAAACTCCTACTTGGGCTGTAGCCGTTGTTTGCTTCGTTTTGCTCACCATATCTATCGT
CATCGACCACATCTTCCATCTCATAGGAAAGTGGTTGAAGAAGAAGCACAAAAGAGCACTATATGAGTCACTTGAAAAGA
TCAAATCAGAACTTATGTTATTGGGGTTCATATCGCTGCTTCTAACGGTAGGACAAAGTGTAATATCGAGGATATGTATA
TCAGAAAAGGTTGCAGGGACATTCCACCCCTGCACTCACAAAACAGACCACACCCGCAGATTACTACCGGAGCTTTTCCG
TTCCGATCACAACCAAAATCTACGCCGTATTTTGGCGGCGGGAGGAACTGACAAGTGTGCGGCACAGGGTAAAGTCCCGT
TTGTCTCATCAGAGGGCATTCATCAACTCCATATATTTATCTTCGTGCTGGCTGTTTTTCACGTTCTTTACTGCATTCTC
ACTCTGGCTCTTGGTAGAGCAAAGATGAGAAGGTGGAAACGGTGGGAAGAGGAAACAAAAACAGCACAGTATCAATTTTC
ACACGATCCTGAACGGTTTAGATTTGCGAGGGAGACTTCGTTTGGAAGAAGACACTTGAGTTTCTGGACTCAACACACTA
TCCTAGTTTGGATTATTTGTTTCTTCAGGCAGTTTGTACGCTCAGTTCCTAAAGTTGATTACTTGACCTTGAGGCATGGA
TTTATGATGGCGCATTTGGGACCTCAAAGTCGCCATAAATTCGACTTTAGGCAATATATCAAAAGGTCTTTGGAAGAGGA
CTTCAAAGTGGTCGTTGAAATCAGTCCTCCAATCTGGTTCATCACAGTGCTTTTTCTCCTGTTTCACACCCATGGCTGGT
ACTCTTATCTGTGGCTGCCATTTGCTCCTTTGATAATTGTTCTATTAGTGGGAACGAAGCTGCAAGTGATAATAACGAAG
ATGGGTCAGAGAATTCAAGAAAGAGGAGAGGTTGTAAAGGGCATGCCACTGGTGCAGCCTGGGGATGATCTCTTCTGGTT
TAACAAACCTCGACTTATTCTCTACCTCGTCAATTTCGTGCTCTTTCAGAACGCTTTCCAACTTGCCTACTTTTCATGGA
CTGCGGTTCAGTTTGGGATGAAATCCTGTTTTCATTCGCAGACGGAGGATGTGGTGATTAAAATCTCAATGGGGGTTTTC
GTTCAATTCCTTTGCAGCTACGTAACTCTTCCTCTCTATGCTCTCGTCACACAGATGGGTTCAACCATGAAACCAACCAT
ATTCAACGAAAGAGTAGCCATGGCTCTGAGGAATTGGCACCATGCAGCCAAGAAACACGTCAAGCAACAGAACCGTAGAT
TACAGTCAAGGCCCACAACCCCCAATCACTCTACATCTCAGGCCCATCTCTTACGCCGCTGCCACAGTGAAATTGACATC
TATCCCACTGACTCTGAGGCCCATCATCCCTATGAGATGGATTTGAGCCCATCATCCAAGGTGCATGATCACGAAGTGGA
AATGGAAATGGGCCACTTGGATCATGGCCCACTAGAAGATGTGAATGAGTTGAACTCAGCCCTTGCGGGATCAGGTGTGA
CTCAACACGAGATTAATATTGAACACGGCAAGGAGTTTTCATTTGATAATAGATGA

>PvMLO9 Phvul.005G136800
ATGGGAGGAAATGGAGGAAGGAATTTAGACGAAACACCAACCTGGGCTGTGGCCGTTGTTTGCTTTGTTTTGCTCTCTGT
ATCTATCATCATCGAATACATCTTCAATCTAATAGGAAAGTGGTTGAAGCAGAAACACAAAAGAGCTCTGTACGAGTCAC
TTGAAAAGATCAAATCAGAGCTCATGTTATTAGGGTTCATATCGTTGCTCCTAACGGTAGGGCAAGGTCTAATATCGAGG
ATATGCATATCAGAAAAGGTTGCAGGGACATTTCACCCTTGCGCTCATAGAAGAGTGAAGCAAAACAACACTCCCCCATT
AGAACACGATGAATACCGAAATAACCGCCATCTTTTAGCGTTGGAAGACAAGTGTGCACCGCAGGGTAAAGTGCCATTTG
TGTCATCGGAAGCCATTCACCAGCTCCATATATTTATCTTCGTGCTGGCTGTTTTTCACGTGCTTTACTGCATTCTCACT
CACGCCCTTGGTAGAGCAAAGATGAGAAGGTGGAAGCGATGGGAAGTAGAAACCAAAACACCAGAGTACCAAGTTTCACA

CGATCCTCAACGATTCAGATTTGCAAGAGAGACATCGTTTGGAAGAAGACACCTAAGTTTCTGGACCCAGAATGCTGTCC
TGGTTTGGATTATTTGTTTCTTCAGACAGTTTGTACACTCAGTTCCTAAAGTTGATTACTTGACCTTGAGACATGGATTT
ATGATGGCACATTTGGGGCCTCACAGTCACCAGAAATTCGACTTTGGGAAGTACATCAAAAGATCTTTGGAAGAGGACTT
CAAAGTGGTCGTTGAAATCAGTCCTCCAATCTGGTTCATCACAGTGCTTTTTCTCCTATTTAATACTCACGGCTGGTACT
CTTATCTGTGGCTGCCATTTGCTCCTTTGATTATTGTTCTATTAGTGGGAACGAAGCTGCAAGTGATTATAACGAAGATG
GGTCAGAGAATTCAAGAACGAGGAGAGGTTGTAAAGGGCATGCCAGTGGTGCAACCTGGGGATCACCTCTTCTGGTTTAA
CAAACCTCGACTTATTCTCTACCTCATTAATTTTGTGCTCTTTCAGAATGCTTTTCAGCTTGCTTTCTTTTCATGGGCTG
CGCTTCAATTTATGATGAAGTCATGTTTCCAGTCCCAAAAGCAGGATGTGGTGATCAGAATCTCAATGGGGATCTTCGTT
CAATTCCTTTGCAGCTACGTCACTCTTCCTCTCTATGCTCTCGTGACGCAGATGGGTTCAACAATGAAGGCAACGATTTT
CAACGAAAAAGTAGCGATGGCTCTAAGAAATTGGCACCACACTGCGAAGAAGAACGTGAAAGAGAAGCGTGGAGTACGAT
CGCAGAGTCCTTTGTCAACTCGGCCTTCAACCCCGCAGCAGCAGCCAAAGTCGCAGGCTAATCTGCTGAGACGGTACCAC
AGTGAAATGGCGACTTATCCATCTTCTCCGATAAGGTTCGATTTTGAGGCACATCTTCCTTATGGGATCCATTCACCCCC
CTCCTCCATGGTTAATGCTGCAGCTACTTCTTCTATTCATCGCCATGAAATGGAGATGGAAACGGAAATTGAAATGGATC
AGGTCTCAGCTCTCACTCAACGCCAGATTGATATTGACATTGACATTCAAGACAATCAAGAATTTTCTTTTGCTAAAAGA
TAA

>PvMLO10 Phvul.006G133800
ATGGCAGAAGAACTGAACGACTCTCTGGAATATACACCAACATGGATTGTTGCGGTTGTTTGCTCCATAATTGTTTTCAT
CTCTCTTTGTGTTGAACGTGCCCTTCACAAACTCGGAAAGTACCTAAGGAGCAAAAATCAGAATGAATTGTATGAAGTCT
TAACAAAATTGGAAGAAGAATTGATGCTTTTAGGGTTCATTTCCCTCTTATTAACCGTCTTTCAAGGTTTAATAAGCGAC
ATTTGCATCTCACCAAACCTTGCAACCCAAATGCTTCCATGCAAAAGGCCTCATAGGTCTTCACAAGGTTCAGAGCATTA
TCAGATATACTATGATTCTATAATAAACAGAAGAAGGCTTTTTTCTACTGGTTCTGGTTCAGATCATTGTACACATAAGG
GGAAGGTTCCACTGTTATCATTGGAATCTTTACATCAGCTTCACATTTTTATTTTTGTATTGGCCGTTGTACATGCAATA
TTCTGTGTCACAACAATGCTTCTTGGAGGTGCAAAGATACGGGAATGGAAGGCCTGGGAGGATTATTATGGAGACCACAG
AGAAGTAATAGATACCCATGAGTTCTTCAAAACACGTGATCGAGGACATTGGAGAAAAGCAGCTGTGATTAGCTGGCTGA
TATCATTCTTCAAGCAATTTCATGGCTCTGTTACAAAGTATGACTACTATGCACTTCGGCATGGATTTGTCAAGAAACAC
TACCCAGATAATCCCACATATAATTTTCATAATTACATGCTGGGGACACTTGAAGAAGACTTCAAAAGAGTTGTAGGTAT
AAGCTGGTACCTGTGGGTCTTTGTTGTGCTGTTTTTGCTGTTGAACATTGGAGGATGGCACACTTATTTCTGGTTAGCCT
TTTTACCATTGATACTTCTGCTTCTGGTGGGGGCAAAGTTGGAGCACATAATCGCTCGTTTGTATCAGGATTCAATAGGC
GTATTGGGAAGAGAAGATGACAATTCAATGAAGCCATCAGATGAATATTTTTGGTTTACCAGCCCACCCCTAGTCCTCCA
CTTACTTCACTTCATTTTGTTTCAAAACTCTTTTGAGATTGCATTTTTCTTCTGGATTTGGTGCACATATGGATTTGATT
CATGCATTATGGAGAAAGTAACCTACGTTATCCCAAGACTTATAATGGGTGTAATTGTTCAAGTGCTTTGCAGTTATAGC
ACCTTGCCTTTGTACACTATTGTGACTCAGATGGGTAGCAAGATCAAGAGTCAGAAGTCGCCTCCAAGTGACCCCCTCAA
AACACCGCATTCCAACTACATGATAAAAGAATCAACCCAAATTGATGAACAAGCAATAATCATGGTGGAAGATGCAACAT
CTTCAATTGAGCTTCCACACATTGTCCATCTTCCCTTCGAGAAACCTAATACATCTAACAATTAA

>PvMLO11 Phvul.006G216900
ATGGCAGAGGTGCTGCAGCGAGGTCGCTCTCTGGCTGAGACACCAACATATTCAGTTGCCTCTGTTGTTGCTGTCATGGT
CTTTGTCTGCTTATTGGTTGAACGTTCAATCTACCGCTTTGGAGAGTGGTTGAAGAAAACAAGAAGGAAGGCACTTTTTG
CTTCACTGGAGAAACTCAAGGAAGAGCTGATGTTGCTTGGACTTATCTCTTTGCTGTTGGCACAAAGTGCAAGATGGATC
TCTGAAATTTGTGTGACTTCGTCACTTTTTAGCAGTCGATTTTATATTTGTTCTGAGCAAGACGTGGACATAAATGAGAA
TATCATGCTGGAAAGCTCATCATCATCTATCCCTGATGAAACTAGTATTACTGGAGCATTCAACCAATGTGGAGAGGGTC
ATGAACCTTTTGTTTCATATGAAGGTCTTGAGCAGCTGCACCGGTTCTTATTTGTTCTCGGAATCACTCATGTTTTGTAT
AGTTGTCTGGCAGTTGGTTTGGCAATGAGTAAGATCTATAGTTGGCGCAGATGGGAAAATCAGGCTAGGATGCCAAGTGA
TGGAAACCAGCAAGCAAAGAAAATCAAGTTGATGAAGCGACAGACAACATTTATTTTCCATCGCACTTCTCATCCATGGA
GCAGGAGTCCAATCCTAAACTGGATGCTCTGTTTCCTACGTCAGTTTAGGAGCTCTATACGAAAATCAGATTACCTTGCA
CTACGTTTAGGTTTCATCACTGAACACAATTTGCCGTTATCTTATAATTTCCATCAATATATGGTTCGAAGCATGGAAGA
TGAATTTCATGGCATTCTAGGAATAAGCTGGCTGCTTTGGATTTATGCCATAGTGTGCATCTTCGTGAACATCCACGGTC
TGAATATATACTTCTGGCTATCATTTATCCCTGCAATTCTTGTGATGTTGATAGGGGCAAAGCTTCAACATGTTGTATCC
ACCTTAGCACTTGAAATTATGGAGCAGACAGGTCCGTTTCCTAGGACACAAGTAAAGCCACGCGATGATCTATTCTGGTT
TAAAAAACCAGATATACTGTTATGGCTGATACAGTTTGTCATATTTCAGAATGCATTTGAAATGGCAACGTTTATTTGGA
CATTGTGGGGATTCCAAGAACAATCGTGCTTCATGAGAAATCATTACATGATCATCATTCGGTTGGCATCTGGTGCTATT
GTTCAGTTCTGGTGTAGCTACATGACAGTGCCCCTGAGCATAATAGTTTCTCAGATGGGATCGAAATGCAAGAAGGCCTT
GGTGACAGAGAGTGTTAGAGAGTCTCTGCATAGTTGGTGTAAAAGAGTGAAACACAAGTCTAAGCATGATTCGTTGCATT
CACACACTGCAAGATCAGTATGCTCCCTGGAATCAACAATTGATGAGAGGGACGAAATGACAGTAGCATCTGGGACTCTA
ACTCGATCCTCTTCTTTAGGGTCTTTGAATCAGATAACAGTGAGTTCAGTAGAACAGCTGAAGTTGGAGACCCCCAACAA
GGCAAAACATTCAAGGTAG

>PvMLO12 Phvul.007G093600
ATGGGCAAAGAAGAAGTTCTACAGAGGAAGTTGGAGGCAACTCCTACATGGGTAGTTGCAGCTGTGTGCTTTGTGATGCT
TGCTATTTCAATCATCATTGAACATGTTATTGAAGAACTTGGAAAGTGGTTAAAAAAGAAACACAAAAAGGCTCTTCATG
AAGCGCTGGAAAAGATCAAAGGAGAGCTTATGCTGCTGGGATTCCTATCCTTTCTCTTAGTGGTGTTTGAAGATCGAATT
GTTTCTATCTGCATCCCAGAAAGTGTTGCATCCACTTGGAATCCTTGTGATCCCAGTTATGTGAGTAAAGGTGCAGAAGG
ATATGTTGAGAAATGCGCAAAAAAAAAGGAAGGCACAGTTGCTTTTATGTCTAAATATGGGCTTCACCAGCTCCATATAT

TCGTCTTTGTGCTTGCTATTTTTCACATTCTACAGTGCATCATGACACTGACTTTGGGTAGAACTAAGATATCAATATGG
AGGAAGTGGGAAGACGAAACAAAGGGTCTTGAACATCAGTTCTCCCATGATCCTGAGAGGTTCAGGTTTGCTAGGGATAC
AACGTTTGGACGAAGGCACTTGAACTCATGGAGTAAATCACCAACTTTATTATGGATAGTTAGCTTCTGCAGACAATTCT
ATGGATCACTTAATAAAGCTGACTATATGGCATTACGGCATGGATTCGTCGTGGCACATTTGACTCCAGCAAGTGAGCCA
AAATTTGACTTCCAGAACTATATCACCAGAACACTAGATGAGGATTTTGCAGTAGTGGTGGGCATAAGTCCAACCATATG
GTTCTTTGCAGTGGTAATTCTGCTCACAAATACTCACGGGTGGTATTCTTATTTTTGGGTTCCATTTATCCCACTAATTA
TAATCTTGTTGGTGGGAACAAAGCTACAAATGATCATAACAGATATGGCACTAAAGATTCAAGATAGGGGGGAAGTGGTC
AAGGGTGAACCAGTGGTTCAGCCAGGAGATGAATTGTTCTGGTTCAATAATCCTCGCCTCGTTCTCTTTCTGATTCATCT
TGTTCTGTTCCTGAATGCATTTCAATTGGCGTTTTTTACTTGGAGCACATTAGACAATGGGTTCAAAATAAACTCCTGCT
TTCACCGAACTACTGCAGATATTGTCATTAGACTTACAATGGGGGTTCTCACCCAAGTTCTATGCAGCTATGTGACTTTG
CCTCTTTATGCTCTAGTCATACAGATGGGTTCTAGCATGAAACCCACCATTTTCAAAGATAACGTGGCATCAGCACTAAA
GAGCTGGCATCACTCTGCTAAAAAGCATCTGAAACACAACAGGGATTCAACTTCTAACACACCATTCTCAAGCAGACCAG
GAACCCCAACTCGTGGCATGTCTCCAGTGCACCTGCTTCACAAGCACCCTAGACACAGTGATAGTCCACTAGTTTCTCCA
AGAGCATCCGATTATGAAAATGGGCAATGGGATGCTGAAGTGTCACATTCCCCAAGCCACCATGCAAGAGATATTGATCA
TGAAGAGACCATGCAGGTGCAGCCTCCAGCAGCAGCAGAATTGCCTCCTGCTGGACTCAATCCTGTTCGAACTCAACATG
AAATCAACATTGCTTTATCTGAATTTTCATTTGGGAGGGGACGCAACCCTGGTAGAGCAGATGAGTATTAG

>PvMLO13 Phvul.009G006800
ATGGCTGGTGGAGCATCCGGTGGAGAAAGAACGTTGCAGGAAACACCCACGTGGGCAGTGGCTTCTGTTTGCGCAGTCTT
CGTCGTTCTCTCTGTGTTAATTGAACATGGAATCCATTTACTTGAAAAGTGGTTTCAAAAACGAAATAAGAAAGCCATGA
GAGAAGCCTTGGAGAAAATCAAAGCAGAATTAATGCTATTAGGATTTATATCCCTGTTAATTACTTTTGGTACAAAATAC
ATTGCAAAGATATGCATCCCTACATCAGCTGGTGATATCATGCTCCCATGCAAGAAGGTGGAAGAGTCAAAGAACTCCGA
TGATTCCAATGGCAGGAAGTTACTTTACTTTGAGGACAATATGGAATGGCGTCGAGTTTTAGCAACTGCGTCTTCTGGTG
GTGACTTTTGCTCACAGAAAGGTAAAGTGTCATTGATTTCTCAGTCAGGGGTGCACCAATTGCATATATTTATTTTCGTG
CTTGCTGTTTTTCACATACTGTACAGCGTAATGACCATGGTACTAGCCCGAGCAAAAATGAAGAAATGGAAAGCCTGGGA
AGCTGAAACATCATCTTTGGAGTATCAATTTACAAATGATCCTTCAAGGTTCAGGTTTGCGCATCAAACATCATTTGTTA
GGCGTCACTCTGGTTTGTCTAGGATGCCAGGAATTCGATGGATTGTAGCTTTCTTCAGGCAATTCTTTGCGTCTGTCACG
AAGGTGGATTACATGACCATGCGGCATGGATTCATTAACGCACATTTTGCTCCCAATAGCAAATTTGACTTCCAAAAATA
CATTAAAAGATCCATGGAGGACGACTTTAAAGTGGTCGTGGGTATAAGTATTCCGTTATGGGTTTTTGCTATCGTCTTTA
TGCTTGTGAACGTTTACAAATGGTACACTCTCACCTGGCTATCATTAGCGCCGCTAGTGATACTTCTATTAGTAGGCACC
AAACTTGAACTTATAATCATGGAAATGGCCCAGGAAATCCAAGATCGAACCACTGTTGTCAGAGGAGTCCCTATTGTAGA
GCCCAACAACAAGTATTTTTGGTTTAATCGGCCTCAGTGGATTATCTTCTTAATCCACTTTACCTTGTTTGAGAATGCAT
TCCAAATAGCCTTTTTCTTGTGGACATGGTATGAATTTAAGATCACATCTTGCTTCCATGAAAACTTGCCGCTAATACTG
ACAAGGGTTTTCCTTGGGATAGCATTGCAAGTGGTGTGCAGTTACATAACATTTCCTCTCTACTCCCTTGTAACACAGAT
GGGATCTCACATGAAGCAAGCAATTTTTGAAGAGCAAACTGCTAAAGCACTAAAGAAATGGCAAAAGGTTGCAAAAGACA
AGAGAAAGTTGAGAAAAGCAGGGGTAGATGTTCCTTCAGGAACAATGAGTGGGGAAGCAACACCAAGCCAAGGCACATCA
CCCATACATTTACTTCACAAGTACAAGCCTAGCCAAACAGACACAGATGTTGTTCTCTATTCTCCACGATCATACCAATC
TGATACTGACTTCTCTGACACAGAGGGGTCATCACACCAACTCAATCAAATAACACAAACTCATCAAGCTCCAAGAAATG
GAGAGGCTCACAGTATCGAATTTTCCTTTGTGAGCCATAACAATGTCCAATTTTGA

>PvMLO14 Phvul.009G007000
ATGTCGGGAGGAGGAGGCGGTGACTCCAGACAGCTAGATCTCACGCCTACATGGGCTGTCGCCGCGGTTTGTGCAATTAT
CGTCCTCATTTCTATACTCTTGGAAAAAATCATACACAAATTCGCAAGGATGTTTGAAGAAAAAAAGAAACATGCTTTGC
TAGAAGCTCTTGAGAAGATTAAAGCCGAGCTTATGGTTCTGGGATTTATTTCCTTACTTCTAACGTTTGGTCAAAACTAC
ATTTCTAAAGTGTGTATTCCCCAAAAGTATGCACACACAATGCTACCGTGTCTTCCCATTGACCAACGTGACGGAGGGGG
TCCCGCAACCGAACACGGAGCGGGTGCCGCAACCGAACACGGAGCGGGTGCCGCAACCGAACACGGAGCGGGTGCCGCAA
CCGAACATGAAGTTGAACCTGAGGGAGAGGGGGGCGAGAGTGCTGAAGGAGGTGGCCATCGTCGCAGAATTTTATCATAT
GATCGTAGATTTCTATCTGGTGGTGGTGGAGGCCCCAGTTGTAAACCGGGGAAGCTGCCTCTTATATCAGTGAACGGGTT
GCATCAGTTACACATTTTTATATTTTTCTTGGCTGTCTTCCATGTGATATACAGTGCCATAACAATGACCCTTGGAAGAG
CAAAGATTCGTGGGTGGAAGGCATGGGAGGAGGACCATGTCGTGGATGAAGATGCCTTGAATGATCCTAGAAGATTCAGA
CTTACTCACGAGACATCATTTGTGAGGGATCACAGCAGTTTCTGGACTAAAACGCCAGTTTCCTTCTATTTTGTATGCTT
CTTTCGACAATTTTTCAGATCTGTTCGCAGGGCTGACTACTTGACCATGAGACATGGATTTGTGACTGTCCATTTAGCAC
CGGGAAGTAAGTTCGATTTTCAAAAATATATCAAGAGGTCATTAGAAGATGACTTTAAGGTAGTTGTAGGAATCAGTCCA
ATACTTTGGGGATCAGTAGTGTTATTCTTGCTTGTGAATGTCCATGGATGGCATGCTGCTTTTTGGGTGTCTTTTCTCCC
TCTAGTGGTGATTCTTGCTGTTGGAACAAAACTTCAAGCAATTATTACTCGAATGGCACTTGACATAAAAGAAAGGCATG
CTGTAGTGCAGGGGATCCCTCTTGTGCAAGTCTCTGACAAGTATTTTTGGTTTGCATGGCCTCAGTTAGTTCTTTATCTC
ATCCATTATGTCCTCTTTCAGAATGCATTTGAGCTGACATACTTCTGGTGGACATGGTATGAATTCGGCTGGGCATCTTG
CTTTTACGAGGATGATCGTCTCATGATCATCAGAGTAGCTCTTGGGCTGGGAGCTCAAGTCGTTTGCAGTTATGTCACAC
TTCCACTCTACGCACTCGTTACTCAGATGGGATCGACGATGAAGAAGTCAATATTTGATGACCAAACGTCGAGGGCGCTG
AAACAGTGGCATAAGAATGCCCTGAAGAAGAAAGCGTCAAAAGGACGCCACGAAACTCGAACGTTAGGTGGGAGCCCGGG
AGAGCATACACCGGAACATTCGCCGCACCCGCCGCCACCCAGAGACACAGAAATGGCTGCTCAGACCGCCACCATCGTCA
CCAGCGTTGATAACGAAACCTATGATAAGCGGGACCTTCTAACTGGACCGTGA

>PvMLO15 Phvul.009G117900

ATGAGTGGCGGAGGAGGAGAAGAGGGAGATAGTCTGGAGTTCACTCCCACCTGGGTAGTGGCCGCCGTTTGCACAGTCAT
CGTCGCCATATCCCTCGCCGCAGAGCGCTTTCTTCATTATGGCGGAAAGTTTCTCAAAGCCAAGGACCAGAAGCCGCTCT
ACGAAGCTCTCCAGAAGATCAAAGAAGAGTTGATGTTGTTGGGCTTCATTTCCCTCCTTTTGACGGTTACGCAAAACGGC
ATTACCAAAATCTGCGTTCCGCCGGGCTTCACGCGCCACATGCTTCCGTGTAGCCTCGACCTCGCCCCCAACCACGAATC
TCATTTCCAGACTTTTTATTTCCCCGGAACACCCAGACGCCTTCTTGCTGCCACTGCGGAGGAACACGCGCCGCCCCACT
CCAAGGCTGAGACTGAGACCGCGACTGGTTTCTGCTCTCGCAAGAACAAGGTACCTTTGTTGTCTGTGGAAGCACTGCAC
CACCTCCACATCTTCATTTTTGTCTTGGCTGTCGTACACGTCACCTTTTCCGCACTCACCGTTATCTTTGGAGGTGCTAG
AATTCGTCAGTGGAAACACTGGGAAGATTCAATTGCAAAACAGAACTACGCAACCGACCAGGTTCTGAAACCAAAGGTGA
CTAATGTTCAGCAGCATGATTTTATCAGGGACCGTTTTTCTGGTTTTGGCAAAGACTCTGCTATAGTCGGTTGGTTGATA
TCCTTTTCTAAGCAATTTTATGGATCTGTGACAAAATCAGATTATGTGACGTTACGACATGGTTTCATTATGACCCACTG
CAGGACAAATCCAAAGTTTAATTTCCACAAGTACATGATCCGTGCCCTTGAAGATGATTTCAAGAAAGTTGTTGGTATAA
GTTGGTATCTTTGGCTCTTTGTGGTTATCTTCTTGTTGCTCAATATCAATGGTTGGCATACATATTTCTGGATTGCTTTT
ATTCCTGTCATTCTATTGCTTTCTGTGGGCACTAAGCTGGAGCATGTAATAACCCAACTAGCTCACGACGTGGCTGAAAA
GCATGCAGCCATAGAAGGTGACTTAGTTGTGCAACCATCAGATGAACACTTTTGGTTTCATCGGCCCCGTGTTGTCCTCT
TCTTAATTCACTTTATCCTTTTCCAAAATGCTTTTGAGATAGCATTTTTTTTCTGGATATGGGTTACATATGGATTTGAC
TCGTGTATAATGGGGCAAGTTCGATACATTATTCCAAGGCTCGTTATTGGGGTATTTATTCAGGTATTATGTAGCTACAG
CACTCTGCCACTCTATGCATTAGTTACGCAGATGGGAACTCACTATAAGAGGGCAATATTTAATGATCATTTGCAACAAA
ACATTGTTGGTTGGGCACAAAAGGCGAAGAGGAGGAAAGAACAAAATGCTGATGGCAATCCTGGCCAAGGAAGTTCTCAG
GAAAATGGTAATACAGGAATCCAGCTTGGGTCGATTTTCCCGAAGAAGGCATCTGGTCCAGGAAACAGTTCTTCTGCCCC
CAAAGCTGACCGAACCAGCTGA

>PvMLO16 Phvul.010G034400
ATGGGTGGTGGTGGTGGTGAAGAAGGGAACAATTTGGAATTCACTCCCACTTGGGTGGTTGCTGTTGTTTGTTCTGTGAT
CGTTTCTGCTTCATTCGCTGCAGAACGGTTTCTTCACTATGGAGGGACTTTTCTCAAGAAGAAGAATCAGAAGCCACTCT
TTGAAGCTCTGCTGAAGATCAAAGAAGAGTTGATGCTGTTGGGATTTATTTCTCTGCTACTGACTGTAACACAAAATGGG
ATTATCAAAATTTGTGTTCCTGAGAGTTGGACTCGCCACATGCTTCCTTGCAGTCTTAAGGACAAAGAAGAGTTAGAATC
AGCGAAACTCACATCACATTTTCAGACTTTTTTCTCTTTCACTGATATTCCAGCCACTGTAAGGCACCTTTTAGCTGAGA
ATGAGAATGAAGATCACCAATCTGGTGAAAAACTTGGACACTGTGCTAAGAAGGGCAGGGTTCCTCTATTATCTGTGGAG
GCACTGCATCACTTGCATATCTTTATTTTTGTCCTAGCCATAGTCCATGTGACATTTTGTGTTCTCACTGTTGTTTTTGG
AGGGTTAAAAATACGTCAGTGGAAGCACTGGGAAAACTCTATTGTAGATGAGAATAATAGAAAACAACCAGTTTTGGAAT
CAATAGTGACTCATGTTCACGAACACGCTTTCATCCAGAATCATTTTACTGGTTTTGGCAAAGATTATGCTGTTTTGGGT
TGGCTGAAATCATTTTTCAAGCAATTTTATGGATCTGTGACAAAGTTAGATTATGTGACATTAAGGCTTGGTTTCATTAT
GACTCATTGCAGAGGAAATCCAAAGTTTAATTTTCACAAATACATGATTCGTGCCCTTGAAGACGATTTCAAGCAAGTTG
TTGGTATAAGTTGGTATCTTTGGATCTTTGTGGTCATCTTCATGTTGCTTAATGTCCATGGTTGGCACACATATTTTTGG
ATTTCTTTCATTCCTCTCATTCTTCTACTGGCTGTGGGTACTAAGTTGGAGCATGTGATAATTCAATTAGCTCATGAAGT
GGCTGAGAAACATTCAGCCATAGAAGGTGAATTAGTTGTTCAACCAAGAGATGATCACTTTTGGTTTCATCGCCCCCACA
TTGTCCTCTTCTTGATTCACTTCATCCTTTTCCAAAATGCTTTTGAGATTGCATTTTTCTTTTGGATATTGTTTACATAT
GGTTTTGACTCATGTATAATGGGACGCGTCCGTTACATTGTTCCAAGGCTCGTTATTGGGATATTTATTCAAGTACTATG
CAGCTATAGTACCCTACCACTTTATGCAATTGTAACACAGATGGGAACTCACTTTAAGAAAGCCATATTTGATGATCAAG
TGCAAGCACGTCTTGTTGGTTGGGCACAAAAGGCAAAGAAGAAAGGTTTGAGAGGTGATAATAATGGCCAATCTGTTCAA
GGAAGTCCTCATGTGGGTGCTGGAATTCAACTAGGACCAGTCTTCAGGAAGCCATCTCCCCCAGAAGACACTGTTTTACC
CTAG

>PvMLO17 Phvul.011G073800
ATGGCAGGTGGCGGAGGAAGAAACTTGGAGGAAACACCCACATGGGCCGTCTCCACTGTCTGTTTTGTTCTGATCTTTAT
ATCTATAATCATCGAACACATCATCCACCTCATAGGACAGTGGTTGAAGAAGAAGCGCAAAAGAGCGCTGTACGAGTCAC
TCGAAAAGATCAAATCAGAGCTTATGTTATTGGGGTTCTTGTCGTTGCTTTTAACGGTAGGACAAGGTCTGGTATCAAGA
ATATGCATATCAGAAAAAATTGCATCGACATGGCACCCTTGTAGCGCCGACGACACGCATAGTGAAGAATCAGGACAGGG
AACCAACAGCCGCAGGTTGTTAGCGGCGTTCCATGGTTCAAACGATGTAAATCCACGCCGTGTTTTGGCGGGAGGAGGAA
GTGACAAGTGTGGCGAGGGTAAAGTCCCGTTTGTGTCATCGGAGGGAATCCATCAACTCCATATATTTATCTTTGTGCTA
ACTGTTTTTCATGTCCTTTACTGCATTCTCACCATGGCTCTGGGAAGAGCAAAGATGAAGAGGTGGAAACGATGGGAAGA
GGAAACCAAGACACCAGAGTACCAATTTTCACACGATCCTGAACGATTTAGATTTGCGAGAGAAACATCGTTTGGAAGAA
GGCATTTGAGTTTCTGGACCAAAAACCCTGTTCTCATGTGGATTGTTTGTTTCTTTAGGCAATTTGTGCGATCAGTTCCT
AAAGTGGATTACTTGACCTTAAGACATGGATTTATCATGGCACATTTGGCTCCTCAAAGTCACTCCAAATTTGATTTTCG
GAAATACATAAAACGATCACTGGATGAGGATTTCAAAGTCGTTGTTGGAATCAGTCCTCCATTCTGGTTGTTCGCGGTGC
TGTTTCTCCTCTTGAACACTCATGGCTCGTACTCTTATCTGTGGCTGCCATTCATTCCTTTGATTATCATTCTGTTAGTT
GGAACCAAGTTGCAAGTGATCATAACTGAGATGGGTCTAAGAATTCAACAAAGAGGAGAGATTCTGAAGGGAGTGCCACT
GGTTCAACCAGGAGATTATTTGTTCTGGTTTAACAAACCTGGTCTTATTCTTTACCTTATTAACTTTGTGCTCTTTCAGA
ATGCTTTTCAGCTTGCTTTCTTCTCCTGGTCTGTGCTTCAATTTGGGATTAAATCTTGTTTCCACGCACATACAGAGGAT
GTAGTGATCAGAATCACAATGGGGGTTCTTATTCAAATCCTGTGCAGCTACGTTACTCTTCCTCTCTATGCCCTAGTTAC
ACAGATGGGTTCAACAATGAAGCCAACGATATTCAACGAAAGAGTTGCTGAGGCACTGCGGAACTGGCACCAGACCGCGA
AGAAGCAGATAAGACAGAACCGTGTGGGACCCTTGTCGTTGTCGGTGACACCGATGTCGAGCAGACCCACAACCCCGAGC
CACAACATGTCCCCAGTGCACCTCTTGCGCTACTACCGTTCTGAAATTGACAGCTTCCCCACCTCTCCACGACGTTCGAA
CGTCGACGGCGACCACACCCAGCCCTGGGACGTTGAATCCCCTTCTCCTTCCTACTCGCACCACGAAATGGAAATGGGTC

ACACCAATGACCCCACCACTAACACAACCCACCATGAGATTGTTCCTGCTGCAAACACCAGGGAATTCTCCTTTGATAAA
AGACCTACCACAGCTCCGTAG

>PvMLO18 Phvul.011G122800
ATGGACATGGAAGAACAAAGATCCCTTGAAGAAACTTCAACATGGGCTGTCTCAGTTTTCTGCTTCTTCTTTCTTCTAAC
ATCTTTGATCATTGAGGGCAGTCTCCACAAATTGGCTGAGATATTGAGGAAAAGGAAAGACAAATCACTAGGGAGGGCAT
TGACTAAAACCAAAACAGAAATGATGAAATTCGGTTTCATGTCCTTTCTTCTCACCATATCGGAAGTGCCAATATCGAAA
ATCTGCATCAGCAAGGGTGTGGCAACATATTTTCTCCCTTGCAAAGATGCTGTGGACTTAACAGGGTTATCAGCTACTCG
TACATCAACCTCAGAATTGGACGTAGCACATGCAACAAACCAATCTAACGTTGAGTTCAACTATTGTGAAGCAAAGGGTA
TGGTTAGTTTAATTTCAAGTGAAGGAATCCTGCAGCTAAATATATTCATCTCCTTTTTGGCTGTTTTTCACATCTTCTTC
TGCACATTGACAATGTGCCTTGGTAAAGCAAAGATGAGAAGATGGAAAAGATGGGAAGAGGAGACCCGAACACTTGAATA
TCAGATAGCAAATGATCCAAGAAGGTTCCAGTATACAGGTCAAACAATGATTGGAAAGCGCCATTTGAATTTCTGGAGCT
ATCATTCTCCTTTGCTTTGGATGGTCTGCTTTATTCGGCAGTTTTATGGCTCAGTCTCAAAAGATGATTACTATACTCTA
CGAAATGGGTTTATTGCAGCTAATATTTCTTTAGGCAGCAATTTCAACTTCAAGAAATTTCTTTCCCGCACTTATGATGA
AGATTTTGAGAAGGTTATGGGAATCAGGATTTGGATCTGGATATTCTCCATACTTTTCATCTTTTTTAGTGCACATGAGT
TCTATAATTACTACTGGCTTCCATTTATTCCACTTATGGTTGCTTTACTTGCTGGGACAAAACTCCAAGTCATAATAACA
AAGATGTGTGTAGATAGTTGCAAGGCAAACCCAGTGATCAAAGGGAGTCTACTTGTAACACCTAGTGATGCCCATTTCTG
GTTTCATCGACCTGAATGGCTTCTGTATTTACTCAAGTTCATCCTTATCCAGAACTCCTTTCAACTTGCATTCTTCACCT
GGACTTGGTATGAATTTGGTCCAAGGTCTTGCTTCAATCGAGAAAATGAGGACATTGGCATAAGGATTGCCATGGGTATA
GCTGTGCAGCTCCTTTGTGGTTACGTCACACTTCCCCTTTATGCACTTGTTACTCAGATGGGTTCTAGCATGAGGAGAGA
AATTTTCACTGAGAGAATTTCTAGGGGCCTCAAAAATTGGCACAAAAAGGCTAAACAAACTCTGCACAAAAACAACTCAA
CTTCAAGCAAACACTCAGATTCTTTGCATTCCAAAGGAGGTGACAATTCAGCAAGAGGAACAATGCAAAGTGTGCACACC
CCAGATAATGTAGCAGTCACAAACAACCCTTCTCCCTCTAATGAGGGAAAGAGAATTGCTTCCACTAATAATGAACAAGA
TACATCAAGTCCTTCGACTTCAGAAATCACAGCTACTGAGGAAGAGAATCCCAAGATCATCACCAGAGGAACCTATGACG
GTGAGATCTCATTTGGGAGTAGCTGGAAAAATGTGGGAAGTAGCAGGGGCATCAGAGAAATTGGCTCAATAACTGAAGAA
GATGATGCTGAAAAATTGCCTGAACTTATTCCATAG

>PvMLO19 Phvul.011G172300
ATGGAAAAAAATGTACAAGAAACAAGGTCATTGGCCTTGACTCCTACATGGTCTGTTGCTACTGTGTTGACTGTCTTTGT
TGCTGTCTCTCTGCTTGTGGAGCGCTCCATTCACCGTTTAAGCAATTGGCTGCGGAAAACTAATCGAAAACCGTTGCTTG
CAGCTATAGAGAAGATGAAGGAAGAATTGATGTTACTCGGGTTTATATCACTCTTATTAACAGCTACCTCACGCATGATA
GCTAATATTTGCATTCCATCAAAGTTCTACAACAGTGCTTTTGCTCCATGCACTCGATCCCAGATTGATGAACAAATGGA
AGAGAATGGCTCTGAAGAGCGTAAATTACTGATGGCTTCTGCTTATCATGTATTTGGAAGGATGTTAGATGGAATAAATA
GAAGCACTTGCAAAGAGGGTTATGAACCATTTGTTTCCTATGAAGGTCTAGAGCAGTTGCACCGGTTCATTTTTGTCATG
GCAGTAACTCATATATCTTACAGTTGCCTTACTATGTTGTTGGCCATTGTGAAGATTCACAGTTGGAGAGTGTGGGAGGA
TGAAGCTCACATGGATAGGCGTAATTCATTATCGGAGATTACAAGAGAGCTGACAATGAGAAGGCAATCAACCTTTGTCA
AGTCTCATGCATCAAATCCATTGTTCAAAAACAGTTCCCTCATTTGGGTGACATGTTTTTTCCGACAATTTGCACATTCT
GTGGTTCGTGTTGATTATCTTACGCTTCGCAAAGGCTTCATCATGAATCACCACCTTTCATTAAAGTATGATTTCCATAG
CTACATGGTTCGGTCTATGGAAGAGGAATTCCAAAGGATAGTTGGTGTGAGTGGCCCGCTCTGGGGATTTGTTGTTGGCT
TCATGCTTTTCAACATTAAAGGATCTAACCTATATTTCTGGATAGCTATTATTCCTGTATCTCTAGTGCTATTGGTGGGA
ACAAAGCTACAACATGTTATTGCAACCTTGGCGTTGGAGAATGCTGGAATTGCAGGGTTCTTTCCAGAGGCAAAGTTGAG
GCCTCGTGATGAACTTTTTTGGTTTAACAAGCCTGAACTATTGTTGTCCTTGATCCATTTCATTCTCTTCCAGAATGCAT
TTGAGCTGGCTTCATTCTTTTGGTTCTGGTGGCAGTTCGGATATGATTCTTGCTTTATTAGGAATCACCTTCTCCTGTAT
TTAAGGCTAATATTGGGGTTTGCAGGACAGTTTCTTTGTAGCTACAGCACCTTGCCACTATATGCACTTGTTACACAGAT
GGGAACAAACTATAAGGCCGCATTAATCCCACAGAGGATAAGAGAAACAATTCATGGATGGGGTAAGGCAGCTAGGAAGA
AGAGAAGACATGGTATGTTCCCTGATGACTCCGTCACCATTCATACAGACACAAGCACTGTGTTGTCCATTGAAGAAGAT
GACCAATTAATTGATGCCCCTGAAGTAGAATTACAACCAGTCACGGCTGTTACATCCACCCCTTCTCCCATTGCTAATGA
AACTTCAAGCAGGGTAGTTACACCCCTCCTAAGACCCTCTGCCTCAATATCTTCAGTGCATAGCAGTTCTAAAGCTGAAG
TAATTATAAGATGCTCCTCTATGCCAAGTGGGAGTTAA

>VrMLO1 JJMO01000124
ATGGCATCCAAAGTTTATGAGCGAACTCTTGAGGAAACTCCAACATGGGCTGTTGCAGTTGTGTGTTTTGTGCTGCTTGC
TGTTTCAATCGCCATAGAACATCTTATTCATGGTATTGGAAAGTGGTTTAAGAAGAAACGAAAAAATGCTCTATTCGAAG
CGTTGGAGAAGGTCAAAGGAGAGCTTATGGTGCTAGGATTCATATCCTTGCTCTTAAGTGTTTTGCAAGATCAAATTTCA
AAGATCTGCGTGTCGAAACATGTTGCATCCACGTGGCATCCTTGTGAGAACCCAAAGTCCTCAACTACTACATCTGAGGA
TGAATCTGAGGACATTCAGATAAATTATAGGAAACTGCTACAACTTTATGACTCTATACCAAGGCGTGTTCTCGCGACAA
AAGGTTACGACAAGTGCGCTGAAAAGGGAAAAGTTGCTTTTGTTTCAGCATATGGGATTCACCAGCTGCATATATTCATC
TTTGTGTTAGCAATATTTCATATCCTACAGTGCATAATAACACTGGGTTTGGGAAGAACCAAGATGAGAAAATGGAGGGC
ATGGGAAAATGAAACCAAGACCATTGAATATCAATTTTACAACGATCCTGAGAGGTTCAGATTTGCAAGGGACACAACAT
TTGGAAGAAGACACTTGAGTCCTTGGAGTCAGTCAACAATTTCCTTATCCATTGTTAGCTTCTTCCGACAATTCTTTGGA
TCAGTCAACAAAGTGGATTATTTAACATTACGACATGGATTCATCACGGCGCATCTGGCACCGGGAAGTGATGCAAGATT
TGATTTCCAAAAGTACATCGAAAGATCACTTGAAGAGGATTTTAAAACTGTTGTGGGAATAAGCCCAGTCATATGGTTCT
TTGCGGTGCTATTCCTTCTGACAAATACTCATGGGTGGTATTCTTATTATTGGCTTCCATTCATCCCATTTATTATAATC
TTAATAGTGGGTGCTAAACTTCAAATGATAATAACAAAGATGGGACTGAGGATTACAGACAGAGGAGAAGTTGTCAAAGG

TGCACCTCTGGTTGAACCAGGAGATGACCTGTTCTGGTTCAACCGTCCACGCCTCCTCCTCTTTTTGATTCATCTCGTGC
TCTTTCAGAATGCTTTTCAGCTGGCATTTTTTTCTTGGAGTACTTATGAATTCTCCCTACACTCTTGCTTCCACGAAACA
ACTGCAGATATTGTCATTAGACTTACAATGGGGGTTATCATACAAGTTTTATGCAGCTATGTGACACTACCTCTATATGC
TCTAGTCACACAGATGGGTTCAACCATGAAACCTACCATCTTCAATCAGAGAGTGGCATCAGCACTGAAGAACTGGCACA
ACACTGCCAAAAAGCAAGTAAAAAGCAGTAAGCCAACCACACCATTATCAAGCAGGCCATCAACGCCAACCTATGGTATG
TCTCCTATGCATCTGCTGCAAAAACACCTTGCTGGTAGAAGTGATAGTGCACAAACATCTCCTAGGACATCCAACTATGA
AAATGAACAATGGGATATTGAAGGATCACCTTCCAAAAGCAACCATGCAGCTGGTGAAGAAACTCAAATGCAAGTTATGG
AGTCAGGCTCAAGCTCAGCACCAGAATTGCCTACAAGCTCTCAGCTTGAAATCAGAGTTAGTTCATCAGAGTTTTCCTTT
GAGAAGAGACATCTTGGTGCAGAGACGAGAAGTGAAGATGATAGTTTTTATTAA

>VrMLO2 JJMO01000125
ATGGCAGGTGGAGGTGGAGGAAGAAACTTGGAGGAAACACCCACATGGGCCGTCTCCACTGTCTGTTTTGTTTTGATCTT
AATATCTATAATCATCGAACATATCATCCACCTCATCGGAAAGTGGTTGAAGAAGAAGCACAAAAGAGCGCTGTACGAGT
CACTGGAAAAGATCAAATCAGAGCTTATGTTGCTGGGGTTCTTATCGTTGCTTTTAACGGTGGGACAAGGTCTGATATCA
AGAATATGTATATCAGAGAAAGTTGCATCAACATGGCACCCTTGTAGCCACGAAAACACAGACACTACTGAAGAGTCTGA
ACAGGGAACCAACAGCCGCAGGTTGTTAGCGGCGTTCCATGGTTCAAACGAGGTAACTTCACGCCGTGTTTTGGCTGGAG
GAGGAAGCGACAAGTGTGGCGAGGGTAAAGTGCCGTTTGTGTCATCCGATGGCATCCATCAACTCCATATATTTATCTTT
GTGCTAACTGTTTTTCATGTTTCTTACTGCATTACCACCATGGCTCTAGGAAGAGCAAAGATGAAGAGATGGAAAAGATG
GGAGGAGGAAACCAAGACACCAGAGTACCAATTTTCACATGATCCTGAACGATTTAGATTTGCTAGAGAAACATCGTTTG
GAAGAAGGCATTTGAGTTTCTGGACCAAAAACCCTGTCCTTATGTGGATTGTTTGTTTCTTTAGGCAATTTGTGAGATCA
GTACCTAAAGTGGATTACTTGACCTTAAGACATGGATTTATCATGGCACATTTGGCTCCTCAAAGTCACTCCAAATTTGA
TTTTCGGAAATACATAAAACGATCACTGGACGAGGATTTCAAAGTGGTTGTCGGAATCAGTCCTCCATTCTGGTTCTTCG
CGGTGCTGTTTCTTCTCTTGAACACTCATGGCTCGTACTCTTATCTGTGGCTGCCATTTATTCCTTTGATCATCATTCTG
TTAGTTGGAACCAAGTTGCAAGTGATCATAACTGAGATGGGTCTGAGAATTCAACAAAGAGGAGAGATTCTAAAGGGAGT
GCCATTGGTACAACCAGGGGATTATTTGTTCTGGTTTAACCGACCTGCTCTTATTCTTTACCTTATTAACTTTGTGCTCT
TTCAGAATGCTTTTCAGCTGGCTTTCTTCTCATGGTCTGTGCTTCAATTTGGGATTAAGTCTTGTTTCCACGCACATACT
GAGGATGTAGTGATCAGAATCTCAATGGGGGTCCTTATTCAAATCCTGTGCAGCTACGTCACTCTTCCTCTATATGCTCT
GGTGACACAGATGGGTTCAACGATGAAGCCAACGATATTCAACGAAAGAGTTGCGGAGGCACTGCGGAAGTGGCACCAGA
CAGCGAAGAAGCAAATAAGACAGAACCGTGTGGGATCCTTGTCGTTGTCGGGGACACCCATGTCGAGCAGACCCACGACC
CCGAGCCACCACATGTCTCCAATGCACCTCTTGCGCTACTACCGTTCTGAAATAGACAGCTTCCCCGCCTCTCCACGGCG
TTCGAACTTCGACGGCGACCACACTCAGCCCTGGGAAATCGATTCCCCTTCTCCTTCGTACTCGCAGCACGAAGTGGAAA
TGGGTCCCACCAATGACCCCAATACTAACACAAGCCAACATGAAATTGTTACGATTGCACACACCAAGGAATTTTCCTTT
GATAATAGACCTACCACTGCCACGTAG

>VrMLO3 JJMO01000125
ATGCTTACACATCACAATTATAGTTTTGTCTTCTCTTTTCTCAAGATCTTTGGCAGAAAGTGTAGAAAATTAAGGAATCA
AAGGCTTGTTCTTATGGAAATAGGAGAAGAAAGATCCCTGGAAGAAACTGCAACATGGGCTGTATCAGTTTTTTGCTTCT
TCCTTCTTCTAATATCTTTGATCATTGAGGGTAGTCTCCACAAGTTGTCTGAGATTTTAAGGAAAAGGAAAGGAAAATCA
CTAGGGAGGGCATTGACTAAAACCAAAACAGAAATGATGAAATTCGGTTTCATGTCCTTTCTTCTCACCATCATGTCGGA
AGTGCCAATATCGAAAATATGCATCAACAAGGATGTGGCAACATATTTTCTGCCATGCAAAGATGCTGCAGAATTAACAG
GGTTTTCAGCTACTCATACATCAACCTCACAATTTGACGTAGCACCTGCAACAAACGAATCTGAAGTTGAGGTCAACTAT
TGTGAAGCAAAGGGTATGGTTAGTTTAATTTCAAGTGATGGAATCCTGCAACTGAATATATTCATCTCCTTTCTGGCTGT
GTTTCACATCTTGTTCTGTACATTGACAATGTGCCTCGGGAAGGCAAAGATGAGAAGATGGAAAAGATGGGAGGAGGAGA
CTCGAACACTGGAATATCAGATAGCTAATGACCCAAGAAGGTTCCAATATACAGGTCAAACAATGATTGGAAAACGTCAT
TTGAATTTCTGGAGTTATCATTCTCCCTTGCTTTGGATGGTTTGCTTTGTTCGGCAGTTTTATGTCTCAGTCTCAAAAGA
TGATTACTATACTCTACGAAATGGGTTCATTGCAGCTAATATTTCTTTAGGCAGCAATTTCAACTTCAAGAAATTTCTTT
CCCGCACTTATGATGAAGATTTCGAGAAGGTTATGGGAATCAGGATTTGGATCTGGATTTTCTCCATACTTTTCATCTTT
TTTAGTGCACATGAGTTCTATAATTACTACTGGCTTCCATTTATTCCACTTATGGTTGCTTTACTAGCTGGGACAAAACT
CCAAGTCATAATAACAAAGATGTGTGTAGATAGTTGCAAGGCAAACCCAGTGATCAAAGGAAGTCTACTAGTAACACCCA
GTGATGCCCATTTCTGGTTTCATAGACCTGAATGGCTTCTGCATTTACTCAAGTTCATCCTTATCCAGAACTCCTTTCAA
CTTGCATTCTTCACCTGGACTTGGTATGAATTTGGTCCAAGGTCTTGCTTCAATAGAGAAAATGAGGACATTGGCATAAG
GATTGGAATGGGTATAGCTGTGCAGCTTCTTTGTGGTTATGTCACCCTTCCCCTTTTTGCACTTGTTACTCAGATGGGTT
CTAGCATGAGGAGAGAAATTTTCACCGAGAGTGTTTCTATGGGCCTCAAAAATTGGCACAAAAGGGCTAAACAATCTCTG
CAGAACAACTCAACTTCAAGAAAACACTCAGACTCTTTGCATTCCAAAGGAGGTGACAATTCAGCAAGAGGAACAATGCA
TAGTTTGAACAACCCAGATATTGTAGTGCTCACAAACAACCCTTCTCCCTCTAATGAGGGAGAAAGCATTGCTCCCACCA
ATAATGAGCAAGATGTATCAAGTCCTTCGACTTCAGAAATCACAGCTACTGAGGAAGAGAATTCCAAGATCATCACCAGA
GGAACCTATGACGGTGAGATCTCATTTGGTAGCAGCTGGAAGAATGTAGGAAGTAGCAGAGGCATTGGAGAAATTGTCTC
AATAACTGAAGAAGATGACGTTGAAAAATTACCTGAATTTATCCATAGATAA

>VrMLO4 JJMO01000126
ATGGCAACACCCAGTTATTCTTTACAGCACACTCCAACATGGGCTATTGCCCTTGTTTCCTTCATTCTTATCTCAATCTC
AATCATCTTGGAGCATTTCATCCATCTCATAATCAAATGGCTCAGGAAACATCGAAAGAGCGATTTGGTGGAAGCAATAG
AGAGGCTTAAATCAGAGTTGATGATCTTAGGATTCATGTCGCTGTTGTTGACAGTAACACAAGACGCCATAATAGAAATC
TGCATTCCAGTGAGGGCTGCAGATACTATGCTTCCATGTCGAAAACGTACTGCCAATGATACTGCAATTTTGGATTCTTG

CAGTGCCAAAAGTGCTAGTGAGGTTGCTTTAGTGTCAAAACATGGAATCCATCAACTTCATATGTTCATTTTTGTTTTAG
CTGTCATGCAAATTGTGTACAGTATTCTCACCGTTTCTCTGGCAAGGGCTAAGATAAGGCACTGGAAAGCTTGGGATGAA
GAGACTCAAACAATTGAATATGAAATTGCCAACGACCCTAATAGATTCAGATACGCAAGGCAAACAACATTCGGAAGACG
CCACATATCTACTACTACTGCATCACCAGTGTCTGTTTGGATTAAATGTTTTTTCAGACAGTTCTTTCATTCAGTAGAAA
AAGTTGATTACCTCACCTTGAGGCATGGTTTCATATCAGCTCACTTTTCTGCTAGAAACAGCAACTTCGACTTCCAAAAG
TACATTGAACAGTCACTTGAGGAAGATTTCAGAATCATAGTGAGCATTAGCCCTGTGATGTGGTTCACTGTTGTTATTTT
TCTGCTCGTGGATGTACATGGCTGGCATGTATATCTCTGGTTATCCTATATTCCCCTCTTGCTGGTTCTGGTTGTAGGAG
CTAAACTTGAAGTCATTGTAGACGAAATGGCTCTTCAGATGAAAGATGTCAACAGTGTAACCAAAGGGACCCCACTTGTT
TCTCCAAGTGATGAATTCTTCTGGTTTGGCCATCCAGGATTTGTGTTAACACTTCTGCATTACACTTTATTTGTGAATGC
ATTCGAGCTTGCCTTCTTTATATGGGTATCAACACAGTTTGGAATAAATTCTTGCTACCACGAACACAGAACATTTACAG
TCATAAGGGTGGTGATAGCGGTAGCAGTTCAAGTCCTATGCAGCTATGTAACTCTTCCCCTTTACGCACTAGTGGCACAG
ATGGGGTCACAGGTGAAGAGCAAAGCCTTGGCTAAGATTCTGAGGCAGTGGCATGTTGAGGTGAGGGAGAGAAGAAGGAA
GCAACAGAAACTTGTGAAGTCTTTTAGTTTTAGCCATATGTCTTCAGAATGGAACCAAGGAAATAAGAGTGCCCTTGAGT
TTTCTTCAGGGCTACGTGAAAACGCTCGTTCCTCTAGTGAAGGAGAAATTGTAGAAGCGTTGGAGCATCCGATGATAACA
CAGGCAAACTCATCAAGTGATCCACCTCCTAGTGTTGTCTAA

>VrMLO5 JJMO01000120
ATGGCAGGAAGTGGAGGAAGAAATTTGGACGAAACACCTACCTGGGCGGTGGCCACTGTTTGCTTTGTTTTGCTCTCTGT
ATCTATCATCATCGAATACATCTTCAATCTAATAGGAAAGTGGTTGAAGCAGAAACACAAAAGAGCTCTGTACGAGTCAC
TTGAAAAGATCAAATCAGAGCTTATGCTATTAGGGTTCATATCGTTGCTCCTGACGATAGGGCAAGGTCTGATATCGAGA
ATATGCATATCAGAAAATGTTGCAGGGACGTTTCACCCTTGCACTTACAGAAGGGAGGAGAAAAAGTACCCCCCGCCATT
AGAAGACGACTATTACCACCATAACCGCCACATTTTAGCGGCGGAAGCAGGTTACGACAAGTGTGCCGCCCAGGGTAAAG
TCCCATTTGTCTCATCGGGAGCCATTCATCAACTCCATATATTTATCTTCGTGCTAGCTGTTTTTCACATCCTTTACTGC
ATTCTCACTCTGGCTCTGGGTAGGGCAAAGATGAGAAGGTGGAAACGATGGGAAGTAGAAACCAAGACAGCAGAGTACCA
ATATTCACACGATCCCCAGCGATTCAGATTTGCAAGAGAGACATCGTTTGGAAGAAGACACTTGAGTTTTTGGACTCAAC
ATGTTGTCCTGGTTTGGATAGTTTGTTTCTTCAGACAGTTTGTACGCTCAGTTCCTAAGGTTGATTACTTGACCTTGAGG
CATGGATTTATGATGGCGCATTTGGGGCCACACAGTCACCAGAAATTCGACTTCCGGAAATATATTAAAAGATCTTTGGA
AGAGGACTTCAAAGTGGTCGTCGAAATCAGTCCTCCAATCTGGTTCATTACAGTGTTTTTTCTCCTGTTTAACACTCACG
GCTGGTACTCTTATCTGTGGCTTCCATTTGCTCCTTTGATTATTGTTCTATTAGTGGGAATGAAGCTGCAAGTGATAATA
ACGAAGATGGGTCAAAGAATTCAAGAACGAGGAGAGGTTGTAAGGGGCATGCCTCTGGTGCAACCAGGGGATCACCTCTT
CTGGTTTAACAAACCTCGACTTATTCTATACCTCATTAATTTTGTGCTCTTTCAGAACGCTTTTCAGCTTGCTTTCTTTT
CATGGGCTGCGCTTCAATTTATGATGAAGTCCTGTTTCCTTTCCCAAAAGAAGGAGGTGGTGATCAGAATCTCAATGGGG
ATCTTCGTTCAATTCCTTTGCAGCTACGTCACTCTTCCTCTCTACGCTCTCGTGACGCAGATGGGTTCAACGATGAAGGC
AACGATATTCAACGAAAGAGTAGCGATGGCTCTGAGAAATTGGCACCACACAGCGAAGAAGAACGTAAAAGAGAAGCGTG
GATTACGATCGCAGAGTCCATTGTCAACGAGGCCTTCAACACCGAAGCATCCTAAGTCGAAGGCTAATCTGCTGCGACGG
TATCACAGTGAAATGGCCACTTATCCATCTTCTCCTATAAGGTTCGATTTTGAGGCACATCTTCCTTATGGGATCCATTC
ACCTCCTTCGTCCAAGGTTAATGCTGCAGCTACTTCTTCCATTGATCAGAATGAAATGGAGATGGAGGTGGAAATGGAAA
TTGACATGGATCAGGTCTCAGATCTCACTCAACACCACATTGATATCCAAGACAAGCAAGGATTTTCTTTTGATAAAAGA
TGA

>VrMLO6 JJMO01000120
ATGGAAGGAAGAAGCTTGGAGAATACTCCGACTTGGGCTGTGGCCGTTGTTTGCTTCGTTTTGCTCTCGGTATCTATCAT
CATCGAATACATCTTCAATATAATAGGAAAGTGGTTGAAGAACAAGCACAAAAGAGCGCTGCATGAGTCACTTGAAAAGA
TCAAATCAGAACTTATGTTATTGGGGTTCATATCGCTGCTTCTAACGGTAGGACAAAGTGTAATATCGAGGATATGTATA
TCAGAAAAGGTCGCCGGAACATTCCACCCCTGCACTCACCAAAGCGACCACAAAACCGATGCCCGGATATTACTATCGGA
GCTTTTCGGTTCCGATCATGGCCAAAATCAACGCCGTATTTTGGCTGCCGGAGGAACTGACAAGTGCGCGGCTCAGGGTA
AAGTTCCGTTTGTCTCATCAGAGGGCATTCATCAACTTCATATATTTATCTTCGTGCTGGCTGTTTTTCACGTTCTTTAC
TGCATTCTCACTCTGGCTCTTGGTAGAGCAAAGATGAGAAGATGGAAACGATGGGAAGAAGAAACAAAAACAACACAGTA
TCAATTTTCACACGATCCAGAACGGTTTAGATTTGCAAGAGAAACATCGTTCGGAAGAAGACACTTGAGTTTCTGGACTC
AAAATACTGTCCTTGTTTGGATTATTTGTTTCTTCAGGCAGTTCGTACGCTCAGTTCCTAAAGTTGATTACTTGACCTTG
AGGCATGGATTTATGATGGCACATTTGGGGCCTCAAAGTCGCCACAAATTTGACTTTCGGCAATATATTAAAAGAACTTT
GGAAGAAGACTTCAAAGTGGTGGTTGAAATCAGTCCTCCAATATGGTTCATCACAGTGCTTTTTCTCCTGTTTCACACAC
ATGGCTGGTACTCTTATCTGTGGCTGCCATTTGCTCCTTTGATCATTGTTCTATTAGTGGGAACTAAGCTGCAAGTGATA
ATAACGAAAATGGGTCAAAGAATTCAAGAACGTGGAGAGGTCGTAAAGGGTGTGCCACTGGTTCAACCTGGGGATGATCT
CTTCTGGTTTAACAAACCTCGACTCATTCTCTACCTCGTCAATTTCGTGCTCTTTCAGAACTCTTTCCAACTCGCCTACT
TTTCATGGACTGCGCTTCAGTTTGGGATGAAATCTTGTTTCCATTCGCAGACGGAGGATGTGGTGATCAAAATCTCAATG
GGGGTTTTCGTTCAATTCCTCTGCAGCTACGTAACTCTTCCTCTCTATGCTCTCGTCACACAGATGGGTTCGACCATGAA
GCCAACCATATTCAACGAACGAGTAGCCATGGCTCTGAGGAATTGGCACCACGGAGCCAAGAAACACGTAAAGCAACAGA
ACCGTAGATCACAGTCGCAGGCCCCTTTGTCAGGGCCCACAACCCCCGATCATTCTATGTCTCAGGCCCATCTCTTACGC
CGTTGGCACAGTGAAATGGACACCTATCCCACTGACTCTGAAGCCCATCGTCCCTGTGAGATCGATTTAGGCCCATCATC
CAAGGTGCATCATCAGGAAATGGAAATGGATCAGCTGGACCATGGCCCACCACAAAATGTCAATGACCAGACTCAACACG
AGATTAATATTGAACATGGCAAGGAGTTTTCGTTTGATAATAGATGA

>VrMLO7 JJMO01000121
ATGGCCGGTGGAGCATCCGGCGGAGAAAGAACGTTGCAGGAAACACCCACATGGGCAGTGGCTGCTGTTTGCGCAGTCTT
CGTTATTCTCTCTGTGTTGATTGAACAGGGAATCCATTTACTTGAAAAGTGGTTTAAAAAACGACATAAGAAAGCCATGA
GTGAAGCCTTGGAGAAAATCAAAGCAGAGTTAATGTTATTAGGATTTATATCCCTGTTACTTACTTTTGGTACACAATAC
ATTGCAAAGATATGCATCCCTGCATCAGCTGGTGATATCATGCTTCCATGCAAGAAGGTGGAAGCGTCAAAGGACCCAGA
TGATTCAAATGGCGGAAGGAAGTTACTTTACTTTGAGGACAACATGGAATGGCGTCGAGTTTTAGCAACTGCGTCTTCTG
GTGGTGATTATTGCTCACAGAAAGGTAAAGTGCCATTGATTTCTCAGTCAGGGGTGCACCAGTTGCACATATTTATCTTC
GTCCTCGCTGTTTTTCACATATTCTACAGCGTAATGACCATGGTACTAGCCCGAGCAAAAATGAAGAAATGGAAAGCTTG
GGAAGCTGAAACATCCTCTCTCGAGTATCAATTTACAAATGATCCTTCGAGGTTCAGGTTTGCGCATCAAACATCGTTTG
TTAGGCGTCACTCTGGTTGGTCTAGGATGCCAGGAATTCGATGGATTGTAGCATTCTTCAGGCAATTCTTTGCGTCTGTC
ACCAAGGTTGATTACATGACCATGCGGCATGGATTTATTAACGCACATTTTGCTCCCAATAGCAAATTTGACTTCCAAAA
GTACATTAAAAGATCCATGGAGGACGACTTTAAAGTGGTCGTGGGTATAAGTATTCCGTTATGGGCTTTCGCTATCGTCT
TTATGCTGGTGAACGTTTACAAATGGTACACTCTCACCTGGCTATCGTTAGCGCCGCTAGTGATACTTCTATTAGTGGGC
ACCAAGCTTGAACTTATAATCATGGAAATGGCCCAAGAAATTCAAGATCGAACCACAATTGTCAGAGGAGTCCCTATTGT
AGAGCCTAACAACAAGTATTTTTGGTTTAATCGGCCCCAGTGGATTACCTTCTTAATCCACTTTACCTTATTCGAGAATG
CATTCCAAATAGCCTTTTTCTTGTGGACATGGTATGAATTCAAGATCACATCTTGCTTCCATGAAAACTTGCCGCTGATA
CTGACAAGGGTTTTCCTTGGAATAGTCTTGCAAGTCGTGTGTAGTTACATTACATTTCCTCTCTACTCCCTAGTAACACA
GATGGGATCTCACATGAAAAAAGCAATTTTTGAAGAGCAAACTGCAAAGGCACTAAAGAAATGGCAGAGGGTTGCAAAAG
ACAAGAGAAAGTTGAGAAAAGCAGGGGTAGATGTTCCTTCGGGAACAATGAGTGGGGAAGCAACACCAAGCCAAGGATCA
TCACCAATACATTTACTTCACAAGTACAAGCCTAACCAAACAGACACTGATACTGTTCTTTATTCTCCACGATCATACCA
ATCTGATACCGACTTGTCTGACACAGAAGGATCATCGTACCAATTGAATGAAATAATACAGACACAGACACCTCAAGCTC
CAAGAAATGGAGAACCTCATATTACCGAATTTTCCTTTGTAAGCTTTAAAAGTGAACAATTTTGA

>VrMLO8 JJMO01000121
ATGTCGGGAGGAGGAGGCGGTGACAACAGGCAGCTAGATCTCACGCCAACATGGGCCGTGGCTGCGGTTTGTGCAATCAT
CGTCATCATTTCTATACTCTTGGAAAAAATCATACACAAATTCGCAGCGGTGTTTGAAGAAAAAAAGAAACATGCGTTGC
TAGAAGCTCTTGAAAAGATTAAAGCCGAGCTTATGGTTTTGGGATTTATTTCGTTACTTTTAACGTTTGGTCAAAACTAC
ATTTCTAAAGTGTGTATTCCCCAAAGGTATGCACACACAATGCTACCGTGTCTTCCCCTTGAACAACGAGGAGGGGGCGC
GGCAGCTGAACACGGAGCGGGTGGCGGTGAGGCTGAAGGAGGTAGCCATCATCGCAGACTTTTTTCATACGAGCGTAGAG
TTCTATCTGGTGGTGGTGGAGGCCCCAGTTGTAAACCGGGGTTCCTGCCTCTTATATCAGTGAGCGGGTTGCATCAGTTA
CACATTTTTATATTCTTCTTGGCTGTCTTTCATGTGATCTACAGTGCCATAACAATGACCCTTGGAAGAGCAAAGATTCG
TGGGTGGAAGGCATGGGAGGAGGACCATGTCGTGGATGAAGATGCCCTGAATGATCCTAGAAGATTCAGACTTACTCACG
AGACATCATTTGTGAGGGATCACAGCAGTTTCTGGACTAAAACACCAGTTTCCTTCTATTTTGTATGCTTCTTTCGACAA
TTTTTCAGATCTGTTCGTAGGGCTGACTACTTGACCATGAGACATGGATTTGTTTCTGTCCATTTAGCACCAGGAAGTAA
GTTCGATTTTCAAAAATATATCAAGAGGTCATTAGAAGATGACTTTAAGGTAGTTGTAGGAATCAGTCCGATACTTTGGG
GATCAGTAGTGTTATTCTTGCTCGTGAATGTTCACGGATGGCATGCTGCCTTTTGGGTGTCTTTTCTCCCTCTATTGGTG
ATTCTGGCTGTTGGAACAAAACTTCAAGCAATTATTACTCGGATGGCACTTGACATAAAAGAAAGGCATGCTGTAGTGCA
GGGGATCCCTCTAGTGCAAGTGTCTGACAAATATTTTTGGTTTGCATGGCCTCAGTTAGTTCTTTATCTCATCCATTACG
TCCTCTTTCAGAATGCTTTTGAGCTGACATACTTCTGGTGGACATGGTATGAATTTGGTTGGGCATCTTGCTTTTACGAG
GATGATAAACTCATGATCGTCAGAGTAGCTCTTGGGCTGGGAGCTCAAGTCGTTTGCAGTTATGTCACACTTCCACTCTA
CGCACTGGTTACTCAGATGGGGTCGACGATGAAGAAGTCAATATTCGATGACCAAACATCGAAGGCGCTGAAACAGTGGC
ATAAGAATGCTCTGAAGAAGAAAACGTCAAAAGGACGCCAGGAAACTCGAACGTTGGGTGGGAGCCCGGGAGAACATACA
CCGGGACATTCTTCGCCGCACCACCCACCACCGCACAAAGACACGGAAATGGCTTCTCAGACCGCCACCATCGTCACCAG
TGTTGATAACGAAACCTATGATAAGCGGGACCTTCTGAGTGGACCGTGA

>VrMLO9 JJMO01000121
ATGAGTGGCGGAGGAGAAGAAGGAGAGAGTCTGGAGTTCACTCCCACTTGGGTGGTGGCCGCCGTATGCACAGTCATCGT
CGCCATCTCCCTCGCCGCAGAGCGCTTTCTTCATTTTGGCGGAAAGTATCTCAAAGCTAAGGACCAGAAGCCGCTCTACG
AAGCACTGCAGAAAATTAAAGAAGAGCTCATGCTTTTGGGTTTCATTTCCCTCCTTTTGACGGTTACACAAAACGGCATT
ACCAAAATCTGCGTTCCCCCGGCCTTCACGCGCCACATGCTTCCGTGTAGCCTTGACCTCGCTCCCAAACATGAATCTCA
TTTCCAAACATTTTATTTCCCGGGAACACCCAGACGTCTTCTCGCTGCCACTGCGGCGGAACACGCGCCGCCCCACTCCG
AGGCTGAGGCGACCGAGACTGGAACCGAGACTGGGTTCTGCGCTCGCAAGAATAAGGTGCCTTTGTTGTCTGTGGAAGCA
CTGCACCACCTCCACATCTTCATTTTTGTATTGGCTGTCGTACACGTCGCCTTTTCCGTACTCACCGTTGTCTTTGGAGG
TGCCAGAATTCGTCAATGGAAACACTGGGAAGATTCAATTGCAAAAAAGAACTACGAAACCGACCGAGTTCTGAAACCAA
AGGTGACCAATGTTCAGCAGCATGATTTTATCAGGGGCCGTTTTTCTGGTTTTGGGAAAGATTCTGCTATAGTCGGTTGG
ATGATATCCTTTTCTAAGCAATTTTATGGATCTGTGACAAAATCGGATTATGTGACGCTAAGACATGGTTTCATTATGAC
CCACTGCAAGTCAAATCCAAAGTTTAATTTTCACAAGTACATGATTCGTGCCCTTGAAGATGATTTCAAGCAAGTTGTTG
GTATAAGTTGGTATCTTTGGCTCTTTGTGGTTATCTTCTTGTTGCTCAATATCAACGGTTGGCATACATATTTCTGGATT
GCTTTTATTCCTGTCATTCTTTTGCTTGCTGTGGGCACTAAGCTCGAGCATGTAATAACCCAACTAGCTCATGAGGTGGC
TGAAAAGCATGCAGCCATAGAAGGTGACTTAGTTGTGCAGCCATCAGATGAACACTTTTGGTTTCATCGGCCTCGTGTTT
TCCTCTTCTTAATTCACTTTATCCTTTTCCAAAATGCCTTTGAGATAGCATTTTTTTTTCTGGATATGGGTTACATATGGG
TTTGACTCGTGTATAATGGGGCAAGTTCGATACATTATTCCAAGGCTCGTTATTGGGGTATTTATTCAGGTATTATGTAG
CTACAGCACCCTGCCACTCTATGCAATAGTTACGCAGATGGGAACTCACTATAAGAGGGCAATATTTAATGAGCATTTGC
AACACAACATTGTTGGTTGGGCACAAAAGGCGAAGAAGAGGAAAGAACTAAAAGCTGATGGCCGTCCTGGCCAAGGAAGT

TCTCAGGAGAGTGCTAATACAGGAATCCAGCTTGGCTCAATTTTCACGAAGAAGGCATCTGCTCCAGGCGACACTTCTTC
TGCCCCCAAAGATGACGGAACCAGCTGA

>VrMLO10 JJMO01000123
ATGTCTGTCTCAAAATACAAAATACTGTCTCTTTGTCTGGTTTCATGGCTGTGGTGCGGTTATGTGGCTGTGGCTTCAAG
TGAGAGTAGTATCGGTTCCAAAGACCTTGATCAGACACCAACATGGGCGGTTGCCTGTGTCTGCACTGTTTTCATCTTGA
TATCCTTAACTCTGGAGAAAAGCCTTCACAAAGTGGGAACGTGGCTACATGAAAAGCACAAGAAGGCTTTGCTTGAGGCT
TTGGAAAAGGTCAAAGCTGAGTTGATGATTCTTGGTTTCCTTTCACTGCTTCTTACTTTCGGGCAGAGTTACATTGTCAG
AATATGTATTCCTACTGATATTGCAGACAAATTGTTGCCATGTCCGCTTGCTGGTACCGAAGAAGAATCAAGTAGTGAAG
AGGAACACCGTAGGAAGCTTCTTTCTTATGAACGCAGATATTTGTCAGGTGACGCTACCAATTACCAATGCAAGAAGGGA
CAACAACCACTTATATCAGTCAATGGATTGCACCAGTTACACATCCTCATATTCTTCTTAGCAGTCCTTCACGTGTTTTA
CAGTGCTGTAACAATGCTTCTTGGGAGACTAAAGATTCGAGGATGGAAGGCATGGGAGGAGGAGACTTCATCTCATGGAT
ATGAGTTTGCCAATGATCCCTCAAGATTCCGCCTTACACATGAAACATCATTTGTGAGAGCTCATGCTTCTTTTTGGACA
AGATATTCAATCTTCTTCTATATAGGATGCTTTTGTAGGCAATTTTATAGGTCTGTAGGCAAGGCTGACTACTTGGCTTT
GCGCAATGGATTTATCACTGTACACCTGGCTCCTGGAAGTAAATTTAACTTCCAAAAGTATATCAAAAGATCCTTGGAGG
ATGACTTCAAGGTTGTTGTTGGAGTCAGTCCGATTCTCTGGGCTTCATTTGTTGTATTCCTGCTACTAAATGTTAATGGA
TGGCATGCTATGTTCTGGGCATCCTTAATTCCAGTTGTGATAATTTTGGCTGTCGGAACAAAACTGCAAGTCGCACTCGC
AAAGATGGCTATTGAAATAACAGAAAGACATGCAGTTGTCCAAGGAATTCCTCTTGTTCAAGGCTCAGACAGATATTTTT
GGTTTGGTCGGCCTCAGTTAGTTCTTCATCTTATCCATTTTGCTTTGTTTCAGAATGCTTTCCAAATAACATATTTCTTG
TGGATATGGTATTCTTTTGGACTGAAAAATTGTTTCCACGCTGACTACAAGCTTGCAATAGTGAAAGTAGCATTAGGGCT
TGCGGCACTATGCCTCTGCAGCTATATCACCCTTCCATTATATGCTCTTGTTACTCAGATGGGCTCAAGAATGAAAAAAT
CAATATTTGATGAACAAACATCAAAAGCATTAAAGAAATGGCACATGGCAGTGAAAAAGAAGCAGGGAGTGAAACTTGGA
AACTCCAAGGTGCGAGCCATGGATGGAAGCACCACTGATTCAACAGTACACTCTTCTGGCCCCAAACTTCACCGTTTCAA
AACCACTGGTCACTCAACTCGCACCATGTCAGCCTATGATGACGACCATTCTGACATTGAGCTGTCTCCCGTTTCACCGA
CAGCAAACTTGATTGTAAGAGTGGACCATGATGAGCATGAAGCAGAAGAAGCTGAGCATCACCCAACAGCCAACAATCAA
GAGCTATCACGTTTGCCAACCCTTGAAAGAAGCATGAAATAG

>VrMLO11 JJMO01000128
ATGGGAGAGGAAGTTTTACAGAGGAAGTTGGAGACAACTCCTACATGGGCTGTTGCAATTGTGTGCTTTGTCATGCTCGC
TATTTCAATCCTCATTGAACATATTATTGAAGAACTTGGAAAGTGGTTGAAAAAGAAACACAAAAAGGCTCTTCATGAAG
CGCTGGAAAAGGTTAAAGGAGAGCTTATGCTGCTGGGATTCATATCCTTGCTCCTAGTGGTGTTTGAAGATCGAATTACC
TCTATCTGCATCTCAGAAAGTGTTGCTTCCACTTGGAATCCTTGCGATCCTGATTATAAGAGTAAAGGTCCAGAAGGATA
TATTGACAAATGCGCACAAAAAGATAAAGGAAAAGTTGCTTTTATCTCTGAATATGGGATTCACCAGCTCCATATATTCG
TCTTTGTGCTTGCTATCTTTCACATCCTACAGTGCATCATGACACTGACTTTGGGTAGAACTAAGATGTCAATATGGAGG
AAGTGGGAAGACGAAACGAAGAGTCTTGAACATCAGTTCTACCATGATCCTGAGAGGTTCAGGTTTGCTAGGGATACAAC
GTTTGGACGAAGGCACTTGAACTCATGGAGTAGATCACCAATTTCATTATGGATTGTTAGCTTCTGCAGACAATTCTATG
GATCAGTTAATAAAGTTGACTATATGGCATTACGACATGGATTCATAGTGGCACATTTGACTCCAGCAAGTGAGACGAAA
TTTGACTTCCAGAACTATATAAAAAGAACACTTGATGAGGATTTTGCAGTAGTAGTTGGAATAAGTCCAACCATTTGGTT
CTTTGCAGTGCTAATTCTGCTCACAAATACTAATGGGTGGTATTCTTATTTGTGGCTACCATTTATCCCACTAATTATAA
TCTTGTTGGTGGGAACAAAGCTACAAATGATCATAACAGATATGGCACTAAGGATTCAAGATAGGGGGGAAGTGGTCAAG
GGTGCACCAGTGGTTGAGCCAGGAGATGAATTGTTCTGGTTCAGTCGCCCTCGCCTCGTTCTCTTTCTGATTCATCTCGT
TCTGTTTCAGAATGCATTTCAATTGGCGTTTTTTGCTTGGAGCACTTACGACAATAAGTTCAAAATAAACAATTGCTTTC
ACCGCACTACTGAAGATATCGTCATCAGACTTACAATGGGGGTTCTCACCCAAGTTCTATGCAGCTACGTGACTTTGCCT
CTTTATGCTCTAGTCACACAGATGGGTTCTACGATGAAACCCATCATTTTCAAAGATAACGTGGCATCAGCACTGAAGAA
CTGGCATCACACTGCTAAAAAGCATCTAAAACACAACAAGGATTCAACTTCTAACACCCCATTCTCAAGCAGACCAGGAA
CTCCAACTCATGGCATGTCTCCGGTGCACTTGCTTCACATGCACCCTAGACACAGTGATAGTCCACTGGTTTCTCCAAGA
GCATCCAATTATGAAAATGGTCAATGGGATGTTGAAGGGTCACATTCCCCAAGCCACCATGCAAGAGATGTTGACCATGA
AGAGAGCATGATGATGCAGCCTCAAGCAGCAGAATTGCCTCCTTCTGGACCCAATCCTATTCGAACTCAACATGAAATCA
ACATTGCTTTATCTGAATTTTCATTTGGGAGGGGACACAACCCCAGTAGAGCAGATGAGTATTAG

>VrMLO12 JJMO01000129
ATGGGTGGTGGTGGTGGTGGTGAAGAAGGGAACAACTTGGAATTCACTCCCACTTGGGTGGTTGCTGTTGTTTGTTCTGT
GATCGTTGCTGCATCGTTTGCTGCAGAACGGTTTCTTCACTATGGAGGGACGTTTCTCAAGAAGAAGAATCAGAAGCCGC
TGTATGAAGCTCTGCTGAAGATCAAAGAAGAGTTAATGCTGTTGGGATTTATTTCTCTGCTGCTGACTGTGACACAAAAT
GGAATCATCAAAATCTGTGTTCCTGAGGGTTGGACTCACCACATGCTTCCTTGCAGTCTTAAGGATAAAAAAGAGAAAGA
ATCAGCAAAACTCACAGAACATTTTCAGACTTTTTTCTCTTTCAGTCATATTCCTGCCACTGTTAGGCACCTTTTAGCTG
ATAATGAAGATCACCAACCAACAGCTGCTGAAAAACTTGGACACTGTGCTAAGAAGGGCAAGGTTCCTCTGTTATCTGTG
GAGGCACTGCATCATCTGCATATCTTTATTTTTGTCCTGGCCATTGTTCATGTGACATTTTGTGTTCTCACTGTTGTGTT
TGGAGGGTTAAAAATACGTCAGTGGAAGCACTGGGAAAACTCTATAGGAGATCAAATTAATGGAACACCAGATTTGGAAT
CAACAGTGACTCATGTTCGTGAACACGCTTTCATCCAGAATCGTTTTACTGGTTTTGGCAAAGAATCTACTGTTTTGGGT
TGGGTGAAATCATTTTTCAAGCAATTTTATGGATCTGTGACAAAGTTAGATTATGTGACATTAAGGCTTGGTTTCATAAT
GACTCATTGCAGAGGAAATCCAAAGTTTAATTTTCATAAATACATGATTCGTGCCCTTGAAGACGATTTCAAGAAAGTTG
TTGGAATAAGTTGGTATCTTTGGATCTTTGTGGTCATCTTCTTGTTGCTTAATATCAATGGTTGGCACACATATTTCTGG
ATTTCTTTCGTTCCTCTGATTCTTCTACTAGCTGTGGGTACGAAGTTGGAGCATGTAATAATTCAACTAGCTCATGAAGT

AGCTGAGAAACATACAGCCATAGAAGGTGAATTAGTTGTTCAACCAAGAGATGATCACTTTTGGTTTCATCGCCCCCACA
TTGTCCTCTTCTTGATTCACTTCATCCTTTTCCAAAATGCTTTTGAGATTGCATTTTTCTTTTGGATATTGTTTACATAT
GGCTTTGACTCATGTATCATGGGACGAGTCCGTTACATTATTCCAAGGCTTGTTATTGGAGTATTTATTCAAGTACTATG
TAGCTATAGTACCCTACCACTTTACGCAATTGTAACACAGATGGGAACTCACTTTAAGAAGGCGATATTTGATGAACAAG
TGCAAGCACGTCTTGTTGGTTGGGCACAAAAGGCAAAGAAGAAAGGTTTGAGAGGTGATAGTAATGCCCAATCTGCTCAA
GGAAGTGCTCAGGTGGGTGCTGGAATTCAACTGGGAGCAGTCTTCAGGAGGGCATCTGCCCCAGAAGAGAATGTTATTGT
CCCTACAAATGAAGGGCATCAATGA

>VrMLO13 JJMO01000320
ATGGCAGAAGTGCTGCAGCAAGGTCGCTCTCTGGCTGAGACACCAACCTATTCAGTTGCTTCTGTTGTTGCTGTCATGGT
TTTTGTCTGCTTGTTGGTTGAACGTTCTATCTACCGTTTTGGAGAGTGGTTGAGGAAAACAAGAAGGAAGGCACTTTTTG
CATCACTGGAGAAACTCAAGGAAGAGCTGATGTTGCTGGGGCTTATCTCTTTGCTGTTGGCACAAAGTGCAAGGTGGATC
TCTGAAATTTGTGTGAATTCGTCACTTTTTAGCAGTCGATTTTATATTTGTTCTGAACAAGACCTGGACATCAATGAGAA
TATCATGTTGGAAAGCTCATCATCTATACCTGGTGAAACTAGTATTCCAAAGGGATTAAATACTGGAGCATTCAACCAAT
GTGGAGAGGGTCATGAACCGTTTGTTTCATACGAGGGTCTTGAGCAGCTGCACCGGTTTTTGTTTGTTCTCGGAATCACT
CATGTTTTGTATAGCTGTCTGGCAGTTGGTTTGGCGATGAGCAAGATATATAGTTGGCGCCGATGGGAAAATCAGGCTAG
GATGCCAAGCGATGGAAACCAGCAAGCAAAGAAAATCAAGCTGATGAAGCGACAGACAACATTTATTTCCATCGCACTT
CTCATCCGTGGAGCAGGAGTCCAATCCTAAACTGGATGCTCTGTTTTCTACGTCAGTTTAGGAGCTCTATACGAAAATCA
GATTATCTTGCACTACGTTTAGGTTTCATCACTGAACACAAATTGCCGTTATCTTATAATTTCCATCAATATATGGTTCG
AAGCATGGAAGATGAATTTCAAGGCATTCTAGGAATAAGCTGGTTGCTTTGGATCTATGCCATAGTGTGCATCTTCGTGA
ACATCCACGGTCTGAATATATACTTCTGGCTATCATTTATCCCTGCAATTCTTGTCATGTTGATAGGGGCAAAGCTTCAA
CATGTTGTATCAACCTTAGCACTTGAAATTATGGAGCAGACAGGTCCATTTCCCAGGACCCAAGTAAAGCCACGCGATGA
CCTATTTTGGTTCAAAAAACCAGATATACTGTTATGGCTGATACAATTTGTCATATTTCAGAATGCATTCGAAATGGCAA
CGTTCATTTGGACATTGTGGGGATTCCAAGAAAAGTCATGCTTCATGAGAAATCATTACATGATCATCATTCGGTTGGCA
TCTGGTGCTATTGTTCAGTTCTGGTGTAGCTATATGACAGTGCCCCTGAATATAATAGTTTGTCAGATGGGATCTCGATG
CAAGAAGGCCTTAGTGGCGGAGAGTGTTAGAGAGTCCCTGCATAGTTGGTGTAAAAGAGTGAAACACAAGTCTAAGCATG
ATTCATTGCATTCACACACGGCAAGATCAGTATGCTCCCTCGAATCAACAATCGATGAGAGGGACGAAATAACAGTAGCA
TCTGGTACTCTGACTCGAAGCTCTTCTTTAGCCTCTTTGAATCAGATAACAGTGAGTTCAGTAGACCAGCTGAACTTCGA
GACTCCCAACAAAGCAAAAGATTCAATCAAGGTGGCTGGATGTTTGTCTGGATCTGTACATACGAATCCCCCACAACCAT
CCCACAACGTTGAAGATCGTGTCGGTAATGGAGAGGAAGCAAAAGCTATCACTCTCCTTGATTTGTTTCAGAAAACATGA

>VrMLO14 JJMO01000320
ATGGCAGAAGAACTTAACCACTCTCTGGAATATACACCAACATGGATTGTTGCGGTTGTTTGCTGCATAATTGTTTTCAT
CTCTCTTTGTGTTGAACGTGCCCTTCACAAACTCGGAAACTACCTAAAGAGAAAAGGTCAGAAAGCACTGTATGAAGTCT
TGACAAAATTGGAAGAAGAATTGATGCTTTTAGGGTTCATTTCCCTTCTATTAACCGTCTTTCAAGGTTTAATAAGCGAA
ATTTGCATCTCACCAAACCTTGCAACCCAAATGCTTCCATGCAAAAGGCCTCATAGGTCTTCAGAAGGTTCAGCGCATTA
TCAGATATACTATGATGCTATAATAAACAGAAGGAGGCTTTTGTCTAGTGGTTCTGGTTCAGATCATTGTACACATAAGG
GTAAGGTTCCACTGTTATCATTGGAATCTTTACATCAGCTTCACATTTTTATCTTTGTATTGGCCGTTGTACATGCCATA
TTCTGTGTCACAACAATGCTTCTTGCAGGTGCAAAGATGCGGGAATGGAAGGTATGGGAGGATTATTATCGAGATCACAG
TGAAGCAATAGATAAACATGAGTTCTTCAAAGAAAAAAGTCGAGGACATTGGAGAAAAGCAGCTGTTATTAGTTGGCTGA
TGTCATTCTTCAAGCAATTTCATGGCTCTGTTACAAAGTATGACTACTATGCACTTCGGTATGGATTTATCAAGAAACAC
CACTCAAATAACCTCAAGTATAATTTTCATAATTACATGCTGAGAACACTTGAAGTAGACTTCAAAAGAGTTGTTGGTAT
CAGCTGGTACCTGTGGGTCTTTGTTGTGCTGTTTTTGCTGTTGAACATTGAAGGATGGCACACTTATTTCTGGTTAGCCT
TTTTACCCTTGATACTTCTACTTCTGGTGGGGGCAAAGTTGGAGCACATAATCGCTCGTTTGTATCAGGATTCAGTAGAC
ATGTTGGGAAGAGATGATGACAAATTAGTGAAGCCATCAGATGAATATTTCTGGTTTACCAGCCCTCTCCTAGTCCTCCA
CTTACTTCACTTCATTTTGTTTCAAAACTCTTTTGAGATTGCATTTTTCTTCTGGATTTGGTGCACATATGGATTTGATT
CATGCATTATGGAGAAAGTAGCCTACGTTATCCCAAGACTTATAATGGGTGTAATTGTTCAAGTGCTTTGCAGTTATAGC
ACCTTGCCTTTGTACACTATTGTGACTCAGATGGGTAGCGAAATCAAGCCTCAGAAGTTGCTTTCAAGTGACTCCCTCAA
TACACCGCATTCCAACTGTATGATCAAAGAATCAACCCAAACTGATGAACAAGCAATAATCATGGTGGAAGATGCAACAT
CTGCAATCGAGCTTGTTCCCTTGGAGAAACCTAACACATCCAACAGTTAA

>VrMLO15 JJMO01000319
ATGGCAGCGAGTGAAAGTGGCAGCAGTTCCAGAGAGCTCGACCAGACACCAACGTGGGCCGTTGCTGTCGTGTGCACTGT
TTTCATTTTGATTTCCATGGCACTCGAAAAAAGTCTCCATAAAGTTGGGACGTGGCTTGGAAAAAAGCAAAAGAAGGCTT
TGCTCGAAGCCCTGGAGAAGGTCAAGGCTGAGTTGATGATTTTGGGTTTCATTTCACTGCTTTTGACTTTCGGGCAGAGT
TACATTGTCAAAATATGTATTCCTGAAAAGCTGGCAGACAACATGTTGCCATGTCCATATAAATATAAGGATGCTGAAAA
GGCATCAGACGATGGAGAGAAACATCGTAGAAAACTTTTGTCTTATGAACGTAGATATTTAGCTGCTGATACTACCTCGT
TTAAATGCAGCAATGAGGGACATGAGCCACTATTATCCGTGAATGGTTTGCACCAGTTACACATCCTCATATTCTTCTTA
GCAGTCTTTCATGTGCTTTACAGTGCTATCACAATGCTTCTTGGAAGACTGAAGATACGTGGATGGAAGGCATGGGAGGC
AGAGACTTCAACTAATAATTTTGAGTTCGCCAATGCTGCTTCAAGATTTAGGCTTACTCATGAAACATCATTCGTGAGAG
CTCATGCAGATTTATGGACAAGGATTCCCATTTTCTTCTATATTGGATGCTTCTTTAGGCAGTTTTATAGGTCTGTAACT
AAGACTGATTACCAGACTTTGCGGAACGGGTTTATCACTGTGCACCTGGCTCCTGGAAGTAAATTTAATTTCCAAAAGTA
TATCAAAAGATCATTTGAAGATGACTTCAAGGTGGTTGTGGGAGTCAGTCCTGTTCTCTGGGCATCAGTTGTAGTTTACC
TTCTCGTCAATGTTAATGGATGGCGTACCACACTTTGGGCAGCCGCAATTCCTGTTGTTATAATTTTGGCAGTTGGAACA

AAACTTCAAGCCATATTGGCAAAGATGGCGATTGAAATCACCGACAGACATGCAGTTGTCCAAGGAATACCTCTTGTCCA
AGGCTCAGACAAATACTTTTGGTTTGGTCAGCCACAGTTAGTCCTTCACCTTATCCATTTTGCTTTGTTCCAGAATGCGT
TCCAAGTAACATATATCTTGTGGATATGGTATTCATTTGGGGTGAGAAACTGTTTCCGTAATGATTACAAGCTTGCAATA
ATGAAAGTAGCTTTAGGGATTGTGATGCTATGCCTCTGCAGCTACATCACCCTCCCATTATATGCTCTTGTAACGCAGAT
GGGTTCAAGGATGAAAAAAGCGATCTTTGACGAGCAAACAAACAAGGCTCTAAGGAACTGGCACATGGCTGTGAAAAAGA
AGCAAAGGGTGGTGAAGCTAGGAAAGTTCAATGCAGAATCTGTTGATGGAAGCCCCGCTGCTTCATCTTCAGCCGTACAC
CCTTCTGGGCCTACACTTCACCGTTTCAAAACCACCGGCCATTCCACCCGCTCCCAGTCGTACGAGGATCAAGATCATGA
ATACGAGTCCGATGTTGAGTTGTCTCCTGAGTTGTCCCAAACAGCCAGCTTCATTGTGAGAATGGATCATGCTGATCACC
AACAACTACAAGAACATGAACATTCTGAGGAAGAAACCAACTCCAGTGAAGAAGCTGAATTCTCTTTCAAACCTAGTGAT
CTTGTGGAAAAACCACCACATAGCTTATAA

>VrMLO16 JJMO01000319
ATGGGGGAAGAAAATGGCGAAGGAAGATCCTTAGCTGACACCCCCACCTATGCCGTGGCAACTGTGATCACTGTCCTTGT
TTCTTTTAGTTTCTTGTTTCAGGGCACGTTAAAAAAACTGGTAAAGTGGTTGGACAGAACCAAGAGAAAATCTCTGCTTT
CTGCTCTGGAAAAGATCAAAGAAGAATTGATGCTTTTTGGGCTTCTGTCATTGCTGATGGGTCATTGGATTATTTTTGTG
GCAAAGATTTGCGTTAAGTCATCGGTAATAAGCAGCAGATTTTTTCCGTGCCCATTGGAAAAGAATTCTGTGAAACGTGT
GGTCTGGTTGAGTTCAGAATATTCAAATAGAACAGTTGTCAAAGGGCAAGTGAACAATGGCTTGCGAAATTATTGCCCTG
AGGGTCACGAATCATTGGCTTCTTATGAAAGTCTTGAGCAGCTTCATCGCTTTGTGTTTGTTCTTGGGGTCACCCATGTT
ACCTATAGCTTTCTTGCTGTTGCCCTGGCCATGATCAAGATATATAGCTGGAGAACATGGGAAAACGAAGCAAAGACTAT
TGCTGTACAAAGCATACAAGATACTTCACAAAGTACAACAAGCATAAGATTGAGGCGGCTTAATACATTCGTCTTTCACC
ATACATCCCATCCTTGGAGTCATCACAAAATTCTTGTTTGGCTGCTATGCTTCAGCCGCCAATTTTGGAGTTCTATTCAC
CGAGCTGATTACATGGCATTGCGCTTAGGCTTCATTACTAACCATGATCTTCCCACAACATACGATTTCCACAACTATAT
GCTTCGAAGCATGGATGAGGAATTTCGTGACATAGTTGGAGTTAGCGTACTTCTCTGGGTATATGCCATCTGCTGTATTT
TCCTAAATTTTCATGGTAGCAACTTTTACTTTTGGCTTTCCTTCGTTCCAGCAATATTGATCCTTATTATTGGTACTAAA
TTGCACCGAGTGGTAGTAAAGTTGGCTGTTGAAATAATAAATCGTTGCCCAAATATGAAACCTCACCAATTTAACTTAAG
AGATGAGCTCTTTTGGTTTGGAAAGCCCAGGTTCCTATTACGGTTAATTCAATTGATTTCCTTCTTGAATGCATTTGAAA
TGGCTACATTCCTATGGTCTTTGTGGGAAATTAAAGATCCTTCTTGCTTCATGAACAACCGAACCTTTATTGTGATACGC
TTGTCATTCGGGGTCATCTCCCAAGTCTGGTGTAGCTTTATAACGTTTCCTCTCTACGTTATCATCACACAGATGGGTTC
AAGGTTCAAGAAGACCGTGGTCTCTGAAAATGTGAGAAAATCACTCTCTAAATGGCAGAGAAGAGTGAAGGAGAGACAAA
GTTATTCTTCTGCACTCCTTACTGCAACATCAACAGCATCGATAAGTTCTTTTATCTGTGGAAACGAGCGTGGTTTGATG
ACTGAGGAAGGAAGCTCCTCAGGGACTAAAGATATTTGTCTCTCTTATCTTGATCATGTGTCAAGGACTCAAGCAGATGC
ATCATTTCCAAGCTATGAAAATGATCCTCATGATATAATCCATGATTCGTCTCCTCCATTGTCTTGA

>VrMLO17 JJMO01000831
ATGGCTGAAGAAAAAATTTATAAAAGATCACTGGAGGAAACACCAACCTGGGCTTTTGCAGTAGTTTGCTTTGTGCTGCT
TGCTATTTCAATCATCATTGAGCATGTTATTCATGCTATTGGAAAGTGGTTTAAAAAGAAACACAAAAGTGCTCTTTATG
AATCGTTGGAAAAGGTCAAAGGAGAGCTTATGATGCTAGGGTTCCTTTCCATGCTCCTAACTGTGTTCCAAGCTCCAGTT
TCTAAGATCTGCATATCAAAAAATGCTGCATCAACGTGGAATCCCTGCTCCAACCCAAAGGCCATCAGTAATTCGAATGC
AACATCTGCTACCATTGATAGGAAACTTCTCCAATATTTGGACCCCATGCCAAGACGTACTCTTGCTGCAAAAAGTTATG
ACAAATGTGCTGACCAGGGAAAAGTTGCTTTTGTTTCAGCATATGGGATTCACCAGCTCCATATATTCATCTTTGTGTTA
GCAATTTTTCATATCATACAGTGCATTGTAATACTAGCTTTGGGAAGAACTAAGATGAGACGCTGGAAGAAATGGGAAGA
CGAAACAAAGACAATTGAATACGAATTCTATAATGATCCTGAGAGATTCAGGCTTGCAAAGGACACAACATTTGGACAAA
GGCACTTGAATACTTGGAGTCAATCATCAATTTCCTTAAGGATAGTCAGCTTCTTCAGACAATTCTCTGGATCTGTTAAG
AAAGTTGACTATTTCGCATTACGGCATGGATTTATAATGGCACATCTGGCACCGGGAAGTGATACAAGATTTGACTTCCA
CAAGTATATCAAAAGATCACTGGACGAGGATTTTAAAGTTGTCGTAGGCATAAGTCCAATTATTTGGTTCTTTGTTGTTC
TCTTCCTGCTGACTAATACTCATGGGTGGTATTCTTACTATTGGCTTCCATTTGTCCCATTAGTTACCATTTTGATAGTG
GGTGCTAAGTTACAAATGATCATAACAAAGATGGGACTGATGATTGAAGACAAAGGGAAAGTAGTTAAGGGTGCACCTGT
GGTTGAGCCAGGAGATGACTTGTTCTGGTTCAATCGTCCAAGACTCCTTCTTTTCGTCATTCAATTTGTTCTCTTTCAGA
ATGCCTTTCAATTAGCACATTTTGCATGGAGTATATATGAATTCTCCATGAAATCTTGCTTCCACAAAACAACTGCAGAC
AGTATGATTAGACTCACAACGGGGGTTGTTATACAAGTTCTGTGTAGCTACGTGACTTTGCCTCTTTATGCTCTAGTCAC
ACAGATGGGTTCATCTTTGAAACCAACCATTTTCAATGATCGAGTAGCAGCAGCACTGAAGAAATGGCACCATACTTCCA
AAAAGCATGTCAAACATAGAAAGCACTATGAGGCTAAAAATTTCTCACCATTCTCCAGCAAATCATCAACCCCTACAGTT
GGCATGTCTCCCATTCATCTTCTGCATGGACACCCTGCTGGCAGAAGTGACAGTGCACGAACTTCTCCAAGAAACTCCAA
CTATGAAAATGAAGAATGTGATGTGCAAGGTGAATCTTCC

>VrMLO18 JJMO01001841
ATGGACAATAATGAACAGGAAACAAGGTCATTGGCCCTGACTCCTACATGGTCAGTTGCTACTGTGTTGACTGTCTTTGT
TGCTGTCTCTCTGCTTGTGGAGCGCTCCATTCACCGTTTAAGCAATTGGCTGCGGAAAACTAACCGAAAACCTTTGCTTG
CAGCTTTAGAGAAGATGAAGGAAGAATTGATGTTACTCGGGTTTATATCACTCTTATTAACAGCTACCTCACGCATGATA
GCAAATATTTGCATTCCATCAAAGTTCTACAACAGTGCTTTTGCTCCGTGCACTAGATCCCAGATTGATGAACAAATGGA
AGAGAATGGATCTGAAAAGCGTAAACTATGGATGGCTTCTGCTTATCATGTATCAGGGAGGATGTTGAACGGAATAAATA
GAAGCACTTGTAAAGAGGGTTATGAACCATTTGTTTCGTATGAAGGTCTAGAGCAGCTGCACAGGTTCATTTTTGTCATG
GCAGTGACTCATATATCTTACAGTTGCCTAACTATGTTGTTGGCCATTGTGAAGATTCACAGTTGGAGAGTGTGGGAAGA
TGAGGCTCACATGGATCGGCGTGATTCATTATCTGAAATTACAAGAGAGTTGACTATGAGAAGGCAATCAACCTTTGTCA

AGTCTCATTTATCAAATCCATTGTTCAAAAACAGTTCCCTTATTTGGGTGCTGCAGACATGTTTTTTCCGACAATTTGCA
CATTCTGTGGTTCGTGCTGATTATCTTACACTTCGCAAAGGCTTCATCATGAATCACCACCTTTCATTAAAGTATGATTT
CCACAGCTACATGGTTCGGTCTATGGAAGAGGAATTCCAAAGGATAGTTGGTGTGAGTGGCCCACTCTGGGGATTTGTTG
TTGGCTTCATGCTTTTCAACATTAAAGGATCTAACCTATATTTCTGGATAGCTATCATTCCTGTATCTCTTGTGCTTTTG
GTGGGAACAAAGCTACAGCATGTCATTGCAACCTTGGCATTGGAGAATGCTGGAATTGCAGGGTTCTTCCCAGAAGCAAA
GTTGAGGCCTCGTGATGAACTCTTTTGGTTTAATAAGCCTGAGCTATTATTGTCCTTGATCCATTTCATTCTCTTCCAGA
ATGCATTTGAGCTGGCTTCATTCTTTTGGTTCTGGTGGCAGTTCGGATATGATTCTTGCTTTATTAGGAATCACCTTCTC
CTCTATTTAAGGCTAATATTGGGCTTTGCAGGACAGTTTCTTTGTAGCTACAGCACCTTGCCACTATATGCACTTGTTAC
ACAGATGGGAACAAACTACAAGGCAGCATTAATCCCACAGAGGATAAGAGAAACAATTCATGGATGGGGTAAGGCAGCTA
GGAGGAAGAGAAAACATGGTATGTTCCCTGATGATTCCACCATTCATACAGACACAAGCACTGTGTTGTCTATTGAAGAA
GATGATCAATTAATTGATGCCCCTGAAGTAGAATTACAACCAGTCAATGCTGTTACATCTACCCCTTCTCCCATTGCCAA
TGAAACTTCAAGTAGGGTTGCTACACCCCTCCTAAGACCCTCTGCTTCAATATCTTCAGCTCATAGCAGTTCTAAAGCTG
AAGTAATTTTAAGATGCTCCTCTATGCCAAGTGGGAGATAA

Supplementary Figure S11. List of legume MLO deduced protein sequences.

>ArMLO1 ARADU.27DYI
MHASKCKMQFFMISIACSLLWNGVMASSDSSGTGSELDQTPTWAVAGVCTVFILISIVLEKGLHKVGTWLNDRHKRALLE
ALEKVKAELMVLGFISLLLTFGQSYIVRICIPGSAADKLLPCKYAGSGDESSGKDQIHRKLLSEDGVYKCKEGYAPLISS
NGLHQLHILIFFLAVLHVLYSAVTMLLGRLKIRGWKAWEAETSSHGYEFATDASRFRLTHETSFVKAHATFWTRNPVFFY
IGCFFRQFYRSVGKADYLALRNGFISVHMAPGSKFDFQKYIKRSLEDDFKIVVGVSPVLWASFVIFLLLNINGWHAMFWA
SLIPVVIILAVGTKLQATLANMAIEITEKHAVVQGIPLVQGSDQYFWFGRPQLVLHLIHFALFQNAFQITYFLWIWYTFG
FRNCFHADFKLAILKVVLGLGVLCLCSYITLPLYALVTQMGSTMKRSIFDEQTSKALKKWHMAVKKKHGERLGKSKARTL
DDGSTIGSTVHSSGPMLHRFKTTGHSTRHTSSYEDHDDDEDYQSETELSPITPRSSLIVRVDNGDQQEEIARENRHPTVG
EQTNNGFGG

>ArMLO2 ARAIP.Y3LT4
MSGGGVEEGNDLEFTPTWVVAVVCSVIVAVSLLAERLLHYAGKFLKRKNQKPLFEALLKVKEELMLMGFISLLLTVTQNG
IIRICVPEHWTHHMLPCKFEDKEEETKTTSHFQNFFSFSGNLRHLLEASKSAETGSQTGYCARKGKVPLLSIEALHHLHI
FIFVLAIVHVTFCVLTVVFGGLRIRQWKNWEDAIAKENYNSERVLEPRPMVTEVHQHEFIRDRFTGFGKDSAILGWMKSF
FKQFFGSVTKLDYVTLRLGFIMTHCRGNPKFNFHKYMIRALEDDFKKVVGISWYLWIFVVIFLLININGWHTYFWIAFIP
VIVLLAVGTKLEHVITQLAHEVAEKHAAIEGELVVQPSDDHFWFHRPRIVLFLIHFILFQNAFEIAFFFWIWVTYGFDSC
IMGQVRYIVPRLIIGVFIQLLCSYSTLPLYALVTQMGTHFKKAIFDEQLQVRLVGWAQKAKKKGPRPESSHNGSSTAANN
NGVQLGSIFRRASASPPEDHANSIVPI

>ArMLO3 ARADU.65SUF
MAAGEQEEQRSLQETPTWAVSICCFFFILLSLSIEVGLHKLSAILKKNNRRSLGKALAKIKTEMIKYGFISFLLTISQQI
PLSKICVREAVANSFLPCSDPPLMRSLTEEVASWDFGEEKGRSLNATANEVNYCESKGMVSLISDDGIQQLNIFIVFLAV
FHISSCTLTMCLGMAKMRRWKKWENETQTLEYQIAHDSSRFQYTRQTSVGKRHLNFWSNYSPLLWMVCFLRQFYRSVSKD
DYFTLRNGFIAANISVGGNFNFMKFLSRTYDQDFEKIVRIRSWIWIFSILFIFFSAHEFYNHYWLPFIPLVVAIIGGTKL
QVVITNMCVDSVKEKAVIKGTILVKPNDDYFWFGRPEWLLYLIQFILLQTSFQLAFFAWTWYEFGPRSCFNREVESIAIH
VIMGVTVQILCGYVTLPLYALVTQMGSGMRSAVFTERVTEGLKSWHKRAKQSVSNNNSTSSKYLASLHSRKSESSIKENL
SNRGDSRESNENTMHPLPLPLASGSGEEEEESTTGETQEEEISTHSISEIKTVEEEENENNNDNNNQIIVRGGTFIGEAS
FGSSWKNMDSPRTIISKIGSVTEEDDNISDIYLT

>ArMLO4 ARADU.N8G7F
MAEEELNESLEYTPTWIVAVVCTIIVFISLVVERGLHKLGKYFKKKNQTPLFDALQKLQEELMLLGFISLLLTVFQTAIS
HICISPELTTTMLPCKRPHESSEGSAHDQIYYDGIINKRRLFSVEDTSQHCRSKGKVPLLSQESLHHLHIFIFVLALVHA
IFCVTTLFLGITRMRQWKRWEEEIKSKITRTGDSSSIQALHHHEFFKKHADGYWRRAAVVGWLISFFKQFYGSVTESDYI
ALRYGFIKEHCPRKPQFDFHNYMVRTLEVDFRRIVGISWYLWLFVVLFLLLNIAGWHTYFWLAFLPVIILLLVGAKLEHI
IARLAQEPAQESARVKPSDEYFWFSRPTLVLDLLHFTLFQNSFEIAFFFWIWCTYGFDSCIMEKISYIIPRLIMGVIVQV
LCSYSTLPLYTLVTQMGSGFKKGMLDPRVEEALLNWAEERHENSGTSRWRSISSHRMTKEQTQNDEHEIAMEMEDATTSS
IELPSSIVQIPLDRPISYSLRHAQ

>ArMLO5 ARADU.U7W18
MAEVLRRGRSLAETPTYSVASVITVMVFVCFVVERSIYRFGKWLKKTRRKALFASLEKIKEELMLLGLISLLLAQSARWI
SEICVNSSLFNSEFYICSEKDLDANQNVMPDGSSFSPDETDIPKGLISGSFNQCPAGHEPFVSYEGLEQLHRFLFVLGIT
HVLYSCLAVGLAMSKIYSWRRWENQTTAAADGNLQGKRNKVMRRQITFVFHHASHPWSRNPILIWMLCFLRQFRSSIKKS
DYLALRLGFITEHRLPLSYNFHQYMVRSMEDEFHGILGISWPLWIYAIICIFVNIHGLNIYFWLSFIPAMLVMLIGTKLQ
HVVSTLALEIMEQQGPLARTQVKPRDDLFWFKKPEILLWLIQFVIFQNAFEMATFIWSLWGLEQKSCFMKNHYMIIIRLA
SGVLVQFWCSYMTVPLNVIVSQMGSRCKKALVTESVRDSLHSWCKRVKQKSKHDHSHHTARSVCSLESTIDERDEITVVS

GTLTRSSSLDLESLNQITVTSVDQLKFEASNSSRDQDSMKVAEYLSKSVHIDSTQPPDNGEDHVGGSEDVAKSETLSDLF
QKT

>ArMLO6 ARAIP.HRR0E
MMEPIGEERSLSETPTWAVATVVTILVSLSFLFHGTLKKFLKWMARTKRKSLLSALEKIQEELMLFGLLSLIMGHWIIFV
AKICVKSSALSSRFFPCAVKDISGTAVQHNIFWHTSHYNNSDSKEKLHIGLRNYCPEGHESLASYESLEQLHRFMFVLAI
THVTYSFVAVALAMIKIYSWRTWENEAKTMALQQSLQGTPQTTSSIRLNRLTTFVFHQTSHPWSNHKVLVWLLCFSRQFW
SSINCADYMALRLGFITNHELPLTYDFHNYMLRSMDDEFRDIVGVSVLLWIYAICCIFLNFHGSNFYFWLSFVPAILILI
IGTKLHRVVVKLATEIVDRCPYVKPHQFNLRDELFWFGKPRFLLRLIHLISFLNAFEMASFLWSLWEIKEPSCFMKNKTF
IAIRLSFGVVSQVWCSFITFPLYVIITQMGSRFKKSVVSENVRESLSKWQRRVKHKQSSPQALVSETSPSSDSGINNAMD
KLISGEGSSSGTKHDYCYSHHHFDYVSSRTQRNEPFLSHDDDDDDDSYPLPLS

>ArMLO7 ARADU.0YR51
MAGGEGGAGERSLQETPTWAVAVVCSVFVILSVLIEHGIHSLGKWFQKKQKKAMNEALEKIKSELMLLGFLSLLLTFGTN
YIKKICIPHSIGDTMLPCKKVVSHGGDGRRHLLSFETEDDNDVLSWRRILATSISGDDYCSTKGKVPLISASGLHQLHIF
IFVLAVSHIFYSVMTMVLSQAKMKKWKSWEAETSSLEYQFSNDPARFRLAHQTSFVKQHSGWSRMPGIRWIVAFFRQFFA
SVTKVDYMTMRHGFINAHLGPNSKFNFHKYIKRSMEDDFKVVVGISMPLWVSAIIFLLLNVYKWNTLTWLSFIPLVILLL
VGTKLELIIMEMAQEIQDRTTIVRGVPVVEPNNKYFWFNRPQWILFLIHFTLFQNAFQIALFLWTVYEFKIKSCFHENLK
LILIRVILGVFLQFICSYITFPLYALITQMGSHMKKAIFEEQTAKAIKKWQKTAKDRTKLRKAGMDNVSNSGYMSGETTP
SQGTSPIHLLHKYKPSSDHTDTESVLYSPRSYPSDTELSETELAYTHHHRIQLNEITTSHGHGPPNNRQETHNVDFSFDK
P

>ArMLO8 ARAIP.L2HMX
MAVRGEERALDHTPTWAVAAVCASIVLISIVLEKIIHKFEKMFERKKKMALLQALAKIKGELMVLGFISLILTFGQTYIS
KWCIPEKYANTMLPCVRSTKEEGEGGGGGHNQATPAGAGVVEEKGGAKEAAAEGEPPHRRLLTYERRFLAAASEGCKPGM
EPLISVHGMHQLHIFIFFLAVFHVVYSAITMTLGRAKIRGWKAWEQDHIVDQEALNDPRRFRLTHETSFVRDHNSIWTKT
PVSFYFVCFFRQFFRSVRKADYLTMRHGFVTVHLAPGSKFDFQKYIKRSLEDDFKVVVGISPILWASVVFFLLVNVHGWH
ASFGVSFLPLMVILAVGTKLQAIITRMALDIKERHAVVQGIPLVQVSDKYFWFEWPELVLYLIHFVLFQNAFELTYFWWT
WYEFGWASCFYEDEILMISRVALGIGAQIITSYVTLPLYALVTQMGSTMKKSIFDEQTSKALKKWHQNALKKIASRGRHA
TLGGTTMSKSPDGSPSKMSTRSPNAGQSATILSSVDNTIPAYDNRDLLTGP

>ArMLO9 ARAIP.CFV17
MNVESSIMGGGRSLEETPTWAVALVCFVLLLISIFMDHIFHLIAKWLRKKRKKALYESLAKIKSELMLMGFISLLLTVLQ
GPISRICVPEKLAATWHPCNHQQEPEDHHVFLLAPGSSTHQCAAHQGKVALVSAQSIHQLHIFIFVLALFHVLYCIFTLA
LGTAKMRRWKQWEEETKKPEYQFSHDPEKFRLTRQTSFGRRHLSVWTTNPILVWIVCFFRQFVCSVPKVDYLTLRHGFVM
AHLAPQSQQKFDFMQYIERTLEEDFKVVVEISPPIWFITVLFLLFHTHGWYSYLWMPFVPLIIIVVVGSKLQVIITKMGL
RIGERGDIVKGTPVVQPGDNLFWFKKPQLMLYLINFVLFQNAFQLAFFSWTALQFGMKSCFQSKTEDVVIKISMGILVQI
LCSYVTLPLYALVTQMGSNMKPTIFNERVASALRNWHQSAKRNIKKKNRGGSSFSQTTTPSPNHSYDADSLSSSNGARAH
SCFSSSSSFSSSSHHHEIEMAHLAHHQHQNLKEPNSVHVASDLPLPQHEIQIETSK

>ArMLO10 ARADU.WJ2TG
MSAGGAEEEANLEFTPTWVVAAVCTVIVAISLAAERFLHYGGKFLKNKNQKPLYEALQKIKEELMLLGFISLLLTVSQNG
ITKICVPPSVTRYMLPCSLDEKEKVTVEHLLGQDRFHFERFFSFPRRLLAETRGTETETHALAKTEFCARKNKVPLLSVE
ALHHLHIFIFVLAVVHVTFSVLTVIFGGARIRQWKHWEDSIAKQNYETDQVLKRKVVDVQQHDFIKGRFSGFGKDSAIVG
WLKSFFKQFYGSVTKSDYVTLRHGFIMTHCKANPKFNFHKYMIRALEDDFKQVVGISWYLWVFVVIFLLLNINGWHTYFW
IAFIPFILLLAVGTKLEHVITQLAHEVAEKHAAIEGELVVQPSDDHFWFHRPHFVLFLIHFILFQNAFEIAFFFWIWVTY
GFDSCIMGQVRYIVPRLIIGVFIQVLCSYSTLPLYAIVTQMGTHFKAAIFREHVQVRLAGWAQEAKKKKGVKADSQPGEG
SSHAVAGIQLGSVFQKRASAAEDGASVPKVEGSAE

>ArMLO11 ARAIP.SR8Z2
MSDSVQEARSLALTPTWSVATVLTIFVAVSLLVERSIHRLSNWLRKTNRKPLLAALEKMKEELMLLGFISLLLTATSRII
ANICLPSKFYNSAFAPCTRSEIDEEMEDNGSEGRKLLMAFAYPSLTRRMLNGMNRSSCKEAGYEPFVSYEGLEQLHRFIF
VMAITHISYSCLTMLLAIVKIHSWRVWEDEAHMDQHGSLAVITRELTMRRQSSFVKVHTSNPMARNSFLVWVTCFFRQFG
HSVVRADYLTLRKGFILNHNLTLKYDFHSYMVRSMEEEFQRIVGVSGLLWGFVVAFMLFNIKGSNLYFWIAIIPLVLLVG
TKLQHVIATLALENAGITGYFSGSKLRPRDELFWFNKPELLLSLIHFILFQNAFELASFFWFWWQFGYNSCFIRNHMLVY
LRLILGFAGQFLCSYSTLPLYALVTQMGTNYKAALIPQRIRETIHGWGKAARRKRRHGMFADDSTIHTDTSTVLSLEEDD
RMVDIPETGTDHVTGTEVELQPVAAATATPSPPAANEASSRAATPLLRPSASISSSGPFSLRAESIPRCSSMPMPSGR

>ArMLO12 ARAIP.WMV74
MSIRGVDEEQDIELEFTPTWVVAVVCSVIVGVSFAVERFLHYAGKRLKKKNQTTLYEALLKIKEELMVLGFISLFLSVSQ
RGITKMCVPKSWSHHMLPCSLKEKEELEAETNITSSHFQNSLSFSSNNLRHLLALAHAQVAAEAQLQYCALKNKVPLLSN
EAVHHLHIFIFVLAIAHVTFCVLTVAIGLLRIRQWKQWEDSVATQNNETHRDLESTVTLVHQHAFIRDHFIGIGKNSTLL
GWVKSFFKQFYGSVTKLDYVTLRLGFIMTHCRRNPKFNFHKYMIRAVEDDFKKVVGISWYLWIFVVIFMLININGWHTYF
WIAFIPVILLLAVGTKLEHVIMELANEVAERHVAIEGELVVQPSDHHFWFNRPRIILFLIHFTLFQNAFEISYFFWILVT

YGIHSCIMGPIGYIIPRLIIGLFIQLLCTYSTLPLYAIVTQMGTHFKKNIFEEQLERPIVYYGGEKAKNNGAKADESHGE
HERRASSPEANTNVPKEDKSN

>ArMLO13 ARADU.SSV2N_48N80
MAGGEKERTLEETPTWAVAIVCFVLLAISIIIEHIIHAIGKWLKRKNKTALYEALEKVKGELILLGFISLLLTVFQNPIS
RICISKNVASTWHPCAVPKKSKSGYEDSDDDDGDDGNRRKLLEYLGHIPRRVLATKGYDKCAEKARCLKNYKIFHILQCI
ITLALGRTKMRRWKKWEDETKTIEYQFYNDPERFRFARDTTFGRRHLSAWSRTSVSLWIVSFFRQFYGSVTKADYVALRH
GFIMAHLAPGSEAKFDFQKYIHRSLEEDFKIVVGISPIIWIFAVLFLLTHTHGWYSYYWLPFIPLTIILLVGAKLQTIIT
KMGLRIQERGEVVQGSPLVEPGDELFWFNRPGLLLSLVHLVLFQNAFQLAFLAWSTYEFSIHSCFHKTTVDVVLRISIGV
VIQVLCSYVTLPLYALVTQMGSTMKTTVFNDRVATALKNWHHSAKKHVKDSKHGEGNNSVTPFSSRPTTPTHGLSPVHLL
YRHTVGHSDSVQTSPRISNYENEQWHLQGSPSTTSNHDTDERQVVEQPSTLESPVIGAQHEINISLSEFSFANRQNSRD

>ArMLO14 ARAIP.MQE1N
MSDSVQEARSLALTPTWSVPTVLTIFVAVSLLVERSIHQLSNLRKTNRKPLLAALEKMKEELMLLGFISLLLTATSRIIA
NICLPSKFYNSAFAPCTRSEIDEMEDNGSEGRKLLMAFAYPSLTRRMLNGMNRSSCKEAGYEPFVSYEGLEQLHRFIFVM
AITHISYSCLTMLLAIVKIHSWRVWEDEAHMDQHGSLAVITRELTMRRQSSFVKVHTSNPMARNSFLVWVTCFFQQFGHS
VVRADYLTLHNRLSWNHNLTLKYDFHSYMVRSMEEEFQRIVGVSGLLWGFVVAFMLSNIKGSNLFLDSYHPCHLVLLVGT
KLQHVIATLALENAGITGYFSGSKLRPRNELFWFNKPELLLSLIHFILFQNAFKLASFFWFWWQFGYNSCFIRNHLLVYL
RLILGFAGQFLCSYSTLPLYALLTQMGTNYKAALIPERIRETIHGWGKAARRKRRHGMFADDSTIHTDTSTVLSLKEDDR
MVDIPETGTDHVTGTEVELQPVAAATASSEAI

>CaMLO1 Ca_04814/LOC101504273
MAGAALGERTLKETPTWAVAAVCAVFIIISVLIEYGIHSLEKWFHKKHKKAMSEALEKIKAELMLLGFISLLLTFGTKYI
AKICIPVKLGDSMLPCKKERVEEDSKDDRRRLLSFDDDNLVWRRSLAAAAGGDDYCSNKNEVPLISQSGVHQLHIFIFVL
AVFHIFYSVMTMVLARAKMQKWKAWEMETSSVEYQFTHDPARFRFAHQTTFVKRHSGWSRKPGIRWIVAFFRQFFASVSK
VDYMTMRHGFINAHFAPDSKFDFHKYIKRSMEDDFKVVVGISIPLWAFAIIFLLMNVYSWYTLTWLSLAPLVILLLVGTK
LELIIMEMAQEIQDRTTIVRGVPVVEPNNKYFWFNRPHWIIFLIHFTLFQNAFQIAYFLWTWYEFTITSCFHENLPLIVT
RVVLGLALQVLCSYITFPLYALVTQMGSHMKRGIFEEQTTKALKKWQKSARDKRKLRKAGGGGSIDVPSVSGETTPSQGT
SPMHLLHKFKPSSHTDTDSVLYSPRSYQSDQTDFSDTEGSTHRLNLDQIMSQPQHNPPGNQLTHNIDFSFDKP

>CaMLO2 Ca_23649/LOC10500098
MAAQVYERTLEETPTWAVAVVCFVLLAISILIEHIIHVIGKWLKKKHKNGLYEALEKVKAELMLLGFISLLLTVFQDPIS
KICISQKVGNTWHPCSNPNKKSKSNETSENENNDRRKLLEYFDPIPRRILATKGYDKCVDKGKVALVSAYGIHELHVFIF
VLAIFHILQCIITLALGRIKMRRWKKWEDETRSVEYQFYNDPERFRFARDTTFGRRHLSMWSQSTISLWIVSFFRQFFGS
INKVDYLALRHGFVMAHLAPGNDAQFDFQKYISRSLEKDFKVVVGISPTIWFFAVLFLLTNTHGWYSHYWLPFIPLIIIL
LVGVKLQMIITKMGLRIQDSGEIIKGAPLVEPGDHLFWFNRPDLLLFLIHLVLFQNAFQLAFFSWSTYEFSVNSCFHKTT
AEIIIRLTLGVVIQILCSYSTLPLYALVTQMGSTMKPTIFHERVATALKSWHNTAKKHVKESKHSNNTTPFSSRPSTPTH
GMSPVHLLHRHTVSSSDSLQTSNYENQQWDNVEGSNSPTINHTEAHEIRIPTELPVSNRHEISISSKDFSFEKRYIVSD

>CaMLO3 Ca_04697/LOC101490933
MSGGEEEEETTLEFTPTWVVAGVCTVIVAISLVAERLLHYGGKFLKRKDQKPLYEALQKIKEELMLLGFISLLLTVSQNS
LTKICVPPSVLRHMLPCTLKEKEKANQVSHYSAFSFPGLARRILANEEDEHPKTPFCARKNKVPLLSLEALHHLHIFIFV
LAVVHVTFSVLTVVFGSARIRQWKHWEDSIAKQNYDTHQVLKPKLTHVGQHEFIKGRFSGFGKDSTLIGWLQSFFKQFYG
SVTKSDYVTLRLGFIMTHCKTNQKFNFHKYMIRALEDDFRQVVGISWYLWLFVVIFLLLNINGWHTYFWIAFVPLILLLA
VGTKLGHVITQLAHEVAEKHAAIEGDLVVQPSDEHFWFHRPRVVLFLIHFILFQNAFEIAFFFWIWVTYGFDSCIMGHIR
YIVPRLVIGVFIQVLCSYSTLPLYALVTQMGTHFKRVIFNEHVQDGLVGWAQKVKKKKVVKADGQGSSHEGSSTGVQLGS
VFQKRASAHETNNGQGSSHEGSSTGIQFGSAFQKRASASEDNTPPITKTDGSN

>CaMLO4 Ca_01959/LOC101505951
MEKENGNGRSLAETPTWAVATVVALLVSLSFLSHGTLKKLFKWLDKTKRKSLLSAMEKMQEELMLFGLLSLLMGHWILFV
AKICVKSSVLSSRFFPCAMEKSFGIVKQVAWSNSEYSNQTILKEQAHHGRHNYCPKGHESFASYESLEQLHRFVFVLGIT
HVSYSFVSVALAMIKIYSWRRWENESKTMAVQSLQDTSQTTSTSSIRLRRLSTFIFHHTSHPWSHHKVLVWLICFSRQFW
SSINRADYMALRLGFITNHDLPLTYDFHNYMLRSMDEEFCDIVGVSVVLWIYAICCIFLNFHRSNFYFWLSFVPAILILI
IGTKLHRVVVKLAVEVRDHCPYMKPHQFNLRDELFWFGKPWILLRLIQLISFLNAFEMSTFLWSLWEINDPSCFMNNRTY
VAIRLSFGVVSQIWCSFITFPLYVIITQMGSRLKKSVISENVRKSLSKWHRRVKAKRSSSYALLTKTSPTSLDFLLYGID
NSNSNKSAFISETEEGSSSGTKDNSLSNLDDVSMTQKL

>CaMLO5 Ca_04811/LOC101503406
MSGGGGGGDERQLDVTPTWAVAAVCAVIVIISISLEKLIHKIAKVFEEKKKHALLEALEKIKAELMVLGFISLLLTFGQN
YISKVCIPTRYSNTMLPCLPLDQRQAGHPSEPAQTGHEPPTTGHEPPTTGHEPPTTEHEPPTTEHEPPTTEHEPAPTEHE
PPPTAHEAPAEEAEPSKGEHHRRLLSYERRVLSGGGGGHGCKLGYVPLIAVNGLHQLHIFIFFLAVFHVIYSAITMTLGR
AKIKGWKEWEQDHVVDEDALNDPRRFRLTHETSFVRDHNSFWTKTPVSFYFVCFFRQFFRSVRRADYLTMRHGFVSVHLA
PGSKFDFQKYIKRSLEDDFKVVVGISPLLWASVVLFLLLNVHGWHAAFWVSFLPLVVILAVGTKLQAIITRMALDIQERH
AVVQGIPLVQVSDKNFWFEWPQLVLYLLHYVLFQNAFELTFFWWTWYEFGWASCFYEDDSFMIVRVALGLGAQFVCSYIT

LPLYALVTQMGSTMKRSIFDEQTSKALKNWRNKAMHKKPSIKRPTETRTLGSPMDDGSPLDSPLTNINTSAETELQEHGT
ATILTSVDNDHYDNRDLLTGP

>CaMLO6 Ca_02517/LOC101492588
MAGGRSLTETPTWAVAVVCFVILSISIIIEHIFHIIGKWLKNKHKSALYESLEKIKSELMLLGFISLLLTIGEGLISKIC
ISEKVAATWHPCTNNAERNSDDHQPIEHETGGRRLLAALLASQNKNSRRILAAGGIDKCAAQGKVAFVSSEGIHQLHIFI
FVLAVFHVLYCILTLALGRAKMRRWKRWEEETKTLEYQFSHDPERFRFASETSFGRRHLSFWTKNPVLIWIVCFFRQFVR
SVPEVDYLTLRHGFMMAHLAPQSHQKFDFRHYIKRCLEEDFKVVVEISPPIWFITVLFLLFHTHGWHSYLWLPFVPLIIV
LLVGTKLQVIITQMGLRIQKQGMVVKGEPVVQPGDDLFWFSKPRLILHLINFVLFQNAFQLAFFSWTALQFGLTSCFNSR
TEDVVIRISMGVFVQILCSYVTLPLYALVTQMGSTMKPAIFNERVATALRNWHNTAKKHIKQNRGSGFQSPMSTRSITPA
HSMSPAHILRPYHSETDTHPTTSPTRLNFETYNPYEAYSPSPSNKVEARATSSSSIYFHEMEMGHLAHDHEQETNKPNCV
SVGSGLTQLEIDVQHSDEFSFSKMATNNLK

>CaMLO7 Ca_11758/LOC101501959
MSGGGEGGEEGPDLQFTPTWVVALVCTVIVAVSFAVERSLHYVGKFLKKKNQKALFQALQKIKEELMLLGFISLFLTVTQ
NRITKICVPENWTHHMLPCSLDEKDEESKKSTSHFQTFFSSNDVFGTTRRLLGDDHPSTEETLGYCAAKGKVPLLSVEAL
HHLHIFIFVLAVVHVTSCVLTIVFGGLNIRRWKHWEDSITDENHETQHAPDRTVTHVHQHAFIKDHFTGFGKDSAIMGWV
RSFFKQFYGSVTKLDYVTLRLGFIMTHCKANPKFNFHKYMNRALEDDFKKVVGISWYLWVYVVLFLLLNINGWHTYFWIA
FIPLILLLAVGTKLEHVIIQLAHEVAEKHTAIQGELVVQPSDDHFWFHRPRIVLFLIHLILFQNSFEIAFFFWILVTYGF
DSCIMGRIRYIIPRLIIGVFIQLLCSYSTLPLYALVTQMGTHFKSVIFDEQVQARLVGWAQKAKKKGLRGDNNQSNQESA
HHGSSVNIQLGSIFRRGSVPENNTIVSRDEESG

>CaMLO8 Ca_00046/LOC101508058
MEDVMHRGRSLAETPTYSVASVVTVMVFVCFLVQRSIYRFGKWLKKTRRKALFASLEKIKEELMLLGLISLLLAQSARWI
SEICVNSSLFSSRFYTCAEQHLGTIENIMIENSSSLPDETNIPKGLSSVALHQCGEGHEPFVSYEGLEQLHRFLFVLGIT
HVLYSCLAVALAMSKIYSWCRWENQAAMATDANLQGKKNKVMRRQITFVFHRTSHPWSRSPILIWMLCFLRQFRSSIQKS
DYLALRLGFITEHKLPLSYNFHKYMVRSMEDEFHAILGISWPLWIYTIVCIFVNIHGLNIYFWISFIPAILVMIIGTKLQ
HVVSTLALEIREQQGPSARTQVKPRDGLFWFKKPDILLWLIQFVIFQNAFEMASFIWTLWGFKEKSCFMRHHYMIIIRLT
SGVFVHFWSSYMTVPLNVIVSQMGSRCKKALVTESVRESLHSWCKRVKQKSKNESMHSHTARSVCSLESTIDERDEITVV
SGTLSRTTSLDLESLNQITVTSVDQLNFVTSNNLHDSTNLSESVHINSPYNDGDHVGNAEEAKIESTLFELFQKT

>CaMLO9 Ca_11277/LOC101495573
MLATEQGRNIISNGLIEIKKNMAVKGEQRSLEDTPTWAVSVCSFFVLIVSLIIEAGLHKLTEHLKKKKRISMGKALAKTK
TEMMKFGFVSLLLTISQVPISKICINKDVANSFLPCNDSMESEYQFINYCEAKGKVSLLSSDGILQLNIFISFLAVFHIL
FCTFTMCLGKAKMRRWKKWEDETQTLEYQIDNDSRRFQYISQTLGGKRHLKLWNNYSPLLWMVCFMRQFYNSVSKDDYFA
LRNGFIVANISMGSNFNFNFKKFLSRTFDEDFEKVVGIRFWIWFFSILLIFLSAHEFYNYYWLPFIPLVVALLAGTKLQM
IITNMHLDSCKQSPVIKGTILVRPSDEYFWFHRPEWLLHIIKLILIQNSFQLAFFTWTWYEFGPRSCFNREKADIAIRIA
LGLAVQLLCGYVTLPLYTLVTQMGSNMRKEIFTERVVEGLKSWQKKAKRSLSKNNSTCSRHTVSASLHSKASENSVKESS
ESMHNKVDDNENMQSITVVTGSGEEEEINIATNSSVEQEMIIPTEEHNAKIITRGIYDGEISFGSSWKNVGSSRSIREIV
SIAEEEEENEDEDVTVTNYHI

>CaMLO10 Ca_10710/LOC101494654
MAATESDSYNSSRKLDETPTWAVASVCTVFILISIALEKSLHKLGMWLGRRQKKALLEALEKIKAELMILGFISLLLTFG
QNYIVRICILEEVANKMLPCPHKYSNISDDKKSSSEVVNVRKLVSYEGRFLAADTPSFKCSRKGHEPLLSINGLHQLHIF
IFFLAVFHVLYSAITMLLGRLKIRGWKEWEEETSTHGNQFANDAAKFRLTHETSFVRAHTSFWTRIAISFYIGCFFRQFY
RSVRRVDYQTLRNGFISVHLAPGSKFNFQKYIKRSLEDDFKVVVGVSPVLWASAVVFLLLNIDGWRTPLWAFLIPIIIIL
AVGTKLQVILARMALEITETHAIIQGMPLVQGSDKYFWLDQPQLVLHLIHFALFQNAFQITYILWTWYTFGLENCFRLGY
KLAVIKVACGVVMLCLCSYITLPLYALVTQMGSRMKRSIFDEQTSKALMKWHMTVKKKHVGEVVKLGKSTIDGSNIGSTL
QSPGPALHRFKTTGHSTRSSTFEDQDEYESDNELEKTKLILRVDQLAEEDHRHHSEGEANNEDEFTFLKHTTIQS

>CaMLO11 Ca_06880/LOC101511064
MAAENEQETRSLALTPTWSVATVLAIFVAVSLLVERSINRLSIWLRKTNRKPLLEALEKMKEELMLLGFISLLLTATSGM
IANICIPSKFYNSAFDPCSQSEIDKEIEENGSEGRKLLMAASYPRIIRRVLNGINRNSCKVAGYEPFVSYEGLEQLHRFI
FVMAITHVSYSCLTMLLAIVKIHSWRVWEEEAHMDRRNYLTEITRQMTMRRQSTFVKSHTSNPLVIWMTCFFRQFGRSVV
RADYLTLRKGFIMNHNLSSKYDFHSYMVRSMEEEFQRIVGVSGLLWGFVVCFMLFNIKGSNLYFWIAIIPISLVLLVGTK
LQQVIATLALENAGITGFTSGAKLRPRDELFWFKKPELLLSLIHFILFQNAFELASFFWFWWQFGYNSCFIRNHRLVYLR
LILGFAGQFLCSYSTLPLYALVTQMGTNYKAALIPQRIRETIHGWGKLARRKRRHGMFIDDSTIHTDTSTVLSIEEEDQI
IDDSFKDLVTGTGVELQPVKTVTSISSPIANETSSRAVTPLLRPSASLSSSVPFSSRHEPVIRSSSMPSGR

>CaMLO12 Ca_09883/LOC101496809
MCISMYKKRLSFCVVSFSWVLLNGGVAMASSESSDSSRDLDQTPTWAVACVCTVFILISITLEKSIHKVGTWLGERHKKA
LLEALEKVKAELMILGFISLLLTFGQSYIVRICIPANMADKLLPCPYRHTGSKNEEEHHRKLLHRYLSEDATPYQCKEGH
KPLISVNGLHQLHILIFFLAVLHVLYSAVTMLLGRLKIRGWKAWEAETSSHGYDFANDPSRFRLTHETSFVRQHATFWTK
IPIFFYISCFFRQFYRSVDKADYLTLRNGFITVHLSAGSKFNFQKYIKRSLEDDFKVVMGVSPVLWASFVLFILLNVDGW

HAMFWASLIPVVIILAVGTKLQATLAKMAIEITERHAVIQGIPLVQGSDKYFWFGRPQLLLHLIHFALFQNAFQITYFLW
IWYSFGLKNCFHADFKLAIVKVALGVGVLCLCSYITLPLYALVTQMGSRMKKSIFDEQTSKALKNWHNTVKKKHGLKLGK
SSVRTMEGSTTDSTVHSTTPTLLRFKTTGHSTRSVSAYDDQDDYHSDIEMSPTSPTSNLLVRVDHVEQHTIENEHQTEVK
TIDKQPTMRGSFSFVKPDDHVERSTD

>CaMLO13 Ca_07953/LOC101510624
MAEELNQSLEYTPTWIVAVVCSIIVFISLCAERALHYLGKFLKKRSQTALYEALTKLEEELMLLGFISLLLTVFQGLISD
ICITPKHATQMLPCKRSDEGLEHEIHYDTIINKRRLFSSDSGSQHCRQEGKVPLLSLEALHHLHIFIFVLAVVHVVFCVV
TMILGGVRIREWKSWEDHAQSMAISSSGETLRVEEAGFFDKYAQGYWRKAAVVGWLRSFFKQFYGSVTKYDYLALRHGFI
KEHCPNKPSFDFHSYMIRTLEIDFKRVVGISWYLWLFVVLFLLLNLEGWHTYFWLAFLPLIILLLVGAKLEHIITRLAQE
AVANVDRTERVKPSDEYFWFGRPAIVLHLLHFTLFQNAFEIAFFFWIWCTYGFDSCIMEKIAYVIPRLIMGVIVQVLCSY
STLPLYTIVTQMGSSYKKFDDMGESSSLFHRMTKESNQHPQIGEQSIIMMENHAISSTIELSHTV

>CcMLO1 C.cajan_46346
MVEEKRVYERTLEETPTWAVAVVCFVLLAISILLEHIIHAIGKWFKRKNKNALFEALEKVKGELMVLGFISLMLTVLQDP
ISKICVSPKVASSWHPCANPKPFGTTEDKSGEDESGDDDVEINSRKLLQYYYETIPRRVLATKGYDKCFAKASSPSSLLF
GKVAFVSAYGIHQLHMFIFVLAIFHILQCIVTLTLGRTKMRKWRAWENETKTIEYQFYNDPERFRFARDTTFGRRHLNSW
SQSAFSLAIVSFFRQFIGSVNKVDYLTLRHGFITAHLAPGSDARFDFQKYIQRSLEEDFKVVVGISPIIWFFAVLFLLTN
THGWYSYYWLPFMPLIIILLVGAKLQMIITKMGLRIADRGEVVKGAPVVEPGDDLFWFNRPRLLLFLIHLVLFQNAFQLA
FFSWSTYEFSIDSCFHETTVDIVIRLAMGIIIQVLCSYVTLPLYALVTQMGSTMKPTIFNQRVASALKNWHNTAKKQIKN
SKQTTPFSSRTSTPTHGMSPIHLLQRHLAGRSDSAQTSPRASNYENEQWDLEGSPSPSNHAVASNETQMQVFEPGTTSEL
PVSSQLEIRVSSSEFSFEKRHHEGGD

>CcMLO2 C.cajan_07194
MGDKVQVLQKKLDQTPTWAVAVVCLVMLAISIFIEHAIESLGKLLKKKHKKALHEALEKVKGELMLLGFISLLLVVFEDR
ISDICISKEAASSWHPCGPDDKHKDPKGYYDKCAEKASLFPHFFIIYFHCIRYYQQLKPTCFPSILFFDILQGKVAFVSE
YGIHQLHIFIFVLAIFHILQCIITLTLGRTKMSIWRMWEDESKSLEHQFYHDPERFRFARDTTFGRRHLSSWSRSPISLW
IVSFFRQFYGSVNKVDYMALRHGFIVAHLTPASEAKFDFQNYIKRTLEEDFVVVVGISPIMWFFAVLILLTNTHGWYAYF
WLPFIPLIIILLVGTKLQMIITDMALRIQDRGEVVKGAPLVEPGDELFWFNRPRLVLSLIQLVLFQNAFQLAFFAWSTYD
NGLKINSCFHRTTADIVIRLTMGVLTQVLCSYVTLPLYALVTQMGSTMKPTIFNDNVAAALKKWHHTARKHIKHKDSSHS
AANTPFSSRPGTPTHGMSPVHLLHKHPRHSDSPLTSPRAPHYHNEQWDVEGSPSPSHHARNHEDTMQPPTAELTPIGLQP
IRTQHEINIALSEFSFGRGHHTNNSN

>CcMLO3 C.cajan_32587
MAEDKIYERSLEETPTWAVAVVCFVLLAISIIIEHIIHAIGKWFKKKHKSALYESLEKVKGELMMLGFISMLLTVFEGPL
SKICISQNVASTWHPCHKPKSDTGTNGRKLLQYLELISRRVLAAKANDKCAEKAILMHYVAFNLQGRVAFVSAYGIHQLH
IFIFVLAVFHILQCIITLALGRTKMRRWKKWEDETKTIEYQFYNDPERFRFAKDTTFGQRHLNTWSHSSISLWIVSFFRQ
FFGSVKKVDYFALRHGFITAHLAPGSDARFDFQKYIKRSLDEDFKVVVGISLTFWFYGWYSYYWLPSIPLIIILLVGAKL
QKIITKMGLRIQDRGEVVKGAPVVEPGDDLFWFNRPRLLLFIIHLVLFQNAFQLAHFAWSTYEFSINSCFHRSTADIVIR
LTMGVVIQVLCSYVTLPLYALVTQMGSTLKPTIFNERVAAALKKWHHAAKNHVKDSKHCEANSTTAFCSRSSTPTFGMSP
VHLLNRHLAGKSDSPQTSPRTSNYENEQFDFENEPSTSTHSGTEETPMQVFGPPSTTELPISTKLEVHVSSSDFSFEK

>CcMLO4 C.cajan_12516
MGEENEEGRSLAETPTYAVATVITVLVSLSFLFQGTLKKLVKWLDKTKRKSLLSALEKIKEELMLFGLLSLLMGHWIIFV
AKICVKSSVLSSRFFPCAMDKNSAKRIVWPSSESINKTDFKGLEISGRHNYCPEGHESLASYESLEQLHRFVFVLGVTHI
TYSFIAVALAMIKIYSWRTWENEAKTTAIQSIQDTPQSTSSIRLRRLSTFIFHRTSHPWSNHKILVWLLCFSRQFWSSIH
RADYMALRLGFITNHELPLTYDFHNYMLRSMDEEFRDIVGVSVLLWVYAICCIFLNFHGSNFYFWLSFVPAILILIIGTK
LHRVVVKLAVEIVDRCPVIKPHRFNLRDELFWFGKPRFLLRLIQLISFLVIFQNAFEMATFLWSLWEIKDPSCFMSNHTF
IVIRLSFGVISQVWCSFITFPLYVIITQMGSRFKKTVVTENVRKSLSKWQRRVKDRQSSSYAVLTAISASSYGTVSNSRA
SNSEEGSCSGIKDICLDNVSNTQENAPFQSYGDHHDHNHNVNDDSSSPPPS

>CcMLO5 C.cajan_34110
MEGRSLAETPTWAVAVVCFVLLSISIFIEHILHLIGKWLKQKQKSDLYESLEKIKSELMLLGFISLLLTVGQGVISRICI
TEKVADTFHPCPKDHHQNTSARRLLSDNQNHRRILAAGGSDKCPPGKVPFVSSEGIHQLHIFIFVLAVFHVLYCILTLAL
GRAKMRRWKRWEEETKTAEYQFSHDPERFRFARETSFGRRHLKFWTQNLVLVWIVCFFRQFVRSVPKVDYLTLRHGFMMA
HLGPQSHMKFDFRQYIKRSLEEDFKVVVEISPPIWFITVFFLLFNTHGWYSYLWLPFVPLIIILLVGTKLQVIITKMGQR
IEQRGEVVKGVPLVQPGDDLFWFKKPRLILYLINFVLFQNAFQMAFFSWTALQFRMTSCFHSETKDMAIRISMGVFVQFL
CSYVTLPSYALVTQMGSTMKPTIFSGRVAKALRKWHHTAKEHVKQNRGLQSHNSLPCKPTTPKPPTSPAQLLRHCHSQMD
TYPSSPTRSDSEAQYHYEIDSTSFSDEHHHKSKAVADSNSIQLHEMEMGHLAHDQPQRDNEPSSTLEGLGLSHHEIHIEH
TK

>CcMLO6 C.cajan_34112
MAGRSLEETPTWAVAVVCFVLLSISIFIDHIFHLIAKWLQKKHKKALFESLEKIKSELMLLGFISLLLTVGQGVISRICI
TEKVADTFHPCPKDHHQTSSRRRLLSDNQNHRRILAAGGTDKCPHGKVPFVSSEGIHQLHIFIFVLAVFHVLYCILTLAL

GKAKMRRWKRWEEETKTAEYQFSHDPERFRFARETSFGRRHLKFWTQNPVLVWIVCFFRQFVRSVPKVDYLTLRHGFMMA
HLGSQSHQKFDFRQYIKRSLEEDFKVVVEISPPIWFIAVLFLLLNTHGWYSYIWLPFAPLIIIILLVGTKLQVIITKMGQR
IEQRGEVIKGMPLVQPGDDLFWFKKPRLILYLINFVLFQNAFQLAYFSWTALQFGVKSCFHSHTEDLLIRISMGLFVQFL
CSYVTLPLYALVTQMGSSMKATIFSERVAEAIRKWHHTAKKQIKNNHGLASQSQTPFSSRPTSPAHPLRHYRSEIDTFLS
SPIRSESLAHQPNDIDSLSYFNSHHHRSKVNAASISIEMGHLAHDPPQNVKEPSSALEGSDPTQHEREFSFDKRLQI

>CcMLO7 C.cajan_21483
MAGGSGGRNLEETPTWAVSAVCFVLITISIIIEHIIHLIGKWLKKKHKRAMYESLEKIKSELMLLGFLSLLLTVGQGPIS
RICISEKVAGTWHPCSKDDESHDSDSEDSQHGTNGRRLLAAFYASDTRRLLAGGGSHKCAQGKVPFVSSDGIHQLHIFIF
VLAVFHVLYCVITMALGRAKMKRWKRWEEETKTPQYQFSHDPERFRFARDTSFGRRHLSFWTKNPVLMWLVCFFRQFVRS
VPKVDYLTLRHGFIMAHLAPQSHSKFDFRKYIKRSLDEDFKVVVGISPPFWFFAVLFLLLNTHGWYSYLWLPFIPLIIIL
LVGTKLQVIITEMGLRIQQRGEVLKGVPLVQPGDHLFWFNRPGLILYLINFVLFQNAFQLAFFSWSALQFGIRSCFHSHT
EDVVIRITMGVLIQILCSYVTLPLYALVTQMGSTMKATIFNERVAVALRNWHQTAKKHIRQNRVGPLSLSGTPTSSRPTT
PSHHMSPVHLLRYYRSEADSFPASPRRSNLDSQPWDNSNINVDSPSPSYSHHQLEMAPQTNDPTVDTTHHEIVVARHSKE
FSFDKRPTTNNT

>CcMLO8 C.cajan_16636
MAEELSQSLEYTPTWIVAVVCSIIVFISLCVERAIHRLGKYLKKKDQTALYEALQKLEEELMLLGFISLLLTVFQGLISH
ICISPNLATQMLPCKIPHGSSEASEHDQIYYDAIINRRRLFSTDTGSEHCRQKGKIPLLSLESLHHLHIFIFVLAVVHAA
FCVITMVLGGARIRQWKKWENFNRKEYISSNKEYHEFFNIHTDGHWRRAAVVSWLRSFFKQFFGSVTKNDYLALRYGFVQ
QHHPNVPEYDFYDYMQRTLEVDFKKVVGISWYLWLFVVLFLLLNLEGWHTYFWLAFLPLILLLLVGAKLEHIIARLAQES
IDMMGKKGTKHKQVKPSDEYFWFARPALVLDLLHFILFQNSFELAFFFWIWCTYGFDSCIMEKISYIIPRLIIGVIVQVL
CSYSTLPLYTLVTQMGSKIKADKVYESPLMGPEGGSSNSRRSNRMIKESNQCAQIDEQAIIMVEDATSIIELPHIVQSPY
ERPNASNIH

>CcMLO9 C.cajan_01454
MSSSKLRNLFWGVLFSWIWFGGLAMAASDSSSSSSRDLDQTPTWAVAAVCTVFILISITLEKSLHKLGTWLGQKHKKAML
EALDKVKAELMILGFISLLLTFGQSYIVKICISEKIASKMLPCPYNYESTDSASDTEENHRRKLLSYERRYLAADTTSYK
CSKEGHEPLLSVNGLHQLHILIFFLAVFHVLYSAITMLLGRLKIRGWKAWEAETSTHDYEFAHDASRFRLTHETSFVRAH
ASYWTRISIFFYISCFFRQFYRSVRKTDFQTLRHGFINVHLAPGSKFNFQKYIKRSLEDDFKVVVGVSPVLWASVVVFLL
INVNGWRTPIWAALIPIIIILAVGTKLQAILARMALDITERHAVVQGMPLVQGSDKYFWFGQPQLVLHLIHFALFQNAFQ
VTYILWIWYCFGVRNCFRTDYKLAMIKVAIGVVMLCLCSYITLPLYALVTQMGSRMKKAIFDEQTSKALKKWHMAVKKKH
GAVKLGKSSARTLDNGSPIGSTMSSPGPTLHRFKTTGHSTRSSTYEDQDDYESDDVEVSPVSQTTSFIVRVDPGEQHAEE
EEEDHKHHSHSEGEINDEAEFTFVKLKPHPGQRTTT

>CcMLO10 C.cajan_18941
MSGGGGGDARQLDLTPTWAVAAVCAIIVIISILLEKIIHKFAKMFEEKKKHALLEALEKIKAELMILGFISLLLTFGQNY
ISKVCIPERFSYTMLPCLPVEKRGGGHGEHGAAPHEEEGGEAEAEEHHRRLLSYERRFLSAEAGGHSCELGHVPLISVNG
LHQLHIFIFFLAVFHVIYSAITMTLGRAKIRGWKAWEQDHIVDQDALNDPRRFRLTHETSFVRDHNSFWTKTPVTFYFVC
FFRQFFRSVRRADYLTMRHGFVTVHLAPGSKFDFQKYIKRSLEDDFKVVVGISPILWASVVLFLLVNVHGWHASFWVSFL
PLLVILAVGTKLQAIITRMALDIKERHAVVQGIPLVQVSDKYFWFAWPQLVLYLIHYVLFQNAFELTYFWWTWYEFGWAS
CFYEDDSLMIVRVALGIGAQVVCSYVTLPLYALVTQMGSTMKKSIFDDQTSRALKQWHKNALKKKASKGRAETRTLGASP
GESPDVSPRPTTAEETEMAEHGATIVSTVDNEQQQYNNRDLLTGP

>CcMLO11 C.cajan_18939
MAVAASGERSLQATPTWAVAVVCSVFVIISVIIEHGIHSLGKWFQKRHKKAMNEALEKIKAELMLLGFISLLLTFGTKYV
AKICIPESFGDTMLPCKKVKPDDDSNGRRRLLSLDESVEWRRVLEAASGGGDSCSQQGKVPLISQSGVHQLHIFIFVLAV
FHIFYSVMTMVLARAKMKKWKAWEAETTSLEYQFTNDPARFRFAHQTSFVRRHSGWSRMPGIRWIVAFFRQFFASVTKVD
YMTMRHGFINAHFAPNTKFDFQKYIKRSMEDDFKVVVGISIPLWAFAIIFLLVNVYKWYTLTWLSLAPLVILLLVGMKLE
LIIMEMAQEIQDRTTIVRGVPVVEPNNKYFWFNKPQWIIFLIHFTLFENAFQIAFFLWTWYEFKITSCFHENLPLILTRV
VLGIALQIVCSYITFPLYSLVTQMGSHMKKAIFEEQTAKALKKWQKTAKDKRKLRKAGIEIASGETTPSHGTSPLHLLHK
SKPSHSHIEMDSVLYSPRSYQSDTDLSETEGSSHQQNEITQTHQPPRNNGLTHDIDFSFEKL

>CcMLO12 C.cajan_36621
MSVSKYKKLCLCLVSLFCFGCVAMASSESSSGSKELDQTPTWAVACVCTVFILISLTLEKSLHKLGTWLREKHKKALLEA
LEKVKAELMILGFLSLLLTFGQSYIVRICIPLNVADKLLPCKYVGTDNESSSEEEHRRKLLSYERRYLSDEATPYQCGKG
HQPLISVNGLHQLHILIFFLAVLHVLYSAVTMLLGRLKIRGWKAWEAETSSHGYEFANDPSRFRLTHETSFVRGHASFWT
RYSIFFYIGCFFRQFYRSVGKADYLALRNGFIAVHLSPGSKFNFQKYIKRSLEDDFKVVVGVSPVLWAAFVFFLLLNVYG
WHAGVWASLIPFFIIFAVGTKLQAALAMMAIEITERHAVVQGIPLVQGSDRYFWFGQPQLVLHLIHFALFQNAFQITYFL
WIWYSFGLKNCFHADTEVAIVKVALGVGVLCLCSYITLPLYALVTQMGSRMKKSIFDEQTSKALKKWHMAVKKKQGVKLG
QSRVRAMDGSTTDSTVHSSGPTLHRFKTTGHSTSHTMSTYGDQDDYHSDNELTSISPTSSLIVRVDHDEQEPAKEIEHHP
TANNQ

>CcMLO13 C.cajan_17246

MAEVLQRGRSLAETPTYSVASVVTVMVFVCFLVERSIYRFGEWLRKTRRKALFASLEKIKEELMLLGLISLLLAQSARWI
SEICVDSSLFSSRFYICSAQDLGTTENIMHESLSSFPDETTIPKGLNSGAFNQCGAGHEPFVSYEGLEQLHRFLFVLGIT
HVMYSCLAVGLAMSKIYSWRRWENQAKMAIDGNLQGKKIKVMRRQTTFVFHHTSHPWSRSPILNWMLCFLRQFRSSIQKS
DYLALRLGFITEHKLPLSYNFHKYVVRSMEDEFHAILGISWPLWLYAIVCIFVNIHGLNIYFWLSFIPAILVMLIGTKLQ
HVVSTLALEIMKQTGPSARTQVKPRDDLFWFKKPDILLWLIQFVIFQNAFEMATFIWTLWGFQEQSCFTRNHYMIITRLA
SGALVQFWCSYMTVPLNIIVSQMGSRCKKALVAESVRESLHSWCKRVKHKSKHDSVHSHTARSVCSLESTIDERDEITVV
SGTLTRSSSLESLNQITVTSVDQLNFETPNNPKDSIKVTEYLSESVYTNTNNPPQPSYNGEDHVGNGEEAKVDTLLDLFQ
KT

>CcMLO14 C.cajan_23778
MSGGGGEEGDTLEFTPTWVVAAVCTVIVAVSLAAERFLHYGGKFLKAKGQKPLFEALQKIKEELMLLGFISLLLTVTQNG
ITKICVPPGLTRHMLPCDPNQAPNHESHFQYHSFFPGTARRLLAEAHSESETQTGYCARKHKVPLLSLEALHHLHIFIFV
LAIVHVTFSLLTVVFGGARIRQWKHWEDSIAKQNYETARVLKPKVTNVQQHDFIRGRFAGFGKDSAIVGWLVSFSKQFYG
SVTKSDYVTLRHGFIMTHCKSNPKFNFHKYMIRALEDDFKQVVGISWYLWLFVVIFLLLNINGWHTYFWIAFIPVILLLA
VGTKLEHVITQLAHEVAEKHAAIEGDLVVQPSDEHFWFHRPRVVLFLIHFILFQNAFEIAFFFWIWVTYGFDSCIMGQVR
YIVPRLVIGVFIQVLCSYSTLPLYAIVTQMGTHYKRAIFNEHLQQNIVGWAQKAKRRKGLKAETESHSGQGSAHESSGAG
AGAGSATGIQLGSIFKKASAPGDSTSVPKADGN

>CcMLO15 C.cajan_47280
MGGGGAEEGNNLEFTPTWVVAVVCSVIVAVSFAAERFIHYGGIFLKKKNQKPLFEALQKIKEELMLLGFISLLLTVTQNG
IVKICVPVSWTHHMLPCSFDLKEKEESTKTTSHFQTFFSSSNISGIARRLLEESKSESENHEPSTVTKLGHCAAKGKVPL
LSVEALHHLHIFIFVLAIVHVAFCVLTVVIGGLQIRQWKHWEDSIVKENYETQPVLEPTVTHVQEHAFIQNRFTGFGKDS
AVLGWVKSFFKQFYGSVTKLDYVTLRLGFIMTHCRGNPKFNFHKYMIRALEDDFKKVVGISWYLWIFVVIFLLLNINGWH
TYFWIAFIPVILLLAVGTKLEHVIIQLAHEVAEKHSAIEGELVVQPRDDHFWFKRPHIVLFLIHFILFQNAFEIAFFFWI
WVTYGFDSCIMGKVRYIVPRLVIGVFIQILCSYSTLPLYAIVTQMGTHFKKAIFDDQVQARLVGWAQKAKKKGLRGDNNG
QGSSHAGAGIQLGPMFRRASATEDNVVVPRNDGSE

>CcMLO16 C.cajan_13783
MDIGKERTLEETPTWAVSVFCFFFLTISLIIEGGLHKLTEILRKRKGKSLGKALTKTKTEMMKFGFMSFLLTISEVPISK
ICITKDVANSLLPCKDAVDLTGLTSAPATRTSTSEFDIAPATNESTIEVNYCEAKGMVSLISSDGILQLNIFISFLAVFH
ILFCTLTMCLGKAKMRRWKRWEDETRTLEYQIANDPRRFQYTGQTLIGKRHLKFWSYHSPLLWMVCFFRQFYGSVSKDDY
FTLRNGFIAANISLGSNFNFKKFLSRTYDEDFEKVMGIRIWIWIFSILFIFFSAHEFYNYYWLPFIPLVVALLAGAKLQV
IITKMCVDSCKENPVIKGSLLVTPSDAHFWFHRPEWLLHLLKFILIQNSFQLAFFTWTWYEFGPRSCFNREKEDIAIRIA
MGVAVQLLCGYVTLPLYALVTQMGSSMRREVFTESVSKGLKNWHKKAKQSLCKNNSTSSKHSNSLHSTVGESSIRGAVDS
VHNPDNVAVTSYSSPLTSEVEKRIAPTHEQEMSSSEIPTIEEENNPKIITRGTYDGEISFGSSWKNVGSSRGIGEFGSIT
EEDDAEKLSELNP

>CcMLO17 C.cajan_37300
MEENVQETRSLALTPTWSVATVLTVFVAVSLLVERSIHRLSNWLRKTNRKPLLAALEKMKEELMLLGFISLLLTATSRMI
ANICIPSKFYNSAFAPCTRSEIDEQMEENGSEERKLLMASVYPHLFRRVLNGINRSSCKEGYEPFVSYEGLEQLHRFIFV
MAVTHISYSCLTMLLAIVKIHSWRVWEDEAHMDRHNSLTEITKELTMRRQSTFVKSHASNPLVKNGSLIWVTCFFRQFGR
SVVRADYLTLRKGFIMNHNLSLKYDFHSYMVRSMEEEFQRIVGVSGPLWGFVVAFMLFNIKGSNLYFWIAIIPVSLVLLV
GTKLQHVIATLALENAGITGFFSEAKLRPRDELFWFNKPELLLSLIHFILFQNAFELASFFWFWWQFGYNSCFIRNHLLL
YLRLILGFAGQFLCSYSTLPLYALVTQMGTNYKAALIPQRIRETIHGWGKAARRKRRHGMFTDDSTIHTDTSTVLSIEED
DQLIDTPGDPVTGPEVELQPVTTVTSSPSPIANETSSRAVTPLLRPSASISSVQCSSKTEAIPRCSSMPSGR

>CcMLO18 C.cajan_37291
MEENEQESRPLAFTPTWSIAIVLTFFLAFSLLLERAIHHLSDWLKKTNQKPFLAALEKMQEEMQLLGFVSLLLIATSDMI
ANICIPEKFYNDAFAPCTRSEIAEETEKNDTEEHKLLMASTNPRVIRRILDDINKKSCNEGYEPFVSHEGLEQLHRYIFI
IAVTHISFSCLTMLLAIVKIHSWRTWENEAQIDPHNCSFSEIPRHVPALSQTSLIMVHRSNPLFRNTFLIWVACFLRQFW
NSLVRSNYLTLRKGFIMVSNSVCLKSKCFLFESNIICTTRKIKICDENFVPLWGFVVALMLFNIKGSNLYFWIAIIPIFL
VLLVGTKLQHVVVTLVLENAEITDFFSGAKLTPRDELFWFKKPELLLSLIHFILFQNAFELATFCWSWWEFGNSSCFNRN
HLLVYSRLILGFIGQFICSYSTLPLYALVTHMGTNYKAALIPESVRATIDVWGKAAKRNRKQGKVTDDFELEPLREETSA
NNVSSRPVDPVLRPSASVNSELRFRTETIPRSTSMPREI

>CcMLO19 C.cajan_09813
MAAYASGYSLEHTPTWAIALVSFILISISIILEHLIHLIIKWLRKHRKIVEAIERLKSELMILGFMSLLLTVTQDAIIEI
CIPMKTADTMLPCRKRTTNDTATNLDSCSAKNASKVALVSKHGIHQLHMFVFVLAVMQIVYSFLTVSLARAKMRHWKAWD
EETQTIEYEIANDPNRFRYSRQTTFGRRHISTRTASPVYVWIKCFFRQFFHSVEKVDYLTLRHGFISAHFSARNSNFDFQ
NYIEQSLEEDFRIIVSISPVMWFTVVIFLLVDVHGWHVYLWLSYVPLMLVLVVGAKLEVIVDEMALQMKDINNVTKGTPL
VCPSDKLFWFGHPGFVLTLIHYTLFVNAFELAFFIWVSTQFGINSCYHEHRSFTIIRVVIAVAVQVLCSYVTLPLYALVA
QMGSQVKSKALAKILKQWHVEVRERRRKQEKLVKSFSFRHRNKSSEWGQGNKSAPEFSSTLGESNRSYDEGEIVEELEHS
VKTKASSSSDPPSFV

>CcMLO20 C.cajan_24758
MKQPNISNCPLIVHMANVSEGTLENTPTWAVTTVCFVLLVISISIEQLLHLLARYFKRKRRKSLTKALEKIESELMLLGF
MSLLLTVSEKRIANICITKSVGETLLPCATMTFDVTKEETKCAEQGKVSLVSRKGVRELQYFIFYLALCHVISCILTFSL
GMAKMRRWESWEAETRTLEYQFQYDPRRFQLTRETSFGKRHLNYWSNNPILFWPVCLVRQFYRSVPKVDYFTLRRGFITA
HFAEGSNFDFQNFIKRALEKDFGVVMGISWWIWIFSVLYIFLNANVFYSHFWLPFSPLMVLLLVGTKLQGIVTHMCLDSD
DKSQIIKGSLLVRPSDHFFWFGWPNLLLHLISFVLFQNSFQVAFFTWTSIRFGIRSCFHRGIENIIIRVAMCVSVQILCG
YVTLPLYALVTQMGTSMSKDVFAENVIRGIHIWREKAKKNVAHRNPHSQDSSLDTSLSLEISLESPSFRFDVSTSILMDR
SSDDNKHVAVTEENISSEQLGSFQGFHLQNTGKPRQF

>LaMLO1 AOCW01137726_AOCW01137727
MAETRIYERTLEETPTWAVAVVCFVLLFISIIIEYSIHAVGKWFKKKHKKALYEALEKVKGELMLLGFLSLLLTVLQDPI
SKICISKNVASTWHPCSTPKASKTEEDSHSDENDTNSRKLLQYLNPMPRRFLATKGYDKCAAKGKVAFVSAYGAHQLHIF
IFVLAIFHILQCIITLALGRTKMRRWKTWENETRTIEHQYYNDPERFRFARDTTFGRRHLKKWSESSISLWMVSFFRQFI
GSINKADYFALRHGFIMTHLAPGSDAKFDFQKYINRSLEEDFNVVVGITPLIWFCAVLFLLTNTHGWYSHFWLPFIPFII
VLLVGTKLQMIITKMGLRIQDTGEVVRGAPLVQPGDELFWFNRPRFLLFLIHLALFQNAFQLAFFTWSTYEFGLHSCFHD
TTADIVIRVTMGVLVQFLCSYVTLPLYALVTQMGSTMKHTIFNDRVATALKNWHHTAKKHVKDSKHASANVTPFSSRPTT
PTHGLSPVHLLQQHHGRSDSTQTSPRTSNYENEEWDIEGSASPTKHIIGEGDETQILEQDSTTQLPISSQHEISISVSEF
SFEKRLHRDYE

>LaMLO2 AOCW01065981_AOCW01147792
MGEKVYERTLEETPTWAVAVVCFVLLFISIIIEYIIHAIGKWFKKNHKKALYEALEKVKGELMLLGFLSLLLTVFQEPIS
KICVSKNVASSWNPCSTPKASKTENHSDNTNSRKLLQYLEPMHRRILATKGYDKCAEKGKVAFVSAYGIHQLHIFIFVLA
IFHILQCIITLTLGRTKMRRWKTWEDETRTIEYQYYNDPERFRFARDTTFGRRHLNKWSESSISLWMVCFFRQFIGSISK
VDYFALRHGFIMAHLAPGNDAKFDFQKYINRSLEEDFNVVVGITPIIWFFTVLFLLTNTHGWYSQFWLPFIPFIIVLLVG
AKLQMIITRMGLKIQDRGEVVRGAPLVQPGDELFWFNRPRFLLFLIHLVLFQNAFQLAFFTWTTYEFTLYSCFHDTTADI
VIRVTMGVVVQILCSYVTLPLYALVTQMGSTMKHTIFNDRVATALKSWHHTAKKHVKDSKHASASVTPFSSRPATPTHGL
ISGTPTVQAPRPSDSTQTSPRASNYENEEWGIEGSASPNNHNTINIGEGYETQMQVLEPDSTTELPISSEHEISLSVSDF
SFEKQHIGRDYE

>LaMLO3 AOCW01159416
MEENGKRRSLDATPTWAVATVITLMVSLSFMFQITLEKFGKWLDRTKRKSMLSALEKVKEELMLFGLLSLLMGHWTIFVA
KICVKESVLKTRFIPCAIEKNSGTVEHIFWLSSEYSNRTMLEENVNKGLHNYCPEGKESFASYESLEQLHSLLFVLGVTH
VFYSFIAVALAMIKIYSWRIWESDAKTMATQSFQDTPQNTLNIRLSRLGTFIFHHTSHPWSHHKILVWLLCFSRQFWSSI
NRADYMALRFGFITNHELPLTYDFHNYMLRSMDEEFRDIVGISVPLWIYAICCIILNFHGSNIYFWLSFLPSILILMIGT
KLHRVVVKLAVEITSHCQDTRPLNLRDELFWFGKPKLLLRLIHLISFLNAFEMATFLWSLWEIKEASCFMKNRTFIMIRL
SFGVISQVWGSFITFPLYVIITQMGSKMKKSVISENVRKSLSKWQGRVKNRQGSSLMHDYESFPSCEDYHHHNV

>LaMLO4 AOCW01114694_AOCW01136624
MAGGIGGRNLEQTPTWAVAVVCFVLISISITIEHTIHLIGKWLTKKNKRSLYESLEKIKSELMLLGFISLLLTIGQSLIS
KICISEKVARTWHPCSDKEDHTSSSKNETNQRRLIATTLFFGLESENPRRVLAGGGSDKCGKGKVPFVSTDGIHQLHRFI
FVLAGFHVLYCILTLTLGRAKMRRWKQWEEETKTLEYQFSHDPERFRFARDTSFGRRHLSFWTKNPLLIWIVCFFRQFVR
SVPKVDYLTLRHGFITAHLAPQSQQKFDFRKYIKRSLEEDFKVVVGISPLFWFFAVMFLLFNTHGWYSYLWLPFIPLIII
LLVGTKLQVIITKMALSIQQRGEVLKGMPLVQLGDHLFWFNRPSLILHLINFVLFQNAFQLAFFAWTSFEFGIKSCYHSH
TEDVVIRISMGVLIQILCSYVTLPLYALVTQMGSSMKASIFNERIAMALRNWHHRAKKHKKQNRNGSLSMTPISSRATTP
THSMSPTHLLSHYRSQIDSSYYASPRRSIEIEDSPSTSYSYHHKNKKAKVDYFFDPTPSTTTTTSTNHHEIEMDQHINEV
NPTTTQHEIVIAHHSKEFSFDKGTTQSSAQNTSKLGR

>LaMLO5 AOCW01127929
MAEELNQSLEYTPTWIVAGVCSIIVFISLIVERCLHWLGKYLKREKQTALYEALIKLQEELMLLGFISLLLTVTQNIISD
ICIPPKLATQMLPCKRPHESSEESTHRLIYYDTIINRRRLFSTETGFEHCKEKGKVPLLSQEALHHLHIFIFVLAIVHAI
FCVTTMLLGDHLYIRNGIDLSLKFPIFSVFSYPDDYHHHEFFKKHADGYWRRAAVVGWLISFFKQFYGSVTESDYTALRQ
GFIKEHCSHKPDFNFHKYIMRTLEVDFKKIVGIRWHLWLFVVLFLLLNLHGWHTYFWLSFLPLIILLLVGAKLEHIIVRL
AQESVEKLAEGNFVTQAVGNLGRRKEQDAKPVKPSDGYFWFNRPALVLDLLHFTLFQNSFEIAFFFWIWCTYGFDSCIME
KIGYIIPRIIMGVIVQVLCSYSTLPLYTLVTQMGSRFKKGMFDEVVEAGILSWAGKSISGADDESSEPDRRRTMSHKMIK
ESFQPSQISGQAIIMVEDETTSTIELPTLSKFH

>LaMLO6 AOCW01133085
MGGGGGGGEGGHGHTDNRTLEETPTWSVAMVCAVIVIISISLEKFIHKFAKVFEAKKKLALLEALEKIKAELMVLGFISL
LLTFGQSYISKVCVPQYYANQMLPCSRKKSHGPPEEGHDTGGGGGENEAAEHRRRLLSYERRFLAGGGGFVECKLGSEPL
ISVGGLHQLHIFIFFLAVFHVIYSAITMTLGRAKIRAWKQWEQSHLVDEDALNDPRRFRLTHETSFVRDHSSFWTKTPVS
FYFVCFFRQFIRSVRRADYLTMRHGFVSVHLAPGSKFDFQKYIKRSLEDDFKVVVGISPILWASVVLFLLVNVNEWHASF
GLSFLPLAVVLSVGTKLQAIITRMALDIKERHAVVQGIPLVQVSDKYFWFQWPQLVLYLIHYVLFQNAFELTFFCWTWYE
FGLNSCFYENKKLMYFRVAVGVGAQIVCSYVTLPLYALVTQMGSTMKKSIFDDQTSRALKKWHKNALKKANSKGRGTEIR
TLGGSPRHSPRASENAYSNIAVTETQMTEQTHQGL

>LaMLO7 AOCW01138453
MGGGGDAGAGSHGHGDNRTLEQTPTWSVAAVCAIIVIISISLEKLIHKFASMFEQKKKLALLEALEKIKAELMVLGFISL
LLTFSQSYISKVCVPHHYANQMLPCSRPEESYGAAHGPPKEEEGGEDEGEHRRRLLSYERRFLAGGGGMVNCKPGYEPLI
SVNGLHQLHIFIFFLAVFHVIYSAITMTLGRAKIRAWKEWEQDHLIEEDALNDPRRFRLTHEISFVRDHNSFWTKTPISF
YFVCFFRQFIRSVRRADYLTMRHGFVSVHLAPGSKFDFQKYIKRSLEDDFKVVVGISPLLWASVVLFLLVNVHEWHAAFV
LSFLPLSVVLAVGTKLQAIITRMALDIKERHAVVQGIPLVQVSDKYFWFQWPQLVLYLIHFVLFQNAFELTFFCWTWYEF
GLGSCFYEDKFFMFFRVAIGIGTQIVCSYVTLPLYALVTQMGSTMKKSIFDDQTSKALKMWHKNALKKANSKGRGTEVRT
LGGSPGHSPPHSPHANGNAGTNIVVAAETQMTQQNAAAIATNADHNQNQQYENIDLLSGP

>LaMLO8 AOCW01146534
MSKSKLKKLLFYVLFSMLVWFGSYGMAASESSGNSRELDQTPTWAVAAVCTVFILVSITLEKSLHKLGTWLGHRQKMALF
EALEKVKAELMILGFISLLLTFGQSYIVKICISASVADKMLPCPYREKGTNNASSSGGEHRRKLFSYERRYLAADATSYK
CSDEGHVPLLSVNGLHQLHILIFFLAIIHVLYSAVTMLLGRLKIRGWKAWEEETSTHGYELSIDASRFRLTHETSFVRAH
ASFWTKISILFYIGCFFRQFYSSVGKVDFQTLRNGFIAVHLAPGSKFNFRKYIKRSLEDDFKVVVGVSPVLWASVVVFLL
LNVNGWQTRIWAALIPVVIILAVGTELQVILTKMALEITERHAVVQGIPLVQASDRYFWFGQPQLVLHLIHFALFQIAFQ
ITYILWTWYSFGLTNCFRIDYKLAVVELALGVIMLCLCSYSTLPLYALVTQMGSSLKKSIFDEHTAKALRKWHMTVKKKQ
GVVKLGKSTMRAMDMDGSTTIGSTKLHSSRPTLHRLKTTGHSTHTSIYEDEDEYQSDIQLSPTSSSTTLVVNVDNGHQQQ
VEENTEETNIQGDFSFVKPSPIEKNHDVELL

>LaMLO9 AOCW01154130
MSVSKYKKLCFCVVSWFCYEVGLVMGSSNEKGSGSSRELDHTPTWAVAFVCTVFILISITMEKSLHKVGMWLQQRHKKSL
LEALEKVKAELMILGFISLLLTFGQRYIVRICIPAKVANKLLPCPLKDKHRTKLVSYEHRYLSEDTTSLRCKGRYVPLLS
VTGLHQLHLLLFFLAILHVLYSAVTMLLGRLKIRGWKAWEEEIASDRFEFDNDRSRFQFTHDTPFVRAHATFWTRYSVFF
YIGCFFRQFYRSVRKADYVTLRNGFITVHLAPGSKFNFQKYIKRSLEDDFKLVVGISPVLWASFVVFLLLNFNGLQAMYW
ASLIPLIIILVVGTKLQATLANMAIEITDRHAVVQGMPLVQGSDKYFWFGRPQLILHLIHFALFQNAIQITHILWIRYSF
GKETCVHAGDILAIVKVALGVVVLCLCSYITLPLYALVTQMGSTMKKSIFDEQTKKALKKWLMDAKKNKGSKAGKSTVRT
ADGSTNGSTIHSSGPTLHRFKTTGHSTRTRTISPDYNQDDHHSDIEMSPMLPTSSLIVRVDHGDQEAKENEHQV

>LaMLO10 AOCW01039454_AOCW01039455
MSNSNFKKMFLFFLFSMPIWFGNSGMAASEGSNDSRDLDQTPTWAVAAVSTVFILISITLEKSLHKIGTWLGHKQKMALL
EALEKVKAELMILGFISLLLTFGQSYIVRICIHANVAAKMLPCPYRDQNTDNEFGNEEEHRRKVLSYQRRYLAADTTSYK
CTNEGHVPLLSVNGLHQLHILIFFLAIIHVLYSAVTMLLGRLKIRGWFHQCYFEIVLYETLRDTSRFRLTHETSCGKAHA
SCWTRSSMFFYIGCFFRQFYRSVGKVDFQTLRNGFIAVHLAPGSKFNFQKYIKRSLEDDFKVVVGVSPVHWASVVVFLLL
NVNGWQTRLWATLIPVAIILAVGTKLQAILTKMALEITERHAVVQGIPLVQASDRYFWFGQPQLVLHFIHFALFQIAFQI
TYILWTWYSFGLRNCFRVDYKLAVLEVALGIIMVCLCSYITLPLYALVTQMGSSLKKSIFDEQTSKALKKWHMTVKKKHG
KVKLGKSTVRSMDMDGSTIGSTTLHSSGPTLHRFKTIGHSTRTSTYEDEDEDEYQSDIEFSPISTSMNLIVKVDHGHEQQ
GEENREEANIEGEKRTMT

>LaMLO11 AOCW01135152
MSMSMYKKVSFCVVFWLCYGILGMGSSSESSNSSRELDQTPTWAVACVCTVFILISITMEKSLHKVGLWLQQRQKKALLE
ALEKVKAELMILGFISLLLTFGQSYIVRICIGANIAEKLLPCPYNSEGIGNESSSKDEHRRKLLSYEHRYLSEDTTSYHC
KEGHVPLLSANGLHQLHILIFFLAIFHVLYSAVTMLLGRLKIRGWKAWEEETSSHGFEFATDPSRFRLTHETSFVRAHAT
FWTRYSIFFYVGCFFRQFYRSVRKADYQTLRNGFIAVHLAPGSKFNFQKYIKRSLEDDFKIVVGVSPVLWASVVVFLLLN
VNGWHAMIWASLIPVVIILVVGTILQATLANMAIEITERHAVVQGIPLVQGSDKYFWFGRPQLVLHLIHFALFQNAFQIT
YFLWIWYSFGLKNCFHADYELAIIKVALGFVVLCLCSYITLPLYALVTQMGSRMKKSIFDEQTAKAIKKWHMAAKMKHGL
KLGKSSVRTADGSTTVGSTIHSSGPTLHRYKTTGHSTRTLSSYHDQDDYHSDIEMSPVSRTSRLYVRMDDGEQEAEENEH
LHVGETNHGDDGLTNVKPAPL

>LaMLO12 AOCW01152069
MGDVHEAGRSLAETPTYSVASVITVMVFLSFLVERSIYRFGKWLKKTRRKALFASLAKIKEELMLLGLISLFLAQSARWI
SEICVDSSLFTSQFYTCSQQEISATNHKIMLQTSPPFPPSESTTVAKEVNNVAFHQCREGHEPFVSYEGLEQLHRFLFVL
GITHVLYSCLAVGLAMSKIYSWRKWENQATMAADGNLQAKKIKVMRRQITFISHQTSHPWSRSRNPLLIWMLCFLRQFRS
SIQKSDYLALRLGFITEHKLPLSYNFHNYMVRSMEDEFQAILGISWPLWIYAIVCIFVNIHGLNIYFWLSFIPALVVMLI
GTKLQHVVSTLAIEILEQTGPYARTQVKPRDDLFWFKKPDILSWLIQFVMFQNAFEMASFLWTLWGFKEQSCFMRNHYMI
IIRLTSGVLVQFWCSYMTVPLNVIVSQMGSRCKKALVAESVRESLHSWCKRVKQKSKHDSLHTARSVCSIESTIDEITVV
SGTLTRSSSLEYMNQLTVTSVDQLNFHTCDNNNQDSIHVANGEEQNVQTLLHLFQKT

>LaMLO13 AOCW01152789
MGGGGGEDTTLEFTPTWVVAGICTVIVAISLAAERVLHYCGLFLKRKNQKPLYEALQKIKEELMLLGFISLLLTVTQNSI
TKICVPSAFTHHMLPCTFQSHSQTHSHSQTIFSFPPFSRRLLSHADANFCSHKDKVPLLSLEAIHHLHIFIFVLAIVHVT
FSVLTVLFGGTKIRQWKHWEDSIAQQNYETNRVLKPKVTNVQQHDFIKGRFSGFGKDSALMGWLLSFFKQFYGSVTKSDY
VTLRLGFIMTHCRGNPKFNFHKYMIRALEDDFRQVVGISWYLWVFVVIFLLLNINGWHTYFWIAFIPFFLLLAVGTKLEH
VITQLAHEVAEKHAAIEGELVVRPSNDHFWFQRPQIVLFLIHFILFQNAFEIAFFFWIWVTYGFDSCIMGQVRYIIPRLI

IGLFIQVLCSYSTLPLYAIVTQMGTNYKKAIFEEHVQVGLIGWAQKAKMKKEQKADSQHESGQGSSHVGIQLGQLFPKRA
SAPQDNTFIPPKPDGST

>LaMLO14 AOCW01138218
MSGGGVEEGSSLQFTPTWVVALVCSVIVAVSLAVERLLHYGGMFLKKKNQKPLYEALQKIKEELMLLGFISLLMTVSQNS
ISKICVSKSWTEHMLPCSREDLEKEGSKTPISHFQTFFSFSGNARRLLSEGQAAPEVKSGYCSSKNQVPLLSIEALHQLH
IFIFVLAIVHVTFCVLTIVFGGLKIHEWKNWEDSIAKENYETQRVMNPKVTHVQQHAFIKDHFTGFGKDSSLLGWVKAFF
KQFYGSVTKLDYVTLRLGFIMTHCRGNQKFNFHKYMVRALEDDFKIVVGISWYLWIFVVLFLLLNISDWHTYFWISFVPF
ILLLSVGTKLEHIITQLAHDVAEKHAAIEGELVVQPSDDHFWFHRPYIVLFLIHFILFQNAFEIAFFFWIWVTYGFDSCI
MGQVSYIFPRLIIGVFIQVLCSYSTLPLYAIVTQMGTHFKKGIFDEQVQARLVGWAQNAKKKGVKGDSQHGEGSSHSNGA
VGVQLGSIFKKQSAPEDNAIVPTNEESKLI

>LaMLO15 AOCW01116641
MAENVEETRSLAMTPTWSVATVLTVFVAVSLLVERSIHRLSNWLRKTNRNPLFAALEKMKEELMLLGFISLLLTATSRMI
ANICIPSKYYNSAFAPCTRSEINEEMEDNGSGERKLLISSAYPHLLRRMLNGINRSSCKEGHEPFLSYEGLEQLHRFIFV
MAVTHISYSCLTMLLAIVKIHSWRVWEDEAHMDRHNSLNMTMQRQSTFVKFHTSNPLASNSVLVWVTCFFRQFGHSVVRA
DYLTLRKGFITNHNLSKKYDFHSYMIRSMEEEFQRIVGVSGHLWGFVVAFMLFNIKGSNLYFWIAVIPISLVLLVGTKLQ
HVIATLALENAGITSFFSEAKLKPRDELFWFKKPELLLFLIHFILFQNAFELASFFWFWWQFGYNSCFISNHLLVYIRLI
LGFAGQFLCSYSTLPLYALVTQMGTNFKAAIIPQRIRETIHGWGKAARKKRRHGMFPDDSTIHTDTSTVLSIEEDDEFID
DTPTTGTGTGTGVELQPITTITSSPLPAAANETSSRAATPLLRPSASISLSSPSSARAEAVARCFSMPSGRH

>MtMLO1 Medtr6g033330
MAEDKVYERTLEETPTWAVAVVCFVLLAISIVIEHIIHAIGKWFKKKNKNALYEALEKVKGELMLMGFISLLLTVFQDYI
SKICISEKVGSTWHPCSTPKTKTASNDENSESENHDRKLLEYFDPNPRRILATKGYDQCADKGKVALVSAYGIHELHIFI
FVLAIFHILQCIITLALGRFKMRRWKKWEDETRTVEYQFYNDPERFRFARDTTFGRRHLSMWTKSPISLWIVCFFRQFFG
SISRVDYLALRHGFIMAHLAPGNDAEFDFQKYISRSLEKDFKVVVGISPTIWFFAVLFLLTNTHGWYSSYWLPFLPLIII
LLVGAKLQMIITKMGLRIQDRGEVIKGAPVVEPGDHLFWFNSPNLLLFIIHLVLFQNAFQLAFFSWSTYEFSINSCFHRT
TADNVIRVSVGILIQFLCSYVTLPLYALVTQMGSTMKPTIFNERLATALKKWHHTAKKQVKHNKHSNNTTPYSSRQSTPT
HGMSPVHLLHRQTFGNSDSLQTSPRTSNYENEQWDVEGGGSTSPRNNQTVASEIEIPIVESFSTTELPVSVRHEIGTTSS
SKDFSFEKRHIGSN

>MtMLO2 Medtr5g022400
MVEENREEGRSLAETPTWAVATVITLLVSLSFLSNGTLKKLVKWLGRTKRKSLLSALEKMQEELMLFGLLSLLMGHWIIF
VAKICVKSSVLSSRFFPCALKESFGIVKHNVWSNLEYSNKTILKEQVNNDGLHNFCPKGHESFASYESLEQLHRFVFVLG
ITHVSYSFVAVALAMIKIYSWRTWENEAKTMAIQSLQDTSQSTSTSSIRLKRLSTFIFHQASHPWSHHKILVWLLCFSRQ
FWSSISRTDYMALRLGFITTHDLPLTYDFHNYMLRSMDEEFRDIVGVSVLLWIYAICCIFLNFHGNNFYFWLSFVPAILI
LIIGTKLHRVVVKLAVEVIDHSPYMKPHRFNLRDELFWFGKPRLLLRLIQLISFLNAFEMSTFLWSLWEIDDPSCFMDNR
TFIAIRLSFGVVSQVWCSFITFPLYVIVTQMGSRFKKTVISESVRKSLSKWQRRVKEKQSSSYELLNTSTTTSLESLLHG
MDNSNHSDLISEIEEGSSSGIKDICHSNFDDVSMIQSNAKIPSYDEDYHDHYHDVKHDLPPPPLS

>MtMLO3 Medtr2g093750
MAGGSVGRSLTETPTWAVAVVCFVILSISIFIEHIFHIIEKWLKKKHKSALYESLEKIKSELMLLGFISLLLTVGEGLIS
RICISEKVAATWHPCSNNANIESDDEELIDHETGGSRRLLAALLASQGDNHHRILAGGGGDKCAEEGKVAFVSAGAIHQL
HIFIFVLAVFHILYCILTLALGRAKMRRWKRWEEATKTPEYQFSHDPERFRFANETSFGRRHLSFWTKNPVLIWIVCFFR
QFVRSVPEVDYLTLRHGFMMAHLAPQSHLKFDFRQYIKRCLEEDFKVVVGISPPIWFITVFFLLFHTHGWHSYLWLPFLP
LIIVLLVGTKLQVIITQMGLRIQKQGMVVKGEPVVQPRDDLFWFNKPRLILFLINFVLFQNAFQLAFFSWTALQFGVTSC
YNSRKDGVVIRICMGVFVQILCSYVTLPLYALVTQMGSTMKPTIFNERVATALRNWHHTARKNIKHNRGSGSQTPFSSRS
ITPARSMSPAQILRHYRNQMDTPTRLNFETSHHYESYSPSPSNSHHHKVEINVASSSSTHLHEMEMGHLAHVEQQEVIKP
NSISVGSGRPQFEIDIQQSDELSFSTMPTNQLE

>MtMLO4 Medtr2g028470
MAEEELNESLEYTPTWIVAVVCSIIVFISLCAERALHRLGKYLKRKKQKALYRALNKLQEELMLLGFISLLLTVFQGVIS
HICISPEHATQMLPCKRSHESVQGSEHDHYDTIINRRRLLSTDTGSQHCRLEGKVPLLSLEGLHHLHIFIFVLAVVHVVF
CVTTMILGGARIRQWKSWEDHARKKTTNSSGETLRSEIDEFFNKHAQGYWRKAAVVGWFRSFFKQFYGSVAKYDYLALRH
GFIKEHYPNDPNFNFHSYITRTLEVDFRKVIGISWYLWLFVVLFLLLNLEGWHTYFWLAFLPLIILLLVGAKLEHIITRL
GQESVAKEYPTERVKPSDEYFWFNRPAIVLDLLHFTLFQNSFEIAFFFWIWSTYGFDSCIMEKVAYIIPRIVMGVIVQVL
CSYSTLPLYTLVTQMGSRGKLDKVDDKAEMESSPLFHRMTKESNQHSQIGEQAIIMMEDHAISSTIELHPIIQSPLEKT

>MtMLO5 Medtr3g115960
MGGGGGGGDSRQLDVTPTWAVAAVCAIIVIISILLEKLIHKFASVFEERKKHALLEALEKIKAELMVLGFISLLLTFGQN
YISKVCIPVKYSNTMLPCQPLAERTADHPGEPALEPQGTEHEPTPNIPAGEGESKGEHHRRLLSYERRFLGGGGGGPGCK
PGFTPLISVDGLHQLHIFIFFLAVFHVIFSAITMTLGRAKIKGWKEWEQDHLVDEDALNDPRRFRLTHETSFVRDHNSFW
TKTPVTFYFVCFFRQFLRSVRRADYLTMRHGFVSVHLAPGSKFDFQKYIKRSLEDDFKVVVGISPILWASVVLFLLVNVH
GWHASFWVSFLPLAVILAVGTKLQAIITRMALDIQERHAVVQGIPLVQVSDKYFWFEWPQLVLYLLHYVLFQNAFELTFF

WWTWYEFGWASCFYEDDSLMIVRVALGVGAQFVCSYITLPLYALVTQMGSTMKRSIFDEQTSKALQNWRKNAMHKKPSKG
RSKETRTLGGGEAGSPVHSPLHDDGSETELQEHNTATIVTSVDRNDHYDNHDLLSGP

>MtMLO6 Medtr5g024700
MFKRVFSCLCLFYGGLSMAASDSRNSSRTLDQTPTWGVAAVCTVFILISIALEKSLHKLGTWLGKREKKALLEALEKVKA
ELMILGFISLLLTIGQNYIVRICISEKIANKMLPCPHKYIGGDKKSNSKVEQNRIPVSFEGRYLAADTPSFKCSRKGHEP
LLSVNGLHQLHILIFFLAVFHVFYSAVTMLLGRLKIRGWKEWEEETLTHGYEFANDAAKLRLTHETSFVRAHTSFWTRVY
IGCFFRQFYRSVRKVDYQTLRNGFINVHLAPGSKFNFQKYIKRSLEDDFKVVVGVSPVLWASAVVFLLLNIDGWRTPIWA
FLIPIIIILAVGTKLQVILARMALEISERHIVVQGMPLVQGSDRYFWLGQPQLVLHLIHFALFQNSFQITYILWTWYSFG
LENCFRLDYKLAIIKVAFGIVMLCLCSYITLPLYALVTQMGSRMKKSIFDEQTSKALKKWHMTVKKKHVKLGKSTIGTMD
GSTIVGSTMQSPGPALHRFKTTGHSTRSSTIDEQDEYELSDNELMETNLIVRVDHDEQEAEDRHHHSEGEANNDDFTFFK
HVIKRLV

>MtMLO7 Medtr2g006280
MEHAVEQGRSLAETPTYSVASVVTVMVFVCFLVERSIYRFGKWLKKTRRKALFASLEKIKEELMLLGLISLLLAQSARWI
SEICVNSSLFSSRFYTCSEQDLPMIEHVMIVNSSSFSDETTIPKGLYSGALHQCGEGHEPFVSYEGLEQLHRFLFVLGIT
HVLYSCLAVGLAMSKIYSWCRWENQATMALIDGNLQGKNNKVMRRQTTFVFHHASHPWSRNPILIWMLCFLRQFRSSIQK
SDYLALRLGFITEHKLPLSYDFHKYMVRSMEDEFHGILGISWPLWVYTIVCIFVNIHGLNIYFWLSFIPAILVMLIGTKL
QHVVSILALEIWQQQGPSARTQVKPRDGLFWFKKPDILLWIIQFVIFQNAFEMASFIWTLWGFKEKSCFMRHHYMIIIRL
TSGVLVQFWCSYMTVPLNVIVSQMGSRCKKALVTESVRDSLHSWCKRVKQKSKHDHSLHSHTARSICSLESTIDERDEIT
VVSGTLTRTTSLDLESLNQITVTSVDQLNFSNNLQDSTDLSEPVHINSHYNDVINGEEAKVETLLDLFHKT

>MtMLO8 Medtr3g115940
MAGGAPGERTLKETPTWAVAAVCAVFVIISVLIEHGIHKLEKWFHKKHKKAMSEALEKIKEELMLLGFISLLLTFGTKYI
AKICIPNKLGDSMLPCKKGEVEEESKDDRRRLLSFDFDENVVWRRGLAPAAGGDDYCSNKGQVPLISQSGVHQLHIFIFV
LAVFHIFYSVMTIVLARAKMQKWKAWETETSSVEYQFTHDPSRFRFAHQTTFVKRHSGWSEKPGIRWIVAFFRQFFASVS
KVDYMTMRHGFINAHFAPDSKFDFHKYIKRSMEDDFKVVVGISVPLWAFAIIFLLMNVYNWYTLTWLSLAPLVILLLVGT
KLELIIMEMAQEIQDRTTIVRGVPVVEPNNKYFWFNRPHWIIFLIHFTLFQNAFQIAYFLWTWYEFTITSCFHENLPLIV
TRVVLGVALQVLCSYITFPLYALVTQMGSHMKRGIFEEQTTKALKKWQKAARDKRKLRNAGSIEIPSVSGDTTPSQGTSP
LHLLHKFKPSNQTDTDSVLYSPRSYQSETDFSDTEGSTHQLNLNQIMSPPQHHPAGNNQQNHNIDFSFDKP

>MtMLO9 Medtr3g118190
MSGGGEGGEEENTLEFTPTWVVAGVCTVIVAISLAVERLLHYGGKFLKSKDQKSLYEALQKIKEELMLLGFISLLLTVSQ
NRLTKICVPPGVLRHMLPCSLEDKKESHSHSAFSFPGRIARRLLADRLLAESESAEEPLKTGFCGRKNKVPLLSVEALHH
LHIFIFVLAVVHVTFSVLTVVFGGARIRQWKHWEDSIAKENYDTNKVLKPKLTHVQQHEFIKGRFSGFGKDSALIGWLQS
FFKQFYGSVTKSDYVTLRLGFITTHCKTNPKFNFHKYMIRALEDDFKQVVGISWYLWLFVVIFLLLNINGWHTYFWIAFV
PVILLLSVGTKLEHVITQLAHEVAEKHAAIEGDLVVQPSDNHFWFHRPHIVLFLIHFILFQNAFEIAFFFWIWVTYGFDS
CIMGQVRYIVPRLVIGVFIQVLCSYSTLPLYAVVTQMGSHFKRAIFNDHVQVGIVGWAEKVKKKKALKADGQPSQGSSHI
HEGSTGSSTGIQLGSVFQKRASAPEDTTSVTKPEGSN

>MtMLO10 Medtr8g073130
MFMSKYKQRLFLCLVLFAWLYNGSVAMAASSEDSGSSKDLDKTPTWAVGCVCTVFILVSIILEKSLHKIGTWLGDRHKKA
LLEALEKVKAELMILGFLSLLLTFGQSYMVKLCMPANATDIMLPCQSRATRDAKNDEDEHHRKLLYYERRYLSEDGAPYN
CKEGQEPLISVNGLHQLHILIFFLAVLHVIYSAVTMLLGRLKIRGWKAWEAETSSHGYEFATDPSRFRLTHETSFVRQHA
TFWTKIPIFFYISCFFRQFYRSVEKSDYLTLRNGFIAVHLSPGSKFNFQKYIKRSLEDDFKVVVGVSPVLWASFVVFLVL
NVDGWHAMFWGSLIPVIIILAVGTKLQVTLSKMAIEITERHAVVQGIPLVQGSDKYFWFGKPQLLLHLIHFALFQNAFQI
TYFLWIWYSFGFINCFHADYKLAIVKVALGLVVLVLCSYITLPLYALVTQMGSTMKKSIFDDQTSKALKKWHNTVKKKHG
VKLGNSSVRTRDGSTTDSTVHSIGPTLHRHKTTGHSTRYENDQDDYQSDIELSPTSPTSNLLAVRVDNLEQDAKENEHQA
ILETIEQQQTVLRSLSFVKPDHPERSAN

>MtMLO11 Medtr4g079140
MAEGEQRSLEETPTWAVSVCCFFVLVISLIIEGGLHKLAEFLRKKKRKSMGKALTKTKTEMMKLGFVSFLLTISEVPMSK
ICINKDVANSFRPCKDSEYLAGEVSRKELGYHDNYCEAKGKVGLISSEGILELNILISILAVFHILYCTLTMCLGKAKMR
RWKKWEEETQTLEYQIDNDSRRFQYISQTLGGKRHFKFWNNYSPLLWIVCFMRQFYNSVSKDDYFALRNGFIAKNISEGS
NFNFNFKKFLSRTYDEDFEKVVGIRFWIWLFSIFFIFLSAHEFYNYYWLPFIPLVIALLAGTKLQVIITKMHLDSCKQSP
VIKGTILVKPSDEYFWFHKPEWLLHLIQIILIQNSFQLAFFTWSWYEFGPRSCFNREKEDIAIRIALGLAVQLLCAYVTL
PLYTLVTQMGSNMRKEIFTEHITQGLKNWHKKAKQSLSKSNNNSVSLHSRTSENSVRRSLSIESMQNNNKVENEDMQSVI
VVTSPPPGNNSGSGEEEEIVNVASVEQEISSESTSEIKNVEENNGKIITRGIYDGEISFGVYVGSSSRGIGEIVSIAEEE
DDDTSDQN

>MtMLO13 Medtr8g010500
MVEDKLKKDPICNMLLHGLLPLFAASSVAVSFAAECSLHCLGNFLKKKNQKPLYQALQKIKEELMLLGFISLFLTITQNG
ITKICVPDEWTYHMLPCSLEEIEEEEMKKNSHFQTFFSSDLVSGTARRLLGGGGGGDDPFGFTDEKVGYCTAKGKVPLLS
VEALHKLHIFIFVLAIVHVTSCVLTIIFGGLNIRRWKPWENSIVADENHESQHAPEWTDTVTHVHQHSFIKNRFTGFGKN

YAIMGWVKSFFKQFYGSVTKLDYVTLRRGFIMTHCRGNPKFNFHKYMIRALEDDFQNVVGISWYLWIFVVIFMLLNINGW
HTYFWVAFIPVILLLAVGTKLEHVIIQLAHEVAEKHSAIQGKLVVQPSDDHFWFHRPRIVLFLIHLILFQNSFEIAFFFW
ILVTFGFNSCMMGKLHYTIPRLVIGLLIQVICSYSTLPLYAIVTQMGTYYKKAIFDEQIQTCLLGWAQKAKKKGLRGNNN
QSGEGSTHSGASSNIQLGSVFRRAPAPEDNTIVPKDQQSV

>MtMLO14 Medtr8g010540
MGHGGGEAEEGRDLQFTPTWVVALVCTVIVAVSFAVERSLHYLGKVLKKKNQKPLFEALQKIKEELMILGFISLFLTVTQ
NGMIKICVPVEWTIHMLPCSINGDDEVKETKLPSKSHFQTFFSSKDVFGTAKRLLGDDHDHSSKDEEIGYCTAKGKVPLL
SVEALHKLHIFIFVLAVVHVTSCVLTIVFGGLNIRRWKRWEDSIVADENNQSQTPERTDTVTHVHQHAFIKDHFTGFGKD
SAIMGWLKSFFKQFYGSVTKLDYVTLRLGFIKTHCRGNPKFNFHKYMNRALEDDFKKVVGISWYLWIFVVIFLLLNVNGW
HTYFWIAFIPVILLLAVGTKLEHVIIQLAHEVAEKHSAIQGELIVQPSDDHFWFHRPRIVLFLIHLILFQNSFEIAFFFW
IWIQYGFNSCIMGQVKFIYPRLVMGVFIQVLCSYSTLPLYAIVTQMGTHFKRSIFDDQVQTRLIGWAQKAKKKGLRGDSN
HSVQGSSHNGGSTNIQLGSMFRRGPAPENNAFVPKNDESV

>MtMLO15 Medtr2g079500
MAETVKEPRSLALTPTWSVATVLTIFVAVSLLVERSINRLSNWLRKTNRKPLLEAVEKMKEELMLLGFISLLLTATSGMI
ANICIPSKFYNSAFAPCSRSEINKEIEENGSEGRKLLTASYPRLIRRMLNGMDRNSCKEGYEPFVSYEGLEQLHRFIFVM
AITHVSYSCLTMLLAIVKIHSWRVWEDEAHMDRRNYLSEITRQMTMRRQSTFVKTHTSNSLFIWMTCFFRQFGHSVVRAD
YLTLRKGFITNHNLSSKYDFHSYMVKSMEEEFQRIVGVSGPLWGFVVGFMLFNIKGSNLYFWIAIIPISLVLLVGTKLQQ
VIATLALENAGISGFFSEAKLRPRDELFWFKKPELLLSLIHFILFQNAFELASFFWFWWQFGYNSCFIRNHLLVYLRLIL
GFAGQFLCSYSTLPLYALVTQMGTNYKAALIPQRIRETIHGWGKAARKKRRHGMFADDSTIHTDTSTVLSIEEDYQTIGD
PHKDLVTGTEVELQPVPTVTSTPSPIANETSSRAVTPLLRPSASVSSSVPFNSRQEEPIPRSSSMPSGR

>PsMLO1
MAEEGVKERTLEETPTWAVAVVCLVLLAVSILIEHIIHVIGKWLKKRNKNALYEALEKIKGELMLLGFISLLLTVFQDNI
SKICVSQKIGSTWHPCSTSNTKAKAKSDESLDYKTNNDRKLLEYFDPIPRRILATKGYDKCFDKGQVALVSAYGIHQLHI
FIFVLALFHILQCIITLTLGRIKMRKWKTWEDETRTVEYQFYNDPERFRFARDTTFGRRHLSMWAQSPILLWIVSFFRQF
FGSISRVDYMALRHGFIMAHLPPGHDAQFDFQKYISRSIEEDFKVVVGISPTIWLFTVLFLLTNTHGWYSYYWLPFLPLI
VILLVGAKLQMIITKMGLRIQDRGEVIKGAPVVEPGDHLFWFNRPHLLLFTIHLVLFQNAFQLAFFAWSTYEFSITSCFH
KTTADSVIRITVGVVIQTLCSYVTLPLYALVTQMGSTMKPTIFNERVATALKNWHHTAKKQVKQSNHSNNTTPYSSRPST
PTHAMSPVHLLHRHTAGNSDSLQTSPEKSDYKNEQWDIEGEGPTSLRNDQTGQHEIQIAGVESFSSTELPVRIRHESTSG
SKDFSFEKRHLGSN

>PsMLO2
MSGGGEGGEDETTLEFTPTWVVAAVCTIIVAISLAAERLLHYGGKYLKRKDQKPLYEALQKIKEELMLLGFISLLLTVSQ
NGLTKICVPPGVLRHMLPCTLKEKDQKESELPKSHSAFSFPGIARRLLADRLLAEAEAAEEAHPKTSFCSSKSKVPLLSL
EALHHLHIFIFVLAVVHVTFSVLTVVFGGARIRQWKRWEDSIRKENYDTSKVLKPKFTQVQNHEFIKGRFSGFGKDSALI
GWLQSFFKQFYGSVTKSDYVTLRLGFIMTHCKTNPKFDFHKYMIRALEDDFKIVVGISWYLWLFVVIFLLLNINGWHTYF
WIAFIPVILLLSVGTKLEHVITQLAHEVAEKHAAIEGDLVVKPSDGHFWFHRPHIVLFLIHFILFQNAFEIAFFFWIWVT
YGFDSCIMGQVRYIVPRLVIGVFIQVLCSYSTLPLYAVVTQMGTHYKRAIFNEHVQVGLVGWAEKVKKKKALKAESQGSS
HDQEGSSTSAGIQLGSVFQKKASTPEDNASVPKAEGSN

>PsMLO3
MATQTEQRSLEDTPTWAVSICCFFVLVISLIIEHGLHKFTEVLKKQKRKSMGKALAKTKIEMMKFGFVSFLLTISEVPIS
KICINKDMANSFLPCKDSMEKLAVSSELDQTPSTNELGNQVNYCEAKGMVSLISDDGVLQLNVLISILAVFHILYCTLTM
CLGMAKMRRWKKWEEETQTLEYQIDNDSRRFQYISQTLGGKRHFKFWNNYSPLLWIVCFMRQFYNSVSKDDYFALRNGFI
AANISEGSNFNFNFKKFLSRTYDEDFEKIVGIRFWIWLFSIFFIFLSAHEFYNYYWLPFIPLVIALLAGTKLQVIITKMH
LDSCKESSVIKGTILVKPSDEYFWFHRPEWLLHLLQIILIQNSFQLAFFHWTWYEFGPRSCFNREIEDIAIRIVLGLAVQ
LLCAYVTLPLYALVTQMGSNMRKEIFTEHVNQGLKNWHKKAKLSLSKNSNSASLHSKTSENSVRGSLEELRNNEDIITPN
SANNSGSGEEKEVIVAAVEKXIPSESTSEENGKIITRGIYDGEISFGIYVGSSSRGIGEIVAIAEEEDDYTHHKFESRGI
GLVDCNIYLNKTHSLN
NVYNL

>PsMLO4
MAENGKETRSLALTPTWSVATVLTIFVAVSLLVERSINRLSNWLRKTNRKPLLEALEKMKEELMLLGFISLLLTATSGMI
ANICIPSKFYNSAFAPCTRSDIDKEIEENGSEGRKLLTASSFPRLIRRMLNGINRSSCKEGYEPFVSYEGLEQLHRFIFV
MAITHVSYSCLTMLLAIVKIHSWRVWEDEAHMDRRNYLADITRQLTMRRQSTFVKPHTSNPLAIWMTCFYRQFGRSVVRA
DYLTLRKGFIMNHNLSSKYDFHSYMVRSMEEEFQKIVGVSGPLWGFVVGFMLFNIKGSNLYFWIAIIPISLVLLVGTKLQ
QVIATLALENAGIAGFFSEAKLRPRDELFWFKKPELLLSIIHFILFQNAFELASFFWFWWQFGYNSCFIRNHLLVYLRLI
LGFAGQFLCSYSTLPLYALVTQMGTNYKAALIPQRIRETIHGWGKAARKKRRHGMFPDDSTFHTDTSTVLSIEEDDHRII
DDSLQDIATGVEVELQPVTAVTATPSPIANETSSRGITPLLRPSASVSSSVPFSARQEPVSRSSSMPNGR

>PvMLO1 Phvul.001G175900

MNMATAPGYSLQHTPTWAIATVSFILISISIILEHLIHLIIKWLRKHRKSDLVEAIERLQSELMILGFMSLLLTITQDAI
IEICIPVRAADTMLPCRKRTTTNDTAILDSCSAKNASEVALVSKHGIHQLHMFIFVLAVMQIVYSFLTVSLARAKMKNWK
AWDEETQTMEYEIANDPNRFRYARQTTFGRRHISTSTASPVFVWIKCFFRQFFHSVEKVDYLTLRHGFISAHFSARNSNF
DFQAYIEQSLEEDFRIIVSISPVMWFTVVIFLLVDVHGWHVYLWLSYVPLFLVLVVGAKLEVIVDEMALQMKDVNSVTKG
TPLVCPSDDYFWFGHPGFVLTLLHYTLFVNAFELAFFVWVSTQFGINSCYHEHRTFTIIRVVIAVAVQVLCSYVTLPLYA
LVAQMGSEVKSKALAKILRQWHVEVRERRRKQLKLVKSFSFRRMNMSSEWSQGNKSAPEFSSELRENTRSSNEGEIVEEL
EHRVKTEATSSNDPPSVV

>PvMLO2 Phvul.002G107600
MGKENGEGRSLAETPTYAVATVIAVLVSLSFLFQGTLKKLVKWLDRTKRKSLLSALEKIKEELMLFGLLSLLMGHWIIFV
AKICVKSSVLSSRFFPCALEKNSVKDVVWLSSEYSNRTVVKGQVNNGLRNYCPEGRESLASYESLEQLHRFVFVLGITHV
TYSFLAVALALIKIYSWRTWENEAKTIAVQSIQDTSQSTSSIRLRRLNTFIFHHTSHPWSHHKILVWLLCFSRQFWSSIH
RADYMALRLGFITNHDLPTTYDFHNYMLRSMDEEFRDIVGVSVLLWVYAICCIFLNFHGSNFYFWLSFVPAVLILIIGTK
LHRVVVKLAVEIINRCPDMKPNQFNLRDELFWFGKPRFLLRLIQLISFLNAFEMATFLWSLWEIKDPSCFMNNRTFIVIR
LSFGVISQVWCSFITFPLYVIITQMGSRFKKTVVSENVRKSLSKWQRRVKGRQSYSSALLTATSPTSISSFIYGNDRASI
SEEGSSSGTKDICLSYLDHESRTQANASFPSYENDHNDQDSHDIIHHSSPPPLS

>PvMLO3 Phvul.002G117900
MNSSKFRKLLSCVVVVLPWWLLWSGGSAMAASESGSSSKELDQTPTWAVAVVCTVFILISITLEKSLHKVGTWLGKKQKK
ALLEALEKVKAELMILGFISLLLTFGQSYIVKICIPEKMADKMLPCPYKYKDAEKASNDDGEKHRRKLLSYERRYLAADS
ISFKCSNEGYEPLLSVNGLHQLHILIFFLAVLHVLYSAITMLLGRLKIRGWKAWEAETSTHNYEFATAASRFRLTHETSF
VRAHADLWTRIPIFFYIGCFFRQFYRSVNKTDYLTLRNGFITVHLAPGSKFNFQKYIKRSFEDDFKVVVGVSPILWASVV
VYLLINVNGWRTTLWVSVIPVVIILAVGTKLQAILAKMAIEITERHAVVQGMPLVQGSDKYFWFGQPQLVLHLIHFALFQ
NAFQVTYILWIWYSFGVRNCFRNDYKLATIKVALGIVMLCLCSYITLPLYALLTQMGSRMKTAIFDEQTNKALKKWHMAV
KKKQRVVKLGKSNAESVYGSPTASTSTVHPSGPTLHRYKTTGHSTRSSTYEDQDHEYESDVELSPELSQTTSFIVRMNHA
DHQQLQEHEHRTEGENNSSEESDFSFVKVKPSDPVEITTTPHGI

>PvMLO4 Phvul.003G271300
MSVSKCKRLSLCLVSWLWFCCVSVASSESSIGSKDLDQTPTWAVACVCTVFILISLTLEKSLHKVGTWLQEKHKKALLEA
LEKVKAELMILGFLSLLLTFGQSYIVRICIPTDVADKLLPCPLVGTDRESSSEEEHRRKLLSYERRYLSDDATPYQCKKG
DHQPLISVNGLHQLHILIFFLAVLHVFYSAITMLLGRLKIRGWKAWEEETSSHGYEFANDPSRFRLTHETSFVRAHASFW
TRYSIFFYIGCFFRQFYRSVGKADYLALRNGFITVHLAPGSKFNFQKYIKRSLEDDFKVVVGVSPVLWASFVVFLLLNVN
GWHAMFWASLIPVVIILAVGTKLQVTLAKMAIEITERHAVVQGIPLVQGSDRYFWFGRPQLVLHLIHFALFQNAFQITYF
LWIWYSFGLRNCFHADYKLAIVKVALGLAALCLCSYITLPLYALVTQMGSRMKKSIFDEQTSKAIKKWRMAVKNKQGVKL
GNSKVRAMDGSTTDSTVHSSGPTLHRFKTTGHSTRTMSAYDDDQDYHSDIELSPISPTANLIVRVDHDEHQAEKAQHHPT
PNNQDLSRLSTLETSMK

>PvMLO5 Phvul.004G071700
MANEVYERTLEETPTWAVAVVCFLLLAVSIAIEHLIHGIGKWFKKKQKNALFEALEKVKGELMVLGFISLLLSVLQDQIS
KICVSEEVAASWHPCANPKSSTATKTDEDESDDIQINSRKLLQLYDSIPRRILATKGYDKCAEKGKVAFVSAYGIHQLHI
FIFVLAIFHILQCIITLGLGRTKMRKWRAWENETKTIEYQFYNDPERFRFARDTTFGRRHLNPWSQSTISLWIVSFFRQF
FGSATKVDYLTLRHGFITAHLAPGSDARFDFQKYIQRSLEKDFKVVVGISPVIWFFAVLFLLTNTHGWYSSYWLPFIPFV
VILLVGAKLQMIITKMGLRITDRGEVVKGAPLVEPGDDLFWFNRPRLLLFLIHLVLFQNAFQLAFFSWSTYEFSLDSCFH
ETTADVAIRLTMGVVIQVLCSYVTLPLYALVTQMGSTMKPTIFNQRVASALKNWHSTAKKQVKNSKQNTPFSSKPSTPTY
GMSPMHLLQKHLAGRSDSAQTSPRTSNYENEQWDVEGSPSTSNHAAAEETQMQVMEPGSSSAPEFPTSSQIEIRVSSSEF
SFEKRHTGASEEI

>PvMLO6 Phvul.004G071800
MGEAKIHKRSLEETPTWAFAVVCFMLLAISIIIELVIHAIGKWLRKKHKSGLYESLEKVKGELMMLGFLSMLLTVFQDPL
SKICISQNAASTWHPCSNPKAVSNSNATSHTIDRKLLHLDPIPRRVLAAKGYDKCADKGKVAFVSAYGIHQLHIFIFVLA
IFHIIQCIVTLALGRTKMRRWKKWEDETKTIEYQFYNDSKRFRLAKDTTFGQRHLNTWSQSSISLWIVSFFRQFFGSVKK
VDYFVLRHGFIMAHVSPRSDSGFDFQRYIKRSLDEDFKVVVGISPIIWFFAVLFLLTNTHGWYSNYWLPFIPLITILIVG
ADLQMIITKMGLRIQDRGVVMGAPVVEPGDDLFWFNRPRLLLFIIHLVLFQNAFQLAHFAWSTYEFSIESCFHKTNAENV
VRLTMGVVTQVLCSYVTLPLYALVTQMGSTLKPTIFNDRVAAALKKWHHTSKKHLKHSQHSEAKKSIPFSSTSSSPIFGM
SPIHLLHRHPVGRSDSAQTSPRTSLYENQQCDVQGESSVSNHPGTNETQMQVLGSPQTT

>PvMLO7 Phvul.005G082500
MEKNVQETRSLALTPTWSVATVLTVFVAVSLLVERSIHRLSNWLRKTNRKPLLAALEKMKEELMLLGFISLLLTATSRMI
ANICIPSKFYNSAFAPCTHSQIDEQMEENGSEERKLLTASAYHVFGRMLDGINRSTCKEGYEPFVSYEGLEQLHRFIFVM
AVTHISYSCLTMLLAIVKIHSWRVWEDEAHMDRRNSLSEISRELTMRRQSTFVKSHASNPLFKNSTLIWVTCFFRQFAHS
VVRADYLTLRKGFIMNHHLSLKYDFHSYMVRSMEEEFQRIVGVSGPLWGFVVGFMLFNIKGSNLYFWIAIIPVSLVLLVG
TKLQHVIATLALENAGIARFFPEAKLRPRDELFWFNKPELLLSLIHFILFQNAFELASFFWFWWQFGYDSCFIRNHLLLY
LRLILGFAGQFLCSYSTLPLYALVTQMGTNYKAALIPQRIRETIHGWGKAARKKRRHGMFPDDSTIHTDTSTVLSIEEDD
QVIDAPEVELPPVTAVTPTPSPIANETSSRAVTPLLRPSASISSVHSSSKAEVIIRCSSMPNGS

>PvMLO8 Phvul.005G136700
MGGRSLEETPTWAVAVVCFVLLTISIVIDHIFHLIGKWLKKKHKRALYESLEKIKSELMLLGFISLLLTVGQSVISRICI
SEKVAGTFHPCTHKTDHTRRLLPELFRSDHNQNLRRILAAGGTDKCAAQGKVPFVSSEGIHQLHIFIFVLAVFHVLYCIL
TLALGRAKMRRWKRWEEETKTAQYQFSHDPERFRFARETSFGRRHLSFWTQHTILVWIICFFRQFVRSVPKVDYLTLRHG
FMMAHLGPQSRHKFDFRQYIKRSLEEDFKVVVEISPPIWFITVLFLLFHTHGWYSYLWLPFAPLIIVLLVGTKLQVIITK
MGQRIQERGEVVKGMPLVQPGDDLFWFNKPRLILYLVNFVLFQNAFQLAYFSWTAVQFGMKSCFHSQTEDVVIKISMGVF
VQFLCSYVTLPLYALVTQMGSTMKPTIFNERVAMALRNWHHAAKKHVKQQNRRLQSRPTTPNHSTSQAHLLRRCHSEIDI
YPTDSEAHHPYEMDLSPSSKVHDHEVEMEMGHLDHGPLEDVNELNSALAGSGVTQHEINIEHGKEFSFDNR

>PvMLO9 Phvul.005G136800
MGGNGGRNLDETPTWAVAVVCFVLLSVSIIIEYIFNLIGKWLKQKHKRALYESLEKIKSELMLLGFISLLLTVGQGLISR
ICISEKVAGTFHPCAHRRVKQNNTPPLEHDEYRNNRHLLALEDKCAPQGKVPFVSSEAIHQLHIFIFVLAVFHVLYCILT
HALGRAKMRRWKRWEVETKTPEYQVSHDPQRFRFARETSFGRRHLSFWTQNAVLVWIICFFRQFVHSVPKVDYLTLRHGF
MMAHLGPHSHQKFDFGKYIKRSLEEDFKVVVEISPPIWFITVLFLLFNTHGWYSYLWLPFAPLIIVLLVGTKLQVIITKM
GQRIQERGEVVKGMPVVQPGDHLFWFNKPRLILYLINFVLFQNAFQLAFFSWAALQFMMKSCFQSQKQDVVIRISMGIFV
QFLCSYVTLPLYALVTQMGSTMKATIFNEKVAMALRNWHHTAKKNVKEKRGVRSQSPLSTRPSTPQQQPKSQANLLRRYH
SEMATYPSSPIRFDFEAHLPYGIHSPPSSMVNAAATSSIHRHEMEMETEIEMDQVSALTQRQIDIDIDIQDNQEFSFAKR

>PvMLO10 Phvul.006G133800
MAEELNDSLEYTPTWIVAVVCSIIVFISLCVERALHKLGKYLRSKNQNELYEVLTKLEEELMLLGFISLLLTVFQGLISD
ICISPNLATQMLPCKRPHRSSQGSEHYQIYYDSIINRRRLFSTGSGSDHCTHKGKVPLLSLESLHQLHIFIFVLAVVHAI
FCVTTMLLGGAKIREWKAWEDYYGDHREELPNSNAVIDTHEFFKTRDRGHWRKAAVISWLISFFKQFHGSVTKYDYYALR
HGFVKKHYPDNPTYNFHNYMLGTLEEDFKRVVGISWYLWVFVVLFLLLNIGGWHTYFWLAFLPLILLLLVGAKLEHIIAR
LYQDSIGVLGREDDNSMKPSDEYFWFTSPPLVLHLLHFILFQNSFEIAFFFWIWCTYGFDSCIMEKVTYVIPRLIMGVIV
QVLCSYSTLPLYTIVTQMGSKIKSQKSPPSDPLKTPHSNYMIKESTQIDEQAIIMVEDATSSIELPHIVHLPFEKPNTSN
N

>PvMLO11 Phvul.006G216900
MAEVLQRGRSLAETPTYSVASVVAVMVFVCLLVERSIYRFGEWLKKTRRKALFASLEKLKEELMLLGLISLLLAQSARWI
SEICVTSSLFSSRFYICSEQDVDINENIMLESSSSSIPDETSITGAFNQCGEGHEPFVSYEGLEQLHRFLFVLGITHVLY
SCLAVGLAMSKIYSWRRWENQARMPSDGNQQAKKIKLMKRQTTFIFHRTSHPWSRSPILNWMLCFLRQFRSSIRKSDYLA
LRLGFITEHNLPLSYNFHQYMVRSMEDEFHGILGISWLLWIYAIVCIFVNIHGLNIYFWLSFIPAILVMLIGAKLQHVVS
TLALEIMEQTGPFPRTQVKPRDDLFWFKKPDILLWLIQFVIFQNAFEMATFIWTLWGFQEQSCFMRNHYMIIIRLASGAI
VQFWCSYMTVPLSIIVSQMGSKCKKALVTESVRESLHSWCKRVKHKSKHDSLHSHTARSVCSLESTIDERDEMTVASGTL
TRSSSLGSLNQITVSSVEQLKLETPNKAKHSR

>PvMLO12 Phvul.007G093600
MGKEEVLQRKLEATPTWVVAAVCFVMLAISIIIEHVIEELGKWLKKKHKKALHEALEKIKGELMLLGFLSFLLVVFEDRI
VSICIPESVASTWNPCDPSYVSKGAEGYVEKCAKKKEGTVAFMSKYGLHQLHIFVFVLAIFHILQCIMTLTLGRTKISIW
RKWEDETKGLEHQFSHDPERFRFARDTTFGRRHLNSWSKSPTLLWIVSFCRQFYGSLNKADYMALRHGFVVAHLTPASEP
KFDFQNYITRTLDEDFAVVVGISPTIWFFAVVILLTNTHGWYSYFWVPFIPLIIILLVGTKLQMIITDMALKIQDRGEVV
KGEPVVQPGDELFWFNNPRLVLFLIHLVLFLNAFQLAFFTWSTLDNGFKINSCFHRTTADIVIRLTMGVLTQVLCSYVTL
PLYALVIQMGSSMKPTIFKDNVASALKSWHHSAKKHLKHNRDSTSNTPFSSRPGTPTRGMSPVHLLHKHPRHSDSPLVSP
RASDYENGQWDAEVSHSPSHHARDIDHEETMQVQPPAAAELPPAGLNPVRTQHEINIALSEFSFGRGRNPGRADEY

>PvMLO13 Phvul.009G006800
MAGGASGGERTLQETPTWAVASVCAVFVVLSVLIEHGIHLLEKWFQKRNKKAMREALEKIKAELMLLGFISLLITFGTKY
IAKICIPTSAGDIMLPCKKVEESKNSDDSNGRKLLYFEDNMEWRRVLATASSGGDFCSQKGKVSLISQSGVHQLHIFIFV
LAVFHILYSVMTMVLARAKMKKWKAWEAETSSLEYQFTNDPSRFRFAHQTSFVRRHSGLSRMPGIRWIVAFFRQFFASVT
KVDYMTMRHGFINAHFAPNSKFDFQKYIKRSMEDDFKVVVGISIPLWVFAIVFMLVNVYKWYTLTWLSLAPLVILLLVGT
KLELIIMEMAQEIQDRTTVVRGVPIVEPNNKYFWFNRPQWIIFLIHFTLFENAFQIAFFLWTWYEFKITSCFHENLPLIL
TRVFLGIALQVVCSYITFPLYSLVTQMGSHMKQAIFEEQTAKALKKWQKVAKDKRKLRKAGVDVPSGTMSGEATPSQGTS
PIHLLHKYKPSQTDTDVVLYSPRSYQSDTDFSDTEGSSHQLNQITQTHQAPRNGEAHSIEFSFVSHNNVQF

>PvMLO14 Phvul.009G007000
MSGGGGGDSRQLDLTPTWAVAAVCAIIVLISILLEKIIHKFARMFEEKKKHALLEALEKIKAELMVLGFISLLLTFGQNY
ISKVCIPQKYAHTMLPCLPIDQRDGGGPATEHGAGAATEHGAGAATEHGAGAATEHEVEPEGEGGESAEGGGHRRRILSY
DRRFLSGGGGGPSCKPGKLPLISVNGLHQLHIFIFFLAVFHVIYSAITMTLGRAKIRGWKAWEEDHVVDEDALNDPRRFR
LTHETSFVRDHSSFWTKTPVSFYFVCFFRQFFRSVRRADYLTMRHGFVTVHLAPGSKFDFQKYIKRSLEDDFKVVVGISP
ILWGSVVLFLLVNVHGWHAAFWVSFLPLVVILAVGTKLQAIITRMALDIKERHAVVQGIPLVQVSDKYFWFAWPQLVLYL
IHYVLFQNAFELTYFWWTWYEFGWASCFYEDDRLMIIRVALGLGAQVVCSYVTLPLYALVTQMGSTMKKSIFDDQTSRAL
KQWHKNALKKKASKGRHETRTLGGSPGEHTPEHSPHPPPPRDTEMAAQTATIVTSVDNETYDKRDLLTGP

>PvMLO15 Phvul.009G117900
MSGGGGEEGDSLEFTPTWVVAAVCTVIVAISLAAERFLHYGGKFLKAKDQKPLYEALQKIKEELMLLGFISLLLTVTQNG
ITKICVPPGFTRHMLPCSLDLAPNHESHFQTFYFPGTPRRLLAATAEEHAPPHSKAETETATGFCSRKNKVPLLSVEALH
HLHIFIFVLAVVHVTFSALTVIFGGARIRQWKHWEDSIAKQNYATDQVLKPKVTNVQQHDFIRDRFSGFGKDSAIVGWLI
SFSKQFYGSVTKSDYVTLRHGFIMTHCRTNPKFNFHKYMIRALEDDFKKVVGISWYLWLFVVIFLLLNINGWHTYFWIAF
IPVILLLSVGTKLEHVITQLAHDVAEKHAAIEGDLVVQPSDEHFWFHRPRVVLFLIHFILFQNAFEIAFFFWIWVTYGFD
SCIMGQVRYIIPRLVIGVFIQVLCSYSTLPLYALVTQMGTHYKRAIFNDHLQQNIVGWAQKAKRRKEQNADGNPGQGSSQ
ENGNTGIQLGSIFPKKASGPGNSSSAPKADRTS

>PvMLO16 Phvul.010G034400
MGGGGGEEGNNLEFTPTWVVAVVCSVIVSASFAAERFLHYGGTFLKKKNQKPLFEALLKIKEELMLLGFISLLLTVTQNG
IIKICVPESWTRHMLPCSLKDKEELESAKLTSHFQTFFSFTDIPATVRHLLAENENEDHQSGEKLGHCAKKGRVPLLSVE
ALHHLHIFIFVLAIVHVTFCVLTVVFGGLKIRQWKHWENSIVDENNRKQPVLESIVTHVHEHAFIQNHFTGFGKDYAVLG
WLKSFFKQFYGSVTKLDYVTLRLGFIMTHCRGNPKFNFHKYMIRALEDDFKQVVGISWYLWIFVVIFMLLNVHGWHTYFW
ISFIPLILLLAVGTKLEHVIIQLAHEVAEKHSAIEGELVVQPRDDHFWFHRPHIVLFLIHFILFQNAFEIAFFFWILFTY
GFDSCIMGRVRYIVPRLVIGIFIQVLCSYSTLPLYAIVTQMGTHFKKAIFDDQVQARLVGWAQKAKKKGLRGDNNGQSVQ
GSPHVGAGIQLGPVFRKPSPPEDTVLP

>PvMLO17 Phvul.011G073800
MAGGGGRNLEETPTWAVSTVCFVLIFISIIIEHIIHLIGQWLKKKRKRALYESLEKIKSELMLLGFLSLLLTVGQGLVSR
ICISEKIASTWHPCSADDTHSEESGQGTNSRRLLAAFHGSNDVNPRRVLAGGGSDKCGEGKVPFVSSEGIHQLHIFIFVL
TVFHVLYCILTMALGRAKMKRWKRWEEETKTPEYQFSHDPERFRFARETSFGRRHLSFWTKNPVLMWIVCFFRQFVRSVP
KVDYLTLRHGFIMAHLAPQSHSKFDFRKYIKRSLDEDFKVVVGISPPFWLFAVLFLLLNTHGSYSYLWLPFIPLIIILLV
GTKLQVIITEMGLRIQQRGEILKGVPLVQPGDYLFWFNKPGLILYLINFVLFQNAFQLAFFSWSVLQFGIKSCFHAHTED
VVIRITMGVLIQILCSYVTLPLYALVTQMGSTMKPTIFNERVAEALRNWHQTAKKQIRQNRVGPLSLSVTPMSSRPTTPS
HNMSPVHLLRYYRSEIDSFPTSPRRSNVDGDHTQPWDVESPSPSYSHHEMEMGHTNDPTTNTTHHEIVPAANTREFSFDK
RPTTAP

>PvMLO18 Phvul.011G122800
MDMEEQRSLEETSTWAVSVFCFFFLLTSLIIEGSLHKLAEILRKRKDKSLGRALTKTKTEMMKFGFMSFLLTISEVPISK
ICISKGVATYFLPCKDAVDLTGLSATRTSTSELDVAHATNQSNVEFNYCEAKGMVSLISSEGILQLNIFISFLAVFHIFF
CTLTMCLGKAKMRRWKRWEEETRTLEYQIANDPRRFQYTGQTMIGKRHLNFWSYHSPLLWMVCFIRQFYGSVSKDDYYTL
RNGFIAANISLGSNFNFKKFLSRTYDEDFEKVMGIRIWIWIFSILFIFFSAHEFYNYYWLPFIPLMVALLAGTKLQVIIT
KMCVDSCKANPVIKGSLLVTPSDAHFWFHRPEWLLYLLKFILIQNSFQLAFFTWTWYEFGPRSCFNRENEDIGIRIAMGI
AVQLLCGYVTLPLYALVTQMGSSMRREIFTERISRGLKNWHKKAKQTLHKNNSTSSKHSDSLHSKGGDNSARGTMQSVHT
PDNVAVTNNPSPSNEGKRIASTNNEQDTSSPSTSEITATEEENPKIITRGTYDGEISFGSSWKNVGSSRGIREIGSITEE
DDAEKLPELIP

>PvMLO19 Phvul.011G172300
MEKNVQETRSLALTPTWSVATVLTVFVAVSLLVERSIHRLSNWLRKTNRKPLLAAIEKMKEELMLLGFISLLLTATSRMI
ANICIPSKFYNSAFAPCTRSQIDEQMEENGSEERKLLMASAYHVFGRMLDGINRSTCKEGYEPFVSYEGLEQLHRFIFVM
AVTHISYSCLTMLLAIVKIHSWRVWEDEAHMDRRNSLSEITRELTMRRQSTFVKSHASNPLFKNSSLIWVTCFFRQFAHS
VVRVDYLTLRKGFIMNHHLSLKYDFHSYMVRSMEEEFQRIVGVSGPLWGFVVGFMLFNIKGSNLYFWIAIIPVSLVLLVG
TKLQHVIATLALENAGIAGFFPEAKLRPRDELFWFNKPELLLSLIHFILFQNAFELASFFWFWWQFGYDSCFIRNHLLLY
LRLILGFAGQFLCSYSTLPLYALVTQMGTNYKAALIPQRIRETIHGWGKAARKKRRHGMFPDDSVTIHTDTSTVLSIEED
DQLIDAPEVELQPVTAVTSTPSPIANETSSRVVTPLLRPSASISSVHSSSKAEVIIRCSSMPSGS

>VrMLO1 JJMO01000124
MASKVYERTLEETPTWAVAVVCFVLLAVSIAIEHLIHGIGKWFKKKRKNALFEALEKVKGELMVLGFISLLLSVLQDQIS
KICVSKHVASTWHPCENPKSSTTTSEDESEDIQINYRKLLQLYDSIPRRVLATKGYDKCAEKGKVAFVSAYGIHQLHIFI
FVLAIFHILQCIITLGLGRTKMRKWRAWENETKTIEYQFYNDPERFRFARDTTFGRRHLSPWSQSTISLSIVSFFRQFFG
SVNKVDYLTLRHGFITAHLAPGSDARFDFQKYIERSLEEDFKTVVGISPVIWFFAVLFLLTNTHGWYSYYWLPFIPFIII
LIVGAKLQMIITKMGLRITDRGEVVKGAPLVEPGDDLFWFNRPRLLLFLIHLVLFQNAFQLAFFSWSTYEFSLHSCFHET
TADIVIRLTMGVIIQVLCSYVTLPLYALVTQMGSTMKPTIFNQRVASALKNWHNTAKKQVKSSKPTTPLSSRPSTPTYGM
SPMHLLQKHLAGRSDSAQTSPRTSNYENEQWDIEGSPSKSNHAAGEETQMQVMESGSSSAPELPTSSQLEIRVSSSEFSF
EKRHLGAETRSEDDSFY

>VrMLO2 JJMO01000125
MAGGGGGRNLEETPTWAVSTVCFVLILISIIIEHIIHLIGKWLKKKHKRALYESLEKIKSELMLLGFLSLLLTVGQGLIS
RICISEKVASTWHPCSHENTDTTEESEQGTNSRRLLAAFHGSNEVTSRRVLAGGGSDKCGEGKVPFVSSDGIHQLHIFIF
VLTVFHVSYCITTMALGRAKMKRWKRWEEETKTPEYQFSHDPERFRFARETSFGRRHLSFWTKNPVLMWIVCFFRQFVRS
VPKVDYLTLRHGFIMAHLAPQSHSKFDFRKYIKRSLDEDFKVVVGISPPFWFFAVLFLLLNTHGSYSYLWLPFIPLIIIL
LVGTKLQVIITEMGLRIQQRGEILKGVPLVQPGDYLFWFNRPALILYLINFVLFQNAFQLAFFSWSVLQFGIKSCFHAHT
EDVVIRISMGVLIQILCSYVTLPLYALVTQMGSTMKPTIFNERVAEALRKWHQTAKKQIRQNRVGSLSLSGTPMSSRPTT

PSHHMSPMHLLRYYRSEIDSFPASPRRSNFDGDHTQPWEIDSPSPSYSQHEVEMGPTNDPNTNTSQHEIVTIAHTKEFSF
DNRPTTAT

>VrMLO3 JJMO01000125
MLTHHNYSFVFSFLKIFGRKCRKLRNQRLVLMEIGEERSLEETATWAVSVFCFFLLLISLIIEGSLHKLSEILRKRKGKS
LGRALTKTKTEMMKFGFMSFLLTIMSEVPISKICINKDVATYFLPCKDAAELTGFSATHTSTSQFDVAPATNESEVEVNY
CEAKGMVSLISSDGILQLNIFISFLAVFHILFCTLTMCLGKAKMRRWKRWEEETRTLEYQIANDPRRFQYTGQTMIGKRH
LNFWSYHSPLLWMVCFVRQFYVSVSKDDYYTLRNGFIAANISLGSNFNFKKFLSRTYDEDFEKVMGIRIWIWIFSILFIF
FSAHEFYNYYWLPFIPLMVALLAGTKLQVIITKMCVDSCKANPVIKGSLLVTPSDAHFWFHRPEWLLHLLKFILIQNSFQ
LAFFTWTWYEFGPRSCFNRENEDIGIRIGMGIAVQLLCGYVTLPLFALVTQMGSSMRREIFTESVSMGLKNWHKRAKQSL
QNNSTSRKHSDSLHSKGGDNSARGTMHSLNNPDIVVLTNNPSPSNEGESIAPTNNEQDVSSPSTSEITATEEENSKIITR
GTYDGEISFGSSWKNVGSSRGIGEIVSITEEDDVEKLPEFIHR

>VrMLO4 JJMO01000126
MATPSYSLQHTPTWAIALVSFILISISIILEHFIHLIIKWLRKHRKSDLVEAIERLKSELMILGFMSLLLTVTQDAIIEI
CIPVRAADTMLPCRKRTANDTAILDSCSAKSASEVALVSKHGIHQLHMFIFVLAVMQIVYSILTVSLARAKIRHWKAWDE
ETQTIEYEIANDPNRFRYARQTTFGRRHISTTTASPVSVWIKCFFRQFFHSVEKVDYLTLRHGFISAHFSARNSNFDFQK
YIEQSLEEDFRIIVSISPVMWFTVVIFLLVDVHGWHVYLWLSYIPLLLVLVVGAKLEVIVDEMALQMKDVNSVTKGTPLV
SPSDEFFWFGHPGFVLTLLHYTLFVNAFELAFFIWVSTQFGINSCYHEHRTFTVIRVVIAVAVQVLCSYVTLPLYALVAQ
MGSQVKSKALAKILRQWHVEVRERRRKQQKLVKSFSFSHMSSEWNQGNKSALEFSSGLRENARSSSEGEIVEALEHPMIT
QANSSSDPPPSVV

>VrMLO5 JJMO01000120
MAGSGGRNLDETPTWAVATVCFVLLSVSIIIEYIFNLIGKWLKQKHKRALYESLEKIKSELMLLGFISLLLTIGQGLISR
ICISENVAGTFHPCTYRREEKKYPPPLEDDYYHHNRHILAAEAGYDKCAAQGKVPFVSSGAIHQLHIFIFVLAVFHILYC
ILTLALGRAKMRRWKRWEVETKTAEYQYSHDPQRFRFARETSFGRRHLSFWTQHVVLVWIVCFFRQFVRSVPKVDYLTLR
HGFMMAHLGPHSHQKFDFRKYIKRSLEEDFKVVVEISPPIWFITVFFLLFNTHGWYSYLWLPFAPLIIVLLVGMKLQVII
TKMGQRIQERGEVVRGMPLVQPGDHLFWFNKPRLILYLINFVLFQNAFQLAFFSWAALQFMMKSCFLSQKKEVVIRISMG
IFVQFLCSYVTLPLYALVTQMGSTMKATIFNERVAMALRNWHHTAKKNVKEKRGLRSQSPLSTRPSTPKHPKSKANLLRR
YHSEMATYPSSPIRFDFEAHLPYGIHSPPSSKVNAAATSSIDQNEMEMEVEMEIDMDQVSDLTQHHIDIQDKQGFSFDKR

>VrMLO6 JJMO01000120
MEGRSLENTPTWAVAVVCFVLLSVSIIIEYIFNIIGKWLKNKHKRALHESLEKIKSELMLLGFISLLLTVGQSVISRICI
SEKVAGTFHPCTHQSDHKTDARILLSELFGSDHGQNQRRILAAGGTDKCAAQGKVPFVSSEGIHQLHIFIFVLAVFHVLY
CILTLALGRAKMRRWKRWEEETKTTQYQFSHDPERFRFARETSFGRRHLSFWTQNTVLVWIICFFRQFVRSVPKVDYLTL
RHGFMMAHLGPQSRHKFDFRQYIKRTLEEDFKVVVEISPPIWFITVLFLLFHTHGWYSYLWLPFAPLIIVLLVGTKLQVI
ITKMGQRIQERGEVVKGVPLVQPGDDLFWFNKPRLILYLVNFVLFQNSFQLAYFSWTALQFGMKSCFHSQTEDVVIKISM
GVFVQFLCSYVTLPLYALVTQMGSTMKPTIFNERVAMALRNWHHGAKKHVKQQNRRSQSQAPLSGPTTPDHSMSQAHLLR
RWHSEMDTYPTDSEAHRPCEIDLGPSSKVHHQEMEMDQLDHGPPQNVNDQTQHEINIEHGKEFSFDNR

>VrMLO7 JJMO01000121
MAGGASGGERTLQETPTWAVAAVCAVFVILSVLIEQGIHLLEKWFKKRHKKAMSEALEKIKAELMLLGFISLLLTFGTQY
IAKICIPASAGDIMLPCKKVEASKDPDDSNGGRKLLYFEDNMEWRRVLATASSGGDYCSQKGKVPLISQSGVHQLHIFIF
VLAVFHIFYSVMTMVLARAKMKKWKAWEAETSSLEYQFTNDPSRFRFAHQTSFVRRHSGWSRMPGIRWIVAFFRQFFASV
TKVDYMTMRHGFINAHFAPNSKFDFQKYIKRSMEDDFKVVVGISIPLWAFAIVFMLVNVYKWYTLTWLSLAPLVILLLVG
TKLELIIMEMAQEIQDRTTIVRGVPIVEPNNKYFWFNRPQWITFLIHFTLFENAFQIAFFLWTWYEFKITSCFHENLPLI
LTRVFLGIVLQVVCSYITFPLYSLVTQMGSHMKKAIFEEQTAKALKKWQRVAKDKRKLRKAGVDVPSGTMSGEATPSQGS
SPIHLLHKYKPNQTDTDTVLYSPRSYQSDTDLSDTEGSSYQLNEIIQTQTPQAPRNGEPHITEFSFVSFKSEQF

>VrMLO8 JJMO01000121
MSGGGGGDNRQLDLTPTWAVAAVCAIIVIISILLEKIIHKFAAVFEEKKKHALLEALEKIKAELMVLGFISLLLTFGQNY
ISKVCIPQRYAHTMLPCLPLEQRGGGAAAEHGAGGGEAEGGSHHRRLFSYERRVLSGGGGGPSCKPGFLPLISVSGLHQL
HIFIFFLAVFHVIYSAITMTLGRAKIRGWKAWEEDHVVDEDALNDPRRFRLTHETSFVRDHSSFWTKTPVSFYFVCFFRQ
FFRSVRRADYLTMRHGFVSVHLAPGSKFDFQKYIKRSLEDDFKVVVGISPILWGSVVLFLLVNVHGWHAAFWVSFLPLLV
ILAVGTKLQAIITRMALDIKERHAVVQGIPLVQVSDKYFWFAWPQLVLYLIHYVLFQNAFELTYFWWTWYEFGWASCFYE
DDKLMIVRVALGLGAQVVCSYVTLPLYALVTQMGSTMKKSIFDDQTSKALKQWHKNALKKKTSKGRQETRTLGGSPGEHT
PGHSSPHHPPPHKDTEMASQTATIVTSVDNETYDKRDLLSGP

>VrMLO9 JJMO01000121
MSGGGEEGESLEFTPTWVVAAVCTVIVAISLAAERFLHFGGKYLKAKDQKPLYEALQKIKEELMLLGFISLLLTVTQNGI
TKICVPPAFTRHMLPCSLDLAPKHESHFQTFYFPGTPRRLLAATAAEHAPPHSEAEATETGTETGFCARKNKVPLLSVEA
LHHLHIFIFVLAVVHVAFSVLTVVFGGARIRQWKHWEDSIAKKNYETDRVLKPKVTNVQQHDFIRGRFSGFGKDSAIVGW
MISFSKQFYGSVTKSDYVTLRHGFIMTHCKSNPKFNFHKYMIRALEDDFKQVVGISWYLWLFVVIFLLLNINGWHTYFWI
AFIPVILLLAVGTKLEHVITQLAHEVAEKHAAIEGDLVVQPSDEHFWFHRPRVFLFLIHFILFQNAFEIAFFFWIWVTYG

FDSCIMGQVRYIIPRLVIGVFIQVLCSYSTLPLYAIVTQMGTHYKRAIFNEHLQHNIVGWAQKAKKRKELKADGRPGQGS
SQESANTGIQLGSIFTKKASAPGDTSSAPKDDGTS

>VrMLO10 JJMO01000123
MSVSKYKILSLCLVSWLWCGYVAVASSESSIGSKDLDQTPTWAVACVCTVFILISLTLEKSLHKVGTWLHEKHKKALLEA
LEKVKAELMILGFLSLLLTFGQSYIVRICIPTDIADKLLPCPLAGTEEESSSEEEHRRKLLSYERRYLSGDATNYQCKKG
QQPLISVNGLHQLHILIFFLAVLHVFYSAVTMLLGRLKIRGWKAWEEETSSHGYEFANDPSRFRLTHETSFVRAHASFWT
RYSIFFYIGCFCRQFYRSVGKADYLALRNGFITVHLAPGSKFNFQKYIKRSLEDDFKVVVGVSPILWASFVVFLLLNVNG
WHAMFWASLIPVVIILAVGTKLQVALAKMAIEITERHAVVQGIPLVQGSDRYFWFGRPQLVLHLIHFALFQNAFQITYFL
WIWYSFGLKNCFHADYKLAIVKVALGLAALCLCSYITLPLYALVTQMGSRMKKSIFDEQTSKALKKWHMAVKKKQGVKLG
NSKVRAMDGSTTDSTVHSSGPKLHRFKTTGHSTRTMSAYDDDHSDIELSPVSPTANLIVRVDHDEHEAEEAEHHPTANNQ
ELSRLPTLERSMK

>VrMLO11 JJMO01000128
MGEEVLQRKLETTPTWAVAIVCFVMLAISILIEHIIEELGKWLKKKHKKALHEALEKVKGELMLLGFISLLLVVFEDRIT
SICISESVASTWNPCDPDYKSKGPEGYIDKCAQKDKGKVAFISEYGIHQLHIFVFVLAIFHILQCIMTLTLGRTKMSIWR
KWEDETKSLEHQFYHDPERFRFARDTTFGRRHLNSWSRSPISLWIVSFCRQFYGSVNKVDYMALRHGFIVAHLTPASETK
FDFQNYIKRTLDEDFAVVVGISPTIWFFAVLILLTNTNGWYSYLWLPFIPLIIILLVGTKLQMIITDMALRIQDRGEVVK
GAPVVEPGDELFWFSRPRLVLFLIHLVLFQNAFQLAFFAWSTYDNKFKINNCFHRTTEDIVIRLTMGVLTQVLCSYVTLP
LYALVTQMGSTMKPIIFKDNVASALKNWHHTAKKHLKHNKDSTSNTPFSSRPGTPTHGMSPVHLLHMHPRHSDSPLVSPR
ASNYENGQWDVEGSHSPSHHARDVDHEESMMMQPQAAELPPSGPNPIRTQHEINIALSEFSFGRGHNPSRADEY

>VrMLO12 JJMO01000129
MGGGGGGEEGNNLEFTPTWVVAVVCSVIVAASFAAERFLHYGGTFLKKKNQKPLYEALLKIKEELMLLGFISLLLTVTQN
GIIKICVPEGWTHHMLPCSLKDKKEKESAKLTEHFQTFFSFSHIPATVRHLLADNEDHQPTAAEKLGHCAKKGKVPLLSV
EALHHLHIFIFVLAIVHVTFCVLTVVFGGLKIRQWKHWENSIGDQINGTPDLESTVTHVREHAFIQNRFTGFGKESTVLG
WVKSFFKQFYGSVTKLDYVTLRLGFIMTHCRGNPKFNFHKYMIRALEDDFKKVVGISWYLWIFVVIFLLLNINGWHTYFW
ISFVPLILLLAVGTKLEHVIIQLAHEVAEKHTAIEGELVVQPRDDHFWFHRPHIVLFLIHFILFQNAFEIAFFFWILFTY
GFDSCIMGRVRYIIPRLVIGVFIQVLCSYSTLPLYAIVTQMGTHFKKAIFDEQVQARLVGWAQKAKKKGLRGDSNAQSAQ
GSAQVGAGIQLGAVFRRASAPEENVIVPTNEGHQ

>VrMLO13 JJMO01000320
MAEVLQQGRSLAETPTYSVASVVAVMVFVCLLVERSIYRFGEWLRKTRRKALFASLEKLKEELMLLGLISLLLAQSARWI
SEICVNSSLFSSRFYICSEQDLDINENIMLESSSSIPGETSIPKGLNTGAFNQCGEGHEPFVSYEGLEQLHRFLFVLGIT
HVLYSCLAVGLAMSKIYSWRRWENQARMPSDGNQQAKKIKLMKRQTTFIFHRTSHPWSRSPILNWMLCFLRQFRSSIRKS
DYLALRLGFITEHKLPLSYNFHQYMVRSMEDEFQGILGISWLLWIYAIVCIFVNIHGLNIYFWLSFIPAILVMLIGAKLQ
HVVSTLALEIMEQTGPFPRTQVKPRDDLFWFKKPDILLWLIQFVIFQNAFEMATFIWTLWGFQEKSCFMRNHYMIIIRLA
SGAIVQFWCSYMTVPLNIIVCQMGSRCKKALVAESVRESLHSWCKRVKHKSKHDSLHSHTARSVCSLESTIDERDEITVA
SGTLTRSSSLASLNQITVSSVDQLNFETPNKAKDSIKVAGCLSGSVHTNPPQPSHNVEDRVGNGEEAKAITLLDLFQKT

>VrMLO14 JJMO01000320
MAEELNHSLEYTPTWIVAVVCCIIVFISLCVERALHKLGNYLKRKGQKALYEVLTKLEEELMLLGFISLLLTVFQGLISE
ICISPNLATQMLPCKRPHRSSEGSAHYQIYYDAIINRRRLLSSGSGSDHCTHKGKVPLLSLESLHQLHIFIFVLAVVHAI
FCVTTMLLAGAKMREWKVWEDYYRDHSEAIDKHEFFKEKSRGHWRKAAVISWLMSFFKQFHGSVTKYDYYALRYGFIKKH
HSNNLKYNFHNYMLRTLEVDFKRVVGISWYLWVFVVLFLLLNIEGWHTYFWLAFLPLILLLLVGAKLEHIIARLYQDSVD
MLGRDDDKLVKPSDEYFWFTSPLLVLHLLHFILFQNSFEIAFFFWIWCTYGFDSCIMEKVAYVIPRLIMGVIVQVLCSYS
TLPLYTIVTQMGSEIKPQKLLSSDSLNTPHSNCMIKESTQTDEQAIIMVEDATSAIELVPLEKPNTSNS

>VrMLO15 JJMO01000319
MAASESGSSSRELDQTPTWAVAVVCTVFILISMALEKSLHKVGTWLGKKQKKALLEALEKVKAELMILGFISLLLTFGQS
YIVKICIPEKLADNMLPCPYKYKDAEKASDDGEKHRRKLLSYERRYLAADTTSFKCSNEGHEPLLSVNGLHQLHILIFFL
AVFHVLYSAITMLLGRLKIRGWKAWEAETSTNNFEFANAASRFRLTHETSFVRAHADLWTRIPIFFYIGCFFRQFYRSVT
KTDYQTLRNGFITVHLAPGSKFNFQKYIKRSFEDDFKVVVGVSPVLWASVVVYLLVNVNGWRTTLWAAAIPVVIILAVGT
KLQAILAKMAIEITDRHAVVQGIPLVQGSDKYFWFGQPQLVLHLIHFALFQNAFQVTYILWIWYSFGVRNCFRNDYKLAI
MKVALGIVMLCLCSYITLPLYALVTQMGSRMKKAIFDEQTNKALRNWHMAVKKKQRVVKLGKFNAESVDGSPAASSSAVH
PSGPTLHRFKTTGHSTRSQSYEDQDHEYESDVELSPELSQTASFIVRMDHADHQQLQEHEHSEEETNSSEEAEFSFKPSD
LVEKPPHSL

>VrMLO16 JJMO01000319
MGEENGEGRSLADTPTYAVATVITVLVSFSFLFQGTLKKLVKWLDRTKRKSLLSALEKIKEELMLFGLLSLLMGHWIIFV
AKICVKSSVISSRFFPCPLEKNSVKRVVWLSSEYSNRTVVKGQVNNGLRNYCPEGHESLASYESLEQLHRFVFVLGVTHV
TYSFLAVALAMIKIYSWRTWENEAKTIAVQSIQDTSQSTTSIRLRRLNTFVFHHTSHPWSHHKILVWLLCFSRQFWSSIH
RADYMALRLGFITNHDLPTTYDFHNYMLRSMDEEFRDIVGVSVLLWVYAICCIFLNFHGSNFYFWLSFVPAILILIIGTK
LHRVVVKLAVEIINRCPNMKPHQFNLRDELFWFGKPRFLLRLIQLISFLNAFEMATFLWSLWEIKDPSCFMNNRTFIVIR

LSFGVISQVWCSFITFPLYVIITQMGSRFKKTVVSENVRKSLSKWQRRVKERQSYSSALLTATSTASISSFICGNERGLM
TEEGSSSGTKDICLSYLDHVSRTQADASFPSYENDPHDIIHDSSPPLS

>VrMLO17 JJMO01000831
MAEEKIYKRSLEETPTWAFAVVCFVLLAISIIIEHVIHAIGKWFKKKHKSALYESLEKVKGELMMLGFLSMLLTVFQAPV
SKICISKNAASTWNPCSNPKAISNSNATSATIDRKLLQYLDPMPRRTLAAKSYDKCADQGKVAFVSAYGIHQLHIFIFVL
AIFHIIQCIVILALGRTKMRRWKKWEDETKTIEYEFYNDPERFRLAKDTTFGQRHLNTWSQSSISLRIVSFFRQFSGSVK
KVDYFALRHGFIMAHLAPGSDTRFDFHKYIKRSLDEDFKVVVGISPIIWFFVVLFLLTNTHGWYSYYWLPFVPLVTILIV
GAKLQMIITKMGLMIEDKGKVVKGAPVVEPGDDLFWFNRPRLLLFVIQFVLFQNAFQLAHFAWSIYEFSMKSCFHKTTAD
SMIRLTTGVVIQVLCSYVTLPLYALVTQMGSSLKPTIFNDRVAAALKKWHHTSKKHVKHRKHYEAKNFSPFSSKSSTPTV
GMSPIHLLHGHPAGRSDSARTSPRNSNYENEECDVQGESS

>VrMLO18 JJMO01001841
MDNNEQETRSLALTPTWSVATVLTVFVAVSLLVERSIHRLSNWLRKTNRKPLLAALEKMKEELMLLGFISLLLTATSRMI
ANICIPSKFYNSAFAPCTRSQIDEQMEENGSEKRKLWMASAYHVSGRMLNGINRSTCKEGYEPFVSYEGLEQLHRFIFVM
AVTHISYSCLTMLLAIVKIHSWRVWEDEAHMDRRDSLSEITRELTMRRQSTFVKSHLSNPLFKNSSLIWVLQTCFFRQFA
HSVVRADYLTLRKGFIMNHHLSLKYDFHSYMVRSMEEEFQRIVGVSGPLWGFVVGFMLFNIKGSNLYFWIAIIPVSLVLL
VGTKLQHVIATLALENAGIAGFFPEAKLRPRDELFWFNKPELLLSLIHFILFQNAFELASFFWFWWQFGYDSCFIRNHLL
LYLRLILGFAGQFLCSYSTLPLYALVTQMGTNYKAALIPQRIRETIHGWGKAARRKRKHGMFPDDSTIHTDTSTVLSIEE
DDQLIDAPEVELQPVNAVTSTPSPIANETSSRVATPLLRPSASISSAHSSSKAEVILRCSSMPSGR
